# Correlated Mutation in the Evolution of Catalysis in Uracil DNA Glycosylase Superfamily

**DOI:** 10.1038/srep45978

**Published:** 2017-04-11

**Authors:** Bo Xia, Yinling Liu, Jose Guevara, Jing Li, Celeste Jilich, Ye Yang, Liangjiang Wang, Brian N. Dominy, Weiguo Cao

**Affiliations:** 1 Department of Genetics and Biochemistry, Clemson University, Rooms 049 and 051 Life Sciences Facility, 190 Collings Street, Clemson, SC 29634, USA; 2 Department of Chemistry, Clemson University, 367 Hunter Laboratories, Clemson, SC 29634, USA

## Abstract

Enzymes in Uracil DNA glycosylase (UDG) superfamily are essential for the removal of uracil. Family 4 UDGa is a robust uracil DNA glycosylase that only acts on double-stranded and single-stranded uracil-containing DNA. Based on mutational, kinetic and modeling analyses, a catalytic mechanism involving leaving group stabilization by H155 in motif 2 and water coordination by N89 in motif 3 is proposed. Mutual Information analysis identifies a complexed correlated mutation network including a strong correlation in the EG doublet in motif 1 of family 4 UDGa and in the QD doublet in motif 1 of family 1 UNG. Conversion of EG doublet in family 4 *Thermus thermophilus* UDGa to QD doublet increases the catalytic efficiency by over one hundred-fold and seventeen-fold over the E41Q and G42D single mutation, respectively, rectifying the strong correlation in the doublet. Molecular dynamics simulations suggest that the correlated mutations in the doublet in motif 1 position the catalytic H155 in motif 2 to stabilize the leaving uracilate anion. The integrated approach has important implications in studying enzyme evolution and protein structure and function.

Cytosine (C) bases in DNA are prone to deamination to become uracil (U) bases^1^. Because U pairs with adenine (A) during DNA replication, G/C base pairs can be mutated to A/T base pairs due to deamination. The C to T transition mutation is a prominent genetic change^2^. Uracils in DNA are in general removed by uracil DNA glycosylase (UDG) through the base excision repair (BER) pathway^3^. The uracil DNA glycosylase (UDG) superfamily consists of six families with distinct enzymatic and repair properties. With the exception of family 6 hypoxanthine DNA glycosylases, families from 1 to 5 all contain uracil DNA glycosylase activity. Family 1 UNG stands out as an extraordinarily robust UDG that removes uracil from both double-stranded and single-stranded uracil-containing DNA^4^. The UDG activity in families 2, 3, 5 is orders of magnitude lower than family 1 UNG but can act on a variety of deaminated bases from hypoxanthine, a deamination product of adenine; to xanthine or oxanine, deamination products of guanine^5^,^6^,^7^,^8^,^9^.

Family 4 UDGa was initially discovered in the hyperthermophilic bacterium *Thermotoga maritima*^10^, then later in archaea^11^,^12^,^13^. UDGa from thermophilic bacterium *Thermus thermophilus* (Tth) can remove uracil *in vitro* and reduce mutation rates *in vivo*^14^,^15^. A crystal structure of Tth UDGa complexed with a uracil base has been solved, which indicates that family 4 enzymes adopt a similar structural fold as seen in other families within the UDG superfamily^16^. A distinct feature of the UDG superfamily is its catalytic diversity. Even though the catalytic motifs are conserved within a family, they diverge quite significantly among families (Fig. 1A). For example, the Asp residue in the third position of motif 1 in family 1 or the Asn residue in the equivalent position of motif 1 in family 2 is proposed to activate/position a water molecule to initiate nucleophilic attack at the glycosidic bond, however, this catalytic residue is missing in families 4, 5 and 6. Likewise, even though the His residue in motif 2 of family 1 is important for catalysis, it is absent in families 2 and 6. Thus, families within the UDG superfamily have gone on their own evolutionary paths to achieve catalytic diversity. How each family has evolved its own catalytic strategy is not understood.

In this study, we use family 4 UDG as a model to explore the evolutionary possibilities. Extensive mutational, enzyme kinetic analyses coupled with molecular modeling and molecular dynamics analyses have led to a model that relies on a histidine residue in motif 2 to stabilize a departing negatively charged uracilate anion. Mutual information (MI)-based computational analysis reveals that the E41 and G42 positions in motif 1 are highly correlated. Strikingly, a double substitution of E41-G42 by E41Q-G42D in motif 1 is able to rescue the detrimental effects of single substitution by one to two orders of magnitude. The experimental outcome is corroborated by molecular modeling and molecular dynamics analyses, which indicate that the correlated mutation of E41Q-G42D brings the catalytic histidine in motif 2 in a closer position to stabilize the leaving group. This study underscores the significance of correlated mutation in achieving enzyme catalytic efficiency and the power of mutual information analysis in uncovering evolutionary correlation. The methodology presented here has profound impact on understanding enzyme evolution and protein structure and function relationships.

## Results

### Catalytic mechanism of family 4 UDGa

**Figure 1:**
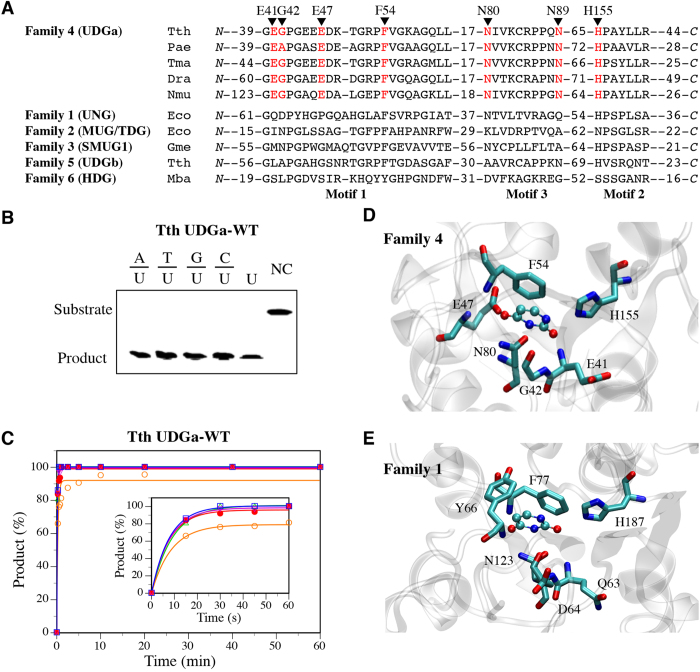
Sequence alignment, UDG activity and uracil binding pocket of Family 4 Tth UDGa. (**A**) Sequence alignment in family 4 UDGa and comparison with other UDG families. The alignment was based on BLAST and CLUSTALW analysis and constructed manually. Family 4 (UDGa): *Tth, T. thermophilus* HB27, YP_004341.1; *Pae, P. aerophilum str*. IM2, NP_558739.1; Dra, *D. radiodurans* R1, NP_295474 (DR 1751); Tma, *Thermotoga maritima* MSB8, NP_228321.1; Nmu, *Nitrosospira multiformis*, YP_412806. Family 1 (UDG): *Eco, E. coli*, NP_289138. Family 2 (MUG/TDG): *Eco, Escherichia coli*, P0A9H1. Family 3 (SMUG1): *Gme, Geobacter metallireducens* GS-15,YP_383069. Family 5 (UDGb): *Tth, Thermus thermophilus* HB8, YP_144415.1. Family 6 (HDG): *Mba, Methanosarcina barkeri* str. Fusaro, YP_304295.1. (**B**) DNA glycosylase activity of Tth UDGa on U-containing DNA substrates. Cleavage reactions were performed as described in Methods under DNA glycosylase activity assay. NC, negative control without addition of enzyme. (**C**) Time course analysis of DNA glycosylase activity of WT Tth UDGa on U-containing DNA substrates. (Δ) C/U; (•) G/U; (○) A/U; (∇) T/U; (□) single-stranded U. The assay was performed as described in Methods under DNA glycosylase activity assay except for that the reactions were quenched at specific time points as indicated. (**D**) Uracil binding pocket in the active site of Tth UDGa crystal structure (PDB code 1UI0). Uracil is colored by atom type. Amino acid residues interacting with the uracil are highlighted in licorice in program VMD. (**E**) Uracil binding pocket in the active site of *E. coli* UNG crystal structure (PDB code 1FLZ). Uracil is colored by atom type. Amino acid residues interacting with the uracil are highlighted in licorice in program VMD.

Family 4 UDGa is a distinct family in UDG superfamily with limited sequence homology with other families (Fig. 1A). Previous reports show that UDGa is a uracil DNA glycosylase that can act on both double-stranded and single-stranded uracil-containing DNA^11^,^12^,^14^,^17^. *Thermus thermophilus* family 4 UDGa exhibited robust glycosylase activity on all uracil substrates but did not show any detectable activity on other deaminated bases (Fig. 1B and data not shown). The robust UDG activity was further confirmed by a time course analysis (Fig. 1C). In an initial measurement, the reactions were largely completed within a minute. This was confirmed by a 60 sec time course analysis. The excision of all uracil-containing substrates except for the A/U base pair was essentially completed within 30 sec (Fig. 1C). Under the assay conditions, the rate constants for A/U, T/U, G/U, C/U and single-stranded U were estimated to be 6.5 × 10^−2^ s^−1^, 1.2 × 10^−1^ s^−1^, 1.2 × 10^−1^ s^−1^, 1.1 × 10^−1^ s^−1^ and 1.3 × 10^−1^ s^−1^, respectively. While enzymes in families 2, 3, 5 and 6 can excise other deaminated bases, it appears that family 4 UDGa has similar narrow substrate specificity as family 1 UNG.

The availability of crystal structures of both family 4 Tth UDGa and family 1 UNG allows a structural comparison of the uracil binding pockets^16^. In Tth UDGa, the uracil binding pocket is defined by E41, G42, E47, F54, N80 and H155 (Fig. 1D), whereas in family 1 *E. coli* (Eco) UNG, uracil is surrounded by Q63, Y66, F77, N123 and H187 (Fig. 1E). Specifically, N80 sidechain in Tth UDGa forms two hydrogen bonds to N3 and O4 of uracil (Figure S1A). H155 sidechain forms a hydrogen bond with O2 of uracil (Figure S1A). Likewise, N123 and H187 in Eco UNG form similar hydrogen bonds with the uracil (Figure S1B). To understand the importance of these structurally identified residues in binding and catalysis, we made a series of amino acid substitutions. N89 was also investigated because it is located in a structurally homologous position to an asparagine in the family 5 Tth UDGb, which has been shown to be catalytically important in that family^8^. The types of point mutations made ranged from highly conserved amino acid substitutions to alanine, and to others that might have appeared in other families. For example, E41 was changed to conserved Asp and Asn and to Ala. E41Q was made because it was a conserved change and because Gln appeared in family 1 UNG in this position (Fig. 1A). Initially, we screened the UDG activity of all 29 mutants using all five uracil-containing substrates (Table S1). The impairment on UDG activity varied depending on the positions and substitutions. The most severe reduction was at the H155 position while the least severe was at the N89 position. Other mutants also showed substantial effects on UDG activity. To more accurately quantify the mutational effects on binding and catalysis, we measured the kinetic constants for the wild type and selected mutants. Because the loss of catalytic activity was too great to allow use of conventional steady state kinetics, we adopted a kinetics method that was previously used for the study of noncognate sites in EcoRI and EcoRV restriction enzymes^18^,^19^. In the case that *k*_obs_ plateaus with increasing enzyme concentrations, *K*_m_ and *k*_2_ would be obtained (Figure S2A,B). In the case that *K*_m_ has increased to a degree that the plot of *k*_obs_ vs the total enzyme concentration is linear, only the *k*_2_/*K*_m_ would be determined (Figure S2C).

Based on the Tth UDGa structure complexed with a uracil base^16^, the mainchain NH of E41 interacts with the O2 of uracil. Substitution of E41 with Ala, Asp, Asn, and Gln all caused a substantial reduction in UDG activity, in particular for the A/U base pair and the single-stranded uracil-containing substrate. It is known that mainchain conformations can be significantly affected by side chain substitutions^20^,^21^,^22^. Kinetic measurements for the E41Q mutant showed that the *k*_2_/*K*_m_ was reduced by three orders of magnitude (Table 1). Similar effects were observed for substitutions in the adjacent G42 position. Interestingly, two substitutions with a carboxyl sidechain (G42D and G42E) were relatively more active than the other substitutions (Table S1). In family 1 UNG, the equivalent position is occupied by an Asp residue (Fig. 1A). The G42D mutant lowered the *k*_2_/*K*_m_ by two orders of magnitude (Table 1). E41Q and G42D also did not show binding affinity to U-containing DNA (data not shown), which was consistent with a rather large *K*_m_ as demonstrated in the kinetic analysis. An E47A substitution also caused a similar two orders of magnitude reduction in UDG activity on the G/U base pair (Table 1). The mutational effects on F54 depended on the nature of substitution. Whereas F54A and F54H had a significant effect on the UDG activity, the conserved change by replacement of F54 with the aromatic Tyr largely retained the UDG activity (Table S1). The loss of the aromatic sidechain caused a close-to-17-fold reduction in *k*_2_/*K*_m_ value (Table 1). N80 makes bidentate hydrogen bonds to the N3 and O4 of uracil (Fig. 1D and Figure S1A). Substitutions at N80 lowered the *k*_2_/*K*_m_ by over 40-fold (Table 1). N89A mutant reduced the UDG activity to a lesser degree and was one of the mutants that both *K*_m_ and *k*_2_ could be obtained (Figure S2B and Table 1). Whereas the *K*_m_ for N89A was slightly reduced as compared with the wild type enzyme, the *k*_2_ was reduced by almost 6-fold (Table 1). These results indicate a role of N89 in catalysis and will be discussed later. H155S exhibited its effects mostly on *k*_2_ while the *K*_m_ was only slightly reduced (Table 1). The *k*_2_ effect was much more profound than the N89A mutant, resulting in an over three orders of magnitude difference as compared with the wild type Tth UDGa (Table 1).

### Correlated mutations in motif 1

The robust and exclusive glycosylase activity on uracil-containing DNA prompted us to compare the sequences of family 4 UDGa and family 1 UNG closely. Whereas several important structural elements for the UDG function are highly conserved, a notable difference is that in motif 1 the E41-G42 doublet is replaced by Q63-D64 (Fig. 1A). The single mutations described above have already shown that substitutions in E41 and G42 are detrimental to the catalytic function of Tth UDGa. The conservation observed in the QD doublet of family 1 UNG enzymes led us to think of a possible correlation between these two residues, probably a result of co-evolution during UDG superfamily divergence.

The correlation was quantified using Mutual Information (MI), which in information theory is the measure or quantification of how much information one random variable provides about another random variable. In the study of protein co-evolution, the implementation of MI allows for studying the relationships present in the different positions within a protein family. In contrast with other methods, which focus on identifying the possible underlying co-evolutionary relationships within each of the sequences that compose the multiple sequence alignment file of a given protein family, MI methods use an inter-sequence approach^23^,^24^. This means that for a given position (x) in all the sequences in an alignment file, the amino acid distribution for x is determined using the entirety of sequences in the multiple sequence alignment file. Afterwards, the information generated for x is used to determine the amino acid distribution of another position, identified as y. This means that the information derived from x will determine the residue identity of y^24^,^25^. This intrinsic ability of MI to analyze the relationship between positions in a set of sequences has made it capable of determining co-evolutionary relationships not only amongst residues located closely to each other but also amongst residues that are spatially distant^23^,^24^.

**Figure 2:**
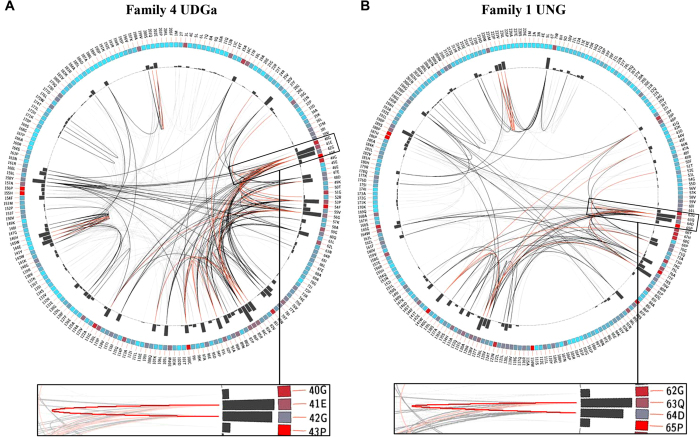
Circos diagrams of family 4 UDGa and family 1 UNG. The diagram contains the amino acid residue positions and residue identities mapped to the selected reference sequence. The square boxes below each residue represent the level of conservation ranging from red (highly conserved) to blue (less conserved). The bars in the histogram represent the co-evolutionary correlations form the mutual information analysis with a value higher than 6.5. The connecting lines between residue pairs follow a color scheme for ranking correlation between positions in the multiple sequence alignment where red indicate the top 5%, black between 95% and 70% and gray the remaining interactions. (**A**) Circos diagram of family 4 UDGa. The circular representation of the multiple sequence alignment using Tth UDGa as a reference sequence. (**B**) Circos diagram of family 1 UNG. The circular representation of the multiple sequence alignment using *E. coli* UNG as a reference sequence.

The details of computational methods were described in Methods and the results of MI analysis are presented in Fig. 2. For family 4 UDGa, the Circos diagram showed that amino acid positions 41 and 42 (among other positions shown as bars in Fig. 2A), corresponding to glutamic acid (E) and glycine (G) in the Tth UDGa sequence, were sites undergoing strong correlated mutations. Interestingly, the MI analysis for family 1 revealed that the residues in positions 63 and 64 of the *E. coli* UNG sequence were sites having strong co-evolutionary relationships (Fig. 2B). This finding is of special interest as these residues are part of the characteristic motif 1 that defines the UDG family 1 (Fig. 1A). In addition, other positions also show various degrees of correlations (Fig. 2). For example, this same pattern was observed in family 4 KCR triplet and family 1 LTV triple in motif 3 (positions 83–85 and 126–128 in family 4 and family 1 reference sequences, respectively). Previous studies in other protein families support the possibility that an underlying co-evolutionary relationship, shared by these neighboring residues, has shaped the amino acid composition of this motif, and thus the family’s activity and substrate preference^24^.

Inspired by the MI analysis, we replaced the EG doublet in family 4 Tth UDGa with the QD doublet in family 1 UNG. Indeed, the E41Q-G42D mutant was more robust than any of the single mutants (Tables 1 and 2). To quantitatively compare the catalytic efficiencies, we measured the kinetic constants. The *k*_2_/*K*_m_ of the Tth UDGa E41Q-G42D was only 5-fold lower than the wild type enzyme, resulting in ∆∆G of 1.1 kcal/mol (Table 2). In contrast, the ∆∆G values between the single mutant and the wt enzyme are 4.5 kcal/mol and 3.0 kcal/mol, respectively for E41Q and G42D (Table 2). The fact of ∆∆G_E41Q-G42D_ is much smaller than the sum of ∆∆G_E41Q_ and ∆∆G_G42D_ indicate a strong interaction between the two residues. The effect of the E41Q-G42D can not be simply attributed to the maintenance of a negatively charged Asp as E41G-G42E mutant was inactive (data not shown) and E41A-G42D mutant was still two orders of magnitude less active then E41Q-G42D (Table 1). Remarkably, the double mutant enhanced the catalytic efficiencies of E41Q and G42D by 167-fold and 17-fold, respectively. These results underscore the important structural and functional correlation of QD doublet in both family 1 UNG and family 4 UDGa.

**Figure 3:**
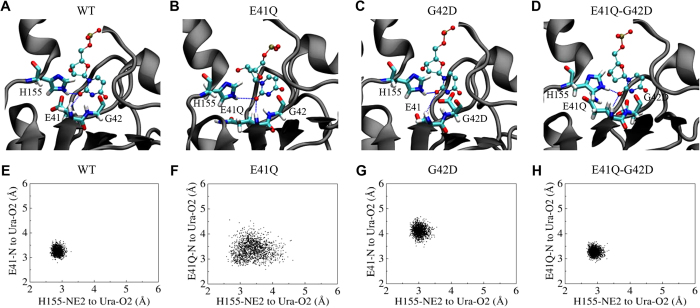
Interactions and two-dimensional scatter plots of the wild type and mutant Tth UDGa proteins with O2 of uracil in the active site. Modeled interactions with O2 of uracil in the active site of Tth UDGa-WT (**A**), Tth UDGa-E41Q (**B**), Tth UDGa-G42D (**C**) and Tth UDGa-E41Q-G42D (**D**). dUMP is colored by atom type. Amino acid residues in the active site of Tth UDGa are shown in licorice in program VMD. Hydrogen bonds are shown as dashed lines. Two-dimensional scatter plots of heavy atom distances between E41, H155 and uridine in Tth UDGa-WT (**E**), Tth UDGa-E41Q (**F**), Tth UDGa-G42D (**G**) and Tth UDGa-E41Q-G42D (**H**). The distances were obtained from MD trajectories in the modeled enzyme-DNA complexes.

The MI analysis to UDG superfamily reveals that QD of motif 1 in family 1 and EG of motif 1 in family 4 are highly correlated (Fig. 2). Remarkably, our experimental results show that changing the doublet EG in Tth family 4 UDGa to QD vastly improve the catalytic efficiency (Tables 1 and 2). Then, what is the underlying structural adjustment that results in such an improvement? To understand the structural and functional correlation between E41 and G42 positions in family 4 UDGa, we conducted molecular dynamics (MD) analysis. In the wild type enzyme, the average hydrogen bond distances between the mainchain of E41 and O2 of uracil and between the sidechain of H155 and O2 of uracil are 3.26 Å and 2.86 Å, respectively (Fig. 3A,E). The short distance between H155-NE2 to the O2 of uracil is suggestive of a strong hydrogen bond. E41Q mutation increased the distances between the O2 of uracil to the mainchain of E41Q and the sidechain of H155 to 3.38 Å and 3.39 Å, respectively (Fig. 3B,F). This change would substantially weaken the hydrogen bonds to O2, resulting in a large loss of UDG activity. The structural effect caused by the G42D mutation is more profound for the hydrogen bond distance between the uracil and the E41 than that between the uracil and H155, with the average distances as 4.12 Å and 3.03 Å, respectively (Fig. 3C,G). The concurrent change of E41Q and G42D, however, shortens the hydrogen bond distances between O2 of uracil and the mainchain of E41Q and between O2 of uracil and the sidechain of H155 to 3.27 Å and 2.91 Å, respectively, likening what is observed in the wild type Tth UDGa (Fig. 3D,H). The correlation between the QD doublet of motif 1 and the His residue of motif 2 is likely due to the fact that they both interact with O2 of uracil. The structural alignment of the two important hydrogen bonds brought about by E41Q-G42D doublet is in line with the large recovery of the lost UDG activity in individual amino acid change (Tables 1 and 2). These analyses suggest that these two positions are intrinsically correlated and the EG doublet or the QD doublet works in concert to exert its structural and functional impact on family UDGa.

## Discussion

**Figure 4:**
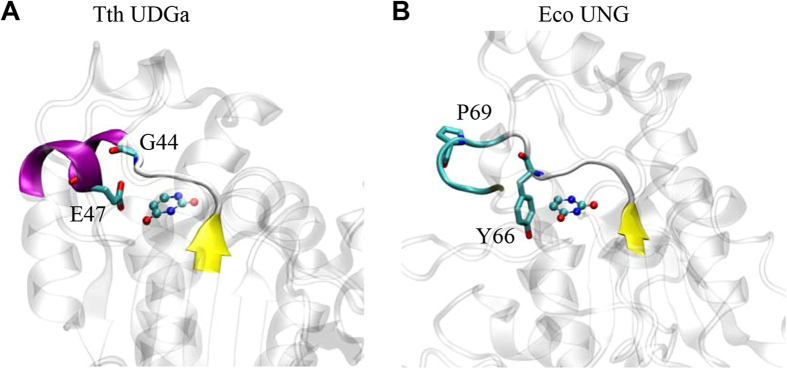
Comparison of E47 of Tth UDGa with Y66 of Eco UNG. (**A**) Amino acid residues 40–50 of Tth UDGa and uracil in the crystal structure (PDB code 1UI0). Uracil is colored by atom type. Amino acid residues are shown in licorice in program VMD. (**B**) Amino acid residues 62–72 of Eco UNG and uracil in the crystal structure (PDB code 1FLZ).

Family 4 UDGa enzymes are found in prokaryotes, while family 1 UNG enzymes are common in eukaryotes and bacteria. Consistent with a previous work^16^, data presented here indicate that family 4 UDGa is a glycosylase with a rather narrow substrate specificity. Despite its low sequence homology, the uracil binding pocket of family 4 UDGa shares some similar features as seen in family 1 UNG (Fig. 1D,E and 4A)^16^. As pointed out previously^16^, a distinctly different arrangement is E47 in Tth UDGa, which blocks the entry of thymine (Fig. 1D,E and 4A). In Eco UNG, Y66 plays a similar role in distinguishing uracil from thymine. The crystal structures complexed with uracil show that E47 in Tth UDGa and Y66 in Eco UNG are located in different structural contexts (Fig. 4). In Tth UDGa, the sidechain of E47 is brought into close proximity with C5 of uracil by an α-helix, while the sidechain of Y66 in Eco UNG is located in the loop facing the C5 of uracil (Fig. 4A,B). The helix structure does not seem possible with Eco UNG because the position equivalent to E47 is occupied by a highly conserved proline residue (P69) (Fig. 4B).

**Figure 5:**
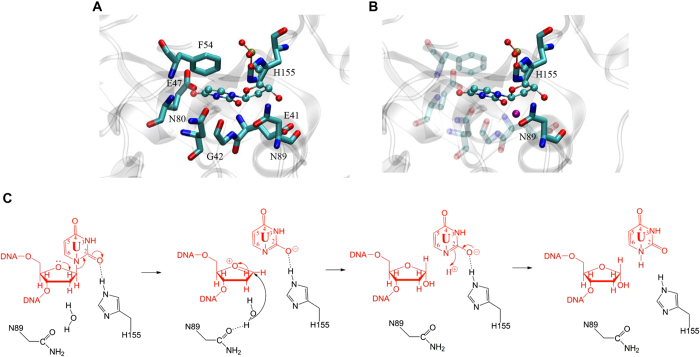
Modeled structure and proposed catalytic mechanism of Tth UDGa. (**A**) Modeled structure of Tth UDGa complexed with uracil-containing DNA in the energy minimized structure. The protein structure is shown in the background in light gray. dUMP is colored by atom type. Amino acid residues in the active site of Tth UDGa are shown in licorice in program VMD. (**B**) Interactions of N89 and H155 with dUMP in the modeled structure. The water molecule found in the modeled structure between N89 and the C1’ carbon is shown as a sphere in purple. (**C**) Proposed catalytic mechanism of family 4 Tth UDGa glycosylase. See text for details.

The cleavage of the N-glycosidic bond between the uracil and deoxyribose is achieved through the formation of an oxacarbenium ion intermediate and attacking of the anomeric carbon by a water molecule^26^,^27^,^28^. Activation of the leaving group, stabilization of the oxacarbenium ion and activation/positioning of water as a nucleophile may contribute to the catalysis. The catalytic mechanism underlying the hydrolysis of the N-glycosidic bond in family 4 UDGa is not understood. In family 1 UNG, a His residue (H187 in Eco UNG) in motif 2 can act as a general acid to stabilize the uracil leaving group and an Asp residue (D64 in Eco UNG) in motif 1 is proposed to activate a water molecule as a general base^29^,^30^,^31^. Part of the challenge in suggesting a catalytic mechanism for family 4 UDGa lies in the fact that the water-activating Asp residue in motif 1 of family 1 UNG is a small Gly or Ala residue in motif 1 of family 4 UDGa (Fig. 1A)^16^. This work implicates two residues as playing an important role in catalysis. Mutational effects at N89 and H155 positions are mainly at the catalytic step (Table 1). The four orders of magnitude change in *k*_*2*_ and *k*_*2*_/*K*_m_ by H155S substitution indicates that H155 in motif 2 is critical for catalysis. The contact made between the DE2-NH and O2 of uracil can stabilize the uracil leaving group, thus promoting the cleavage of the N-glycosidic bond (Fig. 5A,B). Similarly, H187 in Eco UNG makes a large contribution to transition state stabilization by forming a short distance hydrogen bond^30^,^32^. In the modeled structure, N89 in a sequence segment we now named motif 3 is located on the opposite site of the uracil relative to the deoxyribose (Figs 1A and 5B). In the sequence alignment shown in Fig. 1A, N89 corresponds to N120 in family 5 Tth UDGb. The kinetic analysis shows that N89 in Tth UDGa plays a significant catalytic role (Table 1). Previously, we proposed that N120 in family 5 Tth UDGb can contribute to catalysis by positioning a water molecule observed in the crystal structure^8^. In the *E. coli* MUG cocrystal structure, a water molecule is bound to N18^33^. It is proposed that the bound water molecule initiates the nucleophilic attack on the C1’ carbon. Analogously, we suggest that N89 in family 4 Tth UDGa can position a water molecule for attacking the anomeric carbon (Fig. 5B). The fact that family 4 Tth UDGa N89A mutant still retained some catalytic activity suggests that water positioning does not contribute to the catalytic power as much as the His residue in motif 2 for the UDG activity. Overall, we propose an SN1-like catalytic mechanism for the family 4 Tth UDGa, in which H155 stabilizes the uracil leaving group and N89 positions a water molecule for attacking the anomeric carbon (Fig. 5C).

In the UDG superfamily, families 1 UNG and 4 UDGa have some unique catalytic features as they share narrow substrate specificity and high catalytic efficiency. However, they are quite diverse in sequences and catalytic motifs. The analysis presented above highlights their distinct differences in catalytic mechanisms. The analysis of residue co-evolution in protein families has been described using different methods. Mutual information analysis appears to be the most common widespread method to identify evolutionary relationships between residues^23^. The MI of an amino acid position’s identity can be used to determine the identity of either a neighboring or distant position in the multiple sequence alignment. The amount of information that one variable provides regarding the other can be quantified, allowing for the establishment of information thresholds. Such boundaries can be used to define the significance of the information one variable provides about another.

The ability to determine evolutionary relationships amongst residues not found in the same domain or secondary structure provides an interesting approach to study the evolution of protein functionality^24^,^25^. By identifying co-evolving residues, it is possible to understand the role of balancing mutations in distant residues^23^,^34^,^35^. In such cases, an amino acid change in a non-critical, spatially distant site could buffer the effect of a mutation in a critical site by rescuing or maintaining the enzyme’s activity. In addition, MI theory-based methods have been used to explain the possible interactions found amongst neighboring residues. It appears that certain residues, which are close to each other due to their position in the protein and its secondary structure, could be the subjects of mutations to balance the effects of a change in their neighbor that could lead to a deleterious effect^24^. During evolution, these deleterious effects were purged by natural selection.

In summary, this study reveals divergent evolutionary paths to define substrate specificity and catalytic efficiency in UDG superfamily. While both families 1 and 4 glycosylases use histidine-mediated transition state stabilization for the cleavage of the N-glycosidic bond, they differ by how to activate/position a water molecule for attacking the anomeric carbon. While family 1 UNG enzymes possess a conserved Asp residue in motif 1 to activate a water molecule for in-line nucleophilic attack on the C1’ carbon, family 4 UDGa enzymes rely on a Asn residue in motif 3 to position a water molecule. This may in part explain why family 1 UNG is highly efficient. Furthermore, family 4 enzymes distinguishes themselves from family 1 enzymes by using a Glu residue, rather than a Tyr residue to define a tight uracil binding pocket. Apparently, co-evolution plays an important role in the divergence of UDG superfamily. The application of mutual information theory, coupled with experimental and molecular dynamics analyses underscores a powerful integrated approach to understanding enzyme evolution, catalysis and structural and functional diversity.

## Methods

### Reagents, media and strains

All routine chemical reagents were purchased from Sigma Chemicals (St. Louis, MO), Fisher Scientific (Suwanee, GA), or VWR (Suwanee, GA). Restriction enzymes, Phusion DNA polymerase, and T4 DNA ligase were purchased from New England Biolabs (Beverly, MA). Bovine serum albumin and dNTPs were purchased from Promega (Madison, WI). Gel DNA recovery Kit was purchased from Zymo Research (Irvine, CA). Oligodeoxyribonucleotides were ordered from Integrated DNA Technologies Inc. (Coralville, IA) and Eurofins Genomics (Huntsville, AL). The LB medium was prepared according to standard recipes. Hi-Di Formamide and GeneScan 500 LIZ dye Size Standard for ABI3130xl were purchased from Applied Biosystems. The *Tth* UDGa sonication buffer consisted of 20 mM Tris-HCl (pH 7.5), 1 mM ethylenediaminetetraacetic acid (EDTA) (pH 8.0), 2.5 mM DTT, 0.15 mM PMSF, and 50 mM NaCl. The GeneScan stop buffer consisted of 80% formamide (Amresco, Solon,OH), 50 mM EDTA (pH 8.0), and 1% blue dextran (Sigma Chemicals). The TE buffer consisted of 10 mM Tris-HCl (pH 8.0) and 1 mM EDTA.

### Cloning, expression and purification of Tth UDGa

The uracil DNA glycosylase gene from *T. thermophilus* HB8 (TtUDGA) (GenBank accession number: AB109239.1) was amplified by PCR using the forward primer Tth UDGaF (5′ TCG TATGTCCATATGACCCTGGAACTGCTTCAGGC-3′ (NdeI)) and the reverse primer Tth UDGaR (5′ ATCGTACTCGAGGAAGAGGGGCTCCTGGC TCACC-3′ (XhoI)). The PCR reaction mixture (20 μl) consisted 10 ng *T. thermophilus* HB8 genomic DNA, 500 nM forward and reverse primers, 1x phusion polymerase buffer, 200 μM each dNTP and 0.2 unit of Phusion polymerase (New England Biolabs). The PCR procedure included a pre-denaturation step at 98 °C for 30 s; 30 cycles of three-step amplification with each cycle consisting of denaturation at 98 °C for 15 s, annealing at 60 °C for 15 s, and extension at 72 °C for 20 s; and a final extension step at 72 °C for 10 min. The PCR product was purified and cloned into pET21a vector. The recombinant plasmid was confirmed by DNA sequencing.

Site-directed mutagenesis was performed by using an overlapping extension PCR procedure similarly as previously described^7^. Taking the mutant E41Q as an example: The first round of PCR was carried out using plasmid pET21a-Tth-UDGa as template DNA with two pairs of primers, Tth-UDGaF and E41QR (5′-CTCCTCCCCGGGG CCCTGCCCCACGATCATGAGCT-3′) pair; E41QF (5′-CTCATGATCGTGGGGCAG GGCCCCGGGGAGGAGGA-3′) and Tth-UDGaR pair. The PCR products were electrophoresed on 1% agarose gel and the expected PCR fragments were purified from gel slices by Gel DNA Clean Kit. The second run of the PCR reaction mixture (20 μL), which contained 1 μl of each of the first run PCR fragments, 200 μM dNTPs, 1 × Phusion DNA polymerase buffer, and 0.2 units of Phusion DNA polymerase (New England Biolabs), was initially carried out with a pre-denaturation step at 95 °C for 30 s; 5 cycles with each cycle of denaturation at 98 °C for 15 s, annealing at 60 °C for 15 s, and extension at 72 °C for 30 s; and a final extension at 72 °C for 5 min. Afterward, 500 nM of outside primers (Tth-UDGaF and Tth-UDGaR) was added to the above PCR reaction mixture. The subsequent overlapping PCR amplification included a pre-denaturation step at 98 °C for 15 s; 30 cycles with each cycle of denaturation at 98 °C for 15 s, annealing at 60 °C for 15 s, and extension at 72 °C for 30 s; and a final extension at 72 °C for 10 min. Subsequent molecular cloning procedures were performed as previously described. The purified PCR products digested with a pair of BamHI and XhoI endonucleases were ligated to the cloning vector pET21a treated with the same pair of restriction endonucleases. The recombinant plasmids containing the desired mutations were confirmed by DNA sequencing and transformed into *E. coli* strain BL21 (DE3).

The pET21a-Tth-UDGa was transformed into *E. coli* strain BL21 (DE3) by the standard protocol to express the C-terminal His-6-tagged Tth UDGa protein. Briefly, the protein was induced by 0.5 mM IPTG at 16 °C for 12 h. After sonication and purification, fractions (300–400 mM imidazole, 60–80% chelating buffer B) containing the Tth UDGa protein as seen on 12.5% SDS-PAGE were pooled and concentrated by Amicon YM-10 (Millipore). The concentration of Tth UDGa protein was determined by SDS-PAGE analysis using bovine serum albumin as a standard and confirmed by measuring absorption at A_280_. The protein was stored in aliquots at −80 °C. Prior to use, the protein was diluted with 2 × storage buffer (20 mM Tris-HCl pH8.0, 2 mM DTT, 2 mM EDTA, 400 μg/ml BSA, 100% Glycerol).

### Oligodeoxynucleotide substrates

Oligodeoxynucleotides containing deoxyuridine (U), deoxyinosine (I), deoxyxanthosine (X) or deoxyoxanosine (O) were obtained or constructed as previously described^7^. The sequences of the U-containing DNA susbtrates are 5′-TA CCC CAG CGT CTG CGG TGT TGC GTN AGT TGT CAT AGT TTG ATC CTC TAG TCT TGT TGC GGG TTC C-3′/3′-GGG GTC GCA GAC GCC ACA ACG CAY TCA ACA GTA TCA AAC TAG GAG ATC AGA ACA ACG CCC-FAM-5′, where N=A, T, G, C and Y=U.

### DNA glycosylase activity assay

DNA glycosylase cleavage assays for *Tth* UDGa were performed under optimized reaction conditions at 60 °C in a 10 μl reaction mixture containing 10 nM oligonucleotide substrate, 100 nM glycosylase, 20 mM Tris-HCl (pH 7.6), 100 mM KCl, 1 mM DTT, and 1 mM EDTA. After 60 min incubation, the resulting abasic sites were cleaved by incubation at 95 °C for 5 min after adding 1 μl of 1 M NaOH. Samples for ABI 377 sequencer (Applied Biosystem) were prepared by mixing equal volume of GeneScan stop buffer and reaction mixture. After incubation at 95 °C for 5 min, 3.5 μl samples were loaded into 10% denaturing polyacrylamide gel. Electrophoresis was conducted at 1500 V for 1.5 h using the ABI 377 sequencer. Cleavage products and remaining substrates were quantified using the GeneScan analysis software. Samples for ABI 3130xl sequencer (Applied Biosystems) were prepared by mixing 2 μl of reaction mixture with 7.8 μl Hi-Di Formamide and 0.2 μl GeneScan 500 LIZ Size Standard. A total of 10 μl sample was loaded into ABI 3130xl and run with a fragment analysis module. Cleavage products and remaining substrates were analyzed by Gene Mapper.

### Enzyme kinetic analysis

Uracil DNA glycosylase assays were performed at 60 °C with 20 nM G/U substrates with enzyme in excess ranging from 100 nM to 3200 nM. Samples were collected at 2 s, 5 s, 10 s, 30 s, 1 min, 2.5 min, 5 min, 10 min, 15 min, 25 min, 30 min, 40 min and 60 min. The apparent rate constants for each concentration were determined by curve fitting using the integrated first-order rate eq. (1):


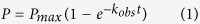


where P is the product yield, P_max_ is the maximal yield, t is time and *k*_*obs*_ is the apparent rate constant.

The kinetic parameters *k*_*2*_ and *K*_m_ were obtained from plots of *k*_*obs*_ against the total enzyme concentration ([E_0_]) using a standard hyperbolic kinetic expression with the program GraphPad 4.1 following the equation (2)^18^


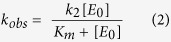


For some mutants with a large *K*_m_ in which *K*_m_ ≫ [E_0_], the kinetic parameter *k*_*2*_/*K*_m_ values were obtained from plots of *k*_*obs*_ against total enzyme concentration ([E_0_]) using a linear regression with program GraphPad 4.1 following the equation (3)^19^.


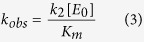


### Dataset acquisition and construction for mutual information analysis

The first step was to obtain the available uracil DNA glycosylase (UDG) sequences. These were acquired from UniProtKB^36^. A general search was done in order to find significant hits that could be used to generate a raw dataset, composed of representatives of all the UDG families. Subsequently, the raw dataset was sorted using a Perl script designed to separate sequences based on the presence of the distinct UDG family 4 and 1 motifs (GE[A/G][V/P]G and GQDPY, respectively), as reported previously^37^. The output of the script was two distinct files, each containing approximately 1000 family 4 UDGa and family 1 UNG protein sequences.

The resulting sequence files were then subjected to sequence clustering using BlastClust to reduce redundancy^38^,^39^. This is of special importance, as it reduces sequence redundancy as well as the bias effect that overrepresented sequences might have later on the residue co-evolution analysis^40^,^41^. The parameters used were: sequence similarity threshold of 75% and coverage percentage value of 85%. The selection of these parameters reduced the amount of highly similar sequences with different accession entries from each dataset. This resulted in a significant reduction of sequence entries, with each data file containing approximately 200 sequence representatives for each family.

### Multiple sequence alignment

The sequences in the dataset files were then aligned using the ClustalW alignment tool incorporated in MEGA6^42^,^43^,^44^. Preliminary alignments were performed using the default parameters followed by manual curation of the alignment. The process eliminated noise from outliers that lack typical motifs in family 4 UDGa or family 1 UNG. After the first alignment was completed, all gaps were removed from the datasets. A new alignment was performed using MEGA6’s implementation of ClustalW. The parameters were the following: the substitution matrix was BLOSUM62^45^,^46^, with gap opening penalty of 20 and gap extension penalty of 5. These stringent parameters were selected in order to reduce the number of gaps within the alignment. After repeating the process one more time, the ClustalW alignment output was refined using MUSCLE^47^ (The multiple sequence alignment file is provided in Supplementary Information).

### Mutual information analysis

Residue co-evolution was determined using a mutual information-based tool, MISTIC (Mutual Information Server to Infer Co-evolution). This approach uses mutual information to determine the evolutionary relationship between two residue positions in a multiple sequence alignment file. The calculation of the MI co-evolution values on the MISTIC server was carried out as described^48^. This consisted of calculating the frequency of amino acid pairs by means of weighting and low count correction. The calculated frequency is then compared with the expected frequency. MISTIC also assumes that mutations between amino acids are uncorrelated^48^. Afterwards, the MI scores for the protein family alignment were calculated. These scores were obtained by calculating a weighted sum of the log ratios of the expected and the observed frequencies from the amino acid pairs^48^. Mutual information background signal noise was corrected by implementing the Average Correction Product^48^,^49^. Subsequently, a Z-score normalization was applied to the MI values. A threshold of 6.5 was used to report co-evolving residues identified by MI. This value has been reported to have significant values in specificity and sensitivity^48^.

### Identification and visualization of co-evolving residues

The curated sequence alignment files were uploaded into the MISTIC web server, and a reference sequence for each family was selected. The selection was based on two criteria: being the representative of the largest cluster and second, having a similarity of more than 25% with the family’s canonic structure. This allows for the reference to be a significant representative of each of the UDG families studied.

After alignment file uploaded and reference sequences selected, the next step was modification of the default web server parameters. The protein structure file was left blank, as our analysis was intended to identify co-evolving relationships using only protein sequences. Within the advanced options the maximum fraction of gaps per column allowed in the calculations was set from 0.5 (default value) to 0.3. This number was selected in previous co-evolution analysis, and has yielded good results^41^. Finally, the file was submitted to the web server for analysis.

After the analysis was completed, results were visualized using the tools incorporated within the MISTIC web server. A sequential circular representation of the multiple sequence alignment, known as the Circos diagram, maps the amino acid positions to the reference sequence. In this diagram, there are three major components represented with a color scale. The first is the square boxes under each amino acid position, these boxes represent the conservation of the residue. The colors of these boxes range from red (highly conserved) to blue (less conserved). The second is the histograms associated with each position representing the cumulative mutual information, which illustrates the correlation a given residue has with other positions. The higher the histogram, the more residues that position is correlated with. Finally, the edges or lines connecting the co-evolving residues describe the relationship between positions in the multiple sequence alignment based on their mutual information. Red lines represent the top 5%; black lines refer to the MI relationships between 95% and 70%; and the gray lines denote the remaining MI relationships^48^.

### Molecular modeling

The crystal structure of TthUDGa and product complex was acquired from the RCSB Protein Data Bank (accession code 1UI0), and used as a model for subsequent computational analysis. A structure of DNA with a flipped-out uracil base analog was extracted from the crystal structure of human UNG-DNA complex (PDB accession code 1EMH)^50^ using the Swiss-Pdb Viewer (SPDBV) program^51^. The family 4 apo structure of TthUDGa (1UI0) was superimposed upon the family 1 1EMH crystal structure (bound to DNA) using the TopMatch server^52^. Removing the 1EMH protein coordinates resulted in a model of TthUDGa bound to DNA. Mutants E41Q, G42D and E41Q/G42D of TthUDGa complexed with DNA were also made using the mutation tool in the Swiss-Pdb Viewer program and the “best rotamer” was chosen with the lowest clash score.

### Molecular dynamics simulations

After building the initial complex structures, an explicit solvent system using the TIP3P water model was constructed in the CHARMM c35b6 molecular mechanics package^53^ using a suitably sized box. The minimum distance between any of the atoms of the solvated TthUDGa-DNA complex and the box boundary was maintained to at least 9 Å. Sodium chloride ions were added to the system to achieve an electrically neutral system. The CHARMM 27 all hydrogen force field for proteins^54^ and nucleic acids^55^ were used. Particle-mesh Ewald summation^56^ was applied in the periodic boundaries condition for the efficient calculation of long-range electrostatic interactions. Energy minimization was performed by using 4000 steepest descent steps followed by adopted basis Newton-Raphson (ABNR) method with harmonic constraints decremented from 10 to 1 kcal/(mol·Å^2^) in decrements of 3 kcal/(mol·Å^2^) every 1000 steps to remove any unfavorable van der Waals clashes while minimally perturbing the original model x-ray structure. Using a Langevin barostat^57^, an isothermal-isobaric ensemble (NPT) was constructed in NAMD program^58^ and the system was heated gradually from 100 K to 300 K over a period of 400 ps. An integration time step of 1fs was used in order to avoid any significant structural deformation during heating, equilibration, and production runs. Coordinates were saved every 2 ps. A total of 2 ns equilibration and 3 ns production simulation were performed for each structural analysis. RMSD analysis indicated that the simulations were stabilized within 2 ns (Figure S3). VMD 1.9.1^59^ had been used for visualization purposes.

## Additional Information

**How to cite this article:** Xia, B. *et al*. Correlated Mutation in the Evolution of Catalysis in Uracil DNA Glycosylase Superfamily. *Sci. Rep.* **7**, 45978; doi: 10.1038/srep45978 (2017).

**Publisher's note:** Springer Nature remains neutral with regard to jurisdictional claims in published maps and institutional affiliations.

## Supplementary Material

Supplementary Information

## Figures and Tables

**Table 1:** Kinetic constants of Tth UDGa on G/U substrate^a^.

Enzymes	*K*_m_ (M)	*k*_2_ (s^−1^)	*k*_2_/*K*_m_ (s^−1^ M^−1^)
Wild type	9.7 (2.4) × 10^−7^	2.3 (0.1) × 10^−1^	2.4 × 10^5^
E41Q	N.D.^b^	N.D.	2.7 × 10^2^
G42D	N.D.	N.D.	2.7 × 10^3^
E47A	N.D.	N.D.	2.0 × 10^3^
F54A	N.D.	N.D.	1.4 × 10^4^
N80A	N.D.	N.D.	5.5 × 10^3^
N89A	7.4 (1.9) × 10^−7^	2.5 (0.3) × 10^−2^	3.5 × 10^4^
H155S	7.6 (2.9) × 10^−7^	7.2 (1.0) × 10^−5^	9.4 × 10^1^
E41A-G42D	N.D.	N.D.	4.3 × 10^2^
E41Q-G42D	1.7 (0.2) × 10^−7^	7.5 (0.2) × 10^−3^	4.5 × 10^4^

^a^The reactions were performed as described in Methods under enzyme kinetic analysis. Data are an average of three independent experiments. SD values are shown in parentheses.

^b^Not determined. Individual *K*_m_ and *k*_2_ values were not determined due to a relatively large *K*_m_.

**Table 2:** Enhancement of Tth UDGa E41Q-G42D double substitution on UDG activity and free energy^a^.

Enzyme	Substrate	*k*_2_/*K*_m_ (s^−1^ M^−1^)	Activity Change (fold)^b^	Fold Enhancement over Single Substitution^c^	∆∆G (kcal mol^−1^)^d^
Wild Type	G/U	2.4 × 10^5^	1		
E41Q		2.7 × 10^2^	888	167	4.5
G42D		2.7 × 10^3^	89	17	3.0
E41Q-G42D		4.5 × 10^4^	5.3		1.1

^a^The reactions were performed as described in Methods. Data are an average of three independent experiments.

^b^Activity change was calculated by the ratio of *k*_2_/*K*_m_ of the wild type to *k*_2_/*K*_m_ of a mutant.

^c^Fold enhancement over single substitution was calculated by the ratio of of *k*_2_/*K*_m_ of E41Q-G42D to *k*_2_/*K*_m_ of single mutant.

^d^∆∆G was calculated using ∆∆G = −*RT*ln[(*k*_*2*_*/K*_*m*_)_mutant_/(*k*_*2*_*/K*_*m*_)_wild type_].
