# Supplementary material for: Correlated Mutation in the Evolution of Catalysis in Uracil DNA Glycosylase Superfamily
Source: Sci Rep. 2017 Apr 11;7:45978. doi: 10.1038/srep45978 (PMC5387724; doi:10.1038/srep45978)
Supplement: Supplementary Information [file srep45978-s1.pdf]

Supplementary Information for

Correlated Mutation in the Evolution of Catalysis in Uracil DNA Glycosylase Superfamily

Bo Xia[1], Yinling Liu[2], Jose Guevara[1], Jing Li[1], Celeste Jilich[1], Ye Yang[1],

Liangjiang Wang[1], Brian N. Dominy[2] and Weiguo Cao[1,*]

[1]Department of Genetics and Biochemistry, Clemson University
Rooms 049 and 051 Life Sciences Facility
190 Collings Street
Clemson, SC 29634
USA

[2]Department of Chemistry, Clemson University
367 Hunter Laboratories
Clemson, SC 29634
USA

* Corresponding author: wgc@clemson.edu; Tel.: (864) 656-4176; Fax: (864) 656-6879.

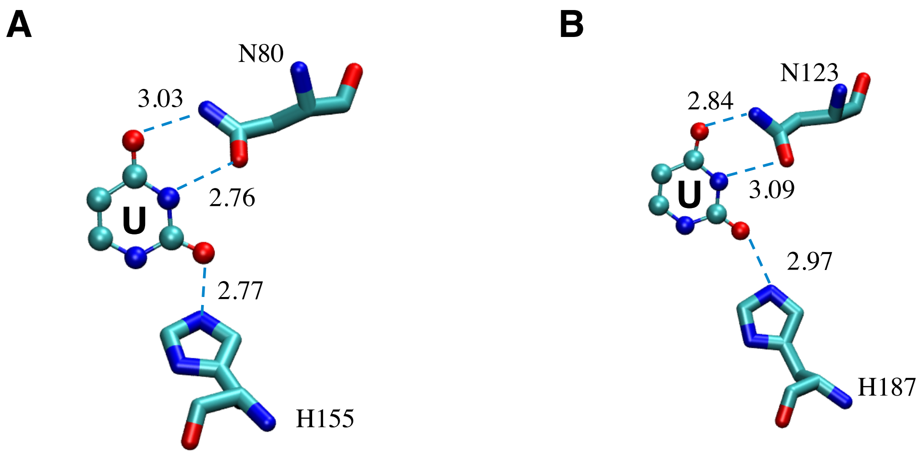

Figure S1. Interactions with uracil base in the active site. **A**. Family 4 Tth UDGa and uracil. Hydrogen bonding distances (Å) between N80 and H155 are shown as dashed lines based on PDB 1UI0. **B**. Family 1 Eco UNG and uracil. Hydrogen bonding distances (Å) between N123 and H187 are shown as dashed lines based on PDB 1FLZ.

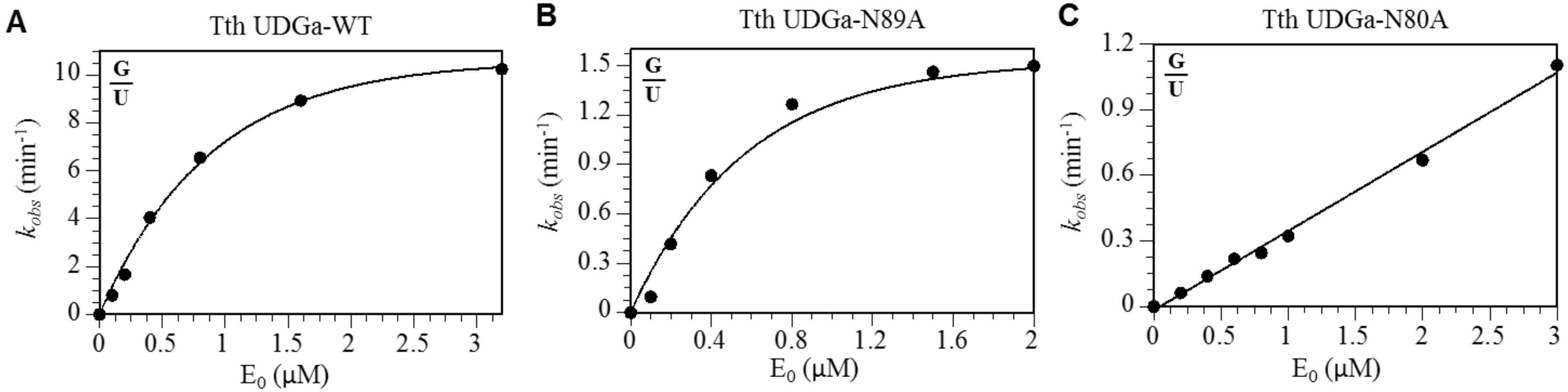

Figure S2. Representative kinetic analysis of the wild type and mutant Tth UDGa glycosylase. See Enzyme kinetic analysis in Methods for details. **A**. Tth UDGa-WT. **B**. Tth UDGa-N89A. **C**. Tth UDGa-N80A.

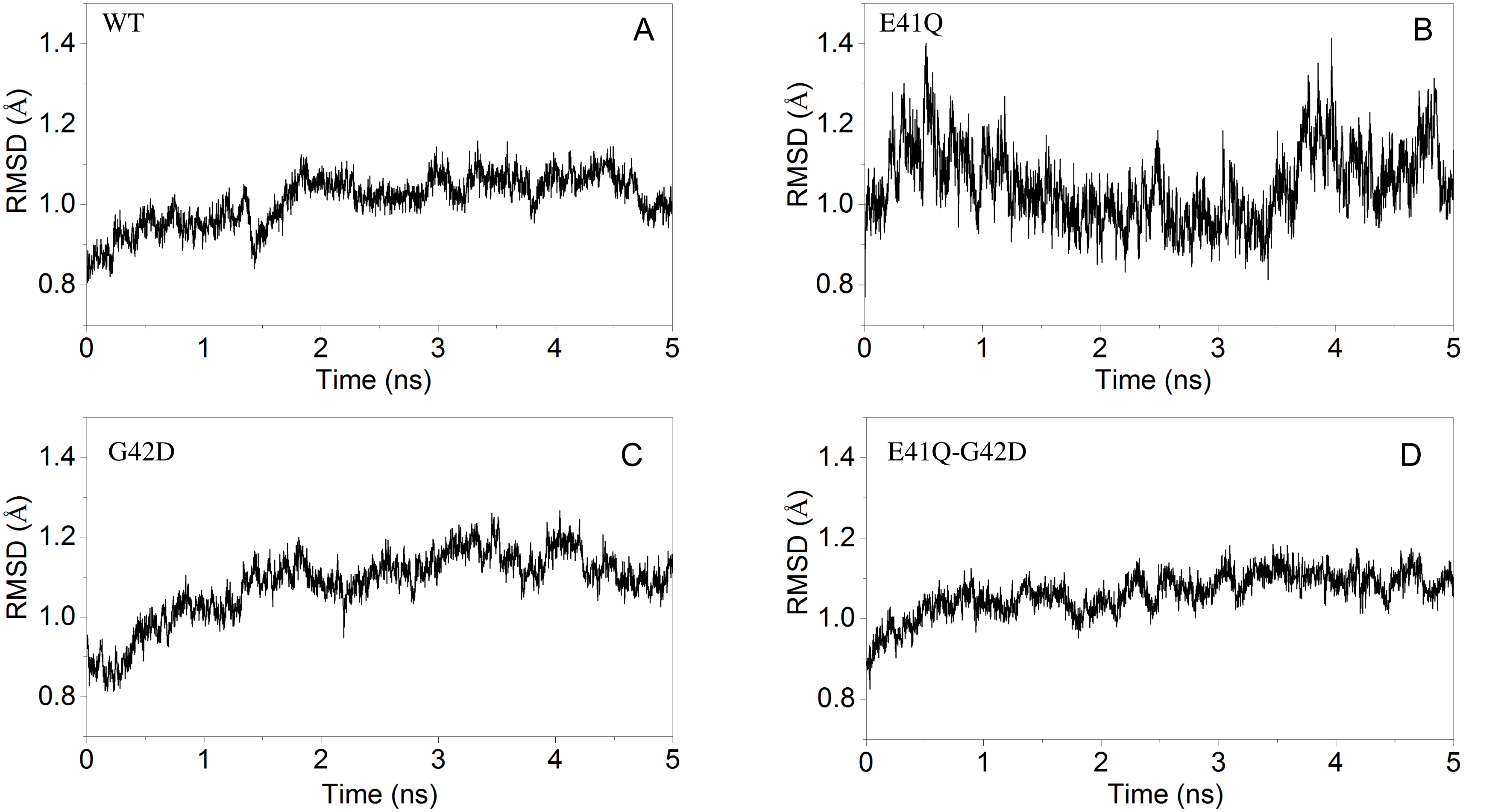

Figure S3. Root mean square deviations (RMSDs) of the backbone atoms as a function of the simulation time. A. WT. **B.** E41Q. **C.** G42D. **D.** E41Q-G42D.

Table S1. Relative glycosylase activity of Tth UDGa on uracil substrates[a]

Enzymes	A/U	T/U	G/U	C/U	U
WT	100 ± 0	100 ± 0	100 ± 0	100 ± 0	100 ± 0
E41A	1 ± 1.2	15 ± 0.1	10 ± 0.5	30 ± 0.4	10 ± 0.6
E41D	0 ± 0	4 ± 0.3	0 ± 0.8	9 ± 0.3	0 ± 0
E41N	1 ± 1.4	34 ± 0.7	18 ± 0.4	62 ± 8.3	15 ± 0.3
E41Q	0 ± 0	19 ± 0.6	10 ± 0.5	29 ± 0.9	5 ± 0.4
G42D	2 ± 1.4	33 ± 8.9	14 ± 3.6	64 ± 7.2	6 ± 3.6
G42E	0 ± 0	12 ± 3.6	8 ± 3.6	34 ± 3.6	0 ± 0
G42H	0 ± 0	0 ± 0	0 ± 0	0 ± 0	0 ± 0
G42L	0 ± 0	0 ± 0	0 ± 0	0 ± 0	0 ± 0
G42N	0 ± 0	1 ± 1	0 ± 0.6	2 ± 2.8	0 ± 0
E47A	0 ± 0	32 ± 0.6	10 ± 0.1	57 ± 0.5	1 ± 1
E47D	0 ± 0	1 ± 1.6	0 ± 0	4 ± 3.8	0 ± 0
E47N	0 ± 0	0 ± 0	0 ± 0	0 ± 0	0 ± 0
E47Q	0 ± 0	0 ± 0	0 ± 0	0 ± 0	0 ± 0
E47S	0 ± 0	0 ± 0	0 ± 0	0 ± 0	0 ± 0
F54A	6 ± 5	44 ± 3.7	13 ± 3.6	61 ± 8.9	16 ± 3.7
F54H	0 ± 0	40 ± 9	10 ± 3.6	57 ± 8.9	7 ± 5.6
F54Y	77 ± 3.6	98 ± 1.9	98 ± 2.2	99 ± 1.5	96 ± 3.4
N80A	11 ± 6.5	52 ± 1.8	21 ± 2.1	85 ± 6.1	19 ± 6.4
N80Q	1 ± 1.2	6 ± 1.5	2 ± 1.5	14 ± 1.5	0 ± 0
N89A	12 ± 1.2	99 ± 1.2	82 ± 0.2	100 ± 0	44 ± 0.9
N89D	5 ± 0.2	96 ± 0.4	70 ± 0.4	100 ± 0	36 ± 2.8
N89E	0 ± 0	45 ± 0.4	16 ± 0.3	65 ± 0.2	5 ± 0.2
N89Q	19 ± 0.6	90 ± 0.5	83 ± 0.4	95 ± 0.7	32 ± 0.2
H155A	0 ± 0	0 ± 0	0 ± 0	0 ± 0	0 ± 0
H155F	0 ± 0	0 ± 0	0 ± 0	0 ± 0	0 ± 0
H155M	0 ± 0	0 ± 0	0 ± 0	0 ± 0	0 ± 0
H155N	0 ± 0	0 ± 0	0 ± 0	0 ± 0	0 ± 0
H155S	0 ± 0	1 ± 0.8	0 ± 0	0 ± 0	7 ± 1.6
H155Y	0 ± 0	0 ± 0	0 ± 0	0 ± 0	0 ± 0
E41Q-G42D	7 ± 0.2	59 ± 0.8	41 ± 1.4	89 ± 0.3	36 ± 1.1

[a]: The reactions were performed as described in Methods. Data are an average of at least three independent experiments (average ± SD). Wild type UDG activity (100%) was defined as percent cleavage product generated after 60 min incubation.

**sp|P12295**	UNG_ECOLI_Uracil-DNA_glycosylase_OS_Escherichia_coli_(strain_K12)_GN_ung_PE_1_SV_2
**tr|Q8KLI8**	Q8KLI8_THETH_Uracil-DNA_glycosylase_OS_Thermus_thermophilus_GN_udga_PE_4_SV_1
**sp|A1QYK3**	UNG_BORT9_Uracil-DNA_glycosylase_OS_Borrelia_turicatae_(strain_91E135)_GN_ung_PE_3_SV_1
**sp|A1SUE5**	UNG_PSYIN_Uracil-DNA_glycosylase_OS_Psychromonas_ingrahamii_(strain_37)_GN_ung_PE_3_SV_1
**sp|B0B8I2**	UNG_CHLT2_Uracil-DNA_glycosylase_OS_Chlamydia_trachomatis_serovar_L2_(strain_434/Bu_/_ATCC_VR-902B)_GN_ung_PE_3_SV_1
**sp|B1AJI8**	UNG_UREP2_Uracil-DNA_glycosylase_OS_Ureaplasma_parvum_serovar_3_(strain_ATCC_27815_/_27_/_NCTC_11736)_GN_ung_PE_3_SV_1
**sp|P12888**	UNG_EBVB9_Uracil-DNA_glycosylase_OS_Epstein-Barr_virus_(strain_B95-8)_GN_UNG_PE_1_SV_1
**sp|Q057V4**	UNG_BUCCC_Uracil-DNA_glycosylase_OS_Buchnera_aphidicola_subsp._Cinara_cedri_(strain_Cc)_GN_ung_PE_3_SV_1
**sp|Q315T1**	UNG_DESDG_Uracil-DNA_glycosylase_OS_Desulfovibrio_desulfuricans_(strain_G20)_GN_ung_PE_3_SV_1
**tr|A0LFG4**	A0LFG4_SYNFM_Phage_SPO1_DNA_polymerase-related_protein_OS_Syntrophobacter_fumaroxidans_(strain_DSM_10017_/_MPOB)_GN_Sfum_0466_PE_4_SV_1
**tr|A0LH32**	A0LH32_SYNFM_Uracil-DNA_glycosylase_superfamily_OS_Syntrophobacter_fumaroxidans_(strain_DSM_10017_/_MPOB)_GN_Sfum_1040_PE_4_SV_1
**tr|A0LRS0**	A0LRS0_ACIC1_Phage_SPO1_DNA_polymerase-related_protein_OS_Acidothermus_cellulolyticus_(strain_ATCC_43068_/_11B)_GN_Acel_0356_PE_4_SV_1
**tr|A0RV64**	A0RV64_CENSY_Uracil-DNA_glycosylase_OS_Cenarchaeum_symbiosum_(strain_A)_GN_CENSYa_0598_PE_4_SV_1
**tr|A1BDC5**	A1BDC5_CHLPD_Phage_SPO1_DNA_polymerase-related_protein_OS_Chlorobium_phaeobacteroides_(strain_DSM_266)_GN_Cpha266_0343_PE_4_SV_1
**tr|A1RYC0**	A1RYC0_THEPD_Phage_SPO1_DNA_polymerase-related_protein_OS_Thermofilum_pendens_(strain_Hrk_5)_GN_Tpen_0798_PE_4_SV_1
**tr|A1US05**	A1US05_BARBK_Uracil-DNA_glycosylase_family_4_OS_Bartonella_bacilliformis_(strain_ATCC_35685_/_KC583)_GN_BARBAKC583_0440_PE_4_SV_1
**tr|A4GHT2**	A4GHT2_9BACT_Uracil-DNA_glycosylase_OS_uncultured_marine_bacterium_EB0_39H12_GN_ung_PE_3_SV_1
**tr|A4HXS3**	A4HXS3_LEIIN_Putative_uracil-DNA-glycosylase_OS_Leishmania_infantum_GN_LINJ_18_0480_PE_3_SV_1
**tr|A5KS51**	A5KS51_9BACT_Phage_SPO1_DNA_polymerase-related_protein_OS_candidate_division_TM7_genomosp._GTL1_GN_TM7_0110_PE_4_SV_1
**tr|A5UQ47**	A5UQ47_ROSS1_Uracil-DNA_glycosylase_superfamily_OS_Roseiflexus_sp._(strain_RS-1)_GN_RoseRS_0318_PE_4_SV_1
**tr|A6DBB2**	A6DBB2_9PROT_Uracil-DNA_glycosylase_superfamily_protein_OS_Caminibacter_mediatlanticus_TB-2_GN_CMTB2_03873_PE_4_SV_1
**tr|A6DK00**	A6DK00_9BACT_Uracil-DNA_glycosylase_OS_Lentisphaera_araneosa_HTCC2155_GN_ung_PE_3_SV_1
**tr|A6GN76**	A6GN76_9BURK_Phage_SPO1_DNA_polymerase-related_protein_OS_Limnobacter_sp._MED105_GN_LMED105_01338_PE_4_SV_1
**tr|A7ANQ0**	A7ANQ0_BABBO_Uracil-DNA_glycosylase_family_protein_OS_Babesia_bovis_GN_BBOV_III006230_PE_3_SV_1
**tr|A8A982**	A8A982_IGNH4_Phage_SPO1_DNA_polymerase-related_protein_OS_Ignicoccus_hospitalis_(strain_KIN4/I_/_DSM_18386_/_JCM_14125)_GN_Igni_0301_PE_4_SV_1
**tr|A8E1M5**	A8E1M5_MUHVK_M114_protein_OS_Murid_herpesvirus_1_(strain_K181)_GN_m114_PE_4_SV_1
**tr|A8F067**	A8F067_RICCK_N-terminus_of_bacteriophage-type_DNA_polymerase_OS_Rickettsia_canadensis_(strain_McKiel)_GN_A1E_05420_PE_4_SV_1
**tr|A8MA01**	A8MA01_CALMQ_Phage_SPO1_DNA_polymerase-related_protein_OS_Caldivirga_maquilingensis_(strain_ATCC_700844_/_DSM_13496_/_JCM_10307_/_IC-167)_GN_Cmaq_1649_PE_4_SV_1
**tr|A8SNH5**	A8SNH5_9FIRM_Uracil-DNA_glycosylase_OS_Parvimonas_micra_ATCC_33270_GN_ung_PE_3_SV_1
**tr|A8UAT5**	A8UAT5_9LACT_DNA_polymerase_bacteriophage-type_OS_Carnobacterium_sp._AT7_GN_CAT7_11735_PE_4_SV_1
**tr|A8UU74**	A8UU74_9AQUI_N-terminus_of_phage_SPO1_DNA_polymerase_OS_Hydrogenivirga_sp._128-5-R1-1_GN_HG1285_16840_PE_4_SV_1
**tr|A9AZ06**	A9AZ06_HERA2_Phage_SPO1_DNA_polymerase-related_protein_OS_Herpetosiphon_aurantiacus_(strain_ATCC_23779_/_DSM_785)_GN_Haur_4414_PE_4_SV_1
**tr|A9BHU0**	A9BHU0_PETMO_Phage_SPO1_DNA_polymerase-related_protein_OS_Petrotoga_mobilis_(strain_DSM_10674_/_SJ95)_GN_Pmob_1351_PE_4_SV_1
**tr|A9F5H8**	A9F5H8_SORC5_DNA-directed_DNA_polymerase_OS_Sorangium_cellulosum_(strain_So_ce56)_GN_sce7637_PE_4_SV_1
**tr|A9NHD4**	A9NHD4_ACHLI_Uracil-DNA_glycosylase_OS_Acholeplasma_laidlawii_(strain_PG-8A)_GN_ung_PE_3_SV_1
**tr|B0VFE1**	B0VFE1_CLOAI_Uracil-DNA_glycosylase_OS_Cloacamonas_acidaminovorans_(strain_Evry)_GN_ung_PE_4_SV_1
**tr|B1GA08**	B1GA08_9BURK_Uracil-DNA_glycosylase_OS_Burkholderia_graminis_C4D1M_GN_ung_PE_3_SV_1
**tr|B1H0K1**	B1H0K1_UNCTG_Uracil-DNA_glycosylase_OS_Uncultured_termite_group_1_bacterium_phylotype_Rs-D17_GN_TGRD_550_PE_4_SV_1
**tr|B1I4W5**	B1I4W5_DESAP_Phage_SPO1_DNA_polymerase-related_protein_OS_Desulforudis_audaxviator_(strain_MP104C)_GN_Daud_1530_PE_4_SV_1
**tr|B1VA82**	B1VA82_PHYAS_Uracil-DNA_glycosylase_OS_Phytoplasma_australiense_GN_ung_PE_3_SV_1
**tr|B1Y7I1**	B1Y7I1_LEPCP_Uracil-DNA_glycosylase_OS_Leptothrix_cholodnii_(strain_ATCC_51168_/_LMG_8142_/_SP-6)_GN_ung_PE_3_SV_1
**tr|B1ZZ66**	B1ZZ66_OPITP_Phage_SPO1_DNA_polymerase-related_protein_OS_Opitutus_terrae_(strain_DSM_11246_/_PB90-1)_GN_Oter_3864_PE_4_SV_1
**tr|B2A668**	B2A668_NATTJ_Phage_SPO1_DNA_polymerase-related_protein_OS_Natranaerobius_thermophilus_(strain_ATCC_BAA-1301_/_DSM_18059_/_JW/NM-WN-LF)_GN_Nther_0483_PE_4_SV_1
**tr|B2KCZ8**	B2KCZ8_ELUMP_Uracil-DNA_glycosylase_OS_Elusimicrobium_minutum_(strain_Pei191)_GN_Emin_0839_PE_4_SV_1
**tr|B3DV47**	B3DV47_METI4_Uracil-DNA_glycosylase_OS_Methylacidiphilum_infernorum_(isolate_V4)_GN_Minf_1145_PE_4_SV_1
**tr|B3U4N1**	B3U4N1_9BACT_Uracil-DNA_glycosylase_OS_Candidatus_Nitrospira_defluvii_GN_ung_PE_3_SV_1
**tr|B3U4U7**	B3U4U7_9BACT_Uracil-DNA_glycosylase_OS_Candidatus_Nitrospira_defluvii_GN_udgA_PE_4_SV_1
**tr|B5II11**	B5II11_9CHRO_Bacteriophage-type_DNA_polymerase_N-terminal_domain_protein_OS_Cyanobium_sp._PCC_7001_GN_CPCC7001_2580_PE_4_SV_1
**tr|B5LRM7**	B5LRM7_9ALPH_UL2_OS_Anatid_herpesvirus_1_GN_UL2_PE_3_SV_1
**tr|B5Y8I5**	B5Y8I5_COPPD_DNA_polymerase_bacteriophage-type_OS_Coprothermobacter_proteolyticus_(strain_ATCC_35245_/_DSM_5265_/_BT)_GN_COPRO5265_0734_PE_4_SV_1
**tr|B5YEZ9**	B5YEZ9_DICT6_DNA_polymerase_bacteriophage-type_OS_Dictyoglomus_thermophilum_(strain_ATCC_35947_/_DSM_3960_/_H-6-12)_GN_DICTH_1285_PE_4_SV_1
**tr|B6A3R0**	B6A3R0_RHILW_Phage_SPO1_DNA_polymerase-related_protein_OS_Rhizobium_leguminosarum_bv._trifolii_(strain_WSM2304)_GN_Rleg2_5870_PE_4_SV_1
**tr|B6AFI4**	B6AFI4_CRYMR_Uracil-DNA_glycosylase_family_protein_OS_Cryptosporidium_muris_(strain_RN66)_GN_CMU_033600_PE_3_SV_1
**tr|B6K2R2**	B6K2R2_SCHJY_Uracil-DNA_glycosylase_OS_Schizosaccharomyces_japonicus_(strain_yFS275_/_FY16936)_GN_SJAG_02528_PE_3_SV_2
**tr|B6KW10**	B6KW10_TOXGO_Uracil-DNA_glycosylase_putative_OS_Toxoplasma_gondii_GN_TGVEG_029730_PE_3_SV_1
**tr|B7FQS1**	B7FQS1_PHATC_Uracil-DNA_glycosylase_(Fragment)_OS_Phaeodactylum_tricornutum_(strain_CCAP_1055/1)_GN_PHATRDRAFT_8763_PE_3_SV_1
**tr|B7KM64**	B7KM64_CYAP7_Phage_SPO1_DNA_polymerase-related_protein_OS_Cyanothece_sp._(strain_PCC_7424)_GN_PCC7424_5828_PE_4_SV_1
**tr|B8C427**	B8C427_THAPS_Glycosylase_OS_Thalassiosira_pseudonana_GN_UNG_PE_3_SV_1
**tr|B8CX81**	B8CX81_HALOH_Uracil-DNA_glycosylase_OS_Halothermothrix_orenii_(strain_H_168_/_OCM_544_/_DSM_9562)_GN_Hore_11480_PE_4_SV_1
**tr|B8EJL3**	B8EJL3_METSB_Phage_SPO1_DNA_polymerase-related_protein_OS_Methylocella_silvestris_(strain_BL2_/_DSM_15510_/_NCIMB_13906)_GN_Msil_0443_PE_4_SV_1
**tr|B8HFW5**	B8HFW5_ARTCA_Uncharacterized_protein_OS_Arthrobacter_chlorophenolicus_(strain_A6_/_ATCC_700700_/_DSM_12829_/_JCM_12360)_GN_Achl_3197_PE_4_SV_1
**tr|B8I5K9**	B8I5K9_CLOCE_Uracil-DNA_glycosylase_superfamily_OS_Clostridium_cellulolyticum_(strain_ATCC_35319_/_DSM_5812_/_JCM_6584_/_H10)_GN_Ccel_2415_PE_4_SV_1
**tr|B9KXL9**	B9KXL9_THERP_Phage_DNA_polymerase-related_protein_OS_Thermomicrobium_roseum_(strain_ATCC_27502_/_DSM_5159_/_P-2)_GN_trd_0206_PE_4_SV_1
**tr|B9XNJ8**	B9XNJ8_9BACT_Phage_SPO1_DNA_polymerase-related_protein_OS_Pedosphaera_parvula_Ellin514_GN_Cflav_PD1728_PE_4_SV_1
**tr|B9Z7Q7**	B9Z7Q7_9NEIS_Uracil-DNA_glycosylase_OS_Pseudogulbenkiania_ferrooxidans_2002_GN_ung_PE_3_SV_1
**tr|C0BNZ8**	C0BNZ8_9BACT_Uracil-DNA_glycosylase_OS_Flavobacteria_bacterium_MS024-3C_GN_ung_PE_3_SV_1
**tr|C0X269**	C0X269_ENTFL_Uracil-DNA_glycosylase_family_4_OS_Enterococcus_faecalis_TX0104_GN_HMPREF0348_0604_PE_4_SV_1
**tr|C1A7L2**	C1A7L2_GEMAT_Uncharacterized_protein_OS_Gemmatimonas_aurantiaca_(strain_T-27_/_DSM_14586_/_JCM_11422_/_NBRC_100505)_GN_GAU_1180_PE_4_SV_1
**tr|C1D9B0**	C1D9B0_LARHH_Uracil-DNA_glycosylase_OS_Laribacter_hongkongensis_(strain_HLHK9)_GN_ung_PE_3_SV_1
**tr|C1F1L1**	C1F1L1_ACIC5_Uracil-DNA_glycosylase_family_4_OS_Acidobacterium_capsulatum_(strain_ATCC_51196_/_DSM_11244_/_JCM_7670)_GN_ACP_0612_PE_4_SV_1
**tr|C1G9A4**	C1G9A4_PARBD_Uracil-DNA_glycosylase_OS_Paracoccidioides_brasiliensis_(strain_Pb18)_GN_PADG_03840_PE_3_SV_1
**tr|C2MIJ3**	C2MIJ3_BACCE_Uracil-DNA_glycosylase_superfamily_OS_Bacillus_cereus_m1293_GN_bcere0001_13930_PE_4_SV_1
**tr|C3X2T7**	C3X2T7_OXAFO_Uracil-DNA_glycosylase_family_4_OS_Oxalobacter_formigenes_HOxBLS_GN_OFAG_00676_PE_4_SV_1
**tr|C5CIT9**	C5CIT9_KOSOT_Phage_SPO1_DNA_polymerase-related_protein_OS_Kosmotoga_olearia_(strain_TBF_19.5.1)_GN_Kole_0203_PE_4_SV_1
**tr|C5NVK4**	C5NVK4_9BACL_Uracil-DNA_glycosylase_OS_Gemella_haemolysans_ATCC_10379_GN_ung_PE_3_SV_1
**tr|C7H0A3**	C7H0A3_9FIRM_Uracil-DNA_glycosylase_OS_Eubacterium_saphenum_ATCC_49989_GN_ung_PE_3_SV_1
**tr|C7M312**	C7M312_ACIFD_Phage_SPO1_DNA_polymerase-related_protein_OS_Acidimicrobium_ferrooxidans_(strain_DSM_10331_/_JCM_15462_/_NBRC_103882_/_ICP)_GN_Afer_0438_PE_4_SV_1
**tr|C8W879**	C8W879_ATOPD_Phage_SPO1_DNA_polymerase-related_protein_OS_Atopobium_parvulum_(strain_ATCC_33793_/_DSM_20469_/_JCM_10300_/_VPI_0546)_GN_Apar_1244_PE_4_SV_1
**tr|C9M5Q7**	C9M5Q7_9BACT_Uracil-DNA_glycosylase_A_OS_Jonquetella_anthropi_E3_33_E1_GN_GCWU000246_00270_PE_4_SV_1
**tr|C9RLM9**	C9RLM9_FIBSS_Uracil-DNA_glycosylase_OS_Fibrobacter_succinogenes_(strain_ATCC_19169_/_S85)_GN_ung_PE_3_SV_1
**tr|D0MK10**	D0MK10_RHOM4_Phage_SPO1_DNA_polymerase-related_protein_OS_Rhodothermus_marinus_(strain_ATCC_43812_/_DSM_4252_/_R-10)_GN_Rmar_0014_PE_4_SV_1
**tr|D0PPD4**	D0PPD4_MHV68_Uracil-DNA_glycosylase_OS_Wood_mouse_herpesvirus_GN_ORF46_PE_3_SV_1
**tr|D0U4F0**	D0U4F0_9GAMM_Uracil-DNA_glycosylase_OS_uncultured_SUP05_cluster_bacterium_GN_ung_PE_3_SV_1
**tr|D1ART8**	D1ART8_SEBTE_Uracil-DNA_glycosylase_OS_Sebaldella_termitidis_(strain_ATCC_33386_/_NCTC_11300)_GN_ung_PE_3_SV_1
**tr|D1C3J5**	D1C3J5_SPHTD_Phage_SPO1_DNA_polymerase-related_protein_OS_Sphaerobacter_thermophilus_(strain_DSM_20745_/_S_6022)_GN_Sthe_1377_PE_4_SV_1
**tr|D1CDM9**	D1CDM9_THET1_Phage_SPO1_DNA_polymerase-related_protein_OS_Thermobaculum_terrenum_(strain_ATCC_BAA-798_/_YNP1)_GN_Tter_0113_PE_4_SV_1
**tr|D1JDZ5**	D1JDZ5_9ARCH_Putative_uncharacterized_protein_OS_uncultured_archaeon_GN_BSM_02300_PE_4_SV_1
**tr|D1PN05**	D1PN05_9FIRM_Uracil-DNA_glycosylase_OS_Subdoligranulum_variabile_DSM_15176_GN_ung_PE_3_SV_1
**tr|D1Y5H9**	D1Y5H9_9BACT_Uracil-DNA_glycosylase_A_OS_Pyramidobacter_piscolens_W5455_GN_HMPREF7215_2148_PE_4_SV_1
**tr|D2EF83**	D2EF83_9EURY_Phage_SPO1_DNA_polymerase-related_protein_OS_Candidatus_Parvarchaeum_acidiphilum_ARMAN-4_GN_BJBARM4_0397_PE_4_SV_1
**tr|D2Z573**	D2Z573_9BACT_Phage_SPO1_DNA_polymerase-related_protein_OS_Dethiosulfovibrio_peptidovorans_DSM_11002_GN_Dpep_0604_PE_4_SV_1
**tr|D3B454**	D3B454_POLPA_Uracil_glycosylase_OS_Polysphondylium_pallidum_GN_uglA_PE_3_SV_1
**tr|D3E6H2**	D3E6H2_GEOS4_Uracil-DNA_glycosylase_superfamily_OS_Geobacillus_sp._(strain_Y412MC10)_GN_GYMC10_2055_PE_4_SV_1
**tr|D3FBP7**	D3FBP7_CONWI_Phage_SPO1_DNA_polymerase-related_protein_OS_Conexibacter_woesei_(strain_DSM_14684_/_JCM_11494_/_NBRC_100937_/_ID131577)_GN_Cwoe_2893_PE_4_SV_1
**tr|D3QZ35**	D3QZ35_CLOB3_Uracil-DNA_glycosylase_OS_Clostridiales_genomosp._BVAB3_(strain_UPII9-5)_GN_ung_PE_3_SV_1
**tr|D4G8B4**	D4G8B4_RIEPU_Uracil-DNA_glycosylase_OS_Riesia_pediculicola_(strain_USDA)_GN_ung_PE_3_SV_1
**tr|D5C4I1**	D5C4I1_NITHN_Phage_SPO1_DNA_polymerase-related_protein_OS_Nitrosococcus_halophilus_(strain_Nc4)_GN_Nhal_2061_PE_4_SV_1
**tr|D5E730**	D5E730_METMS_Phage_SPO1_DNA_polymerase-related_protein_OS_Methanohalophilus_mahii_(strain_ATCC_35705_/_DSM_5219_/_SLP)_GN_Mmah_1470_PE_4_SV_1
**tr|D5EGK6**	D5EGK6_AMICL_Phage_SPO1_DNA_polymerase-related_protein_OS_Aminobacterium_colombiense_(strain_DSM_12261_/_ALA-1)_GN_Amico_1572_PE_4_SV_1
**tr|D5EHP1**	D5EHP1_CORAD_Phage_SPO1_DNA_polymerase-related_protein_OS_Coraliomargarita_akajimensis_(strain_DSM_45221_/_IAM_15411_/_JCM_23193_/_KCTC_12865)_GN_Caka_1060_PE_4_SV_1
**tr|D5MIW4**	D5MIW4_9BACT_Uncharacterized_protein_OS_Candidatus_Methylomirabilis_oxyfera_GN_DAMO_2419_PE_4_SV_1
**tr|D5RKP0**	D5RKP0_9PROT_Uracil-DNA_glycosylase_OS_Roseomonas_cervicalis_ATCC_49957_GN_ung_PE_3_SV_1
**tr|D5U040**	D5U040_THEAM_Phage_SPO1_DNA_polymerase-related_protein_OS_Thermosphaera_aggregans_(strain_DSM_11486_/_M11TL)_GN_Tagg_0210_PE_4_SV_1

**tr|D5U570**	D5U570_BRAM5_Phage_SPO1_DNA_polymerase-related_protein_OS_Brachyspira_murdochii_(strain_ATCC_51284_/_DSM_12563_/_56-150)_GN_Bmur_0231_PE_4_SV_1
**tr|D5V6V9**	D5V6V9_ARCNC_Uracil-DNA_glycosylase_OS_Arcobacter_nitrofigilis_(strain_ATCC_33309_/_DSM_7299_/_LMG_7604_/_NCTC_12251_/_CI)_GN_ung_PE_3_SV_1
**tr|D5WWR6**	D5WWR6_BACT2_Phage_SPO1_DNA_polymerase-related_protein_OS_Bacillus_tusciae_(strain_DSM_2912_/_NBRC_15312_/_T2)_GN_Btus_1029_PE_4_SV_1
**tr|D7CKV6**	D7CKV6_SYNLT_Phage_SPO1_DNA_polymerase-related_protein_OS_Syntrophothermus_lipocalidus_(strain_DSM_12680_/_TGB-C1)_GN_Slip_0557_PE_4_SV_1
**tr|D7CWJ9**	D7CWJ9_TRURR_Phage_SPO1_DNA_polymerase-related_protein_OS_Truepera_radiovictrix_(strain_DSM_17093_/_CIP_108686_/_LMG_22925_/_RQ-24)_GN_Trad_1275_PE_4_SV_1
**tr|D7UXZ6**	D7UXZ6_LISGR_Uracil-DNA_glycosylase_family_4_OS_Listeria_grayi_DSM_20601_GN_ung2_PE_4_SV_1
**tr|D8LFW4**	D8LFW4_ECTSI_Putative_uncharacterized_protein_OS_Ectocarpus_siliculosus_GN_Esi_0152_0030_PE_3_SV_1
**tr|D8UI66**	D8UI66_VOLCA_Uracil-DNA_glycosylase_(Fragment)_OS_Volvox_carteri_GN_VOLCADRAFT_69325_PE_3_SV_1
**tr|D9Q1V3**	D9Q1V3_ACIS3_Uracil-DNA_glycosylase_OS_Acidilobus_saccharovorans_(strain_DSM_16705_/_VKM_B-2471_/_345-15)_GN_ASAC_0885_PE_4_SV_1
**tr|D9QQQ4**	D9QQQ4_ACEAZ_Phage_SPO1_DNA_polymerase-related_protein_OS_Acetohalobium_arabaticum_(strain_ATCC_49924_/_DSM_5501_/_Z-7288)_GN_Acear_1332_PE_4_SV_1
**tr|D9S3H2**	D9S3H2_THEOJ_Phage_SPO1_DNA_polymerase-related_protein_OS_Thermosediminibacter_oceani_(strain_ATCC_BAA-1034_/_DSM_16646_/_JW/IW-1228P)_GN_Toce_1191_PE_4_SV_1
**tr|D9SCW5**	D9SCW5_GALCS_Phage_SPO1_DNA_polymerase-related_protein_OS_Gallionella_capsiferriformans_(strain_ES-2)_GN_Galf_0612_PE_4_SV_1
**tr|D9Y2E5**	D9Y2E5_9BURK_Uracil-DNA_glycosylase_OS_Burkholderiales_bacterium_1_1_47_GN_ung_PE_3_SV_1
**tr|E0S9I8**	E0S9I8_ENCIT_Uracyl-DNA_glycosylase_OS_Encephalitozoon_intestinalis_(strain_ATCC_50506)_GN_Eint_100470_PE_3_SV_1
**tr|E0SQC9**	E0SQC9_IGNAA_Phage_SPO1_DNA_polymerase-related_protein_OS_Ignisphaera_aggregans_(strain_DSM_17230_/_JCM_13409_/_AQ1.S1)_GN_Igag_0230_PE_4_SV_1
**tr|E0TBI7**	E0TBI7_PARBH_Putative_uncharacterized_protein_OS_Parvularcula_bermudensis_(strain_ATCC_BAA-594_/_HTCC2503_/_KCTC_12087)_GN_PB2503_01417_PE_4_SV_1
**tr|E0TIS2**	E0TIS2_ZINIC_Putative_uracil-DNA_glycosylase_family_4_OS_Zinderia_insecticola_(strain_CARI)_GN_ZICARI_078_PE_4_SV_1
**tr|E1IBI3**	E1IBI3_9CHLR_Putative_uncharacterized_protein_OS_Oscillochloris_trichoides_DG-6_GN_OSCT_0684_PE_4_SV_1
**tr|E1L0U9**	E1L0U9_9ACTN_Uracil-DNA_glycosylase_OS_Atopobium_vaginae_PB189-T1-4_GN_ung_PE_3_SV_1
**tr|E1QFX8**	E1QFX8_DESB2_Phage_SPO1_DNA_polymerase-related_protein_OS_Desulfarculus_baarsii_(strain_ATCC_33931_/_DSM_2075_/_VKM_B-1802_/_2st14)_GN_Deba_1220_PE_4_SV_1
**tr|E1QNN8**	E1QNN8_VULDI_Phage_SPO1_DNA_polymerase-related_protein_OS_Vulcanisaeta_distributa_(strain_DSM_14429_/_JCM_11212_/_NBRC_100878_/_IC-017)_GN_Vdis_1954_PE_4_SV_1
**tr|E1QWE2**	E1QWE2_OLSUV_Uracil-DNA_glycosylase_OS_Olsenella_uli_(strain_ATCC_49627_/_DSM_7084_/_CIP_109912_/_JCM_12494_/_VPI_D76D-27C)_GN_ung_PE_3_SV_1
**tr|E1R614**	E1R614_SPISS_Phage_SPO1_DNA_polymerase-related_protein_OS_Spirochaeta_smaragdinae_(strain_DSM_11293_/_JCM_15392_/_SEBR_4228)_GN_Spirs_1652_PE_4_SV_1
**tr|E2IUG8**	E2IUG8_SHV1_Uracil-DNA_glycosylase_OS_Saimiriine_herpesvirus_1_(strain_MV-5-4-PSL)_GN_UL02_PE_3_SV_1
**tr|E2SYG7**	E2SYG7_9RALS_Uracil-DNA_glycosylase_family_4_OS_Ralstonia_sp._5_7_47FAA_GN_HMPREF1004_02068_PE_4_SV_1
**tr|E3D056**	E3D056_9BACT_Phage_SPO1_DNA_polymerase-related_protein_OS_Aminomonas_paucivorans_DSM_12260_GN_Apau_1416_PE_4_SV_1
**tr|E3IP47**	E3IP47_DESVR_Uracil-DNA_glycosylase_OS_Desulfovibrio_vulgaris_(strain_RCH1)_GN_ung_PE_3_SV_1
**tr|E4KQ36**	E4KQ36_9LACT_Uracil-DNA_glycosylase_OS_Eremococcus_coleocola_ACS-139-V-Col8_GN_ung_PE_3_SV_1
**tr|E4RKG8**	E4RKG8_HALSL_Phage_SPO1_DNA_polymerase-related_protein_OS_Halanaerobium_sp._(strain_sapolanicus)_GN_Halsa_1249_PE_4_SV_1
**tr|E6N689**	E6N689_9ARCH_DNA_polymerase_bacteriophage-type_OS_Candidatus_Caldiarchaeum_subterraneum_GN_CSUB_C0833_PE_4_SV_1
**tr|E6PFU2**	E6PFU2_9ZZZZ_Phage_SPO1_DNA_polymerase-related_protein_OS_mine_drainage_metagenome_GN_CARN1_1346_PE_4_SV_1
**tr|E6W1H1**	E6W1H1_DESIS_Uracil-DNA_glycosylase_OS_Desulfurispirillum_indicum_(strain_ATCC_BAA-1389_/_S5)_GN_ung_PE_3_SV_1
**tr|E6W552**	E6W552_DESIS_Phage_SPO1_DNA_polymerase-related_protein_OS_Desulfurispirillum_indicum_(strain_ATCC_BAA-1389_/_S5)_GN_Selin_2416_PE_4_SV_1
**tr|E7A9L4**	E7A9L4_HELFC_Uracil-DNA_glycosylase_OS_Helicobacter_felis_(strain_ATCC_49179_/_NCTC_12436_/_CS1)_GN_ung_PE_3_SV_1
**tr|E7FX98**	E7FX98_ERYRH_Uracil-DNA_glycosylase_OS_Erysipelothrix_rhusiopathiae_ATCC_19414_GN_ung_PE_3_SV_1
**tr|E7H418**	E7H418_9BURK_Putative_uncharacterized_protein_OS_Sutterella_wadsworthensis_3_1_45B_GN_HMPREF9464_01456_PE_4_SV_1
**tr|E7H4Y1**	E7H4Y1_9BURK_Uracil-DNA_glycosylase_OS_Sutterella_wadsworthensis_3_1_45B_GN_ung_PE_3_SV_1
**tr|E8LLT1**	E8LLT1_9GAMM_Uracil-DNA_glycosylase_OS_Succinatimonas_hippei_YIT_12066_GN_ung_PE_3_SV_1
**tr|E8N338**	E8N338_ANATU_Uracil-DNA_glycosylase_OS_Anaerolinea_thermophila_(strain_DSM_14523_/_JCM_11388_/_NBRC_100420_/_UNI-1)_GN_ANT_31640_PE_4_SV_1
**tr|E8T398**	E8T398_THEA1_Phage_SPO1_DNA_polymerase-related_protein_OS_Thermovibrio_ammonificans_(strain_DSM_15698_/_JCM_12110_/_HB-1)_GN_Theam_1267_PE_4_SV_1
**tr|F0EKF3**	F0EKF3_ENTCA_Uracil-DNA_glycosylase_family_4_OS_Enterococcus_casseliflavus_ATCC_12755_GN_ung2_PE_4_SV_1
**tr|F0NSC6**	F0NSC6_LACHH_Uracil-DNA_glycosylase_family_protein_OS_Lactobacillus_helveticus_(strain_H10)_GN_LBHH_0793_PE_4_SV_1
**tr|F0RSJ4**	F0RSJ4_SPHGB_Phage_SPO1_DNA_polymerase-related_protein_OS_Sphaerochaeta_globosa_(strain_ATCC_BAA-1886_/_DSM_22777_/_Buddy)_GN_SpiBuddy_2289_PE_4_SV_1
**tr|F0SG07**	F0SG07_PLABD_Phage_SPO1_DNA_polymerase-related_protein_OS_Planctomyces_brasiliensis_(strain_ATCC_49424_/_DSM_5305_/_JCM_21570_/_NBRC_103401_/_IFAM_1448)_GN_Plabr_0669_PE_4_SV_1
**tr|F0Y985**	F0Y985_AURAN_Uracil-DNA_glycosylase_(Fragment)_OS_Aureococcus_anophagefferens_GN_AURANDRAFT_26105_PE_3_SV_1
**tr|F2LXL6**	F2LXL6_HIPMA_Phage_SPO1_DNA_polymerase-related_protein_OS_Hippea_maritima_(strain_ATCC_700847_/_DSM_10411_/_MH2)_GN_Hipma_0225_PE_4_SV_1
**tr|F1A0X7**	F1A0X7_DICPU_Putative_uncharacterized_protein_OS_Dictyostelium_purpureum_GN_DICPUDRAFT_158140_PE_3_SV_1
**tr|F2NFM2**	F2NFM2_DESAR_Phage_SPO1_DNA_polymerase-related_protein_OS_Desulfobacca_acetoxidans_(strain_ATCC_700848_/_DSM_11109_/_ASRB2)_GN_Desac_2317_PE_4_SV_1
**tr|F3KRD3**	F3KRD3_9BURK_Uracil-DNA_glycosylase_OS_Hylemonella_gracilis_ATCC_19624_GN_ung_PE_3_SV_1
**tr|F3QMU0**	F3QMU0_9BURK_Uracil-DNA_glycosylase_family_4_OS_Parasutterella_excrementihominis_YIT_11859_GN_HMPREF9439_02269_PE_4_SV_1
**tr|F4A8X0**	F4A8X0_CLOBO_Uracil_DNA_glycosylase_superfamily_protein_OS_Clostridium_botulinum_BKT015925_GN_CbC4_2501_PE_4_SV_1
**tr|F4C0J7**	F4C0J7_METCG_Uracil_DNA_glycosylase_OS_Methanosaeta_concilii_(strain_ATCC_5969_/_DSM_3671_/_JCM_10134_/_NBRC_103675_/_OCM_69_/_GP-6)_GN_MCON_1319_PE_4_SV_1
**tr|F4GMI7**	F4GMI7_PUSST_Uracil-DNA_glycosylase_OS_Pusillimonas_sp._(strain_T7-7)_GN_PT7_1741_PE_4_SV_1
**tr|F4GSY3**	F4GSY3_PUSST_Uracil-DNA_glycosylase_OS_Pusillimonas_sp._(strain_T7-7)_GN_ung_PE_3_SV_1
**tr|F4HNK0**	F4HNK0_PYRSN_Uncharacterized_protein_OS_Pyrococcus_sp._(strain_NA2)_GN_PNA2_0058_PE_4_SV_1
**tr|F4NZD5**	F4NZD5_BATDJ_Putative_uncharacterized_protein_OS_Batrachochytrium_dendrobatidis_(strain_JAM81_/_FGSC_10211)_GN_BATDEDRAFT_16329_PE_3_SV_1
**tr|F4QWL4**	F4QWL4_BREDI_Uracil-DNA_glycosylase_OS_Brevundimonas_diminuta_ATCC_11568_GN_BDIM_22570_PE_4_SV_1
**tr|F5SL17**	F5SL17_9BACL_Uracil-DNA_glycosylase_OS_Desmospora_sp._8437_GN_ung2_PE_4_SV_1
**tr|F5Y215**	F5Y215_RAMTT_Uncharacterized_protein_OS_Ramlibacter_tataouinensis_(strain_ATCC_BAA-407_/_DSM_14655_/_LMG_21543_/_TTB310)_GN_Rta_36675_PE_4_SV_1
**tr|F5YGI3**	F5YGI3_TREPZ_DNA_polymerase_domain_protein_OS_Treponema_primitia_(strain_ATCC_BAA-887_/_DSM_12427_/_ZAS-2)_GN_TREPR_2604_PE_4_SV_1
**tr|F7NHV1**	F7NHV1_9FIRM_Phage_SPO1_DNA_polymerase-related_protein_OS_Acetonema_longum_DSM_6540_GN_ALO_08243_PE_4_SV_1
**tr|F7PTA1**	F7PTA1_9BACT_Uracil-DNA_glycosylase_OS_Haloplasma_contractile_SSD-17B_GN_ung_PE_3_SV_1
**tr|F7S094**	F7S094_9GAMM_Uracil-DNA_glycosylase_OS_Idiomarina_sp._A28L_GN_ung_PE_3_SV_1
**tr|F7XVS9**	F7XVS9_MIDMI_Uracil-DNA_glycosylase_OS_Midichloria_mitochondrii_(strain_IricVA)_GN_midi_00471_PE_4_SV_1
**tr|F8ADN2**	F8ADN2_THEID_Phage_SPO1_DNA_polymerase-related_protein_OS_Thermodesulfatator_indicus_(strain_DSM_15286_/_JCM_11887_/_CIR29812)_GN_Thein_1046_PE_4_SV_1
**tr|F8L559**	F8L559_SIMNZ_Uracil-DNA_glycosylase_OS_Simkania_negevensis_(strain_ATCC_VR-1471_/_Z)_GN_ung_PE_3_SV_1
**tr|F9CXI5**	F9CXI5_9ARCH_Phage_SPO1_DNA_polymerase-related_protein_OS_Candidatus_Nitrosoarchaeum_koreensis_MY1_GN_MY1_1229_PE_4_SV_1
**tr|F9DTW2**	F9DTW2_9BACL_Uracil-DNA_glycosylase_OS_Sporosarcina_newyorkensis_2681_GN_ung2_PE_4_SV_1
**tr|F9LK36**	F9LK36_STAEP_Uracil-DNA_glycosylase_family_4_OS_Staphylococcus_epidermidis_VCU105_GN_SEVCU105_0118_PE_4_SV_1
**tr|F9ZLD4**	F9ZLD4_ACICS_Uracil-DNA_glycosylase_family_4_OS_Acidithiobacillus_caldus_(strain_SM-1)_GN_Atc_1154_PE_4_SV_1
**tr|G0LJW8**	G0LJW8_HALWC_Uracil-DNA_glycosylase_OS_Haloquadratum_walsbyi_(strain_DSM_16854_/_JCM_12705_/_C23)_GN_udg1_PE_4_SV_1
**tr|G0QBL8**	G0QBL8_9EURY_Uracil-DNA_glycosylase_family_4_OS_Candidatus_Nanosalinarum_sp._J07AB56_GN_J07AB56_03940_PE_4_SV_1
**tr|G0QFK7**	G0QFK7_9EURY_Uracil-DNA_glycosylase_family_4_OS_Candidatus_Nanosalina_sp._J07AB43_GN_J07AB43_06140_PE_4_SV_1
**tr|G0U4E4**	G0U4E4_TRYVY_Putative_uracil-DNA_glycosylase_OS_Trypanosoma_vivax_(strain_Y486)_GN_TVY486_1013510_PE_3_SV_1
**tr|G2RZ19**	G2RZ19_MYCPK_Uracil-DNA_glycosylase_OS_Mycoplasma_putrefaciens_(strain_ATCC_15718_/_NCTC_10155_/_C30_KS-1_/_KS-1)_GN_ung_PE_3_SV_1
**tr|G4RL69**	G4RL69_THETK_Uracil_DNA_glycosylase_OS_Thermoproteus_tenax_(strain_ATCC_35583_/_NBRC_100435_/_JCM_9277_/_Kra_1)_GN_TTX_1693_PE_4_SV_1
**tr|G4STH7**	G4STH7_META2_Putative_phage_SPO1_DNA_polymerase-related_protein_uracil-DNA_glycosylase_family_OS_Methylomicrobium_alcaliphilum_(strain_DSM_19304_/_NCIMB_14124_/_VKM_B-2133_/_20Z)_GN_MEALZ_31
**tr|G4T6H9**	G4T6H9_PIRID_Probable_uracil-DNA_glycosylase_OS_Piriformospora_indica_(strain_DSM_11827)_GN_PIIN_00737_PE_3_SV_1
**tr|G7WNS9**	G7WNS9_METH6_Phage_SPO1_DNA_polymerase-related_protein_OS_Methanosaeta_harundinacea_(strain_6Ac)_GN_Mhar_1325_PE_4_SV_1
**tr|G8BJ42**	G8BJ42_CANPC_Putative_uncharacterized_protein_OS_Candida_parapsilosis_(strain_CDC_317_/_ATCC_MYA-4646)_GN_CPAR2_404610_PE_3_SV_1
**tr|G8LWR8**	G8LWR8_CLOCD_Uracil-DNA_glycosylase_family_4_OS_Clostridium_clariflavum_(strain_DSM_19732_/_NBRC_101661_/_EBR45)_GN_Clocl_3269_PE_4_SV_1
**tr|G8TXV8**	G8TXV8_SULAD_Phage_SPO1_DNA_polymerase-related_protein_OS_Sulfobacillus_acidophilus_(strain_ATCC_700253_/_DSM_10332_/_NAL)_GN_Sulac_2702_PE_4_SV_1
**tr|G8XT11**	G8XT11_9BETA_Uracil-DNA_glycosylase_OS_Saimiriine_herpesvirus_3_GN_UL114_PE_3_SV_1
**tr|G9EIR0**	G9EIR0_9GAMM_Putative_uncharacterized_protein_OS_Legionella_drancourtii_LLAP12_GN_LDG_5066_PE_4_SV_1
**tr|G9XY09**	G9XY09_SPIME_Uracil-DNA_glycosylase_OS_Spiroplasma_melliferum_KC3_GN_ung_PE_3_SV_1
**tr|H0E6E4**	H0E6E4_9ACTN_Uracil-DNA_glycosylase_family_4_OS_Patulibacter_sp._I11_GN_PAI11_23940_PE_4_SV_1
**tr|H0KUY0**	H0KUY0_9FLAO_Uracil-DNA_glycosylase_OS_Elizabethkingia_anophelis_Ag1_GN_ung_PE_3_SV_1
**tr|H0Q5N1**	H0Q5N1_9RHOO_Uracil-DNA_glycosylase_OS_Azoarcus_sp._KH32C_GN_ung_PE_3_SV_1
**tr|H1D376**	H1D376_9FIRM_Uracil-DNA_glycosylase_family_4_OS_Dialister_succinatiphilus_YIT_11850_GN_HMPREF9453_02064_PE_4_SV_1
**tr|H1P1U4**	H1P1U4_9BACT_Uncharacterized_protein_OS_Holophaga_foetida_DSM_6591_GN_HolfoDRAFT_0546_PE_4_SV_1
**tr|H1P505**	H1P505_9BACT_Phage_SPO1_DNA_polymerase-related_protein_OS_Holophaga_foetida_DSM_6591_GN_HolfoDRAFT_0166_PE_4_SV_1
**tr|H1XWX4**	H1XWX4_9BACT_Phage_SPO1_DNA_polymerase-related_protein_OS_Caldithrix_abyssi_DSM_13497_GN_Calab_0007_PE_4_SV_1
**tr|H2C8Z4**	H2C8Z4_9CREN_Uracil-DNA_glycosylase_family_4_OS_Metallosphaera_yellowstonensis_MK1_GN_MetMK1DRAFT_00030630_PE_4_SV_1
**tr|H2J7L7**	H2J7L7_MARPK_Uracil-DNA_glycosylase_family_4_OS_Marinitoga_piezophila_(strain_DSM_14283_/_JCM_11233_/_KA3)_GN_Marpi_0946_PE_4_SV_1
**tr|H3KDP1**	H3KDP1_9BURK_Uracil-DNA_glycosylase_OS_Sutterella_parvirubra_YIT_11816_GN_ung_PE_3_SV_1
**tr|H3NN78**	H3NN78_9FIRM_Uracil-DNA_glycosylase_OS_Helcococcus_kunzii_ATCC_51366_GN_ung_PE_3_SV_1
**tr|H3NSX2**	H3NSX2_9GAMM_Uracil-DNA_glycosylase_OS_gamma_proteobacterium_HIMB55_GN_ung_PE_3_SV_1
**tr|H3RRL7**	H3RRL7_9LACO_Uracil-DNA_glycosylase_superfamily_protein_OS_Lactobacillus_mucosae_LM1_GN_LBLM1_15570_PE_4_SV_1
**tr|H3SCZ2**	H3SCZ2_9BACL_Phage_SPO1_DNA_polymerase-related_protein_OS_Paenibacillus_dendritiformis_C454_GN_PDENDC454_06935_PE_4_SV_1
**tr|H4GIS2**	H4GIS2_9LACO_Uracil-DNA_glycosylase_superfamily_protein_OS_Lactobacillus_gastricus_PS3_GN_PS3_13020_PE_4_SV_1
**tr|H5SI33**	H5SI33_9ZZZZ_Phage_SPO1_DNA_polymerase-related_protein_OS_uncultured_prokaryote_GN_HGMM_F31F10C09_PE_4_SV_1
**tr|H6RLZ5**	H6RLZ5_BLASD_Uracil-DNA_glycosylase_family_4_OS_Blastococcus_saxobsidens_(strain_DD2)_GN_BLASA_4250_PE_4_SV_1
**tr|H7F338**	H7F338_9LIST_Uracil-DNA_glycosylase_family_protein_OS_Listeriaceae_bacterium_TTU_M1-001_GN_KKC_02779_PE_4_SV_1

**tr|H8I7F5**	H8I7F5_METCZ_Uracil-DNA_glycosylase_family_4_OS_Methanocella_conradii_(strain_DSM_24694_/_JCM_17849_/_CGMCC_1.5162_/_HZ254)_GN_Mtc_2083_PE_4_SV_1
**tr|H8ZDF2**	H8ZDF2_NEMS1_Uracil-DNA_glycosylase_OS_Nematocida_sp._1_(strain_ERTm2_/_ATCC_PRA-371)_GN_NERG_01623_PE_3_SV_1
**tr|H9UI22**	H9UI22_SPIAZ_Uracil-DNA_glycosylase_family_4_OS_Spirochaeta_africana_(strain_ATCC_700263_/_DSM_8902_/_Z-7692)_GN_Spiaf_1078_PE_4_SV_1
**tr|H9ZZZ0**	H9ZZZ0_FERFK_Uracil-Dna_Glycosylase_OS_Fervidicoccus_fontis_(strain_DSM_19380_/_VKM_B-2539_/_Kam940)_GN_FFONT_0307_PE_4_SV_1
**tr|I0GI60**	I0GI60_CALEA_Uracil-DNA_glycosylase_OS_Caldisericum_exile_(strain_DSM_21853_/_NBRC_104410_/_AZM16c01)_GN_CSE_03210_PE_4_SV_1
**tr|I0I6B7**	I0I6B7_CALAS_Putative_uracil-DNA_glycosylase_OS_Caldilinea_aerophila_(strain_DSM_14535_/_JCM_11387_/_NBRC_104270_/_STL-6-O1)_GN_CLDAP_27650_PE_4_SV_1
**tr|I1A005**	I1A005_9MOLU_Uracil-DNA_glycosylase_OS_Mycoplasma_canis_PG_14_GN_ung_PE_3_SV_1
**tr|I1B0L2**	I1B0L2_9RHOB_Uracil-DNA_glycosylase_OS_Citreicella_sp._357_GN_ung_PE_3_SV_1
**tr|I2K0D9**	I2K0D9_DEKBR_Uracil-dna_glycosylase_OS_Dekkera_bruxellensis_AWRI1499_GN_AWRI1499_1416_PE_3_SV_1
**tr|I3CUN9**	I3CUN9_9BURK_Uracil-DNA_glycosylase_OS_Herbaspirillum_sp._GW103_GN_GWL_15730_PE_4_SV_1
**tr|I3VQA4**	I3VQA4_9BETA_B114_OS_Bat_betaherpesvirus_B7D8_PE_4_SV_1
**tr|I4CBR6**	I4CBR6_DESTA_Uracil-DNA_glycosylase_family_4_OS_Desulfomonile_tiedjei_(strain_ATCC_49306_/_DSM_6799_/_DCB-1)_GN_Desti_4374_PE_4_SV_1
**tr|I7LIA9**	I7LIA9_9CLOT_Uracil-DNA_glycosylase_superfamily_OS_Caloramator_australicus_RC3_GN_CAAU_0718_PE_4_SV_1
**tr|I7LKX1**	I7LKX1_METBM_DNA_polymerase_bacteriophage-type_OS_Methanoculleus_bourgensis_(strain_ATCC_43281_/_DSM_3045_/_OCM_15_/_MS2)_GN_31_PE_4_SV_1
**tr|J0UVA7**	J0UVA7_ALCFA_Uracil-DNA_glycosylase_OS_Alcaligenes_faecalis_subsp._faecalis_NCIB_8687_GN_QWA_05052_PE_4_SV_1
**tr|J5SPB4**	J5SPB4_TRIAS_Uracil_DNA_N-glycosylase_OS_Trichosporon_asahii_var._asahii_(strain_ATCC_90039_/_CBS_2479_/_JCM_2466_/_KCTC_7840_/_NCYC_2677_/_UAMH_7654)_GN_A1Q1_04497_PE_3_SV_1
**tr|J7R1V5**	J7R1V5_KAZNA_Uncharacterized_protein_OS_Kazachstania_naganishii_(strain_ATCC_MYA-139_/_BCRC_22969_/_CBS_8797_/_CCRC_22969_/_KCTC_17520_/_NBRC_10181_/_NCYC_3082)_GN_KNAG0B03530_PE_3_SV_1
**tr|J9E0C3**	J9E0C3_9PROT_DNA_polymerase_OS_alpha_proteobacterium_IMCC14465_GN_IMCC14465_12050_PE_4_SV_1
**tr|J9Z1B8**	J9Z1B8_9PROT_Uracil-DNA_glycosylase_OS_alpha_proteobacterium_HIMB59_GN_ung_PE_3_SV_1
**tr|K0IF13**	K0IF13_NITGG_Putative_uracil_DNA_glycosylase_superfamily_protein_OS_Nitrososphaera_gargensis_(strain_Ga9.2)_GN_Ngar_c14290_PE_4_SV_1
**tr|K0KF39**	K0KF39_WICCF_Uncharacterized_protein_OS_Wickerhamomyces_ciferrii_(strain_F-60-10_/_ATCC_14091_/_CBS_111_/_JCM_3599_/_NBRC_0793_/_NRRL_Y-1031)_GN_BN7_361_PE_3_SV_1
**tr|K1JXC9**	K1JXC9_9BURK_Uracil-DNA_glycosylase_family_4_OS_Sutterella_wadsworthensis_2_1_59BFAA_GN_HMPREF9465_00080_PE_4_SV_1
**tr|K1JYF3**	K1JYF3_9BURK_Uracil-DNA_glycosylase_OS_Sutterella_wadsworthensis_2_1_59BFAA_GN_ung_PE_3_SV_1
**tr|K1LNP2**	K1LNP2_9LACT_Uracil-DNA_glycosylase_OS_Facklamia_hominis_CCUG_36813_GN_ung_PE_3_SV_1
**tr|K1ZRE1**	K1ZRE1_9BACT_Uncharacterized_protein_OS_uncultured_bacterium_GN_ACD_57C00018G0002_PE_4_SV_1
**tr|K2A3B6**	K2A3B6_9BACT_Phage_SPO1_DNA_polymerase-related_protein_OS_uncultured_bacterium_GN_ACD_52C00151G0004_PE_4_SV_1
**tr|K2B4F4**	K2B4F4_9BACT_Uncharacterized_protein_OS_uncultured_bacterium_GN_ACD_51C00253G0004_PE_4_SV_1
**tr|K2C359**	K2C359_9BACT_Uncharacterized_protein_OS_uncultured_bacterium_GN_ACD_47C00410G0002_PE_4_SV_1
**tr|K2CLQ5**	K2CLQ5_9BACT_Putative_repair_protein_OS_uncultured_bacterium_GN_ACD_37C00126G0005_PE_4_SV_1
**tr|K2DEQ6**	K2DEQ6_9BACT_Uncharacterized_protein_OS_uncultured_bacterium_GN_ACD_24C00294G0002_PE_4_SV_1
**tr|K2E7J1**	K2E7J1_9BACT_Uracil-DNA_glycosylase_OS_uncultured_bacterium_GN_ung_PE_3_SV_1
**tr|K2E9B4**	K2E9B4_9BACT_Phage_SPO1_DNA_polymerase-related_protein_OS_uncultured_bacterium_GN_ACD_20C00358G0006_PE_4_SV_1
**tr|K2ETW7**	K2ETW7_9BACT_Phage_SPO1_DNA_polymerase_protein_OS_uncultured_bacterium_GN_ACD_21C00032G0008_PE_4_SV_1
**tr|K2F640**	K2F640_9BACT_Phage_SPO1_DNA_polymerase-related_protein_OS_uncultured_bacterium_GN_ACD_13C00015G0004_PE_4_SV_1
**tr|K4KIT8**	K4KIT8_SIMAS_Uracil-DNA_glycosylase_OS_Simiduia_agarivorans_(strain_DSM_21679_/_JCM_13881_/_BCRC_17597_/_SA1)_GN_ung_PE_3_SV_1
**tr|K6GHM9**	K6GHM9_9GAMM_Uracil-DNA_glycosylase_OS_SAR86_cluster_bacterium_SAR86E_GN_ung_PE_3_SV_1
**tr|K7YPM8**	K7YPM8_9PROT_Uracil-DNA_glycosylase_4_family_protein_OS_Candidatus_Endolissoclinum_patella_L2_GN_A1OE_327_PE_4_SV_1
**tr|K8ZAC1**	K8ZAC1_9ENTE_Uracil-DNA_glycosylase_OS_Catellicoccus_marimammalium_M35/04/3_GN_ung_PE_3_SV_1
**tr|K9VTI9**	K9VTI9_9CYAN_Phage_SPO1_DNA_polymerase-related_protein_OS_Crinalium_epipsammum_PCC_9333_GN_Cri9333_0422_PE_4_SV_1
**tr|L0A9A2**	L0A9A2_CALLD_Uracil-DNA_glycosylase_family_4_OS_Caldisphaera_lagunensis_(strain_DSM_15908_/_JCM_11604_/_IC-154)_GN_Calag_0734_PE_4_SV_1
**tr|L0B4R8**	L0B4R8_9PROT_Uracil-DNA_glycosylase_OS_Candidatus_Kinetoplastibacterium_crithidii_(ex_Angomonas_deanei_ATCC_30255)_GN_ung_PE_3_SV_1
**tr|L0B866**	L0B866_9PROT_Uracil-DNA_glycosylase_OS_Candidatus_Kinetoplastibacterium_blastocrithidii_(ex_Strigomonas_culicis)_GN_ung_PE_3_SV_1
**tr|L0B8A6**	L0B8A6_9PROT_DNA_polymerase_bacteriophage-type_OS_Candidatus_Kinetoplastibacterium_blastocrithidii_(ex_Strigomonas_culicis)_GN_CKBE_00599_PE_4_SV_1
**tr|L0DFI2**	L0DFI2_SINAD_Uracil-DNA_glycosylase_family_4_OS_Singulisphaera_acidiphila_(strain_ATCC_BAA-1392_/_DSM_18658_/_VKM_B-2454_/_MOB10)_GN_Sinac_3356_PE_4_SV_1
**tr|L0L178**	L0L178_METHD_Uracil-DNA_glycosylase_family_4_OS_Methanomethylovorans_hollandica_(strain_DSM_15978_/_NBRC_107637_/_DMS1)_GN_Metho_1844_PE_4_SV_1
**tr|L2GQC8**	L2GQC8_VITCO_Uracil-DNA_glycosylase_OS_Vittaforma_corneae_(strain_ATCC_50505)_GN_VICG_00015_PE_3_SV_1
**tr|L2GX11**	L2GX11_VAVCU_Uracil-DNA_glycosylase_OS_Vavraia_culicis_(isolate_floridensis)_GN_VCUG_00417_PE_3_SV_1
**tr|L5N8A0**	L5N8A0_9BACI_Uracil-DNA_glycosylase_OS_Halobacillus_sp._BAB-2008_GN_D479_09806_PE_4_SV_1
**tr|L8HA23**	L8HA23_ACACA_UracilDNA_glycosylase_OS_Acanthamoeba_castellanii_str._Neff_GN_ACA1_227080_PE_3_SV_1
**tr|L9X1W2**	L9X1W2_9EURY_Phage_SPO1_DNA_polymerase-related_protein_OS_Natronolimnobius_innermongolicus_JCM_12255_GN_C493_10877_PE_4_SV_1
**tr|M0BKB2**	M0BKB2_9EURY_Uracil-DNA_glycosylase_OS_Halovivax_asiaticus_JCM_14624_GN_C479_08873_PE_4_SV_1
**tr|M0CBA6**	M0CBA6_9EURY_Phage_SPO1_DNA_polymerase-like_protein_OS_Halosimplex_carlsbadense_2-9-1_GN_C475_21534_PE_4_SV_1
**tr|M0MGS7**	M0MGS7_HALMO_Uracil-DNA_glycosylase_superfamily_protein_OS_Halococcus_morrhuae_DSM_1307_GN_C448_09185_PE_4_SV_1
**tr|M1E4Q4**	M1E4Q4_9FIRM_Phage_SPO1_DNA_polymerase-related_protein_OS_Thermodesulfobium_narugense_DSM_14796_GN_Thena_0773_PE_4_SV_1
**tr|M1LNF1**	M1LNF1_9PROT_Uracil-DNA_glycosylase_OS_Candidatus_Kinetoplastibacterium_desouzaii_TCC079E_GN_ung_PE_3_SV_1
**tr|M1QKH3**	M1QKH3_9AQUI_Phage_SPO1_DNA_polymerase-related_protein_OS_Hydrogenobaculum_sp._HO_GN_HydHO_1460_PE_4_SV_1
**tr|M1VCA8**	M1VCA8_CYAME_Uracil-DNA-glycosylase_OS_Cyanidioschyzon_merolae_strain_10D_GN_CYME_CMI300C_PE_3_SV_1
**tr|M1Z9H8**	M1Z9H8_9CLOT_Uracil-DNA_glycosylase_superfamily_OS_Clostridium_ultunense_Esp_GN_CULT_2040002_PE_4_SV_1
**tr|M2XVU5**	M2XVU5_GALSU_Uracil-DNA_glycosylase_OS_Galdieria_sulphuraria_GN_Gasu_48480_PE_3_SV_1
**tr|M3EFG6**	M3EFG6_9BACL_Uracil-DNA_glycosylase_family_protein_clostridial_type_OS_Planococcus_halocryophilus_Or1_GN_B481_2976_PE_4_SV_1
**tr|M4YU22**	M4YU22_9EURY_Uracil-DNA_glycosylase_family_4_OS_Thermoplasmatales_archaeon_BRNA1_GN_TALC_00227_PE_4_SV_1
**tr|M5ADY8**	M5ADY8_9FLAO_Uracil-DNA_glycosylase_OS_Blattabacterium_sp._(Panesthia_angustipennis_spadica)_str._BPAA_GN_ung_PE_3_SV_1
**tr|M5IVB6**	M5IVB6_9BURK_Uracil-DNA_glycosylase_OS_Alcaligenes_sp._HPC1271_GN_ung_PE_3_SV_1
**tr|M6D8H9**	M6D8H9_9LEPT_Uracil-DNA_glycosylase_family_4_OS_Leptospira_sp._B5-022_GN_LEP1GSC192_2346_PE_4_SV_1
**tr|M7WR77**	M7WR77_RHOT1_Uracil-DNA_glycosylase_OS_Rhodosporidium_toruloides_(strain_NP11)_GN_RHTO_07404_PE_3_SV_1
**tr|N0BBN4**	N0BBN4_9EURY_Uracil-DNA_glycosylase_family_4_OS_Archaeoglobus_sulfaticallidus_PM70-1_GN_Asulf_00995_PE_4_SV_1
**tr|N1V4N6**	N1V4N6_9MICC_Uncharacterized_protein_OS_Arthrobacter_crystallopoietes_BAB-32_GN_D477_006436_PE_4_SV_1
**tr|N1Z687**	N1Z687_9CLOT_Uracil-DNA_glycosylase_family_4_OS_Clostridium_sp._ASF356_GN_C820_02496_PE_4_SV_1
**tr|N2BLL9**	N2BLL9_9HELI_Uracil-DNA_glycosylase_OS_Helicobacter_bilis_WiWa_GN_ung_PE_3_SV_1
**tr|N9SHT1**	N9SHT1_MYCFL_Uracil-DNA_glycosylase_OS_Mycoplasma_flocculare_ATCC_27716_GN_ung_PE_3_SV_1
**tr|N9V498**	N9V498_9MOLU_Uracil-DNA_glycosylase_OS_Mycoplasma_bovigenitalium_51080_GN_ung_PE_3_SV_1
**tr|Q025I0**	Q025I0_SOLUE_Phage_SPO1_DNA_polymerase-related_protein_OS_Solibacter_usitatus_(strain_Ellin6076)_GN_Acid_2350_PE_4_SV_1
**tr|Q0C0Y4**	Q0C0Y4_HYPNA_Uracil-DNA_glycosylase_family_4_OS_Hyphomonas_neptunium_(strain_ATCC_15444)_GN_HNE_1908_PE_4_SV_1
**tr|Q0EZR7**	Q0EZR7_9PROT_Phage_SPO1_DNA_polymerase_domain_protein_OS_Mariprofundus_ferrooxydans_PV-1_GN_SPV1_13674_PE_4_SV_1
**tr|Q12TN4**	Q12TN4_METBU_Uracil_DNA_glycosylase_OS_Methanococcoides_burtonii_(strain_DSM_6242)_GN_Mbur_2338_PE_4_SV_1
**tr|Q1AV32**	Q1AV32_RUBXD_Uracil-DNA_glycosylase_superfamily_OS_Rubrobacter_xylanophilus_(strain_DSM_9941_/_NBRC_16129)_GN_Rxyl_1787_PE_4_SV_1
**tr|Q1AYQ1**	Q1AYQ1_RUBXD_Phage_SPO1_DNA_polymerase-related_protein_OS_Rubrobacter_xylanophilus_(strain_DSM_9941_/_NBRC_16129)_GN_Rxyl_0503_PE_4_SV_1
**tr|Q1GY28**	Q1GY28_METFK_Uracil-DNA_glycosylase_OS_Methylobacillus_flagellatus_(strain_KT_/_ATCC_51484_/_DSM_6875)_GN_ung_PE_3_SV_1
**tr|Q1N1I3**	Q1N1I3_9GAMM_Uracil-DNA_glycosylase_OS_Bermanella_marisrubri_GN_ung_PE_3_SV_1
**tr|Q23W06**	Q23W06_TETTS_Uracil-DNA_glycosylase_family_protein_OS_Tetrahymena_thermophila_(strain_SB210)_GN_TTHERM_00794250_PE_3_SV_2
**tr|Q3SA81**	Q3SA81_9EURY_Uracil_DNA_glycosylase_OS_uncultured_euryarchaeote_Alv-FOS4_PE_4_SV_1
**tr|Q53CZ3**	Q53CZ3_9GAMA_JM77_OS_Macaca_fuscata_rhadinovirus_GN_JM77_PE_3_SV_1
**tr|Q5DC15**	Q5DC15_SCHJA_SJCHGC01559_protein_OS_Schistosoma_japonicum_PE_2_SV_1
**tr|Q5WES9**	Q5WES9_BACSK_Uracil-DNA_glycosylase_OS_Bacillus_clausii_(strain_KSM-K16)_GN_ABC2596_PE_4_SV_1
**tr|Q6CAC0**	Q6CAC0_YARLI_YALI0D04158p_OS_Yarrowia_lipolytica_(strain_CLIB_122_/_E_150)_GN_YALI0D04158g_PE_3_SV_1
**tr|Q6F1M3**	Q6F1M3_MESFL_Uracil-DNA_glycosylase_OS_Mesoplasma_florum_(strain_ATCC_33453_/_NBRC_100688_/_NCTC_11704_/_L1)_GN_ung_PE_3_SV_1
**tr|Q6KIL8**	Q6KIL8_MYCMO_Uracil-DNA_glycosylase_OS_Mycoplasma_mobile_(strain_ATCC_43663_/_163K_/_NCTC_11711)_GN_ung_PE_3_SV_1
**tr|Q7VBS6**	Q7VBS6_PROMA_Uracil-DNA_glycosylase_OS_Prochlorococcus_marinus_(strain_SARG_/_CCMP1375_/_SS120)_GN_Pro_1016_PE_4_SV_1
**tr|Q8TK31**	Q8TK31_METAC_Uracil-DNA_glycosylase_OS_Methanosarcina_acetivorans_(strain_ATCC_35395_/_DSM_2834_/_JCM_12185_/_C2A)_GN_udg_PE_4_SV_1
**tr|Q9RTK9**	Q9RTK9_DEIRA_DNA_polymerase-related_protein_OS_Deinococcus_radiodurans_(strain_ATCC_13939_/_DSM_20539_/_JCM_16871_/_LMG_4051_/_NBRC_15346_/_NCIMB_9279_/_R1_/_VKM_B-1422)_GN_DR_1751_PE_4_SV_1
**tr|Q9WR44**	Q9WR44_9ALPH_Uracil_DNA_glycosylase_OS_Cercopithecine_herpesvirus_9_GN_UDG_PE_3_SV_1
**tr|R1ATM3**	R1ATM3_9CLOT_Uracil-DNA_glycosylase_superfamily_OS_Clostridiaceae_bacterium_L21-TH-D2_GN_L21TH_1987_PE_4_SV_1
**tr|R1E4B2**	R1E4B2_9ARCH_Uracil-DNA_glycosylase_OS_nanoarchaeote_Nst1_GN_Nst1_654_PE_4_SV_1
**tr|R1FRL1**	R1FRL1_EMIHU_DNA-deoxyinosine_glycosylase_OS_Emiliania_huxleyi_CCMP1516_GN_EMIHUDRAFT_226938_PE_3_SV_1
**tr|R1GL12**	R1GL12_9RICK_Bacteriophage-type_DNA_polymerase_OS_Holospora_undulata_HU1_GN_K737_01154_PE_4_SV_1
**tr|R2SFV7**	R2SFV7_9ENTE_Uncharacterized_protein_OS_Enterococcus_asini_ATCC_700915_GN_I579_01584_PE_4_SV_1
**tr|R2SXC9**	R2SXC9_9ENTE_Uncharacterized_protein_OS_Enterococcus_haemoperoxidus_ATCC_BAA-382_GN_I583_00442_PE_4_SV_1
**tr|R4KRR4**	R4KRR4_9FIRM_Uracil-DNA_glycosylase_family_4_OS_Desulfotomaculum_gibsoniae_DSM_7213_GN_Desgi_3990_PE_4_SV_1
**tr|R4VE87**	R4VE87_9GAMM_Uracil-DNA_glycosylase_family_4_OS_Spiribacter_salinus_M19-40_GN_SPISAL_02605_PE_4_SV_1
**tr|R5ARL3**	R5ARL3_9CLOT_Uracil-DNA_glycosylase_superfamily_OS_Clostridium_sp._CAG:1024_GN_BN454_01149_PE_4_SV_1
**tr|R5B4U8**	R5B4U8_9CLOT_Uracil-DNA_glycosylase_superfamily_OS_Clostridium_sp._CAG:226_GN_BN545_01736_PE_4_SV_1

tr|R5DX78 R5DX78_9CLOT_Phage_SPO1_DNA_polymerase-related_protein_OS_Clostridium_sp._CAG:715_GN_BN763_01444_PE_4_SV_1
tr|R5MPG0 R5MPG0_9FIRM_Uracil-DNA_glycosylase_OS_Firmicutes_bacterium_CAG:884_GN_ung_PE_3_SV_1
tr|R5PZ23 R5PZ23_9PROT_Uracil-DNA_glycosylase_family_4_OS_Acetobacter_sp._CAG:977_GN_BN820_00559_PE_4_SV_1
tr|R5QX00 R5QX00_9PROT_Phage_SPO1_DNA_polymerase-related_protein_OS_Proteobacteria_bacterium_CAG:495_GN_BN682_00840_PE_4_SV_1
tr|R5Z1D7 R5Z1D7_9FIRM_Uracil-DNA_glycosylase_OS_Firmicutes_bacterium_CAG:536_GN_ung_PE_3_SV_1
tr|R6HGV3 R6HGV3_9ACTN_Uracil-DNA_glycosylase_OS_Eggerthella_sp._CAG:209_GN_ung_PE_3_SV_1
tr|R6HL97 R6HL97_9FIRM_Uracil-DNA_glycosylase_OS_Firmicutes_bacterium_CAG:552_GN_ung_PE_3_SV_1
tr|R6PFR8 R6PFR8_9CLOT_DNA_polymerase_bacteriophage-type_putative_OS_Clostridium_sp._CAG:306_GN_BN597_01624_PE_4_SV_1
tr|R6PI86 R6PI86_9CLOT_Phage_SPO1_DNA_polymerase-related_protein_OS_Clostridium_sp._CAG:306_GN_BN597_01992_PE_4_SV_1
tr|R6R865 R6R865_9FIRM_Uracil-DNA_glycosylase_OS_Firmicutes_bacterium_CAG:449_GN_ung_PE_3_SV_1
tr|R7BZ83 R7BZ83_9BURK_Uracil-DNA_glycosylase_OS_Sutterella_sp._CAG:397_GN_ung_PE_3_SV_1
tr|R7DQ64 R7DQ64_9FIRM_Uracil-DNA_glycosylase_OS_Coprobacillus_sp._CAG:826_GN_ung_PE_3_SV_1
tr|R7DXQ1 R7DXQ1_9BACT_Phage_SPO1_DNA_polymerase-related_protein_OS_Akkermansia_sp._CAG:344_GN_BN616_02310_PE_4_SV_1
tr|R7I8P1 R7I8P1_9FIRM_Uracil-DNA_glycosylase_family_4_OS_Faecalibacterium_sp._CAG:74_GN_BN770_01068_PE_4_SV_1
tr|R7IAS2 R7IAS2_9BURK_Uncharacterized_protein_OS_Sutterella_sp._CAG:351_GN_BN620_01109_PE_4_SV_1
tr|R7IHQ8 R7IHQ8_9FIRM_Uracil-DNA_glycosylase_superfamily_OS_Faecalibacterium_sp._CAG:74_GN_BN770_02294_PE_4_SV_1
tr|R9ADN8 R9ADN8_WALI9_Uracil-DNA_glycosylase_OS_Wallemia_ichthyophaga_(strain_EXF-994_/_CBS_113033)_GN_J056_000820_PE_4_SV_1
tr|R9LKN0 R9LKN0_9FIRM_Uracil-DNA_glycosylase_OS_Firmicutes_bacterium_M10-2_GN_ung_PE_3_SV_1
tr|S0EV15 S0EV15_9BACT_Uracil-DNA_glycosylase_family_4_OS_Chthonomonas_calidirosea_T49_GN_CCALI_01414_PE_4_SV_1
tr|S0FG57 S0FG57_9CLOT_Uracil-DNA_glycosylase_OS_Clostridium_termitidis_CT1112_GN_CTER_4473_PE_4_SV_1
tr|S2J4F7 S2J4F7_MUCC1_Uracil-DNA_glycosylase_OS_Mucor_circinelloides_f._circinelloides_(strain_1006PhL)_GN_HMPREF1544_10285_PE_3_SV_1
tr|S3JT40 S3JT40_TREMD_Uracil-DNA_glycosylase_family_4_OS_Treponema_medium_ATCC_700293_GN_HMPREF9195_01519_PE_4_SV_1
tr|S4NBY8 S4NBY8_9LACO_Uracil-DNA_glycosylase_OS_Lactobacillus_otakiensis_JCM_15040_GN_LOT_0797_PE_4_SV_1
tr|S5MFT9 S5MFT9_9MOLU_Uracil-DNA_glycosylase_OS_Spiroplasma_taiwanense_CT-1_GN_ung_PE_4_SV_1
tr|S5RQ34 S5RQ34_9PROT_Uracil-DNA_glycosylase_OS_Candidatus_Profftella_armatura_GN_SSDC_01595_PE_4_SV_1
tr|S6G4W1 S6G4W1_ANAPH_Uracil-DNA_glycosylase_OS_Anaplasma_phagocytophilum_str._HGE1_GN_HGE1_06007_PE_4_SV_1
tr|S9VE50 S9VE50_9TRYP_Uracil-DNA_glycosylase_OS_Angomonas_deanei_GN_AGDE_04999_PE_4_SV_1
tr|T0AYQ1 T0AYQ1_9FUNG_Uracil-DNA_glycosylase_domain-containing_protein_OS_Rozella_allomycis_CSF55_GN_O9G_001747_PE_4_SV_1
tr|T0DRC9 T0DRC9_HELPX_Uracil-DNA_glycosylase_OS_Helicobacter_pylori_UM038_GN_N199_01575_PE_4_SV_1
tr|T0LSQ5 T0LSQ5_9EURY_Uracil-DNA_glycosylase_family_4_OS_Thermoplasmatales_archaeon_E-plasma_GN_AMDU2_EPLC00005G0515_PE_4_SV_1
tr|T0MGQ8 T0MGQ8_9MICR_Uracil-dna_glycosylase_OS_Nosema_apis_BRL_01_GN_NAPIS_ORF02231_PE_4_SV_1

```
                       1                   10        20        30
                       .                   .         .         .
sp|P12295  ...........MANELT...........WHDVLA....EEKQ..QPYF......LN.TLQTVA.SE....R.QS..GVT.
tr|Q8KLI8  ..........................MTLELLQA....QAQN............................C.TA......
sp|A1QYK3  ....................MEVKIEESWKEMLK....GEFC..KSYF......KR.LVNFIKNEY....KTKK......
sp|A1SUE5  ..........................MSWKKFIT....QQES..MEYY......NA.LQAFLKSQK....ELGK......
sp|B0B8I2  ...............MHEAFTIEQLPPSWQEQLK....DEWS..QPYW......SQ.LLAFLKSEY......AQ......
sp|B1AJI8  ..........................MKWKEFII....NETK..QSYL......KN.IIKKINNIE....N.HQ......
sp|P12888  ...............IGVKGENLLLPDLWLDFLQ......LS.PIFQR......KL.AAVI.A.......CVRR......
sp|Q057V4  .................MKNIYK..ISWLDFFL....IEKK..KEYF......LY.LLNKIK.NI....R.RN..T...
sp|Q315T1  ..............MSRSDVFADAPPADWAQAVP....LLREGAHLPL.........LRSVARLRR......SK......
tr|A0LFG4  ........WSDPAVGLPEPEKAIEQEPDPAGGLTVIAGELEG............................C.RR......
tr|A0LH32  ....................MARRDKERSLHALY....DAIR..RDAF......................YRKE......
tr|A0LRS0  ................MSPMSRTAADDAWDQLAA....QIRR............................C.RR......
tr|A0RV64  ............QLNRARPVCTFIGARIIHRMVD....GPLGGIAARV......SV..............C.TR......
tr|A1BDC5  .......PFLFSDHDDTLPSVQSEAGAPFRDLIG.LSLAVKS............................C.TK......
tr|A1RYC0  .........MSGKKPAGSNASCGDVLRALEEIAG....EIAK............................C.EK......
tr|A1US05  ........HPDTTQHALSAIEMAKNAKTLDELKS....ALLA............................F.DG......
tr|A4GHT2  ................MSSKIDPNIDISWLNQLS....SELK..KSYL......DE.LWNFLMKEK....LKDR......
tr|A4HXS3  ........QLQPAATSSRWLAGLITNLEWRDFLAPITADSWR..SGAF......AR.IERFLDGEK....EKGR......
tr|A5KS51  ...........MKQAPAAKDHSLSDKRALLDQIR....TDIL............................AANV......
tr|A5UQ47  ............................MMISLV....QQQH............................C.VR......
tr|A6DBB2  ..........................MKWKDKLK....DFYKKRKEYI..........................KK......
tr|A6DK00  ...................MLVDKLSSDWQKALG......LD..KKYF......AE.LAKFLDSER......EK......
tr|A6GN76  ......................LPASLSLESLQE....MVAV............................C.KK......
tr|A7ANQ0  ........QTATGDSEVVSLVSKLLGEEWSDKLN....NEIK..KPYF......GN.LWAKVNKDR......KN......
tr|A8A982  ..........................MCLREVAE....KVKG............................C.CK......
tr|A8E1M5  ........ADVSADVIHQQAEALGIHEAWMSFLK....LSATQASQLV......R..ITDRVDQERRM..C.TI......
tr|A8F067  .........DKMHTNISLARSLADKVNNIEELRE....SLLN............................F.NG......
tr|A8MA01  ................MNRSGPQGEDDELAEINS....EVSN............................C.RK......
tr|A8SNH5  .....................MMSNYGAWNEYLK....DEFK..KDYF......VK.MKNFLNEEY....K.NK......
tr|A8UAT5  ....................MDTVLTTDLVALAK......................................KR......
tr|A8UU74  .............FREIYLKERLHKRKEDKSDKA....ELLRRHFDSF......KD..............C.AK......
tr|A9AZ06  .....................MTHAAATLAAIAE....EVAQ............................C.TA......
tr|A9BHU0  .................MFLYGNEEKQKNIEMIA....KRIE.......................V....C.EK......
tr|A9F5H8  ......RADTAARASATLPDTKEERAARLSLLAE....EVRT............................C.QK......
tr|A9NHD4  ...................MEIQI..PMWTNWMN....EELI..KPYF......KN.LMSFLD.ER....S.LT..E...
tr|B0VFE1  .........QYLEFLKNSGIKELYYPPSDKQKKL....TELQ...KQY......ST..............C.TK......
tr|B1GA08  ........QQHAHGTLTLEAQFAALPAAWRAHLK....PFIE..SDAY......AP.LCRFVDGER....AAGK......
tr|B1H0K1  ......KAVFPKTGRQAFYAEISSSGEELGELRE....KVSF............................C.KK......
tr|B1I4W5  .............MNNLEQLLDRLCNYLPKPAVD....AALEQLGGLS.........GRPSGARTP....CPRR......
tr|B1VA82  ...........................MWKKIID....LQRK..KDYF......QN.IAQFLKAEK....MKNK......
tr|B1Y7I1  ........VPDNRLQAPLADAFRQVAPRWRGLCD....RFLASEAGRS.........LCVEVDRRV....AAGT......
tr|B1ZZ66  ................PVARAATIPAPKPFTLPEGDKAARFAALREHV.........LADAV........C.........
tr|B2A668  ...................MQSYTHLTDLESLKK....EALI............................C.KN......
tr|B2KCZ8  ............KEDSSMPAPQTPKTKKINSNKE....AELNALAEQI......LT..............C.KK......
tr|B3DV47  ....GKHFLSKEDNSSQLVLAKGKVTKELSLLKE....KVEA............................C.RN......
tr|B3U4N1  ................MLPPFPT....GWKQLFT....KQVN..SQRY......RA.LELFLE.QE....A.AA..GQ..
tr|B3U4U7  .....................MSSSPTTLQELAE....SLHN............................C.QR......
tr|B5II11  ....TPSAGPSAGPASGSGLPAVEPDLDLAGVAA....ACAA............................C.RR......
tr|B5LRM7  ......WFNSPTEWEKLSGDYCIG..DAWREVLY....QELQ..SEVG......SR.VLLEYE.RR....C.DI...E..
tr|B5Y8I5  .......................MNQELWKELEQ....FALN............................C.KA......
tr|B5YEZ9  ...................MERLTKIDELEILRR....EAEN............................C.KN......
tr|B6A3R0  .................ALALDVAEAESIAELRH....QAEG............................C.TR......
tr|B6AFI4  ........SDCSDNFPYESLLRSYIGDEWFEYLK....SELA..QPYF......TR.CFETVRRKR....Q.IS......
tr|B6K2R2  ........NLNAEQKKLLQLEITTMDDSWFMALK....DEFL..KPYF......LQ.LKKFLMTEW....QSQR......
tr|B6KW10  ........GEMKATSEAAALWRQTLGDCWFDALK....EELR..LPYF......RD.CMQFIRQER......QK......
tr|B7FQS1  ............................WRKELA....RHLD..SASF......AS.LASFVAKER....L.VN......
tr|B7KM64  ..........MKQRSLLDILPSSNSTSPLKTLKK....TCAR............................C.TQ......
tr|B8C427  ........EPPTPHSFEEDLAHQLTDLGWQSVLS....EHMK..SESF......KS.LMSSIQSDI....DAGA......
tr|B8CX81  .......MIFDRDGKIQSSSLITYPDNDYSLLKE....KALK............................C.DR......
tr|B8EJL3  .........AGADSLERDAREAAAAAKTLEKLRA....ELLA............................F.EG......
tr|B8HFW5  ......MRPADNVDAFIGALAAVEPGADSTNFFD....HTVP..............................EN......
tr|B8I5K9  ...................MDKDTMQNRLEQLCC....EYVQ............................T.........
tr|B9KXL9  ............MTRTGRRNEREEQTVSEERSAR....ELLE...SIA......ER.VR......H....C.TR......
tr|B9XNJ8  ...........................ELESLDS....KIRV............................C.TA......
tr|B9Z7Q7  ...........MPQLDYLAPTLARVHPAWETVLR..SPACFK...............ALSQIDRTLANEAAAGK......
tr|C0BNZ8  ....................MFLDCPSDWKEFFH....KLYS..AEKF......KK.LSHEIN.AL....Y.DE..M...
tr|C0X269  ..........................MHYPEHLK....KAVL............AK..............A.KD......
tr|C1A7L2  ....................MTATPPPRNEARIR....GISV......................................
tr|C1D9B0  ............MSLSCLAPTLNLVHPDWQPHLS....TALLAHLDHR.........LSARLESGE......TV......
tr|C1F1L1  ............LPPTVPHFMPSDSPIVPAAQRP....EALR..................VLREEI..GDC.TR......
tr|C1G9A4  ......GLTKEQKELLQLEIDTLD..ESWLAHLK....DEVL..TNEF......LN.LKRFLK.KE....V.AS..GA..
tr|C2MIJ3  .....IELYNVLQIEIIEVMRKGGGEMKYPDHLV....KQVK..............................ER......
tr|C3X2T7  ....PAFDVADAATASALSAETAAGPDSWEKLLG....EIGH............................C.ER......
tr|C5CIT9  ..................MTEYVKKKELMMGIIR....KIEE............................C.NL......
tr|C5NVK4  ......................MH..NSWNEVIK....NYPK..IDEL......NE.LIKKIDEKR....L.TT......
tr|C7H0A3  ......QILGEGMRFLEDEIDKLG..ESWKRALL....PETE..NKYF......KE.LSGFIE.GE....Y.EK..G...
tr|C7M312  .......................MGLPELEALEA....EVRA............................C.HA......
tr|C8W879  ....ALGRKRMSNVEYSKIAYSKQAGQTLDEICE....QIAD............................C.KK......
tr|C9M5Q7  ..............................MLAR....ELAG............................C.SR......
tr|C9RLM9  ....................MSVKLEESWLKLLA....DQFE..QPYF......KQ.IKAKLLQEH....AEHH......
tr|D0MK10  ...........APADPYARIEALIPPESPLRAMR.....TLDEVARYV......................A.ET......
tr|D0PPD4  ................QRDSVMDFLPQSWIDYLQ...LSTFS..SKKL......EQ.IMAAVENRR....K.QT......
tr|D0U4F0  ...................MVGWALTKDWSEQLD....FIFK..QDHY......RH.LLEFLEYES....AHNK......
tr|D1ART8  ......................MT..DTWKKLLE....EEKE..KEYF......KK.LEEFLD.KE....Y.EE..N...
tr|D1C3J5  ...............MVKDEEIAARSRRMEQIRR....AMLA..APEF......................A.TY......
tr|D1CDM9  .................MKGSAVSDQYELDRVRE....EALF............................C.QR......
tr|D1JDZ5  ......................MTNKEEMKRIEK....EIKD............................C.KR......
tr|D1PN05  ........VEEERFMLLHQQLTPLLDGDWAPFLT....AECS..KPYF......SE.LDSFVTEAA....A.AR......
tr|D1Y5H9  .........................MSALSALHR....RVCQ............................C.RN......
tr|D2EF83  .........................MESLNDISK....EIVK............................C.TL......
tr|D2Z573  ..........................MSWSKLRE....AVGT............................C.RG......
tr|D3B454  ........PDSSDPRDFSNILDYLKDESWKNALK....SEFE..KTYF......KK.IIKELNSTG....ANTK......
tr|D3E6H2  ................MNEVQSAPVDPATIAICR....ARLV..............................HQ......
tr|D3FBP7  ..............MSTAAERRERLKEVWREAST....................................C.TL......
tr|D3QZ35  ......................MSAPNGWEPIIL....SEMQ..QPYF......KE.LRRFIEAER....A.TY......
tr|D4G8B4  .................MKNKRKKIEFSWKKFIL....EESK..KKYF......LN.ILHQLNEAR....KSGK......
tr|D5C4I1  ............................MQTPLD....EQLE...............KVATLN..E....L.KQLCE...
tr|D5E730  ........................MVDSFEDLEE....EINS............................C.TA......
tr|D5EGK6  ..........................MDAFSLRQ....RIEV............................C.EK......
tr|D5EHP1  ..QVVHAAQLPPPPKIDLPEGDAKSQLAWLRERV....EQCE............................TCNS......
tr|D5MIW4  ..................RLGVERLYVAWPERAE....GLPEPSPTLTKVRETVGE..............C.TR......
tr|D5RKP0  ........MTHP....AWEPALAALPECWRAALG....PAFA..MPLM......DA.LAAFLQAER....AAGK......
tr|D5U040  ......................MSLNSEWLEIIY....EIQN............................C.KK......
```

```
tr|D5U570 ........EEKNITNKGIELMKEIKNEDLKKIYN....EVEK...........................C.MK......
tr|D5V6V9 .........................MKTWQDVIN....KQKE..QEYF.....QK.LENEINHLY......ET......
tr|D5WWR6 ..........................MAWWQNLN.....QLE...SAF.....KS..............C.RR......
tr|D7CKV6 ..........WGQDHYFAFLPGVKREESFLEISS....LEELAARAGA.....................C.SR......
tr|D7CWJ9 ......................MLVRAPWRGTLGPMTLSDLE...........QQ..............ARNT......
tr|D7UXZ6 ...........MTCSPAKFQINHFQQKEGKAMLP....KKLI...ETV.....KK.........R....A.AN......
tr|D8LFW4 ........AVSKRPKISSSEGSPIKEDGWATALS....GETT..KPYF.....GR.LQAFLDKQY....A.SK......
tr|D8UI66 ........ESSAPGGPKLQLSALLVEPGWRSALA....GELAGANLRQ........LDSFLGAEW....APGR......
tr|D9Q1V3 ................MSPVSLDNKKASYDDIAE....RVRN...........................C.KA......
tr|D9QQQ4 ....LFHDTKDQLGLFTEQDQEQQRFNNLDEIKE....VAVE...........................C.ER......
tr|D9S3H2 .........ISYGEIIKMVHDKGEQSAKMRDFLK....NEVL...KLL.....YE..............C.GE......
tr|D9SCW5 ....GEAADNSLREFQLKNSGGLLGTTDWPELQQ....KVQG...........................C.TE......
tr|D9Y2E5 ......YRPSFQRRRELMLESSIK..EDWKPVLS....DFEH..SEAG.....KK.IEQLLA.KQ....K.AE..GL..
tr|E0S9I8 .............NPCEYCKLEEKIDPKWRVYLK....SEFG..KDYF.....RG.IKRFLHKNK......NH......
tr|E0SQC9 ......................MSLDYSWQELEN....AIRN...........................C.TR......
tr|E0TBI7 ...........GSDEAVMAATALAEATTTLPALA....DAIA...........................GFDG......
tr|E0TIS2 ........MQNKLFKIFNKFNINLLKNKKNNKFN....KIYK..............LYNKINN......C.KK......
tr|E1IBI3 .................MAPSISAIIAALAQLVP....PANA..............INPYADDGAAGNTIRRA......
tr|E1L0U9 LGFFAPWARQASEVLMATNDADTHNVEKWFPSMQPAVQARVR...AIE.....AQ.LHTLIDVEH....K.TI......
tr|E1QFX8 ......................PDLPLELESMAL....AVGQ...........................C.KR......
tr|E1QNN8 ................MDIIKIKINVVGYDELVN....LMLN...........................C.TK......
tr|E1QWE2 PLPDGSPLPNTGSLLSISPRLADDHLDAWLDLLP....PETI...ERM.....ER.IDKSPELAD....G.TS......
tr|E1R614 .........................ALLLKEIAQ....EVSS...........................C.TA......
tr|E2IUG8 ......VSDRTLDWRRARDVFGIG..EPWRDVIE....PELA..AAAT.....SG.LLSEYF.RR....C.RT...E..
tr|E2SYG7 ...................MSSNDKEAKFLDLVH....RVTA...........................C.QK......
tr|E3D056 ............ETRSGEEPLFQLSPSERRHQEA....ILLEEARHRV.....ER..............C.LR......
tr|E3IP47 ................MRDPLAGT...GWLEAVPLLGDGAHL...EVF.....SR.VEALRRKGR......VV......
tr|E4KQ36 ...................MKL.KLNPSWQVALA....SILD..SPFY.....TA.LEARVIQAY......QT......
tr|E4RKG8 .......MLFDQNGNFSIQQLFSYPNDNYQKLKE....KALK...........................C.TR......
tr|E6N689 ...........................VLDAIAE....EVRA...........................C.RL......
tr|E6PFU2 ....................MSLEERARRERSLQ....QAAA..RASA.....................C.RA......
tr|E6W1H1 ..................MSFDFTLPDSWKAHLA....PLLQEPQCQR........LSQYLAQR.....CQQN......
tr|E6W552 ............PPRQTTTPAVIPSQDSWQRLEE....DLKD...........................C.QA......
tr|E7A9L4 ................MLFHALERLPPEWSAFLQ....PAIQ..NSHF.....KHLNHNYMQALTEAQKHQTR......
tr|E7FX98 ..........................MNWEQLFN....EEMK..KEYF.....KM.LERKIEDER......KA......
tr|E7H418 ..........QPAVGMSTELLSKIASADWQALQT....LSQH...........................C.TA......
tr|E7H4Y1 ...............MKPDFSTESLTGVWRTTVE....HFMA..SPEG.....QN.LVDRIS..S....C.TS......
tr|E8LLT1 .........................MVTWHDLIG....PLKK..TESF.....QH.VMSFVAQRR....AAGD......
tr|E8N338 ......................MQPDQILKEIAQ....QVSE...........................C.KR......
tr|E8T398 ....ELMGFKEVRLPEGVVERAFDPIEELEKVKE....EAQK...........................C.CK......
tr|F0EKF3 .....................MGGVFMQYPKELA....TAVQ...........QA..............S.RS......
tr|F0NSC6 ................KCKIFLINIIINILKKKS....GEMM...DYP.....KK.LLEEVK..E....RSKG......
tr|F0RSJ4 ..........................TNVRQLEA....LVAL...........................C.TK......
tr|F0SG07 ....AEGPDSVTRFMNAPVLSRSERESCLASLAE....RVAG...........................C.KR......
tr|F0Y985 ........TRRVVARAATNGGEWALPSGWRAALG....DELA..SDRF.....RD.LQAFVAAER....ASQT......
tr|F2LXL6 ........................KEEEFEKLKE....KALV...........................C.KG......
tr|F1A0X7 ........VEVTDSNYYDDIESYISDVNWKNALQ....DEFK..KTYF.....KN.LISSLNKVK....KENK......
tr|F2NFM2 ...........ELTLQPASEVPPQEVITLEEVRA....ELGD...........................C.QR......
tr|F3KRD3 ......SAERLRAWAPAEWLAPLT..PDWRALAQ....TFFD..GDAG.....RS.LGRQIQ.AR....L.DA..GA..
tr|F3QMU0 ....ASEARGVRPEAYTEARIHQIATADWQTLKQ....LVQD...........................C.RA......
tr|F4A8X0 ............LKQCIITLILFFKKEVFIIDII....NSLKYRIQEI.....................V.KE......
tr|F4C0J7 ...........................DLILLQE....KIAS...........................C.DL......
tr|F4GMI7 ........KAKQPAPESPEMPRLPVPETWEALIS....HIAG...........................C.QA......
tr|F4GSY3 ......QVQNAFQGDLLAQAASLP..PAWQDAFV....NMRE..QQTL.....HD.LDGFLD.ER....V.QE..GA..
tr|F4HNK0 .....................MPSKEELMKKLEE....RIKN...........................C.KK......
tr|F4NZD5 ........NKLSETQPLLQLEITTMPDDWFNAFR....SEME..SAYF......LNIKRFLQAER....DAKK......
tr|F4QWL4 .....APGAPPSVDEAAVEARRLAACATTLDELA....EAVA...........................AFEG......
tr|F5SL17 ....CDNTSIHPGGIQKQSEMGGGTGMDLESVHQ....RYAR...........................C.SR......
tr|F5Y215 ....................PGGVAEMDWDALAG....TVAG...........................C.RA......
tr|F5YGI3 ..............ALGVEKVTVTVSDTLEAVAA....DIVR...........................C.VA......
tr|F7NHV1 .........MYIYCEPEEKTLEVITVNLHTELIE....KLYQ...........................C.NL......
tr|F7PTA1 ..................MQYFPK..NDWYSILK....DEFT..KPYF.....IE.LQHKLK.NE....Y.RL..H...
tr|F7S094 .........................MVSWQAFMA....QEQQ..QSYF.....QK.LQQKLSEER....A.SG......
tr|F7XVS9 .........SESYTERSASRIMADNSDNFQELCE....AIKK...........................F.DG......
tr|F8ADN2 ....VNTLPARKNIRRFLDLDFTPPPKSLAEIEE....EIKN...........................C.TR......
tr|F8L559 ....................MVMTLEKGWHQALD....EELK..KPYI.....QA.LKTFLSEEK......KR......
tr|F9CXI5 ..................MPDNENMNEELETIRQ....NVKT...........................C.TK......
tr|F9DTW2 ........................MFRIPDDIAQ....TALE...........RS................EG......
tr|F9LK36 ..........................MRYPNDIL....RLVK...........QR..............S.KN......
tr|F9ZLD4 ............MF..................................................A....C.YR......
tr|G0LJW8 ..................MTGDDNHDQDTSSDLN....TATDAPGSAS........TVNGLDVTA....C.KR......
tr|G0QBL8 ..........................MSFRNRHR....ELLD...........................S.IE......
tr|G0QFK7 ...........MLKGQLKNLESMDLRTRYKDFFE....NLDS
tr|G0U4E4 .............LESNIDLFSLILDPGWRSFLEPLARTNFK.............GIEQFINHEI....AKGK......
tr|G2RZ19 .....................MNKINQSWLTLFE...RLAIK...........DQ.INNLLDQVY....NSNQ......
tr|G4RL69 ...................MDDRDIAKELWAIAQ....EVAA...........................C.TK......
tr|G4STH7 ....................KLSDSKTTWTDLQN....EVAA...........................C.RQ......
tr|G4T6H9 ......EPPPPAEQAILDLEQTMG..RDWYEALK....DEFT..KPYF.....RS.IRNFLI.AE....T.RK..KA..
tr|G7WNS9 ...........................PLRSLAE....EIGS...........................C.RR......
tr|G8BJ42 ........SLTPEQKDLLQLEIDTLHISWLSVLY....KELT..KPYF.....LN.LKRFLHSQQ....QQGK......
tr|G8LWR8 .......................MSKSEQLNILY....NRYG.............................KQ......
tr|G8TXV8 ...............................MTD....PQRE...........................C.RR......
tr|G8XT11 ......LSPD.....EQSRLFGIH..PSWITFLD....LFEH..ETAL.....LK.SVVDTV.RL....A.RQ..TE..
tr|G9EIR0 ...................TTYSACEEQLSALAK....EVAA...........................C.TR......
tr|G9XY09 ....................MWIKYAVGWGEFFD....NEIK..KPYF.....KQ.LLKNIDNEYQNKLC.........
tr|H0E6E4 ...............MADPVTAGERRAGLTELAA....RARA...........................C.ER......
tr|H0KUY0 ..........................MTWSEVLA....PIKS..SEYF.....ET.LWKKVNEQY....KTQK......
tr|H0Q5N1 ......................MSLPSSWMSILAELRLDAVVAQRVST........IEAELAARE....AAGS......
tr|H1D376 ............DPEEYAARRHRELYEETEKITY....PSHEALAEAI.....RS..............C.SR......
tr|H1P1U4 ............................MARIRQ....TSVP.............................AR......
tr|H1P505 ............DPIGCPSMEAVSSASSLQDLES....RING...........................C.LA......
tr|H1XWX4 ...................VQYRQTSPELQAFYE....EIRD...........................C.QQ......
tr|H2C8Z4 .........................MKSWEDIER....EILN...........................C.TK......
tr|H2J7L7 .......................MGKMEDMDIIS....EKIK......................K....C.KG......
tr|H3KDP1 ........MRAPDFSTAGL....T..GRWKTLVE....GFLA..SPQG.....EA.VVAKVA.DA....A.KS..AN..
tr|H3NN78 .....................MIG..NEWDIVLK....DIFD..SEEF.....NI.FMNNIY.MD....Y.EN......
tr|H3NSX2 .................MIKLWEQCPHAWRSTLD....QLINGSVGKG........LEAFLDAEI....TSGS......
tr|H3RRL7 .......MITEIFFIYEGESMIIEYPPSLLQQAE....DLLK.............................GH......
tr|H3SCZ2 ..................MIAGTALPEELQAIGN...LDALRGV.........................CERV......
tr|H4GIS2 ..........................MHYPSELV....EAIK...........................AQTV......
tr|H5SI33 .................MAPPAVDGAEALERIAR....EIRS...........................C.TR......
tr|H6RLZ5 ....AAPRPPRFDAAAEEVARTAAAAPDWPSLAR....SASG...........................C.LA......
tr|H7F338 ........NYDIVVNMRKDGETMSQELTYYETPD....SLVE...IVK.....KR.........S......ET......
```

```
tr|H8I7F5   ................MDPDLERMVELLNEID....DILC...........................C.PR......
tr|H8ZDF2   .............SSCDICEMEKTILPGWKTLIG....SEFE..KEYF......SK.IREYLHTID....FFPK......
tr|H9UI22   ...............SDLPGGNAAGPGGLLEAVA....TEVA..........................SCTA......
tr|H9ZZZ0   ......................MMSFISYDEFIK....QIEN...........................C.KK......
tr|I0GI60   ......................MDKEAMLKEIED....DIRH...........................C.MK......
tr|I0I6B7   .............MDALTSPGETSKEDGRLQALRCLEEEVVR..........................C.QR......
tr|I1A005   ......................MKDSFLRILQ....TEGQ..KPYF......RN.ILKKLEESE.....KN......
tr|I1B0L2   ...................MTP..PAAWAGL...........PFF.......RD.TYPVIARAI.....D.AE......
tr|I2K0D9   .....TLTPYQKQLLDLEINTMD..DSWLAVMH....EEMT..KSYF......LD.LKKFLE.GE....W.KS..GK..
tr|I3CUN9   ....IPDGDEEQDAPSVDPLIARLKTMDWPQLKQ....QVAG...........................C.RR......
tr|I3VQA4   .............TNPADQAKLLGIDIEWLRFLQ....LDDV...EFGKIKGVHDA.VTRDRY..R....Y.VV......
tr|I4CBR6   ........IGLNPVMIPEKDFGTTSDKENRLSLESIRTELGD...........................C.KR......
tr|I7LIA9   ................MDINIDALKEEYIKKLK....DELG.................................
tr|I7LKX1   ........MYKMIIRDIVNSLGMVQETGRQEAVERLAAVISE...........................C.RK......
tr|J0UVA7   ....AAPVPVEEVVQRPAVLIQPEKARDMASLLE....QAQA...........................C.QE......
tr|J5SPB4   ......YFKPAGAAATKSSTRATGSALSDAARDAIMKAKDLN..QEVPSPRPAKKA.........K....IANE......
tr|J7R1V5   .......SLDAKTRDLLNLELLTLEDSWFEQLK....EEFT..KPYF......LQ.LKDFVKREQ....STQT......
tr|J9E0C3   .........ISAERASEAGNISAAEAAAQQADTR....EALK...EAM.......EA.............F.EG......
tr|J9Z1B8   ...............MDTSTIQLHPSWLDHLK....DEFQ..KDYM......LLIKQKLLELKK......NN......
tr|K0IF13   ..................MAAQKQHDSLEKVAA....EVRG...........................C.PL......
tr|K0KF39   ........KLTEEQKHLLSLEINTIEDSWFEVLS....DEFL..KPYF......LS.LKKFLITQK....TAKH......
tr|K1JXC9   .........QAPALVLPPEVVAACEKADWATLEG....LAGA...........................C.TC......
tr|K1JYF3   ......TASLFSEGVMPDMTLPAD..PLWHELVE....AFMA..TDKG......RR.AVERVA.ED....V.AA..GH..
tr|K1LNP2   .............MYQLAHFSVVA..SDWAKAIE....AYYD..LKQL......DH.IENQLL.EL....S.Q...QQ..
tr|K1ZRE1   ...................MALNRTVQLKKIRD....EIWN..........................LSKS......
tr|K2A3B6   .....................MTKKEKLAEIK....KRME............AD...............KS......
tr|K2B4F4   ........................MTNLQEIIN....QCGN...........................C.RK......
tr|K2C359   .....................MDKQYAEIIE....AVNK...........................C.RD......
tr|K2CLQ5   ...................MNKVSSMQKIA....KEIE.......................N....C.EI......
tr|K2DEQ6   .....................MSEKEALLENIE....KNIK..........................VCQK......
tr|K2E7J1   ..................MKDVKIEKTWKEILK....TEFQ..KPYW......EG.LTEFVRQQY....LVGK......
tr|K2E9B4   ......MSTELLSAFNYSETDSKTRHKALNDVKE....VCKT...........................C.IK......
tr|K2ETW7   ...................IEPQVSADWQELEK....QVVA...........................C.SL......
tr|K2F640   .....................MTKFEELHKLK....EEME............KD...............DT......
tr|K4KIT8   ................MATIVEQLDAGWRAVFE....QELA..KPYM......QS.LRDFLKTRK....RSGA......
tr|K6GHM9   ...................MSKFKFPNAW...LS....PV.G..KAFC......KN.IFHEIETKL....IKKK......
tr|K7YPM8   .........NFDLNGAVEAARRAANRAASLDALN....VAVE..........................RFDY......
tr|K8ZAC1   ........................MDWSTFIQ....EEMK..QPYF......QL.LAQYLT.EE....Y.RC..Y...
tr|K9VTI9   ...........PKILSVESIPTSAKVPIPAGTY....NSIKGIRQHC......NN...............C.QR......
tr|L0A9A2   ................MIIMQDDKRIKISNLNE....LIKS...........................C.KK......
tr|L0B4R8   ......MSNKLIKKTLYKDFNNLN..PIWKNCFL....QNKL..EQSF......KE.IFSYVE.SE....I.DK..GV..
tr|L0B866   ........MKLLPNNLLGFIEMLP..KVWRDTML....SYAN..IMTV......QH.INKFIE.TR....L.SD..NA..
tr|L0B8A6   ............NNINTINVLQKDIDSSYKTTFC....NDISILKEKVVR...................C.KN......
tr|L0DFI2   ........PPSSLFGESERGNTIVPPAERPAQLAALAAEVSV...........................C.QR......
tr|L0L178   .........MNRNCNTPAMNSGELVKLGLKGITA....RVLE...........................C.KK......
tr|L2GQC8   .............SSCTVCKLETFVEKDWKFLLE....EEFR..KDYF......LK.IKKAL...H....S.TS......
tr|L2GX11   .............ASCKLCNLHLFISSKWLPYLK....EECH..KEYF......KK.IVMMLHNED.....IF......
tr|L5N8A0   .........................MEWPKSML....KDAE............RR..............M.EG......
tr|L8HA23   ........RPTELQPQVKHLIAGLHEPAWRRVLA....NEFT..EGYF......VQ.LAKFLEQER....Q.TK......
tr|L9X1W2   .............MGDSNGADTDVESTGWRFE......SEFA...DAL.......EG.VP...........V.DQ......
tr|M0BKB2   ...............MSSKSPSDPTDPAFPTSRH...VVEPD...........................C.DR......
tr|M0CBA6   ....................MSALDFEEAFADELA....AVPE...EFV.......DE...............ER......
tr|M0MGS7   ...............RGESADRNGLKPAERTLIM....PEYP............DPDSKNVLAPG.....C.ER......
tr|M1E4Q4   ....MYIPSLFDNLEEENKQKENSSKLKLSDILI....EAKN...........................C.KK......
tr|M1LNF1   ......MSNRLTSKDIYKNFELIS..CSWKEEIS....KYFS..LNNL......RN.TFNFLE.NR....L.SE..GI..
tr|M1QKH3   ........KNQETLNNNSATIYKSTASDLLQQLKAFYETCKD...........................C.KK......
tr|M1VCA8   ........PAGYALAATATALEPSLPSCWRQALA....AHFQ..SKSW......AS.LNEFLQ..KEIAQG.KR......
tr|M1Z9H8   ....................MKKQMEKWKGDRV....KEER.MKELN......KE...............IMEK......
tr|M2XVU5   ........RFVESLGTLVDELLVDLEPSWKKQLY....PMLD..QKTL......EN.LAQFVLQER.......KR......
tr|M3EFG6   ......................MYQISKELAELGK....ERIN...........................G.......
tr|M4YU22   ............................MSFKT....PEKD...........................C.SL......
tr|M5ADY8   ...............MMILKEKSEINFDWISLLK....DEFH..KPYF......IK.LLKILRIEYNRFIC.........
tr|M5IVB6   .....LSGNLLTENILNQIARLP..SDWRATLD....RPEL..QDIL......AT.LAKWLD.KE....C.QD..GA..
tr|M6D8H9   ........NWLQDNRFSLSIRVGMSETSKEQELEIIAREVAP...........................C.VR......
tr|M7WR77   ......GSSPTESDLLALECATLH..PSWLEHLR....DEIR..KPYF......LD.LKRFLW.KE....G.LK..GEKD
tr|N0BBN4   ........................MLTSELDEIAK....EIKK...........................C.RK......
tr|N1V4N6   .........MAQPKTVAKSLPAANPVRAFVELLAGTETGASS...TNF.......LD...............C.SD......
tr|N1Z687   ...............MICIKGVGKVEEMEILKQKITTEVLQ............................SD......
tr|N2BLL9   ...............MLIPKTLS..PQWVNLLK....DEFS..KPYF......LE.IIKHYK.QA....L.QT...HKN
tr|N9SHT1   ........GENIFLFFIAIILKMKTKLSFESFFR....SETK..KTYF......HE.LMKKLEIEY.....KN......
tr|N9V498   ..............MICEHLNSFTKKNKFYDFLE....NESK..QDYF......KL.ILEKVNNSS......KN......
tr|Q025I0   .......ALAAPEPKMELPPLAPPNDSLLKIIQ....DIGD...........................C.HR......
tr|Q0C0Y4   ...............AETIAAGCANLDELTAAA....GQFS............................GS......
tr|Q0EZR7   .........LESQVSTPAPAADTVVFKSLDELSA....TAST...........................C.RL......
tr|Q12TN4   .......................MKNEWTTLEG.VEKDILD............................C.TA......
tr|Q1AV32   ..................MGDREAWERLRQ....SSAG.................................
tr|Q1AYQ1   ..........METLFDAVEGGENREERLAELAR....QVSV...........................C.TR......
tr|Q1GY28   ........MSTMLDPVTHPRPESLPPAWVEAI.....PELQ.ALYHS..........ISASLEPEQ....DVR.......
tr|Q1N1I3   ...................MANKITDKLEGQWKQHFD....LEMS..QDYM......AQ.LRGFLKEEI....AQEQ......
tr|Q23W06   ........KEKLAGEFPTFDEFQDDLCSWKDILG....TYIK..SQKF......KS.IYTFVKKEYADKTC........
tr|Q3SA81   ..................MQTSLFSFDSAGDSLD....DKIR........................E....C.KK......
tr|Q53CZ3   ..............NETPTQMLLISDSWLKFLN....LSPFLKKKLA......AL.LRRVMDMSK......AT......
tr|Q5DC15   ........RLSNRSNKNVLNLIQGISTEWFAILL....EQIE..HDKF......QQ.LADFITKEQ....NSSV......
tr|Q5WES9   ......................MISKQLQELGK....KRIA...PYP...........................C.EG......
tr|Q6CAC0   .........GLKPADKELLDTEITHIHDSWLPHLH....KEIS..KPQF......LN.LKKFLASRE....AAGV......
tr|Q6F1M3   ....................MNKKWNSLLT...NQDLS..............KR.IENTIELAY.....KTNE......
tr|Q6KIL8   ................MNSDRREHPSFISFLE....KERK..KIYF......KE.IIGFLFKTN......KE......
tr|Q7VBS6   ............................MNLSNSLH.....DTR...........................C.HS......
tr|Q8TK31   ............EIVAAEEENCGNFEERVQKLVE....AGYE...TVA.......RE.AI......A....C.TR......
tr|Q9RTK9   .....MTGSLPPPRHPLHQAADPPDPAALLALED....RNRG...........................C.AA......
tr|Q9WR44   ........CEQNVANTTSLLDEFCVDPEWHSLLR....VEFE..KPYV......RH.IFEQYRQRV....NNGE......
tr|R1ATM3   .....................MKEQELQKLNN....KILE............................EH......
tr|R1E4B2   ......................MDKEKMIEELNN....QILK...........................C.TR......
tr|R1FRL1   ...............EVASLIEHLTDPQWREALR....GEVC..KSYF......AQ.IARAVSLER....G.KK......
tr|R1GL12   .............VCKDSASLSMNNLENALDALY....QEIK..........................AFEG......
tr|R2SFV7   .......................MNYPDWLTKSV....ANRS............................QG......
tr|R2SXC9   ......................MHYPKKLVEAVE....QKMQ............................GH......
tr|R4KRR4   ......................MKDKALLELYN....RIVN..........................CRCP......
tr|R4VE87   ..........................WARLER....EITR...........................C.EA......
tr|R5ARL3   .............MCRKQFELERAIAKERARLLL....LAPK............................DQ......
tr|R5B4U8   ......................MTDALRLLYDAER....ARLS...GLS.......TQ..........N....N.GD......
tr|R5DX78   .....................MMKKDCLEIVRS....KCES...........................C.KA......
tr|R5MPG0   .....................MFG..NDWDSVLS....DVVS..EKWF......ID.MIDSVR.KE....Y.NS..K...
```

```
tr|R5PZ23 ........AQRPLAADAGTALLAQKASSLEDLKS....VMLN............................F.NG......
tr|R5QX00 ...........TTGLAQATLNATQNAKDLCASID..SLDELRKAVENF........................DG......
tr|R5Z1D7 ......................MKMNNDWQALFD....QEIQ..KDYL......KK.IDYFLAREY....K.TK......
tr|R6HGV3 ...MALXGPLGPRMFFGVEVNNEQDINAWLSGAS....QKVVETTSRL.........MGAVEELRK......TE......
tr|R6HL97 ......................MTISESWDNALK....DEYA..KPYF......TN.LMQRVNEEY....A.TH......
tr|R6PFR8 .........MKHLLVDVKCDSAQIKEKALIELKN....VCCQ............................C.KA......
tr|R6PI86 ...............MSEVLSLKNSEELLEKTRQ....KCAE............................C.QK......
tr|R6R865 ..........................MEWKDFFN....SQRE..LPYY......QN.LHEKVLQEYN...C.KI......
tr|R7BZ83 ..........MRDFARERHLWEGA..GSWQPLCT....EFLK..SPAG......ES.LLARLD.AR....L.EA..GA..
tr|R7DQ64 ......................ME..RTWEKFFE....EEKQ..KEFM......HR.LHNFLA.LE....Y.RS..H...
tr|R7DXQ1 ............PELFPESREPASTVALPEGTME....DKLQ....YL......RD.LA.............QN......
tr|R7I8P1 .......................MPDLSWELFER....QVSQ............................C.QM......
tr|R7IAS2 ....AAHPDSAAGGWDSVKFFSQVTDGTWDQLKA....LLPE............................C.HG......
tr|R7IHQ8 .....................MPSELERFNERMQ....AFFA...PLW......PG.........E......EK......
tr|R9ADN8 ........DRTKPPSDLLALEYKFLGPGWVQALQ....KDLN..SPSF......KR.LKEFLKQEE......QK......
tr|R9LKN0 .....................MIN..NEWNELLE....RESQ..KPYM......KE.LKKIL...V....S.EY..NTQ.
tr|S0EV15 ............MGEETDNRFAEMSSDEGSGTKA....ERLQLVAARA......AT..............C.TA......
tr|S0FG57 ...................MTTSKIRMELDELYN....KYSK............................T.........
tr|S2J4F7 ......GADQETLELLDLEITTMN..YEWLKVLA....PEMT..KPYF......LK.LKRYLK.AE....L.AA..KK..
tr|S3JT40 ........IAGSASGGMYTEAVGGSGGALFRSMQEIYAAVAQ............................C.SA......
tr|S4NBY8 ..FLFYTEDKNKTKEGHTMTLHTILTPEWIHDAK....LMAA..............................EN......
tr|S5MFT9 ...........MKSYWNKTEIFEKIDIEWIQIFK...KNGIK............ND.IFSIFSKIK....NINN......
tr|S5RQ34 ....................MTLKLEISWKYHLR....KEFR..KPYW......RK.LANFVQYEYANKIC.........
tr|S6G4W1 ..................KSSSNANMLQWVEEARKAAANCVD...............LAELRAAVC....AFEG......
tr|S9VE50 ..............GGNGWLGDLITEPSWRKELQPVLQDAAQ............GKGLLSQIEKFIDTAEGKGA......
tr|T0AYQ1 ........EDEQDTNSFKDLEDKYLHESWSCMLK....SEIE..KPYF......KR.LKHFLEEEL....KRGK......
tr|T0DRC9 ........MKLFGIKRMKLFDCAPLSLAWREFLQ....SEFK..KPYF......LEIEKRYLEALK....GPKT......
tr|T0LSQ5 ............................MNRIIR...............................S....C.TK......
tr|T0MGQ8 .............CKCEICLIELLICEKWREILK....DEFS..KNYF......LN.LKKLLHSSP.......F......
```

```
                         40                      50                 60
sp|P12295 .......IY......PPQ..........K....DVFNAFRFT..E.LG.....D.VKVVILGQDP.........YH....
tr|Q8KLI8 ........C......RLA..........E....GRTRVVFGE.GN.PD.....A..KLMIVGEGP...............
sp|A1QYK3 .....DKIF......PSP..........K....LIFNAFDSL..P.FK.....D.IKIVILGQDP.........YH....
sp|A1SUE5 ......NIY......PPE..........H....LVFNAFNLT..P.LE.....N.IKVVILGQDP.........YHR...
sp|B0B8I2 .....AKIY......PKK..........E....NIFAALRST..P.FD.....Q.VRVVILGQDP.........YH....
sp|B1AJI8 ......VVF......PLK..........K....QRFRCFDFF..DIEQ.....T..KVVILGQDP.........YH....
sp|P12888 .LRTQATVY......PEE..........D....MCMAWARFC.DP.SD.....I..KVVILGQDP.........YH....
sp|Q057V4 ......IVY......PKK..........N....MVFNAFLFT..P.LS.....S.IKVVILGQDP.........YHS...
sp|Q315T1 ......TVY......PPE..........G....QVFAALHCT..P.LH.....T.VRVVIVGQDP.........YH....
tr|A0LFG4 ........C......RLH..........S....ERTRIVFGE.GS.AH.....A..PLVFVGEGP...............
tr|A0LH32 ...............NMG...............DVF..VPGD.GS.ME.....D.GVIVLIGEAP...............
tr|A0LRS0 ........C.....AELA..........A....NRRHVVVGV.RPAVA.....R..PVVLVGEAP...............
tr|A0RV64 ........C......GLC..........E....GRTNAVPGT.GP.AS.....A..RAILVGEAP...............
tr|A1BDC5 ........C......RLS..........A....SRKNVVFGE.GD.PR.....A..ALVVIGEAP...............
tr|A1RYC0 ........C......PLS..........R....SRTHTVPGE.GN.PC.....S..GVVFIGEAP...............
tr|A1US05 ........C......SLK..........M....TAKNTCFSD.GT.AG.....S..PLMLIGEAP...............
tr|A4GHT2 ......IIY......PTE..........E....NIFSSLKLT..P.LN.....K.TKVVILGQDP.........YH....
tr|A4HXS3 ......VIL......PPA..........T....DIFNAFNSC..P.FR.....G.LKVVLLGQDP.........YH....
tr|A5KS51 ........C......PHL.........AK....TATNLVMGD.GN.LD.....A..KIVFIGEAP...............
tr|A5UQ47 ........C......PAL.........VA....SRRRIVHGY.GD.VM.....S..GIVFIGEAP...............
tr|A6DBB2 ........Y......LNE..........NIDFTDVFDPFLDN..K.TD.....N..PIMIIGEAP...............
tr|A6DK00 .....YSVY......PKQ..........E....DLFKAFDYC..D.FK.....D.VKVVILGQDP.........YH....
tr|A6GN76 ........C......GLC..........E....TRKQTVFAD.GI.PH.....A..PLMVVGEGP...............
tr|A7ANQ0 .....KRVY......PPE..........H....LVFNAFQIV..P.LS.....K.VKVVIVGQDP.........YH....
tr|A8A982 ........C......DLC..........K....VRKNPVPGE.GD.EG.....A..EVVFVGEAP...............
tr|A8E1M5 ........Y......PEK..........S....DVHRWSRLC.FP.YD.....V..RVVILGQDP.........YHD...
tr|A8F067 ........C......KLK..........K....FSTNTVFGD.GN.QK.....A..QVMLIGEAP...............
tr|A8MA01 ........C......PLY..........L....SRKKAVPGE.GN.PR.....A..EVMLIGEAP...............
tr|A8SNH5 ......TIF......PPN..........E....CVFTIFDKI.SP.KD.....I..KVIILGQDP.........YH....
tr|A8UAT5 ...............SQH..........F....NVEGFVYGQ.GP.VN.....P..KFLLIGEAP...............
tr|A8UU74 ........C......ELH..........R....NRTQVVFGD.GN.PY.....S..PVVFVGEAP...............
tr|A9AZ06 ........C......PLC..........R....TRTNAVPGE.GP.AN.....A..EILLIGEGP...............
tr|A9BHU0 ........C......PLN..........L....TRTNTVPGS.GN.VD.....S..PIMFVGEGP...............
tr|A9F5H8 ........C......PLH..........E....GRTHTVFSR.GD.PS.....S..EVVFVGEGP...............
tr|A9NHD4 ......TIY......PPK..........K....DWFNAFLYT..S.VN.....N.LKVVILGQDP.........YH....
tr|B0VFE1 ........C......PLH..........N....SRRKFVYGE.GN.PN.....A..IAMLIGEGP...............
tr|B1GA08 ......TVY......PTD................VFRALRLT.SP.DD.....V..KVVILGQDP.........YHG...
tr|B1H0K1 ........C......PLG..........E....KRLNAVFGC.GS.PD.....A..DLVFVGEGP...............
tr|B1I4W5 ........C......PEL...............SPCRPVPGQ.GS.RR.....S..RLVILGEAP...............
tr|B1VA82 ......VIY......PET..........K....DIFTAFHLT.AF.HK.....V..KAVILGQDP.........YH....
tr|B1Y7I1 ......PVF......PRQ..........P......LRALRAL.EP.AN.....V..RVVILGQDP.........YH....
tr|B1ZZ66 ........C......AHV..........R....PGKKVVLGV.GS.LE.....A..KIMFVGEAP...............
tr|B2A668 ........C......HLR..........E....TCNGVVFGE.GD.EQ.....A..DIMLIGEGP...............
tr|B2KCZ8 ........C......PLG..........E....TRLNAVPGE.GN.AD.....A..KLMFIGEGP...............
tr|B3DV47 ........C......PHL.........VS....FRTQTVFGT.GN.PY.....A..ELMFVGEAP...............
tr|B3U4N1 ......AIL......PPS..........Q....EIYAALETT..P.YE.....Q.VKVLLLGQDP.........YHT...
tr|B3U4U7 ........C......PLA..........KL...GRHQVVFGV.GN.PH.....A..SVMFVGEAP...............
tr|B5II11 ........C......GLA..........A....TRQHVVVSR.GD.PA.....A..RLMVIGEGP...............
tr|B5LRM7 ......EVL......PPK..........N....EIFTWTRYC..A.PS.....D.VKVIIVGQDP.........YHQ...
tr|B5Y8I5 ........C......ELC..........K....TRRNVVFGS.GD.PN.....S..RLWLVGEAP...............
tr|B5YEZ9 ........C......SLW..........Q....QRTNLVFGE.GN.PN.....S..SIMFIGEAP...............
tr|B6A3R0 ........C......DLY..........R....NATQIVFGE.GP.GK.....A..EIVLVGEQP...............
tr|B6AFI4 ......KVY......PSE..........Q....MMFLAFKLT..P.IS.....K.VSAVIIGQDP.........YHQ...
tr|B6K2R2 .......VF......PPK..........E....DIYSWSRLT..P.LN.....K.VRVIILGQDP.........YHN...
tr|B6KW10 .....YRVY......PPS..........R....LVFNAFKQT..P.LN.....A.VKVVVVGQDP.........YH....
tr|B7FQS1 ......TVY......PPV..........A....DTWSALNLT..P.LD.....Q.VRVVIIGQDP.........YH....
tr|B7KM64 ........C......GLH..........K....NRTKVVFGS.GT.LE.....A..DIMLIGEAP...............
tr|B8C427 ......TIY......PPV..........G....DIFSALNMC..P.LD.....N.IKCVIVGQDP.........YHQ...
tr|B8CX81 ........C......QLR..........Q....GCTQVVMGY.GP.ID.....R..KIMFVGEGP...............
tr|B8EJL3 ........C......GLK..........T....TATQLVFGD.GE.AG.....A..KVMFVGEAP...............
tr|B8HFW5 ...............ALR...............RDNLALYLQ.GM.LE.....RRPSILLVGEAP...............
tr|B8I5K9 ........F........................NDKEIVLGH.GC.ID.....S..PVAIIGEAP...............
tr|B9KXL9 ........C......DLW........RTR......TQAVPGE.GD.PH.....A..EVMFIGEAP...............
tr|B9XNJ8 ........C......PLY..........K....SRTLAVPGE.GK.FN.....S..KVMIIGEAP...............
tr|B9Z7Q7 ......TLY......PPR..........E....QIFHALSYA.AP.AD.....V..RVVILGQDP.........YH....
tr|C0BNZ8 ......VCY......PAK..........E....QLLNAFFQC..P.FV.....G.LKVVIIGQDP.........YH....
tr|C0X269 ........L......PLE..................GFVEGQ.GP.QQ.....P..KVMLIGEAP...............
tr|C1A7L2 ...............PML....................VPAD.GP........V..HVLLVGEAP...............
tr|C1D9B0 ........Y......PPN..........P....DILRALYDT.GP.AD.....V..KVVILGQDP.........YH....
tr|C1F1L1 ........C......QLA.........FE....GRHKIVFGD.GD.PS.....A..RLMFVGEGP...............
tr|C1G9A4 ......KIF......PPF..........D....EVYSWSRHT..P.LH.....T.VRVVILGQDP.........YH....
tr|C2MIJ3 ........S......APY...............QLEGFLSGQ.GP.EN.....P..KFMLLGEAP...............
tr|C3X2T7 ........C......HLC..........K....TRNKVVPGI.GD.RE.....A..DWLFIGEGP...............
tr|C5CIT9 ........C......PLH..........L....TRTNVVPGE.GS.LD.....S..PVVFVGEGP...............
tr|C5NVK4 ......TIY......PPK..........E....QVFRVFDLA...LE.....D.IKVVILGQDP.........YHN...
tr|C7H0A3 ......KIF......PEK..........R....NIFRAFRET..E.YE.....D.LRAVVLGQDP.........YHN...
tr|C7M312 ........C......RLS..........A....GRHQAVPGE.GA.AP.....A..PLLVVGEGP...............
tr|C8W879 ........C......PLW..........E....TRTNIVFG..GPNPT.....A..RIMIVGEAP...............
tr|C9M5Q7 ........C......GLR..........A....GAKAPVFGS.GP.RE.....S..RVVLVGEAP...............
tr|C9RLM9 ......VVY......PPG..........P....QIFAALDYC..P.VD.....K.VKAVIIGQDP.........YHN...
tr|D0MK10 ......VLI......PID..........A....RRTNPVFGV.GN.PE.....A..DLMVIGEAP...............
tr|D0PPD4 ......IVY......PAS..........E....DVMRWAFSC.QP.EN.....V..QVVILGQDP.........YH....
tr|D0U4F0 ......TIY......PPK..........D....QMFNAFDLS..S.FK.....N.TKVIILGQDP.........YH....
tr|D1ART8 ......EIY......PER..........K....NLYNALDLT..S.YE.....G.TNVIILGQDP.........YH....
tr|D1C3J5 ........C........................NEKRPVFGE.GA.LT.....A..DLVLIGEAP...............
tr|D1CDM9 ........C......TLW..........Q....TRTQVVFGA.GK.AG.....S..KIMFVGEAP...............
tr|D1JDZ5 ........C......ELW..........K....TRTNPVVGE.GA.LS.....A..RILFVGEAP...............
tr|D1PN05 ......TVY......PAP..........E....NIFAAFRAC..P.AH.....K.VRVVILGQDP.........YHE...
tr|D1Y5H9 ........C......PLG......QADGVREDGSAFYHVPGE.GP.EN.....A..SLMIVGEAP...............
tr|D2EF83 ........C......ELC..........L....KRTNAVPGE.GN.EK.....A..DIVVIGEAP...............
tr|D2Z573 ........C......SLW..........E....TRNNAVFGE.GP.ED.....S..PVLLVGEAP...............
tr|D3B454 .....EPIY......PPK..........D....EIFTALNWT..P.LD.....K.VKVVIVGQDP.........YH....
tr|D3E6H2 ...............PVE..................GFLLGR.GV.RF.....A..KIMFIGEAP...............
tr|D3FBP7 ........C......ALS..........E....TRQTVVFGA.GN.AD.....A..DLMFVGEAP...............
tr|D3QZ35 ......NVY......PPP..........H....AVFNSFNYC..P.PE.....K.VKVVIVGQDP.........YHQ...
tr|D4G8B4 ......EVY......PKD..........E....DIFNAFYYT.EF.HK.....I..KLVILGQDP.........YH....
tr|D5C4I1 ......HYL......GSEVSK.......G....EKFVF..GE.GS.PQ.....A.E.VMIIGEAP...............
tr|D5E730 ........C......QLH..........D....SAIHKVIMK.GG.QN.....P..KVLFIGEAP...............
tr|D5EGK6 ........C......ELC..........G....MRQQPVIGE.GL.AE.....C..RVMLVGEAP...............
tr|D5EHP1 ...............NLN...............LDGRIVFGE.GS.PN.....A..DIFFCGEAP...............
tr|D5MIW4 ........C......KLH..........K....DRKHIVFGI.GN.PK.....A..WLVFVGEAP...............
tr|D5RKP0 ......AIY......PPE..........P....LVFAALRHT.PL.AR.....V..KAVILGQDP.........YHR...
tr|D5U040 ........C......RLH..........E....NRINTVPGE.GP.LT.....A..KVVLIGEAP...............
```

```
tr|D5U570  ........C.....EALC..........N....SRLNVVFGR.GD.EE.....P..DIVFVGEAP...............
tr|D5V6V9  .....TTIF......PEK..........K....NIYKAFDTT..P.LD.....E.LKVVILGQDP.........YH....
tr|D5WWR6  ........C......GLR..........G....GCSQVVVSR.GT.PG.....P...VVLVGEAP...............
tr|D7CKV6  ........C......RLR..........S....GCRQVVFGE.GD.QK.....A..DLMLVGEGP...............
tr|D7CWJ9  ...............PKP..........AALAELSDNLVFGE.GN.PD.....A..DVVIVGEAP...............
tr|D7UXZ6  ........Y......ALE..........G......F....VI.SPDVE.....H.PTLMLVGEAP...............
tr|D8LFW4  ......VIY......PPR..........D....KLFNAFDSC..P.LS.....N.VKVVILGQDP.........YHQ...
tr|D8UI66  .....KPVF......PPK..........D....CIFQAFNAC..PFYQ.....V..RVVILGQDP.........YH....
tr|D9Q1V3  ........C......PLH..........R....LRTNAVPGE.GN.LS.....A..KIMLVGEAP...............
tr|D9QQQ4  ........C......ELH..........K....ECIQTVFGT.GN.SD.....T..GLMFVGEGP...............
tr|D9S3H2  ........C......PLH........LAE....CHTQKVPGD.GD.WN.....S..PLMLIGEGP...............
tr|D9SCW5  ........C......KLR..........A....ECTQPVFGT.GD.PH.....A..RWLFVGEAP...............
tr|D9Y2E5  ......KIF......PPK..........P......YRALELL..S.AG.....E.VKVVILGQDP.........YH....
tr|E0S9I8  ........L......PPI..........D....KIFTFSKFF..PLEN.....T..KVVIMGQDP.........YHN...
tr|E0SQC9  ........C......QLY..........R....NRKNAVPGE.GP.KD.....A..FVMFIGEAP...............
tr|E0TBI7  ........C......PLK..........A....AARRTVVYD.GV.IG.....A..RLLIIGEGP...............
tr|E0TIS2  ........C......ILH..........K....NKIRNVLGN.KN.IK.....K.IKYFFIGEGP...............
tr|E1IBI3  ...............NLH...............RALERALAH.GP...........DLLLVGEAP...............
tr|E1L0U9  ........Y.....PPLN...............RLFYAMQCT..P.PN.....C.VKAIIIGQDP.........YH....
tr|E1QFX8  ........C......PLA..........H....GRNKAVFGQ.GP.DD.....A..RLMFIGEAP...............
tr|E1QNN8  ........C......KLH..........K....SRRRVVPGE.GP.LN.....A..KIMIIGEAP...............
tr|E1QWE2  ........F.....LPPR..........E....DVLNALRLT..D.PD.....A.VRVIILGQDP.........YHE...
tr|E1R614  ........C......RLH..........E....GRHHAVPGE.GV.VH.....P..MVLIIGEGP...............
tr|E2IUG8  ......EVL......PPR..........G....DVFSWTRSC..A.PD.....D.VRVVIIGQDP.........YHQ...
tr|E2SYG7  ........C......PRM.........AG....SARVLGPGC.GP.LD.....A..PLMFVGEAP...............
tr|E3D056  ........C......PLG..........K....TRNKSVFGE.GN.LW.....G..KLLFVGEGP...............
tr|E3IP47  ........Y......PPS..........G....QVFAALRAT..P.WD.....R.VRVVVLGQDP.........YH....
tr|E4KQ36  .....NQIY......PNQ..........D....QIYAALNAC..D.FD.....Q.VKVVILGQDP.........YH....
tr|E4RKG8  ........C......QLR..........E....GCSNVVMGE.GN.LD.....N..KIMLIGEGP...............
tr|E6N689  ........C......PLW..........R....GRRNAVPGE.GN.PR.....A..ELMFIGEGP...............
tr|E6PFU2  ........C......AIG..........A....QRRNNVYGE.GD.PL.....A..DLMLVGEGP...............
tr|E6W1H1  .....IPMY......PPR..........E....DIFRAFRLT..P.WD.....Q.VKVVIVGQDP.........YH....
tr|E6W552  ........C......ALA.........RQ....GRKTIVMGE.GN.RQ.....A..DIMLIGEGP...............
tr|E7A9L4  .......VF......PLP..........N....ALFKTFEAT..P.LE.....S.LHTILLGQDP.........YHGVFT
tr|E7FX98  .....FTIY......PPK..........D....LVYTAFDYT..K.FE.....D.VRVVILGQDP.........YHQ...
tr|E7H418  ........C......ALS.............AMGRQHVVFSD.GG.PG.....P..KLVVVGEAP...............
tr|E7H4Y1  ......DVF......PPH................PFRALEET..P.FE.....Q.VRAVIFGQDP.........YHT...
tr|E8LLT1  ......VIY......PAD..........C....DIFNAFKYT.SP.DK.....L..KVVILGQDP.........YHE...
tr|E8N338  ........C......QLH..........Y....SRKKAVPGE.GP.AQ.....A..EIMMIGEGP...............
tr|E8T398  ........C......RLC..........K....TRHNVVFGE.GD.PQ.....T..NLMFVGEAP...............
tr|F0EKF3  ........Y......PLE.....................GFVPGQ.GP.LQ.....P..KLMIVGEAP...............
tr|F0NSC6  ........M........................RLEGINSGS.GP.KH.....P..LLMIVGEAP...............
tr|F0RSJ4  ........C......RLC..........E....TRKQVVFGE.GS.IP.....A..RLMVIGEGP...............
tr|F0SG07  ........C......PEL.........AR....TRTQTVFGV.GN.PM.....A..EVMFIGEAP...............
tr|F0Y985  .......VY......PPP..........D....ETLRALRSV..D.LD.....G.VRVCVVGQDP.........YH....
tr|F2LXL6  ........C......ELH..........K....TRTNVVFGE.GN.IN.....A..DLMFVGEAP...............
tr|F1A0X7  .....KPIY......PPK..........D....EVFSAFNYT..P.LK.....D.VKVVIIGQDP.........YH....
tr|F2NFM2  ........C......KLW..........R....TRTHLVFGA.GD.PH.....A..RLVFIGEAP...............
tr|F3KRD3  ......VIY......PPR..........P......LRALELT..P.LS.....A.VRVLILGQDP.........YH....
tr|F3QMU0  ........C......EIS..........S....SRLHPVFGQ.GY.PP.....C..RLMLIGEAP...............
tr|F4A8X0  ........Y......NSE...............ETGGFITGD.GP.IP.....C..DVLFIGEAP...............
tr|F4C0J7  ........C......PLS..........L....SRNLTVPGS.GP.SK.....A..EIMLVGEAP...............
tr|F4GMI7  ........C......GLH..........V....GRSHAVSGA.GA.EH.....M.PEWLVVGEAP...............
tr|F4GSY3  ......QIF......PLR..........P......FRALLET..S.PE.....A.VQVVILGQDP.........YH....
tr|F4HNK0  ........C......PLW..........E....LRTNPVPGD.GS.YN.....A..KVMFVGEAP...............
tr|F4NZD5  ......VIF......PLL..........E....NIYSFTQCP....LK.....K.IKVVIIGQDP.........YH....
tr|F4QWL4  ........C......PLR.............AMGARQSVFAR.GN.PQ.....G..SLMIVGEGP...............
tr|F5SL17  ........C......PLH..........R....GRTRIVLGE.GN.PA.....S..PLMFVGEGP...............
tr|F5Y215  ........C......KLC..........Q....SRRNTVFGV.GD.RQ.....A..DWLIVGEAP...............
tr|F5YGI3  ........C......PLH..........G....GRSQAVPGE.GV.ER.....P..LVMVIGEGP...............
tr|F7NHV1  ........C......HLC.........NE....GNLGPTAFN.GS.MA.....S..PIAIVGEGP...............
tr|F7PTA1  ......TIY......PLQ..........E....HIYKAFELC..S.FN.....D.TKVVILGQDP.........YH....
tr|F7S094  .....VVVY......PPE..........H....DIFKAFELT..P.IE.....Q.VKIVILGQDP.........YH....
tr|F7XVS9  ........C......EIK..........E....AAINTVIYD.GQ.VD.....A..KIMVVGEAP...............
tr|F8ADN2  ........C......KLH..........R....TRKNIVLGE.GP.ET.....A..RLMFIGEAP...............
tr|F8L559  ....GITVY......PPE..........P....YVFNAFRQT..P.FD.....Q.VKVVIMGQDP.........YH....
tr|F9CXI5  ........C......KLS..........T....TRTNSVPGK.GN.FK.....A..DVIFVGEAP...............
tr|F9DTW2  ........F........................QVEGFVRGE.GP.ES.....P..TLLLVGEAP...............
tr|F9LK36  ........F........................DLEGLVEGS.GD.KE.....A..NIMFIGEAP...............
tr|F9ZLD4  ........C......PAL.....CASRKQ....IVLPDV.SD.AM.LH.....L..PLLVIGEAP...............
tr|G0LJW8  ........C..................QGLVESRSQIVNGV.GP.AD.....A..ELVFIGEAP...............
tr|G0QBL8  ........F........................DDERFVPSV.GP.VE.....A..DVVLIGEAP...............
tr|G0QFK7  ...............SFW...............DEERYVPAV.GP.ED.....A..DTVLIGEAP...............
tr|G0U4E4  ......KVL......PPK..........E....FIFSAFNST..P.LD.....G.LKGVLLGQDP.........YHN...
tr|G2RZ19  ......VVF......PSR..........K....DVLKLFELS.DF.NK.....I..KVVIIGQDP.........YH....
tr|G4RL69  ........C......RLH..........L....MRKRAVPGE.GS.AE.....R..GIVIVGEAP...............
tr|G4STH7  ........C......SLC..........E....SRTQTVFGS.GS.AS.....S..GWMVVGEAP...............
tr|G4T6H9  ......TIY......PKT..........E....DIYNWSRLT..P.LE.....D.VRVIILGQDP.........YH....
tr|G7WNS9  ........C......PLA..........E....GRTKAVPGE.GP.AR.....A..EILLVGEGP...............
tr|G8BJ42  ......TIF......PKP..........Q....DIYSWSNLT..PLPK.....V..KCLILGQDP.........YH....
tr|G8LWR8  ........F........................EGKEIVVGD.GN.PD.....S..SLLLIGEAP...............
tr|G8TXV8  ........C......RLA..........D....RRTQVVVGR.GN.PQ.....A..TVMMVGEAP...............
tr|G8XT11  ......TIY......PAP..........E....HVHRWSHLN..E.PQ.....S.VRVIIVGQDP.........YHD...
tr|G9EIR0  ........C......PLH..........Q....TRKQTVFFR.GS.PQ.....A..KLMIIGEAP...............
tr|G9XY09  ........Y......PTK..........T....DIFRLFTLI.EP.ND.....I..KVVIIGQDP.........YH....
tr|H0E6E4  ........C......PQL.........AA....ARTTVVFGN.GD.PD.....A..DLLLVGEAP...............
tr|H0KUY0  ........C.....FPPK..........N....QIFRALELT..P.FE.....D.VEVVIIGQDP.........YHN...
tr|H0Q5N1  ......VVL......PAS..........I....DRYRALDLV.SP.AD.....V..RVCIAGQDP.........YHGIST
tr|H1D376  ........C......PLR.........KE....GGLGPVLST.GP.SD.....S..PLMIVGEGP...............
tr|H1P1U4  ........F......PEE.......................GP........V..RLILYGEAP...............
tr|H1P505  ........C......PLG..........P....GRIRFVFGE.GN.PK.....A..RMMFIGEGP...............
tr|H1XWX4  ........C......ELH..........K....TRKNFVFGY.GN.AQ.....A..EVMFVGEAP...............
tr|H2C8Z4  ........C......KLS..........S....TRTHAVPGE.GN.RE.....S..KIVFVGEAP...............
tr|H2J7L7  ........C......SLS..........L....SRNNVVVGE.GS.LD.....T..PIMFVGEGP...............
tr|H3KDP1  ......V.Y......PPT..........P......FRALELT..P.FE.....R.VRVVVLGQDP.........YHT...
tr|H3NN78  F.....DVY......PKR..........E....DIFKAFELT..D.YS.....D.VKIVILGQDP.........YH....
tr|H3NSX2  ......DIY......PPL..........E....SVFKCLQIL.SV.TD.....V..KVVILGQDP.........YHQ...
tr|H3RRL7  .................................HLTGFVAGQ.GP.KH.....P..KIMLIGEAP...............
tr|H3SCZ2  ........F......VPE...............PGERLVFGE.GP.EH.....A..RLFLLGEAP...............
tr|H4GIS2  ...............DLK................LEDFLEGA.GP.LH.....P..HLMIVGEAP...............
tr|H5SI33  ........C......PLH..........Q....QRTQAVPGE.GP.PD.....A..PLLFVGEGP...............
tr|H6RLZ5  ........C......PEL.........AA....ARRNVVVGD.IP.VA.....GRPLVALVGEAP...............
tr|H7F338  ........F........................HLEGFVKGS.GP.LK.....P..EVMLIGEAP...............
```

```
tr|H8I7F5  ........C......DLA..........L....SRTKVVVGS.GP.LD.....A..RIVLIGEAP...............
tr|H8ZDF2  ...............PAH...............ILRCLSFFE....YS.....D.VKVVILGQDP.........YHG...
tr|H9UI22  ........C......ALH..........A....ERNRTVPGI.GS.PE.....P..LVCVIGEGP...............
tr|H9ZZZ0  ........C......ELY..........L....SRTKPVVGE.GN.LN.....S..KFVLIGEAP...............
tr|I0GI60  ........C......DLH..........K....TKTHYVPGV.GP.AN.....A..KIMFVGEGP...............
tr|I0I6B7  ........C......RLA..........Q....GRTHAVPGE.GN.PN.....A..MVLFVGEAP...............
tr|I1A005  ....NIQIF......PHQ..........M....DMFRPFEFF.QT.NE.....T..KLIILGQDP.........YH....
tr|I1B0L2  ....PLPVY......PPA..........T....HRFRALDLC..P.PD.....A.VKVVILGQDP.........YHT...
tr|I2K0D9  ......TIF......PPK..........D....DIYSWSRLA..P.LS.....K.VRVLILGQDP.........YH....
tr|I3CUN9  ........C......GLC..........Q....GRKNTVFGV.GD.EK.....A..RWLFIGEGP...............
tr|I3VQA4  ........Y......PAP..........E....NVHRWSRLC.RP.GD.....V..KAVIVGQDP.........YHD...
tr|I4CBR6  ........C......RLH..........E....KRHTIVFGE.GK.PD.....A..RLMFVGEGP...............
tr|I7LIA9  .................................IEKKVVFGE.GA.KN.....A..KIMLIGEAP...............
tr|I7LKX1  ........C......PLW..........E....NTHHAVPGE.GD.AG.....A..RLMLIGEAP...............
tr|J0UVA7  ........C......GLH..........T....GRAQVVFGS.ELVAE.....P..DWMIVGEAP...............
tr|J5SPB4  ...............PVA........STE....DIYSWSRFC..P.LK.....D.VRIVIVGQDP.........YH....
tr|J7R1V5  .......VF......PTP..........G....DIYSWSRMT..P.FD.....A.VKVVIIGQDP.........YH....
tr|J9E0C3  ........C......ALK..........R....AAKNTVFSD.GN.PA.....A..KIMLVGEAP...............
tr|J9Z1B8  .....IPFY......PPG..........R....FLFSAFNLT..P.LD.....Q.VKVVILGQDP.........YH....
tr|K0IF13  ........C......KLS..........R....SRKNAVPGE.GQ.LS.....A..KIMFIGEAP...............
tr|K0KF39  ......TIF......PPE..........N....DIYSWSRLT..P.LN.....K.VKVIILGQDP.........YH....
tr|K1JXC9  ........C......SMA..........K....SRQHVVFAE.GG.PG.....M..RIALVGEAP...............
tr|K1JYF3  ......QVY......PPR..........V......FRAMDET..P.SN.....G.VRVVILGQDP.........YH....
tr|K1LNP2  ......TIY......PPS..........H....QVYQALTLT..P.FS.....Q.VRVVIIGQDP.........YH....
tr|K1ZRE1  ...............PLY..........NYRKKNNYFPVIGE.GNHFS........KIIFIGEAP...............
tr|K2A3B6  ........L......PLA..........S....GASRLVFGD.GN.PD.....A..QILMIGEGP...............
tr|K2B4F4  ........C......RLY..........E....GRKNVVPGD.GN.EN.....A..EIMFIGEGP...............
tr|K2C359  ........C......ELG..........S....TRKNAVPGE.GN.IK.....S..VLMFIGEGP...............
tr|K2CLQ5  ........C......KTG...............KNGKAVVGE.GS.AN.....A..EYIFVGEAP...............
tr|K2DEQ6  ........C......RLC..........K....YAKNAVPGE.GN.IN.....T..EIVFAGEAP...............
tr|K2E7J1  .......VY......PPA..........K....NIFRAFDLC..P.LD.....K.VKVVIVGQDP.........YH....
tr|K2E9B4  ........C......DLS..........K....SRTQVVFSD.GN.PE.....A..KLMIIGEAP...............
tr|K2ETW7  ........C......DLH..........K....TRNKVVFGI.GS.QS.....A..KIMFIGEAP...............
tr|K2F640  ........L......PLK..........K....GATKLVFGT.GN.TK.....A..KILCIGEGP...............
tr|K4KIT8  ......EIY......PPD..........A....LWFNAFLTT..P.LA.....D.VKVVIVGQDP.........YH....
tr|K6GHM9  ........SLGARIFPEN..........L....HIFRALELV..H.FE.....D.VKVMILGQDP.........YH....
tr|K7YPM8  ........C......NLK..........L....TANNTVFAD.GI.PG.....A..EVMIIGEAP...............
tr|K8ZAC1  ......SVF......PPK..........E....KIFQAFAYT..P.YD.....E.VKVVLLGQDP.........YH....
tr|K9VTI9  ........C......PLG..........E....TRTHAVVGR.GN.RH.....A..PVMVIGEAP...............
tr|L0A9A2  ........C......PLH..........L....SRTNVVLGN.GN.IN.....T..KIMLIGEAP...............
tr|L0B4R8  ......TIY......PAN..........P......FRSLYNI.KN.LS.....E.INVVILGQDP.........YH....
tr|L0B866  ......IIY......PFN..........P......MKSLHVI.SS.IS.....D.IRVIILGQDP.........YH....
tr|L0B8A6  ........C......FLS..........G....HRKNVVFGD.GVLSD.....V..DCMIIGEAP...............
tr|L0DFI2  ........C......PHL.........AA....SRTRTVFGS.GS.PT.....A..RLMFIGEAP...............
tr|L0L178  ........C......PLH..........E....NATNKVIIR.GS.ER.....P..KVLFIGEAP...............
tr|L2GQC8  ......IFY......PPI..........E....KIFNFTHFS.SF.KD.....I..KVVIMGQDP.........YHN...
tr|L2GX11  ........Y......PPV..........H....KIFYFSHFF..PIAN.....T..RVVIIGQDP.........YHN...
tr|L5N8A0  ........C........................LVEGFVPGK.GN.RN.....A..EIMLVGEAP...............
tr|L8HA23  ......TIY......PPP..........E....HVFSQLNLC..P.FD.....E.VKVVILGQDP.........YH....
tr|L9X1W2  ........Y.....DPER...............FV....GGV.GP.LS.....A..DAMLVGEAP...............
tr|M0BKB2  ........C......PQL.........AA....CRERISWGT.GP.LD.....A..SVFVVGEAPGAGNRDADPWRG...
tr|M0CBA6  ........F.....VPAA......................GP.LD.....A..EIVFVGEAP...............
tr|M0MGS7  ........C......PAL.........AD....AREHISWGN.GS.LD.....A..ELVVVGEAP...............
tr|M1E4Q4  ........C......KLS..........E....TRINVVFGE.GP.MN.....P..DIMVIGEGP...............
tr|M1LNF1  ......IIY......PSN..........P......FRAIHIT.NT.IS.....K.INIVIIGQDP.........YHN...
tr|M1QKH3  ........C......KLS..........E....QRTQVVFGD.GN.PD.....T..GIMFVGEAP...............
tr|M1VCA8  .......IF......PPQ..........N....LVFHAFQRC..P.LE.....K.VKVVIIGQDP.........YHN...
tr|M1Z9H8  ........Y......PEE..................IIIKGY.GN.LN.....S..VIMLLGEAP...............
tr|M2XVU5  .....KVIF......PPE..........P....LVFNAFKKC..P.FN.....K.VRVVIVGQDP.........YH....
tr|M3EFG6  ........Y......PVE..................GFVWGS.GP.EN.....P..AIMLVGEAP...............
tr|M4YU22  ........C......DLC..........R....WRTNLVLPS.GD.LD.....S..PVVLVGEAP...............
tr|M5ADY8  ........F......PKQ..........K....NVFLCLKYC.SF.QR.....L..KVVILGQDP.........YH....
tr|M5IVB6  ......SIY......PAA..........P......FRALELL..P.LQ.....K.VSVVILGQDP.........YH....
tr|M6D8H9  ........C......KLS..........K....TRTQTVFGE.GN.PN.....A..EVMFIGEGP...............
tr|M7WR77  REGGKLKVF......PPA..........R....DVYAWSRYT..P.LQ.....D.VKVVILGQDP.........YHD...
tr|N0BBN4  ........C......KLW..........L....TKQNYVPGE.GN.CK.....A..LIVFVGEAP...............
tr|N1V4N6  ...............PNNAIR.....RRN....LELYLLEML.AR.RP.....T...VLLVGEAP...............
tr|N1Z687  ........Y........................AEKKIVFGE.GK.QN.....A..KICMVGEAP...............
tr|N2BLL9  AVFPQNAIF......PPN..........E....LIFNAFNLT..P.PE.....S.VRIVILGQDP.........YHGSIF
tr|N9SHT1  .....YQVF......PKK..........G....DLFNAIKIT..D.FD.....N.LKIVIIGQDP.........YHQ...
tr|N9V498  .....SVIW......PKK..........S....DWFRALTFF.EP.NE.....T..RLIIIGQDP.........YH....
tr|Q025I0  ........C......KLG..........D....ARNKIVFGV.GD.EK.....A..QLVFVGEGP...............
tr|Q0C0Y4  ...............PLR..........A....SCENTVVYD.GT.PG.....A..SLLVIGEGP...............
tr|Q0EZR7  ........C......SLA..........D....TRTQVVFGV.GN.PQ.....A..DLVFIGEAP...............
tr|Q12TN4  ........C......PLS..........E....TVINKVVRK.GS.DE.....P..KVLFIGEAP...............
tr|Q1AV32  ............................R....FGEDSVFGE.GE.PG.....A..RLAIVGEAP...............
tr|Q1AYQ1  ........C......DLS..........R....SRTNTVFGT.GD.PY.....S..PLMLVGEGP...............
tr|Q1GY28  ...............PRR..........G....LWFEALRQVEPA.AA........RVVLLGQDP.........YHG...
tr|Q1N1I3  ......IVY......PPK..........E....LWFNAFQLT..P.FE.....D.IKAVIVGQDP.........YH....
tr|Q23W06  ........Y......PPA..........H....EIFNVFKQT..P.LN.....T.LKVVIVGQDP.........YHQ...
tr|Q3SA81  ........C......GLW..........K....TRTHAVPGE.GG.FR.....K..KVMFVGEAP...............
tr|Q53CZ3  ......VIY......PPI..........D....RIMWWSYCC.EP.ED.....I..KVVILGQDP.........YHR...
tr|Q5DC15  ......TIY......PPI..........E....QIFTWTKLC.LP.TD.....I..RVVILGQDP.........YH....
tr|Q5WES9  ........F........................LL.....GG.GN.EE.....A..DVLFVGEAP...............
tr|Q6CAC0  ......NIF......PPK..........K....DIYSWTSLT..P.FD.....K.VKVIIIGQDP.........YH....
tr|Q6F1M3  ......KIY......PSK..........E....DCLRLFDLI.SP.DE.....I..KVVIIGQDP.........YH....
tr|Q6KIL8  ......IIC......PNL..........E....DIFRSFSFF..D.FQ.....D.LRIVILGQDP.........YH....
tr|Q7VBS6  ........C......GLD..........K....DTCRIVVSR.GD.PT.....S..NLMLIGEAP...............
tr|Q8TK31  ........C......PLH..........K....SATKRVIGK.GS.CN.....P..KVMFIGEAP...............
tr|Q9RTK9  ........C......PLR..........V....SASQVVVSD.GD.PR.....A..PLLIVGEGP...............
tr|Q9WR44  ......EIF......PIK..........K....DIFAWTRFC..P.PE.....K.VRVVILGQDP.........YH....
tr|R1ATM3  .................................KDKEIVLGD.GT.VD.....S..SIILVGEAP...............
tr|R1E4B2  ........C......PLY..........K....FKTNYVPGD.GN.PN.....S..KILFIGEAP...............
tr|R1FRL1  ......TVF......PPA..........P....EVFSAFNLT..PLPE.....V..RVVVLGQDP.........YH....
tr|R1GL12  ........C......SLS..........K....SCLNTVFSD.GN.PE.....A..SVMIVGEAP...............
tr|R2SFV7  ........F........................LLEGFVPGA.GP.KD.....A..RLVILGEAP...............
tr|R2SXC9  .................................NLEGFLPGK.GP.KN.....A..VLMLVGEAP...............
tr|R4KRR4  ........C......PGE..........R....TGAGLVRDR.GN.LG.....A..RIVFLGEAP...............
tr|R4VE87  ........C......ELH..........Q....GRTQAVPGS.GD.RQ.....A..RLLIVGEAP...............
tr|R5ARL3  ...............PNG...............EYAAPVFGA.GA.VD.....S..PVMLVGEAP...............
tr|R5B4U8  ........Y..........................LNPVFGE.GP.LT.....P..HLMLIGEAP...............
tr|R5DX78  ........C......ALG..........A....TRNNIVFSD.GN.PS.....T.AKIVLIGEAP...............
tr|R5MPG0  ......TIF......PLE..........Q....NIFNAFRCT..P.FS.....N.VKVVILGQDP.........YH....
```

```
tr|R5PZ23  ........C......PLK..........N....SATNMVFGT.GN.PN.....A..DVMIIGEAP...............
tr|R5QX00  ........C......ALK..........L....TASHTVFGD.GN.PQ.....A..RVVFIGEAP...............
tr|R5Z1D7  ......PIY......PKA..........E....NIFHAFQMT.SL.KD.....T..KVVIVGQDP.........YH....
tr|R6HGV3  ......TIY......PAQ..........D....KILNALVFT.AP.ED.....V..RVVILGQDP.........YH....
tr|R6HL97  ......TVY......PAK..........D....NLFAAFKYI..N.YD.....D.VKVVILGQDP.........YH....
tr|R6PFR8  ........C......ELA..........K....TRTSVVFSD.GS.AD.....A..KIMLIGEAP...............
tr|R6PI86  ........C......PLG..........Q....TRTKSVFSS.GA.IN.....N..KLMLIGEAP...............
tr|R6R865  .......VY......PPL..........N....LIYNAFKLT..P.FN.....D.VKVVIMGQDP.........YH....
tr|R7BZ83  ......VIY......PPY..........P......FKALEMT..P.AE.....K.VKVVILGQDP.........YH....
tr|R7DQ64  ......TVY......PPK..........N....LLLNAFNLT..P.YS.....K.VKVVIIGQDP.........YH....
tr|R7DXQ1  ........W.....QPAR...ELNSLRDT....MVF.....AT.GN.PH.....T..ELMLIGEAP...............
tr|R7I8P1  ........C......GLC..........Q....NIHHKVPGQ.GD.RH.....S..PLMLIGEGP...............
tr|R7IAS2  ........C......GLS..........E....TRTRTVFAD.GA.PG.....C..PIVMVGEAP...............
tr|R7IHQ8  ...............PL...............VL.....GE.GC.SE.....R.PPVMLIGEAP...............
tr|R9ADN8  .....YTIY......PPK..........E....LIYNWSRSP....ID.....D.VRCCIIGQDP.........YH....
tr|R9LKN0  ......TIY......PPK..........S....QIFSALELT..P.PS.....H.VKVVILGQDP.........YH....
tr|S0EV15  ........C......DLA..........A....TRTQVVFGD.GN.PE.....A..PLMLVGEAP...............
tr|S0FG57  ........F.......................ENEQIVLGD.GC.LT.....G..PMIIIGEAP...............
tr|S2J4F7  ......TIF......PPL..........N....QIYSWSNYT..P.PS.....K.VKVVILGQDP.........YH....
tr|S3JT40  ........C......VLG..........Q....TRTHPVAGE.GP.EELSRLTNSVEVMVIGEGP...............
tr|S4NBY8  ...............P................RLEGFVPSE.GG.EN.....P..RILLLGEAP...............
tr|S5MFT9  .......IF......PEP..........Q....EIFSVFKLI.KP.SE.....I..KVIILGQDP.........YH....
tr|S5RQ34  ........L......PES..........Q....YIFRAFNTT....PF.....DYVKVIILGQDP.........YHT...
tr|S6G4W1  ........C......EIK..........K....AAVNTVFSD.GN.PK.....S..KIMLIGEAP...............
tr|S9VE50  ......TVL......PPR..........P....CIFTAFNRT..P.FD.....K.VKVVLLGQDP.........YH....
tr|T0AYQ1  ......TIF......PPK..........H....LIYSWSNYC..S.FD.....Q.IKIVIIGQDP.........YHN...
tr|T0DRC9  .......IF......PKS..........S....NLFYALNLT..P.PS.....A.VKIILLGQDP.........YHSTYL
tr|T0LSQ5  ........C......ALF..........E....YRKNAVCGN.GS.LS.....P..EIFLIGEAP...............
tr|T0MGQ8  ........F......PET..........K....NVFAFLNYF..PIES.....T..KIVIIGQDP.........YH....
```

```
                    70            80                                  90          100
sp|P12295   ....G...PGQA.......HGLAFSV.RP............G....I...A.I.PPSLLNMYKE.LE.NT..IPG.....
tr|Q8KLI8   ....G..EEEDK.......TGRPFVG.KA............G...........QLLNRILEA.AG.IP.........
sp|A1QYK3   ....G...KGQA.......NGLAFSV.NS............D....I...K.I.PPSLQNIFKE.IE.RS..L.......
sp|A1SUE5   ...EG....Q.S.......HGLSFSV.PE............G....I...K.I.PPSLRNIYKE.LS.TS..IEG.....
sp|B0B8I2   ....G...EGQA.......HGLSFSV.PR............G....Q...A.L.PPSLRNIFQE.LH.TD..LGI.....
sp|B1AJI8   .......TPKIA.......NGLCFSV.DL............G....N...N.L.PGSLINIFKA.LE.YD..L.......
sp|P12888   ....G....GQA.......NGLAFSV.AY............G....F...P.V.PPSLRNIYAE.LH.RS..LPE.....
sp|Q057V4   ...AG....Q.A.......HGLSFSV.PK............G....V...F.L.PPSLKNIFIE.LK.NN..F.......
sp|Q315T1   ....G...AGQA.......HGLAFSV.PQ............G....V...K.P.PPSLRNVLKE.AA.AQ..KPE.....
tr|A0LFG4   ....G..LEEDR.......QGRPFVG.RA............G...........RLLDQMIKA.LG.FH.........
tr|A0LH32   ....G..RDEER.......EGKPFVG.AA............G...........RNLNALLLE.NG.LA.........
tr|A0LRS0   ....G..AEEDR.......TGLPFVG.RA............G...........RLLDELLVD.AG.LP.........
tr|A0RV64   ....G..REEDL.......QGKPFVG.GA............G...........RVLDKELER.AG.IP.........
tr|A1BDC5   ....G..ADEDS.......QGRPFVG.RS............G...........QLLDKILQA.VQ.FK.........
tr|A1RYC0   ....G..RNEDL.......QGRPFVG.AA............G...........ALLTELISL.AG.LS.........
tr|A1US05   ....G..REEDI.......QGIPFVG.KA............G...........ILLNKILAS.IG.LT.........
tr|A4GHT2   ....G...PGQA.......NGLAFSV.NK............E....V...S.I.PPSLNNIFKE.VS.DD..L......N
tr|A4HXS3   .......DLHQA.......HGLCFSV.LP............E....V...A.L.PPSLRNIYKE.LT.TD..IAG.....
tr|A5KS51   ....G..KKEDE.......QGLPFVG.AA............G...........RFLDEMLAS.AN.LK.........
tr|A5UQ47   ....G.RHGADQ.......TGIPFWGDRS............G...........RILRRMLVR.MG.LA.........
tr|A6DBB2   ....G..ENEVK.......QKSPFVG.KA............G...........ENLNYLISL.SG.LN.........
tr|A6DK00   ....G...PNQA.......MGLSFSV.PR............G....E...K.I.PPSLRNIYKE.LD.SD..LGI.....
tr|A6GN76   ....G..VDEDA.......TGRPFVG.KS............G...........QLLDRMLKA.VG.AS.........
tr|A7ANQ0   .......QPKQA.......MGLSFSV.PR............G....V...A.I.PPSLRNIYKE.IG..........
tr|A8A982   ....G..KEEDE.......EGRPFVG.RA............G...........RLLRSTISQ.IG.FK.........
tr|A8E1M5   ....G.....SA.......CGLAFGT.VR............D....R...P.A.PPSLVTVFKE.LR.RS..IPE.....
tr|A8F067   ....G..STEDL.......KGIPFCG.ES............G...........NLLDNMLHS.IG.IS.........
tr|A8MA01   ....G..RVEDE.......TGRPFVG.AA............G...........KLLDELLHE.IG.VD.........
tr|A8SNH5   ....G...VNQA.......NGMSFSV.NR............G....E...K.I.PPSLRNIYLE.LY.SD..LGI.....
tr|A8UAT5   ....G..ETEAL.......NGIPFSG.RA............G...........KELMRFFDL.LE.VD.........
tr|A8UU74   ....G..EDEDR.......QGRPFVG.RA............G...........KYLNKKIEEVLG.LR.........
tr|A9AZ06   ....G..QREDA.......LGRPFVG.PS............G...........DLLEQWLAE.IG.LT.........
tr|A9BHU0   ....G..ADEDA.......LGEPFVG.RA............G...........QLLEKMLKEYAK.LE.........
tr|A9F5H8   ....G..AEEDL.......QGQPFVG.AA............G...........QLLDKMIAA.MG.YH.........
tr|A9NHD4   ....G...PNQA.......HGLSFST.LN............E.......K.L.PPSLKNIYKE.LK.DD..L.......
tr|B0VFE1   ....G..EQEDI.......TGRPFVG.AA............G...........QLLEKMLSA.IN.LQ.........
tr|B1GA08   ....EDRGTPQA.......HGLAFSV.AP............N....V...R.T.PPSLRNIFKE.IA.TS..LGHE....
tr|B1H0K1   ....G..YDEDH.......IGKPFVG.RA............G...........QLLTKIIEA.MG.QA.........
tr|B1I4W5   ....G..TDEDA.......IGHPFVG.QS............G...........LLLTAALNS.LG.VD.........
tr|B1VA82   ....G...ENQA.......HGLSFSV..K............G....Q...K.R.PPTLNNIFKE.LK.ND..L.......
tr|B1Y7I1   ....G...AGEA.......EGLAFSV.PE............G....V...R.I.PPSLRNIHQE.LM.TD..VGI.....
tr|B1ZZ66   ....G..AEEEI.......QGEPFVG.PA............G...........QLLTKMIGA.MG.LR.........
tr|B2A668   ....G..AKEDE.......LQRPFVG.PA............G...........ELLTKMLAA.IN.FS.........
tr|B2KCZ8   ....G..FDEDH.......QGRPFIG.RA............G...........TLLTKMIEA.MG.LK.........
tr|B3DV47   ....G..AEEDL.......QGEPFVG.PA............G...........QLLTKMIRA.MG.LS.........
tr|B3U4N1   ...PG....M.A.......HGLCFSV.RP............Y....V..AP.L.PRSLGNIYRE.LR.DD..V.G.....
tr|B3U4U7   ....G..FHEDQ.......KGEPFVG.AA............G...........QLLNDLLES.AG.LS.........
tr|B5II11   ....G..AQEDA.......SGQPFVG.RA............G...........QLLDQMLAS.VGIDS.........
tr|B5LRM7   ...PG.....QA.......HGLAFSV.RR............G....I...Q.I.PPSLRNILSA.VR.RS..YPE.....
tr|B5Y8I5   ....G..SNEDE.......QGLPFVG.QA............G...........AILDTFLEK.NG.FK.........
tr|B5YEZ9   ....G..FHEDQ.......QGRPFVG.AA............G...........RLLTELIES.MG.LK.........
tr|B6A3R0   ....G..DQEDL.......AGRPFVG.PA............G...........RLLDRCLEE.AG.LD.........
tr|B6AFI4   ...PG.....QA.......MGLCFSV.PK............G........VTVPPSLRNIYKE.IGCTD.........
tr|B6K2R2   ...VG....Q.A.......HGLSFSV.RA............G....I...P.C.PPSLKNIYRT.IK.ND..YPD.....
tr|B6KW10   .......QPGQA.......MGLAFSV.PR............G.......IPLPPSLQNIFKE.IA.RN.........
tr|B7FQS1   ....G...PGQA.......HGLCFSV.LP............G....Q...A.I.PPSLKNVYRE.LN.ED..S.Q.....
tr|B7KM64   ....G..QTEDK.......TGKPFTG.KA............G...........KLLDKMLAS.IN.LE.........
tr|B8C427   ...PG....Q.G.......HGLAFSV.RK............G....V...S.I.PPSLRNIFKE.AI.DD..V.G.....
tr|B8CX81   ....G..ADEDR.......LGKPFVG.KA............G...........KLLTKILNS.VG.LE.........
tr|B8EJL3   ....G..ADEDR.......QGVPFVG.RA............G...........QLLNRMLAA.IG.LD.........
tr|B8HFW5   ....G..FRGMR.....ITGVPFTN.RTIFEGPANSFGLFG.....PGKGYVLPPEAAGVAAEPTATVM.........
tr|B8I5K9   ....G..KDEVK.......LGKPFVG.AA............G...........KNLNEFIEV.LE.IS.........
tr|B9KXL9   ....G..YHEDR.......QGRPFVG.AA............G...........QFLNELLGR.AG.LR.........
tr|B9XNJ8   ....G..RNEDL.......TGRPFVG.SS............G...........KFLDHVLEG.SE.FD.........
tr|B9Z7Q7   ....G...AGEA.......MGLSFSV.PP............G....V...R.I.PPSLRNVYKE.IA.AD..LGT.....
tr|C0BNZ8   ....G...THQA.......HGLSFSV.PI............G....I..AH...PPSLKNIFIE.LS.ND.........
tr|C0X269   ....G..KEEIK.......ENVPFIG.RS............G...........QLLMEQLQQ.VG.LS.........
tr|C1A7L2   ....G.PRGADK.......SGYPFFGDGA............G...........QHLYTALGR.LGAMS.........
tr|C1D9B0   ....G...AGEA.......CGLAFAV.RR............G....I...R.I.PPSLRNIYRE.LE.DS..LDI.....
tr|C1F1L1   ....G..ADEDA.......QGLPFVG.RA............G...........QLLNNMIGA.MG.LE.........
tr|C1G9A4   .......NHNQA.......HGLCFSV.RP............P....M...P.A.PPSLKNIYTA.LK.ND..YPS.....
tr|C2MIJ3   ....G..ETEIH.......NGIPFSG.RA............G...........KQLMGFLER.IH.VT.........
tr|C3X2T7   ....G..FHEDR.......QGEPFVG.RS............G...........KLLDAMMAA.MR.MK.........
tr|C5CIT9   ....G..ADEDA.......TGRPFVG.RA............G...........KLLTKILES.VG.LS.........
tr|C5NVK4   ....P....EQA.......CGLSFSV.ND............G....V...P.L.PKSLINIYKE.LH.DD..LGI.....
tr|C7H0A3   ...EG....E.A.......DGLAFSV.GR............N....V..KR...PPSLMNIIKE.VT.LE..EAD.....
tr|C7M312   ....G..AREDE.......LGRPFVG.RS............G...........QLLDALLADETG.LD.........
tr|C8W879   ....G..KNEDL.......SGQPFVG.TS............G...........KKLDALLEE.VG.LS.........
tr|C9M5Q7   ....G..AAEDE.......SGIPFVG.AS............G...........RLLSQLLEE.SG.LR.........
tr|C9RLM9   ...PG.....QA.......HGLCFSV.PF............G....I...E.P.PPSLINIFQE.LH.DD..LGI.....
tr|D0MK10   ....G..AEEDR.......QGEPFVG.PA............G...........QLLNKMLKA.IG.FE.........
tr|D0PPD4   ....G....GQA.......TGLAFSV.HE............N....F...P.V.PPSLHNIFQE.LK.RS..VPD.....
tr|D0U4F0   .......NEGQA.......HGLSFSV.PD............G....I...R.V.PPSLRNIYQE.LS.SD..L.S.....
tr|D1ART8   ....G...PHQA.......HGLSFSI.ES............E...KA...K.F.PPSLRNMFKE.LK.TD..L.......
tr|D1C3J5   ....G..GCEER.......LGHPFVG.PA............G...........QVLTEALAQ.AG.ID.........
tr|D1CDM9   ....G..EDEDR.......TGTPFVG.RA............G...........ILLDQLLEE.AG.IG.........
tr|D1JDZ5   ....G..YNEDL.......KGKPFVG.KA............G...........KVLDELLES.IG.WQ.........
tr|D1PN05   ...PG.....QA.......MGLSFSV.PD.....................ACKTPPSLKNIFKE.LE.SE.........
tr|D1Y5H9   ....G..AEEER.......TGRPFVG.RS............G...........RLLTEILAA.AE.LK.........
tr|D2EF83   ....G..KNEDL.......QGRPFIG.MS............G...........KFLTKNLES.LN.IK.........
tr|D2Z573   ....G..AEEDS.......TGRPFVG.RS............G...........RFLSDLMAE.AG.LR.........
tr|D3B454   ....G...PGQA.......HGLAFSV.KK............G....V...K.P.PPSLVNIYKE.LT.TD..IEG.....
tr|D3E6H2   ....G..AHEVL.......EGKPFVG.QA............G...........QEMNAYLER.LK.LT.........
tr|D3FBP7   ....G..ANEDR.......GGLPFIG.QA............G...........RLLDKLLQE.VG.LE.........
tr|D3QZ35   ...PG....Q.A.......IGLSFAV.RR............G....V...K.I.PPSLQNIFRE...........QG...
tr|D4G8B4   .......CPNQS.......HGLCFSV.PP............K....I...E.I.PPSLRNIYKE.LE.EN..VKS.....
tr|D5C4I1   ....G..VQEAK.......TGRPFVG.NA............G...........KLLNTLLNQ.IG.LE.........
tr|D5E730   ....G..KKEDA.......SGIPFCG.RA............G...........KILDGMIED.MD.LQ.........
tr|D5EGK6   ....G..ATEDE.......TGRPFVG.RS............G...........NLLCQLIEE.AG.LS.........
tr|D5EHP1   ....G..ADEEI.......AGQPFVG.KA............G...........QLLTKIISA.MG.LS.........
tr|D5MIW4   ....G..ADEDE.......QGEPFVG.RA............G...........QLLTRIIEA.MK.LT.........
tr|D5RKP0   ...PG.....QA.......MGLSFSV.PV............G....V...R.I.PPSLRNIHRE.LA.AD..CGLP....
tr|D5U040   ....G..RKEDE.......VGSPFVG.PA............G...........RLLNTLLEN.SG.LK.........
```

```
tr|D5U570  ....G..ADEDK.......QGLPFVG.RG............G.............KLLDKWIEK.MN.IN..........
tr|D5V6V9  ....G...AGQA.......QGLSFST.PK............E....I...K.N.PPSMVNILKE.IK.DD..LKR.....
tr|D5WWR6  ....G..ADEDA.......QGFPFVG.RA............G.............QLLDRMLKE.AG.FD..........
tr|D7CKV6  ....G..QVEDE.......QGRPFVG.PA............G.............QLLDRILEA.CA.IR..........
tr|D7CWJ9  ....G..EDEDL.......LGRPFVG.RA............G.............QLLDRILES.AH.IE..........
tr|D7UXZ6  ....G..ETEIH.......TGVPFTG.RA............G.............KELEKMMTR.AN.VQ..........
tr|D8LFW4  ...PG.....QA.......HGLAFSV.MK............G....V...M.Q.PPSLRNMVKE.AV.SC..CGI.....
tr|D8UI66  ....G...PGQA.......MGLAFSV.PR............D....V...R.PLPPSLVNIYKE.AA.AD..LGWGD...
tr|D9Q1V3  ....G..KNEDE.......QGRPFVG.AA............G.............QLLSSLLEQ.AG.LR..........
tr|D9QQQ4  ....G..RQEDE.......QGEPFVG.KA............G.............QLFNKILKA.AE.IK..........
tr|D9S3H2  ....G..FEEDK.......QGKPFVG.RA............G.............QLLTAILNK.LN.ID..........
tr|D9SCW5  ....G..AEEDE.......LCEPFVG.AS............G.............RLLDNMLLA.IG.LQ..........
tr|D9Y2E5  ....G...FNQA.......NGLAFSV.NK............D....V...P.P.PPSLRNIFKE.IK.RE..YPE.....
tr|E0S9I8  ....D....QQA.......MGLSFSV.PL............G....V...S.I.PPSLRNIYLE.AS.TD..IEG.....
tr|E0SQC9  ....G..ATEDE.......MGRPFVG.AA............G.............KLLTMIMEN.LG.IS..........
tr|E0TBI7  ....G..RDEDR.......IGKPFVG.KA............G.............GLLDLMLHA.IGYSRTEADG.....
tr|E0TIS2  ....G..KMEDL.......KGYPFIG.KS............G.............KLLNYLINK.LK.YK..........
tr|E1IBI3  ....G.YNGARR.......TGVPFTS.ERILLEGSQYGVAQGFAIATNDGRISAEPTATIVYRE.MAALN..........
tr|E1L0U9  ....G...PHQA.......NGMAFSV.ND............G....V...K.F.PPSLRNIFVE.LG.ND..LGV.....
tr|E1QFX8  ....G..AQEDQ.......QGLPFVG.PA............G.............RLLDNMLAA.VG.LR..........
tr|E1QNN8  ....G..EREDE.......EGRPFVG.AA............G.............QLLTKLLNN.VG.IR..........
tr|E1QWE2  ...PG....Q.A.......MGLAFSV.RA............G....T...T.Y.PPSLRNILKE.LR.AD..LGH.....
tr|E1R614  ....G..AQEDA.......SGRPFVG.PA............G.............QYLDKWMKA.IG.LS..........
tr|E2IUG8  ...PG.....QA.......HGLAFSV.RP............G....V...P.V.PPSLRNIFTA.VR.SC..YPE....A
tr|E2SYG7  ....G.RLGADG.......SHLPFHGDKS............G.............HNFEKLIEQ.VG.IS..........
tr|E3D056  ....G..ADEDE.......QGMPFVG.RA............G.............QLLTQILQA.AG.IR..........
tr|E3IP47  ....G...EGQA.......HGLAFSV.PE............G....V...P.A.PRSLRNVFRE.IE.TD..LGH.....
tr|E4KQ36  ....G...PNQA.......NGLAFSV.NR............G....K...A.L.PPSLRNIYQE.LQ.QD..LGI.....
tr|E4RKG8  ....G..ATEDR.......MARPFVG.RA............G.............KLMDKILQS.VN.LK..........
tr|E6N689  ....G..EEEDL.......QGRPFVG.RS............G.............RLLTEALEK.AG.IR..........
tr|E6PFU2  ....G..ETEDR.......LGRPFVG.RA............G.............ELLEKMLGA.IG.LA..........
tr|E6W1H1  ....G...PGQA.......NGLAFSV.SS............G....V...A.L.PPSLRNIYKE.IQ.AD..TGS.....
tr|E6W552  ....G..FDEDR.......LGRPFVG.KS............G.............QKLDQIMLA.VG.FQ..........
tr|E7A9L4  YKNQE...HPLA.......MGLSFSV.PT............H....A...P.I.PKSLQNIYKE.LQ.QS..LH......
tr|E7FX98  ...PG....Q.A.......MGMSFSV.FK............D....Q...K.L.PKSLVNIYKE.LE.SD..LGI.....
tr|E7H418  ....G..AEEDL.......QGIPFVG.KS............G.............KLLTAMLDS.LGLVR..........
tr|E7H4Y1  ...PG.....KA.......CGLAFSV.PA............G....E...K.L.PPSLKNIFKV.MA.AS..NEI.....
tr|E8LLT1  ...PG.....QA.......MGLSFSV.PV............G....V...P.V.PPSLRNIYRE.LK.DD..MPG.....
tr|E8N338  ....G..FYENE.......QGRPFVG.AA............G.............KFLDELLEK.CG.IA..........
tr|E8T398  ....G..EQEDL.......QGRPFVG.RA............G.............QLLSKFLNL.YG.VT..........
tr|F0EKF3  ....G..RTEIE.......NFIPFSG.QA............G.............KELMKSLAL.AG.LT..........
tr|F0NSC6  ....G..RNEIV.......NNIPFSG.DA............G.............KELDKSLKQ.IG.LS..........
tr|F0RSJ4  ....G..FEEDA.......SGKPFVG.KA............G.............KYLDSWLGA.IH.MD..........
tr|F0SG07  ....G..ADEDK.......QGEPFVG.RA............G.............KLLNRIIEA.CG.WE..........
tr|F0Y985  ....G...PGQA.......NGLAFSV.SG............G....C...A.I.PPSLRNVLRE.CA.ED..H.G.....
tr|F2LXL6  ....G..RDEDV.......QGRPFVG.RA............G.............RLLRDILRD.IG.IK..........
tr|F1A0X7  ....G...KGQA.......HGLSFSV.KP............G....V...S.P.PPSLINIYKE.LV.QD..CPP.....
tr|F2NFM2  ....G..EAEDR.......QGEPFVG.RA............G.............QLLNRLLSK.LE.IS..........
tr|F3KRD3  ....G...AGQA.......EGLAFSV.AP............G....V...K.P.PPSLRNIHKE.LA.RD..PQL.....
tr|F3QMU0  ....G..AEEDR.......LGQPFVG.PS............G.............KLLDKILEA.AK.LD..........
tr|F4A8X0  ....G..KNEVE.......QGKPFVG.MA............G.............ETFNNYLKS.IG.LS..........
tr|F4C0J7  ....G..REEDL.......KGLPFVG.RA............G.............RLLDEALNE.AG.LK..........
tr|F4GMI7  ....G..SVDDR.......TGLPFQG.KA............G.............QLLQAMLAS.IQVTE..........
tr|F4GSY3  ....G...PNQA.......QGLAFSV.PD............F....C...T.V.PPSLRNMFAE.LA.KE..YPD.....
tr|F4HNK0  ....G..YWEDQ.......MGLPFVG.KA............G.............KVLDELLKS.IG.LS..........
tr|F4NZD5  ....G...LHQA.......HGQCFSV.QK............G....V...S.I.PPSLINIYKE.LE.SD..LGL...DV
tr|F4QWL4  ....G..AEEDV.......RGQPFVG.QA............G.............QLLDRILTA.AGLLD..........
tr|F5SL17  ....G..ADEDR.......QGRPFVG.RA............G.............QLLSRMLEA.AE.IP..........
tr|F5Y215  ....G..ENEDL.......QGEPFVG.QA............G.............KLLDNMLRA.IGLSR..........
tr|F5YGI3  ....G..ADEDA.......SGRPFVG.KA............G.............QLLDKMLGS.VG.LS..........
tr|F7NHV1  ....G..ETEDQ.......FGVPLIG.NS............G.............KLLDKALWS.VG.IT..........
tr|F7PTA1  .......QPNQA.......NGLSFSV.SK............E....V...D.I.PKSLKNIYKE.LE.ND..........
tr|F7S094  .......QPNQA.......HGLAFSV.PK............G....V...K.P.PPSLKNIYKA.II.ND..YPS.....
tr|F7XVS9  ....G..ANEDE.......QGIPFCG.QS............G.............KLLDNIFKT.IGLSR..........
tr|F8ADN2  ....G..KDEDL.......EGRPFVG.RA............G.............QLLDQMLNT.VG.IN..........
tr|F8L559  ....G...AHQA.......HGLCFSV.QH............G....I...N.P.PPSLKNIYRE.MQ.DD..LGI.....
tr|F9CXI5  ....G..KNEDQ.......RGEPFVG.IA............G.............QRLNSALEN.AG.IS..........
tr|F9DTW2  ....G..EHEAV.......HGVPFIG.RA............G.............DELMKSLAS.VG.LS..........
tr|F9LK36  ....G..VNEIK.......TNVPFSG.KS............G.............LILNEKLND.VG.LK..........
tr|F9ZLD4  ....G..ADEDV.......QGRGFVG.RA............G.............RTLHRLLES.FG.LV..........
tr|G0LJW8  ....G..ANEDE.......YGEPFVG.RS............G.............DVLDDALNE.AG.VS..........
tr|G0QBL8  ....G..ENEVE.......EREPFVG.RA............G.............KVLDSTLSD.IG.VE..........
tr|G0QFK7  ....G..ANEVE.......QGEPFVG.RA............G.............KKMNKILSE.IG.VE..........
tr|G0U4E4  ...IG....SGT.......WGSAFLS.WA............G....V...P.L.PPSLKNMYKE.LA.TD..IPG.....
tr|G2RZ19  .......DFNQA.......NGIAFSV.NQ............T....V...S.A.PPSLKNIFKE.LK.AD..L.......
tr|G4RL69  ....G..RREDE.......EGRPFVG.AA............G.............RLLTEILSK.NG.IE..........
tr|G4STH7  ....G..ESEDR.......QDEPFVG.RA............G.............QLLTEMLRA.IG.LK..........
tr|G4T6H9  ....G...PNQA.......HGLAFSV.LP............P....T...R.P.PPSLVNIYKE.LK.NS..IPG.....
tr|G7WNS9  ....G..REEDR.......TGRPFVG.RA............G.............SVLDRCLEE.AG.IE..........
tr|G8BJ42  .......NHNQA.......HGLAFSV.LE............P....T...R.P.PPSLVNIYKT.LS.ID..YPD...T
tr|G8LWR8  ....G..KDEVA.......QKRPFVG.MA............G.............KNLAQFLEI.LG.FS..........
tr|G8TXV8  ....G..RDEDR.......TGYGFQG.AA............G.............KIFDQVLEY.LG.LS..........
tr|G8XT11  ....G.....SA.......SGVAFGT.FP............G....R...Q.T.PASLINVFRE.LS.RT..IMN.....
tr|G9EIR0  ....G..FYEDQ.......QGLPFVG.KA............G.............GLLNQMLQS.VELFE..........
tr|G9XY09  ....G...PGQA.......NGLAFSV.ND............G....I...K.N.PPSLVNIFHE.LK.KD..L.......
tr|H0E6E4  ....G..QDEDR.......QGVPFVG.AA............G.............RLLNELLAE.IG.IR..........
tr|H0KUY0  ...DG....Q.A.......NGLCFSV.SE............S....V...T.A.PPSLKNIFTE.LK.DD..L.......
tr|H0Q5N1  VADGR..QVAEA.......MGLSFSV.PQ............G....V...R.I.PPSLRNIYKE.LA.AD..LGG.....
tr|H1D376  ....G..GVEDD.......YGGPLVG.PS............G.............QLLDKALLS.VG.IT..........
tr|H1P1U4  ....G.PKGADQ.......SGIPFWGDRA............G.............VQVYRALER.AG.LA..........
tr|H1P505  ....G..RDEDL.......QGRPFVG.KA............G.............ELLDKMIGA.LG.YQ..........
tr|H1XWX4  ....G..REEDE.......QGLPFVG.AA............G.............KLLDKMFAS.IG.LS..........
tr|H2C8Z4  ....G..AREDE.......LGRPFVG.AA............G.............NLLTKLIDKYLN.LK..........
tr|H2J7L7  ....G..ANEDL.......QGRPFVG.RA............G.............NLLDKLLKE.VAKIN..........
tr|H3KDP1  ...PG....K.A.......EGLAFSV.PE............H....E...A.L.PPSLKNIFKE.LE.AE..YGV.....
tr|H3NN78  ....Q...RGQA.......NGLAFSV.NK............G....V...K.M.PPSLRNIFKE.IK.ED..LGI.....
tr|H3NSX2  ...PG....Q.A.......TGRAFEV.AE............G....V...S.A.PPSLVNILKM.LE.KD..LGSV....
tr|H3RRL7  ....G..REEAK.......TGVPFVG.AS............G.............KELMKLIEEYLH.MT..........
tr|H3SCZ2  ....G..AKEAE.......SGRPFVG.NS............G.............RLLNKYLQK.AD.IL..........
tr|H4GIS2  ....G..REEIE.......SHIPFHG.AS............G.............KELMRFLNL.AG.YK..........
tr|H5SI33  ....G..AEEDQ.......QGRPFVG.AA............G.............RLLDRLLGS.IR.IP..........
tr|H6RLZ5  ....G..ATEDE.......TGRPFVG.KS............G.............ALLDQLLAE.AG.LD..........
tr|H7F338  ....G..ETEIH.......QLIPFSG.RA............G............AELMKSLTRA..G.LT..........
```

```
tr|H8I7F5  ....G..RNEDE.......KGEPFVG.AA............G.............RNLDALLEK.SG.IQ..........
tr|H8ZDF2  ...PG.....QA.......VGLSFSV.PS............D....I...R.M.PPSLINIRKE.IL.SS..TGT.....
tr|H9UI22  ....G..ADEDR.......MGLPFVG.PS............G.............QYLDKWLKA.IG.LS..........
tr|H9ZZZ0  ....G..KNEDI.......YGRPFVG.RA............G.............EVLNRALDE.IK.VD..........
tr|I0GI60  ....G..RDEDI.......QGLPFVG.AA............G.............KLLTQLLQS.IG.IE..........
tr|I0I6B7  ....G..AEEDR.......LGRPFVG.RS............G.............QYLTEVLAR.CG.VA..........
tr|I1A005  .......QINIA.......DGLAFST.NH............I........K.T.PASLKNIFKE.VK.KD..YPN.....
tr|I1B0L2  ...PG.....KA.......DGLAFSI.SQ............D....F...PGK.LDSLGNIFKE.LH.DD..LG......
tr|I2K0D9  .......NFNQA.......HGLAFSV.HD............P...RT...R.P.PPSLNNIYKC.LK.ID..YPD.....
tr|I3CUN9  ....G..RNEDQ.......QGEPFVG.PA............G.............KLLDNMLLS.MG.VK..........
tr|I3VQA4  ....G....S.A.......CGLAFGT.VP............G....G...K.I.PPSLGNIYKE.LT.RS..IVG.....
tr|I4CBR6  ....G..ADEDA.......QGRPFVG.KA............G.............QLLTRMIQA.MK.FQ..........
tr|I7LIA9  ....G..RFEEK.......IGRPFVG.QA............G.............KNLDEFLNI.LG.LN..........
tr|I7LKX1  ....G..KQEDL.......SGRPFVG.RA............G.............KLLEDLLAG.ID.LS..........
tr|J0UVA7  ....G..TSDDR.......SAQPFDG.KA............G.............LLLQAMLNSAIP.............
tr|J5SPB4  ....G...PGQA.......HGLAFSV.KR............G....V...A.I.PPSLRNMYKE.MT.TA..IPG.....
tr|J7R1V5  .......NFNQA.......HGLAFSV.KS.........................PTPAPPSLKNIYKE.LK.SN..........
tr|J9E0C3  ....G..RDEDR.......EGKPFIG.RS............G.............KLLDKMFAA.IG.LD..........
tr|J9Z1B8  ....G...PHQA.......HGLCFSV.PD............G....I...K.P.PPSLINIFKE.LE.DD..IDK.....
tr|K0IF13  ....G..KSEDE.......KGRPFVG.AA............G.............RVLDNLLEK.AG.IG..........
tr|K0KF39  .......NHNQA.......HGLAFSV.KS............P....T...P.P.PPSLKNMYKA.LK.ID..YPD.....
tr|K1JXC9  ....G..SEEDL.......QGIPFVG.KS............G.............QLLTQMLES.IGIER..........
tr|K1JYF3  ....G...PGQA.......EGLSFSV.PA............G....I...K.V.PPSLRNIKKE.LQ.RD..LGV.....
tr|K1LNP2  ....G...PDQA.......NGLAFSV.NP............G....Q...K.L.PPSLRNIFQE.LA.QD..LGQ.....
tr|K1ZRE1  ....G..QREAE.......TGRPFCG.AA............G.............KILDELLVS.ID.LK..........
tr|K2A3B6  ....G..YWEDQ.......QGLPFVG.NA............G.............KLLDQLLAT.AH.LS..........
tr|K2B4F4  ....G..KDEDL.......QGKPFVG.AA............G.............KFLNELLNS.IN.LE..........
tr|K2C359  ....G..ADEDE.......QGRPFVG.RA............G.............QLLTKILES.VN.IS..........
tr|K2CLQ5  ....G..KNEAI.......VGRPFIG.RS............G.............KLLRSMIVS.IG.LK..........
tr|K2DEQ6  ....G..EVEDA.......TGRPFVG.RA............G.............KLLESLLGE.IG.YK..........
tr|K2E7J1  ....G...EKQA.......NGLSFAV.ND............G....I...T.L.PPSLKNIYKE.IQ.ND..L.......
tr|K2E9B4  ....G..RNEDE.......TGIPFVG.RA............G.............KLLDKILES.QN.IT..........
tr|K2ETW7  ....G..ANEDL.......QGEPFVG.RA............G.............MLLNSMLQS.IG.LK..........
tr|K2F640  ....G..HNEDK.......IGEPFVG.QA............G.............KLLDKLFPL.AG.LT..........
tr|K4KIT8  ....G...PGQA.......HGLSFSV.PV............G....Q...A.L.PPSLKNIYKE.LY.AA...PA.....
tr|K6GHM9  ....G...KGQA.......NGLSFSV.NK............G....C...K.I.PPSLKNIFIE.LK.ND..L.......
tr|K7YPM8  ....G..ADEDH.......QGKPFVG.VS............G.............RLLDSMLDS.IG.LS..........
tr|K8ZAC1  ....G...EGQA.......QGLSFSI.QK............G........RKL.PPSLQNIYKE.LY.ED..LAI.....
tr|K9VTI9  ....G..AQEDE.......TGKPFVG.AA............G.............KVLERMLKK.AN.LS..........
tr|L0A9A2  ....G..RNEDL.......EGIPFVG.LA............G.............KLLDSLLNE.NN.IK..........
tr|L0B4R8  .......NEGQA.......DGLAFSV.PA............N....C...T.I.PPSLRNINKE.LK.QE..YNN.....
tr|L0B866  ....G...KDQA.......HGFAFSV.LE............N....C...K.K.PPSLKNICKE.LS.LE..YPS.....
tr|L0B8A6  ....G..EYEDI.......EGKPFVG.KS............G.............KLLDSMLSS.IF.IS..........
tr|L0DFI2  ....G..ADEDR.......TGEPFVG.RA............G.............VLLTDMITKGMG.LQ..........
tr|L0L178  ....G..KNEDE.......SGIPFCG.RA............G.............KVLDELIDY.MA.LE..........
tr|L2GQC8  ...PG.....QA.......MGLSFSV.PR............G....I...K.I.PPSLINIYQE.LS.KD..IPG.....
tr|L2GX11  ...RG....R.A.......TGLAFSV.PP............N....V...P.R.PPSLRNIMAE.VK.RN..YGN.....
tr|L5N8A0  ....G..EKEAV.......EGVPFIG.RA............G.............KELDKQLEY.LG.LE..........
tr|L8HA23  ....G...PGQA.......HGLAFSV.AL............G....V...T.P.PPSLKNIFKE.LE.ND..PEV....D
tr|L9X1W2  ....G..KQEVT.......QGEPFVG.QA............G.............QQLDRALES.IG.HD..........
tr|M0BKB2  ....G....N.W.......TGMAYTARHS............G.............RRIRRMMAE.VG.YA..........
tr|M0CBA6  ....G..ATEVA.......EGRPFVG.RA............G.............GRLDSLLET.TG.ID..........
tr|M0MGS7  ....GAGTPEADRWKGGNWTGMAYTARHS............G.............RKIRELFGD.LD.FD..........
tr|M1E4Q4  ....G..ETEDI.......QGLPFVG.KA............G.............QLLTKIFES.VS.LN..........
tr|M1LNF1  ...NG....Q.A.......NGLAFSI.NS............N....S...H.P.PPSLKNIYKE.LS.IE..YKQ.....
tr|M1QKH3  ....G..ADEDV.......EGKPFVG.KA............G.............KLLTQELER.VGLLR..........
tr|M1VCA8  ...VG.....QA.......HGLCFSV.PR............G....V...V.P.PPSLLNIYGE.LA.RE..FPESDPEP
tr|M1Z9H8  ....G..AKEIE.......LRKPFVG.QA............G.............KHFEEFLEV.LN.IK..........
tr|M2XVU5  ....G...PGQA.......HGLCFSV.PT............G....V...K.C.PPSLENIFKE.LE.TD..IPG.....
tr|M3EFG6  ....G..ENEVE.......TGIPFTG.RA............G.............KELMASLHS.VG.LA..........
tr|M4YU22  ....G..ENEDL.......QGKPFVG.RA............G.............EILNKILED.VG.LD..........
tr|M5ADY8  .......KDNQA.......DGLCFSV.PD............G....I...P.I.PASLKNIFTE.VN.NC..LNL.....
tr|M5IVB6  ....G...PNQA.......QGLSFSV.PD............T....C...P.A.PPSLRNIFKE.LE.LE..YPD.....
tr|M6D8H9  ....G..KQEDL.......TGRPFVG.KA............G.............ELLTRIIERGMG.VS..........
tr|M7WR77  ...DG....Q.A.......HGLCFSV.RP............G....V...K.I.PPSLRNIYKE.IK.EE..YPA.....
tr|N0BBN4  ....G..REEDI.......HGRPFVG.NA............G.............KLLTEMISRKLN.LS..........
tr|N1V4N6  ....G..YRGMR......MTGVPFSN.RAIIEGRIDPFGLFG...AH..HGFTLPPDARTVAAEPTATVM..........
tr|N1Z687  ....G..GDEEK.......QGRPFVG.KA............G.............KNLSSFLET.IG.LK..........
tr|N2BLL9  TQD.G..EIPQA.......MGLSFSV.PK............V....V...P.I.PPSLKNIYKE.LE.NS..LG......
tr|N9SHT1  ...EG....Q.A.......DGLAFST.KA............K....I.....L.PPSLKNLFEE.IK.NS..Y.......
tr|N9V498  .......NPNQA.......DGLAFST.RL............K..........KCPKSLANIIKE.VK.KD..........
tr|Q025I0  ....G..ADEDE.......QGIPFVG.RA............G.............QLLTQMIENTSK.............
tr|Q0C0Y4  ....G..AQEDR.......QGKPFVG.KA............G.............QLLDKMLAA.IG.RN..........
tr|Q0EZR7  ....G..RDEDL.......QGEPFVG.RA............G.............QLLDRMMAA.IG.LD..........
tr|Q12TN4  ....G..KKEDE.......TGVPFCG.RA............G.............KILDELIEY.MG.LE..........
tr|Q1AV32  ....G..GREVE.......LGRPFVG.RA............G.............RLLDELLEE.AG.ID..........
tr|Q1AYQ1  ....G..ENEDA.......TGLPFVG.RA............G.............KLLDNILAA.VN.LS..........
tr|Q1GY28  ....EDHGIQQA.......HGLSFSV.PE............G.........VRPPPSLRNMLKE.MQ.TD..........
tr|Q1N1I3  ....G...EGQA.......HGLSFSV.PD............G....I...K.I.PPSLRNIYKE.LN.RD...LG.....
tr|Q23W06  ...PG.....QA.......MGLCFSV.KK............G....V...K.P.PPSLVNIYKS.LS.NDPKIKG.....
tr|Q3SA81  ....G..KWEDI.......KGRPFVG.AA............G.............KYLDELLAT.IG.LS..........
tr|Q53CZ3  ....G.....QA.......TGLAFSV.AP............D....Y...S.I.PPSLKNIFKE.IA.NT..VPG.....
tr|Q5DC15  ....G...PRQA.......HGLAFSV.CR............P....V...P.A.PPSLINMYKE.IS.SS..MNLV.NSD
tr|Q5WES9  ....G..RDELQ.......THKPFTG.RA............G.............ANLNGFLKE.AG.IN..........
tr|Q6CAC0  ....G...KNQA.......HGLAFSV.NP............P....T...L.P.PPSLKNMYKC.LK.ND..YPD.....
tr|Q6F1M3  .......NPKQA.......NGIAFSA.SK............D....I...K.T.PKSLINIFKE.LE.ND..L.......
tr|Q6KIL8  .......QKDVA.......DGLAFST.KH............Q........K.T.PASLKNIFKE.LI.YE..FPE.....
tr|Q7VBS6  ....G..AKEDE.......TGIPFVG.RS............G.............KLLNILLEN.EG.FD..........
tr|Q8TK31  ....G..KTEDE.......TGIPFNG.RA............G.............KKLDKMVEY.MG.LS..........
tr|Q9RTK9  ....G..AEEDR.......DGRPFVG.QA............G.............QLLDRILAA.AS.LA..........
tr|Q9WR44  ....G...PGQA.......HGLAFSV.TR............G....T...P.I.PPSLKNIFLA.VR.ES..YPH.....
tr|R1ATM3  ....G..ANEVK.......LKKPFVG.QA............G.............KHLEEFLDI.LE.LQ..........
tr|R1E4B2  ....G..REEDK.......QGKPFVG.RA............G.............QVLTENIKNILN.MD..........
tr|R1FRL1  ....G...PGQA.......HGLAFSV.AR............G....V...A.V.PPSLKNIYKE.LQ.DD..CPG.....
tr|R1GL12  ....G..AEEDR.......LAKPFVG.LS............G.............QLLDKMLSV.LG.WD..........
tr|R2SFV7  ....G..RKEIA.......SLVPFSG.AA............G.............VELDKNLTL.AG.LK..........
tr|R2SXC9  ....G..ETEIR.......TKIPFSG.RS............G.............KKLDGWLAE.SD.LN..........
tr|R4KRR4  ....G..GEEIK.......QGMPFVG.QA............G.............RNLDEYLSL.AG.LG..........
tr|R4VE87  ....G..KTEDA.......RGEPFVG.RS............G.............QLLDAMLAA.IG.LD..........
tr|R5ARL3  ....G..AEETK.......QAVPFVG.KA............G.............KQLDALLQM.TG.IP..........
tr|R5B4U8  ....G..REEAA.......AGHPFVG.KA............G.............RQLNELLEL.AG.IA..........
tr|R5DX78  ....G..EMEDE.......TGRPFVG.RA............G.............QLLNDFLKE.AG.IS..........
tr|R5MPG0  ....G...TGEA.......HGLSFSV.RE............G....V...P.M.PPSLANIFKE.LY.DD..TGI.....
```

```
tr|R5PZ23  ....G..NEEDR.......QGIPFVG.VS............G.............QLLDKMLAS.VG.LN..........
tr|R5QX00  ....G..ADEDR.......IGVPFVG.RS............G.............HLLDKMLAA.VH.MD..........
tr|R5Z1D7  ....G...PGQA.......QGFSFSV.PA............S....M...P.I.PPSLQNIYKE.LE.DE..FQQ.....
tr|R6HGV3  ....G...PNQA.......MGLSFSV.PK............D....E...K.L.PPSLRNIYKE.MV.SD..LGC.....
tr|R6HL97  ....G...EGQA.......NGMAFAV.NE............G....V...P.L.PPSLVNIYKE.IK.SD..IGD.....
tr|R6PFR8  ....G..ADEDA.......SGTPFVG.RA............G.............QLLNKFLEE.AG.ID..........
tr|R6PI86  ....G..YWEDQ.......KGEPFVG.KA............G.............QLLDKIFAS.VG.LS..........
tr|R6R865  .......NPHQA.......MGLAFSV.PE............N....E...V.L.PPSLKNIFKE.IE.NE..YHV.....
tr|R7BZ83  ....G...PGQA.......VGMSFSVPPE............L....R...K.L.PPSLKNIFRE.IA.LE..YDT.....
tr|R7DQ64  .......EPNQA.......MGLSFSV.PE............G....E...P.L.PPSLQNIYKE.IE.ND..LGV.....
tr|R7DXQ1  ....G..YYEEV.......QQEPFVG.RA............G.............EKLNQILKA.MG.LS..........
tr|R7I8P1  ....G..QVEDE.......EGLAFVG.PA............G.............QLLTKMLAA.IS.LP..........
tr|R7IAS2  ....G..AEEDL.......QGKPFVG.KS............G.............QLLTKALES.VG.LQ..........
tr|R7IHQ8  ....G..EQEAM.......SGRPFVG.KA............G.............KNLDGFLTV.LQ.LD..........
tr|R9ADN8  ....G...PGQA.......HGMAFSV.KH............G....V...R.V.PPSLQNIYKE.LK.NE..YPG.....
tr|R9LKN0  ....G...PGQA.......HGLAFSV.NP............T....V...Q.T.PPSLKNIYKE.LE.AE..YNT.....
tr|S0EV15  ....G..MHEDA.......TGKPFVG.RA............G.............VLLDACLRE..NSIS..........
tr|S0FG57  ....G..KDEVS.......QGKPFVG.AA............G.............KNLKEFMDL.IG.IT..........
tr|S2J4F7  .......NFNQA.......HGLCFSV.VK............G....V...K.V.PPSLVNMYKA.LA.ME..YKD.....
tr|S3JT40  ....G..ADEDR.......QGRPFVG.RA............G.............QLLDKMLEA.IQLSR..........
tr|S4NBY8  ....G..DQEVK.......IGRPFMG.PS............G.............KELDRWLAS.LG.LT..........
tr|S5MFT9  ....G...ENQA.......NGIAFSV.SN............R....V...K.T.PPSLRNIFKE.LK.ND..L.......
tr|S5RQ34  ...PG.....IA.......MGYCFSV.PN............N....S...NYI.PPSLRNIFKE.MK.ND..IGI.....
tr|S6G4W1  ....G..STEDR.......EGIPFCG.AS............G.............QLLDKMFAA.IN.LN..........
tr|S9VE50  .......DIGQA.......HGLCFSV.QP............G....V...R.P.PPSLKNMYKE.LE.SD..VPG.....
tr|T0AYQ1  ....H....DQA.......MGLCFSV.PM............G....T...I.L.PPSLKNIFKE.LK.SE..YSD.....
tr|T0DRC9  ENQ.Q..ELPVA.......MGLSFSV.EK............N....A...P.I.PPSLKNIFKE.LH.AN..LG......
tr|T0LSQ5  ....G..KSEDE.......NGIPFSG.RS............G.............QLLRKSLTL.TG.VS..........
tr|T0MGQ8  ....G...EGQA.......TGLSFSV.PK............N....K...K.I.PPSLKNIFKE.LK.DD..IIG.....
```

```
                                          110                           120
sp|P12295 FT..RP...........NH...G...........YLESWA.R...........QG.......VLLL.NTVL.........
tr|Q8KLI8 .........................................R...........EE.......VYIT.NIVK.........
sp|A1QYK3 KIKTIP............N...G...........DLTRWA.T...........QG.......VFLL.NSIL.........
sp|A1SUE5 YK..IP...........ES...G...........NLAHWA.K...........QG.......ILLL.NSVL.........
sp|B0B8I2 ....R.N..........ES...G...........CLQAWA.D...........QG.......VLLL.NTVL.........
sp|B1AJI8 QIKRTN............P...............DLSDWA.K...........QG.......VLLL.NTVL.........
sp|P12888 FSP..P...........DH...G...........CLDAWA.S...........QG.......VLLL.NTIL.........
sp|Q057V4 ..SFYK............KNIHG...........CLESWA.K...........QG.......VFLL.NSIL.........
sp|Q315T1 AP..............AAA...GMQHNGVQ...TDLTPWA.E...........QG.......VLLL.NTAL.........
tr|A0LFG4 .........................................R...........EA.......VYIC.NVVK.........
tr|A0LH32 .........................................R...........ED.......LFIT.NLLK.........
tr|A0LRS0 .........................................R...........AT.......VAIV.NVLK.........
tr|A0RV64 .........................................R...........ST.......VYIT.NVVK.........
tr|A1BDC5 .........................................R...........EE.......VFIC.NILK.........
tr|A1RYC0 .........................................R...........EE.......VFIT.NVVK.........
tr|A1US05 .........................................R...........EN.......VYIA.NTVP.........
tr|A4GHT2 RP..PP...........KN...G...........DLTFWA.K...........QG.......VLLL.NTVL.........
tr|A4HXS3 FQ..AP...........RH...G...........YLQSWS.E...........QG.......MLML.NATL.........
tr|A5KS51 .........................................R...........ED.......VYIT.NIVK.........
tr|A5UQ47 .........................................S...........DETATASLRCFIT.NTVR.........
tr|A6DBB2 .........................................R...........KK.......HFLITNAFP.........
tr|A6DK00 ....SPA..........LD...G...........DLSPWA.Q...........QG.......VLLL.NTSL.........
tr|A6GN76 .........................................R..........TNN.......TYIA.NVVK.........
tr|A7ANQ0 IE..RS............H...G...........DLTYWA.Q...........QG.......VFLL.NTVL.........
tr|A8A982 .........................................K....................YYIT.NVVK.........
tr|A8E1M5 FSM..P...........KC...G...........CLDAWC.R...........EG.......VLLI.NTVF.........
tr|A8F067 .........................................R..........KNN.......AYIT.NTVF.........
tr|A8MA01 .........................................R...........GN.......LYIT.NVVK.........
tr|A8SNH5 ...IPP...........NH...G...........DLTAWV.E...........QG.......VFLL.NATL.........
tr|A8UAT5 .........................................R...........KE.......VYIT.SAFR.........
tr|A8UU74 .........................................R...........EE.......VYIT.NVCK.........
tr|A9AZ06 .........................................R...........EQ.......VFIA.NVVK.........
tr|A9BHU0 .........................................R...........ND.......VFIT.NIVK.........
tr|A9F5H8 .........................................R...........DE.......VYIC.NIVK.........
tr|A9NHD4 NIDIST............K...G...........DLTSWA.R...........QG.......VLLL.NTVL.........
tr|B0VFE1 .........................................R...........SD.......VYIT.NIVK.........
tr|B1GA08 ....PP...........RH...G...........CLDTWA.K...........QG.......VLLL.NTVL.........
tr|B1H0K1 .........................................R...........ES.......VYIA.NIVK.........
tr|B1I4W5 .........................................R...........NR.......VFIT.NVVR.........
tr|B1VA82 NIESHQ............N...N............LTPWA.L...........EG.......VLML.NSIL.........
tr|B1Y7I1 ...ARP...........GH...G...........SLSRWV.G...........QG.......VLLL.NSSL.........
tr|B1ZZ66 .........................................R...........DE.......VYIG.NIMN.........
tr|B2A668 .........................................R...........DD.......VYIT.NIVK.........
tr|B2KCZ8 .........................................R...........EE.......VFIA.NMVK.........
tr|B3DV47 .........................................R...........EQ.......VYIA.NVLK.........
tr|B3U4N1 CS..VP...........NH...G...........CLEPWA.R...........QG.......VLML.NSVL.........
tr|B3U4U7 .........................................R...........AD.......IYIA.NVIK.........
tr|B5II11 .........................................N...........RD.......AYIC.NVVK.........
tr|B5LRM7 TKI..V...........DH...G...........CLEAWA.K...........AG.......VLLL.NTTL.........
tr|B5Y8I5 .........................................R...........HS.......LFIT.NVIK.........
tr|B5YEZ9 .........................................R...........ED.......IYIA.NVLK.........
tr|B6A3R0 .........................................R...........RR.......CYVT.NAVK.........
tr|B6AFI4 ..............................VPHGDLTSWA.N...........QG.......VFML.NSLL.........
tr|B6K2R2 FE..IP...........NT...G...........YLVPWA.E...........QG.......VLML.NVNL.........
tr|B6KW10 ..............YPGSKLVDQHTNRISSFVHGDLTSWASQ............G.......VFLL.NSLL.........
tr|B7FQS1 IKFLMP...........RH...G...........HLIRWA.K...........QG.......VLML.NACM.........
tr|B7KM64 .........................................R...........GQ.......IYLT.NIIK.........
tr|B8C427 IS..PP...........EH...G...........NLEGWA.R...........QG.......VLLL.NTVL.........
tr|B8CX81 .........................................R...........ED.......VYIT.NIVK.........
tr|B8EJL3 .........................................R...........RD.......VYIA.NVVP.........
tr|B8HFW5 ......................................WEVL...........AE.......LGVL.PLLW.........
tr|B8I5K9 .........................................R...........DD.......LYIT.NAIKYRLGRLNPK
tr|B9KXL9 .........................................R...........EQ.......VYIT.NVVK.........
tr|B9XNJ8 .........................................R...........SD.......FFIT.NICK.........
tr|B9Z7Q7 GL..............PAS...G...........DLTPWA.R...........QG.......VLLL.NSVL.........
tr|C0BNZ8 LDCAYP...........IS...G...........DLSLWA.N...........QG.......VLLL.NAAL.........
tr|C0X269 .........................................R...........ET.......VYIT.SAVR.........
tr|C1A7L2 .............................LPPATRTMPWDGARFAAAGLRPEPHG.......VALG.NALD.........
tr|C1D9B0 ...SLP...........AH...G...........DLSAWS.H...........QG.......VLLL.NTVL.........
tr|C1F1L1 .........................................R...........SQ.......VYIA.NIVK.........
tr|C1G9A4 FV..KPP........N.NG...G...........HLAPWA.N...........RG.......VLML.NTCL.........
tr|C2MIJ3 .........................................R...........EE.......VYIT.SAVR.........
tr|C3X2T7 .........................................R..........GEN.......VYIA.NIVK.........
tr|C5CIT9 .........................................R...........ED.......VYIT.NIVK.........
tr|C5NVK4 ....NP..........AKT...G...........NLESWF.N...........QG.......VFLL.NAVL.........
tr|C7H0A3 MYLLKNRNLYIESQNY..D...S...........VLLPWA.E...........QG.......VLLL.NAVL.........
tr|C7M312 .........................................R...........SG.......LYIA.NTVK.........
tr|C8W879 .........................................H...........QD.......VFIS.NVVK.........
tr|C9M5Q7 .........................................R...........DE.......LFIT.NAVK.........
tr|C9RLM9 .T..PP...........PH...G...........NLEGWA.H...........QG.......ILLL.NASL.........
tr|D0MK10 .........................................R...........EE.......VYIT.NVLK.........
tr|D0PPD4 FKI..P...........PS...G...........CLDKWA.E...........QG.......VLLL.NCIL.........
tr|D0U4F0 IT..PS...........QS...G...........NLTHWA.T...........QG.......VLLL.NSAL.........
tr|D1ART8 DIE.RT............E...R...........NLTDWA.E...........QG.......VLLL.NTVL.........
tr|D1C3J5 .........................................R...........ED.......LWIT.NVVK.........
tr|D1CDM9 .........................................R...........ES.......TYIT.NIIR.........
tr|D1JDZ5 .........................................R...........AN.......IYIT.NVLK.........
tr|D1PN05 .........................FGPVAVRHNDLTPWA.R...........QG.......VLLL.NTVL.........
tr|D1Y5H9 .........................................R...........EE.......IFIT.SICK.........
tr|D2EF83 .........................................R...........DS.......VFIT.NAVK.........
tr|D2Z573 .........................................R...........ED.......LFIS.NIVH.........
tr|D3B454 FK..TP...........NH...G...........FLEKWA.K...........QG.......VLML.NDTL.........
tr|D3E6H2 .........................................R...........DQ.......VYIT.SSMRSRPYKDKVL
tr|D3FBP7 .........................................R...........AD.......VFIA.NVLK.........
tr|D3QZ35 LT..SS............H...G...........DLTYWA.K...........QG.......VFLL.NRIL.........
tr|D4G8B4 FK..IP...........NH...G...........CLTRWT.K...........QG.......IFLL.NSIL.........
tr|D5C4I1 .........................................R...........EQ.......IYIS.NILK.........
tr|D5E730 .........................................Q...........SD.......WAVI.NTVK.........
tr|D5EGK6 .........................................R...........EF.......FFIT.NMVK.........
tr|D5EHP1 .........................................R...........ED.......VYIG.NILK.........
tr|D5MIW4 .........................................R...........EQ.......VYIC.NIVK.........
tr|D5RKP0 ....AP...........DH...G...........CLTRWA.D...........QG.......VLLL.NTAL.........
tr|D5U040 .........................................R...........EE.......VFIT.NIVK.........
```

```
tr|D5U570  .........................................K...........EK.......YYIM.NALK.........
tr|D5V6V9  DS..SC...........LD...G...........DLTPWA.K...........QG.......VLLL.NTIL.........
tr|D5WWR6  .........................................P...........AR.......LYIT.NAVK.........
tr|D7CKV6  .........................................R...........DE.......VYIT.NIVK.........
tr|D7CWJ9  .........................................R...........ED.......VYIT.NILK.........
tr|D7UXZ6  .........................................R...........DE.......VYIT.SAVR.........
tr|D8LFW4  TP..TK............S...G...........NLDSWC.S...........QG.......VLLL.NTVL.........
tr|D8UI66  ....RP...........AH...G...........DLSAWS.V...........QG.......VLLL.NTCL.........
tr|D9Q1V3  .........................................R...........QD.......VYIT.NVVK.........
tr|D9QQQ4  .........................................R...........KD.......VYIS.NIVK.........
tr|D9S3H2  .........................................R...........KK.......VYIT.NVVK.........
tr|D9SCW5  .........................................R...........SE.......VYLA.NVIK.........
tr|D9Y2E5  .................AEFRTG...........ELEGWA.K...........QG.......VLLL.NTVL.........
tr|E0S9I8  FE..IP...........SH...G...........DLTKWA.E...........NG.......VLLL.NDVL.........
tr|E0SQC9  .........................................R...........DS.......VYIT.NVVK.........
tr|E0TBI7  .........................................R...........GD.......VLIT.NAVY.........
tr|E0TIS2  .........................................K...........KE.......IYIT.NVIK.........
tr|E1IBI3  .........................................L...........  .......FVVGWNAYP.........
tr|E1L0U9  ...PMP...........SS...G...........NLEPWA.R...........QG.......VLLL.NASL.........
tr|E1QFX8  .........................................R...........EA.......VYVT.NIVK.........
tr|E1QNN8  .........................................R...........EE.......VYIT.NVVK.........
tr|E1QWE2  E...............ARG...G...........DLTPWA.R...........QG.......VLLL.NTVL.........
tr|E1R614  .........................................R..........SEH.......LFIG.NVIK.........
tr|E2IUG8  TPA..A...........GH...G...........CLEKWA.R...........QG.......VLLL.NTTL.........
tr|E2SYG7  .........................................R...........YE.......VFVT.NAVL.........
tr|E3D056  .........................................R...........ED.......VYIT.NVVK.........
tr|E3IP47  ASDEGKKRHADALPEKTAA...GHDGGGLRPPSTDLTRWA.R...........QG.......VLLL.NTVL.........
tr|E4KQ36  ....PVV..........KH...G...........DLSAWA.Q...........QG.......VLLL.NTSL.........
tr|E4RKG8  .........................................R...........DE.......IYIT.NVVK.........
tr|E6N689  .........................................R...........ED.......VFIT.NVVK.........
tr|E6PFU2  .........................................R...........ED.......VYIA.NTVK.........
tr|E6W1H1  EP..............PVD...G...........DLQWWA.R...........QG.......VLLL.NRVL.........
tr|E6W552  .........................................R...........EE.......LYIA.NVVK.........
tr|E7A9L4  IFP..P...........KH...G...........NLQSWA.K...........QG.......VLLL.NAIL.........
tr|E7FX98  ....K.N..........EH...G...........DLSAWA.K...........QG.......VLLL.NTLL.........
tr|E7H418  .........................................R...........RD.......CVIL.NVLK.........
tr|E7H4Y1  R.R..............TR...G...........DLSDWA.A...........AG.......VLLL.NTIL.........
tr|E8LLT1  FS..IP...........DH...G...........CLIPWA.R...........QG.......VLLL.NAVL.........
tr|E8N338  .........................................R...........KD.......VFIT.NVVK.........
tr|E8T398  .........................................R...........DQ.......IYIT.NVLK.........
tr|F0EKF3  .........................................R...........ED.......VYIT.SAVR.........
tr|F0NSC6  .........................................R...........DQ.......VYIT.SAVR.........
tr|F0RSJ4  .........................................R..........SNA.......VYIA.NIVK.........
tr|F0SG07  .........................................R...........EE.......LYIC.NILR.........
tr|F0Y985  AA...P...........AG...G...........DLGGWA.D...........QG.......VLLL.NTAL.........
tr|F2LXL6  .........................................P...........QD.......VYIA.NCLK.........
tr|F1A0X7  FK..QPL..........KN...G...........FLEPWA.K...........QG.......VFLL.NAAL.........
tr|F2NFM2  .........................................R...........QE.......VYIT.NINK.........
tr|F3KRD3  APRPVP...........AD...G...........SLQGWA.R...........QG.......VLLL.NTCL.........
tr|F3QMU0  .........................................R..........EKD.......LCIF.NTLK.........
tr|F4A8X0  .........................................R...........DK.......VRIT.NTCF.........
tr|F4C0J7  .........................................R...........ED.......VFIT.SVIK.........
tr|F4GMI7  .........................................Q...........TP.......VFFT.NVIK.........
tr|F4GSY3  TFV..Q...........QR...H...........SLVRWA.R...........QG.......VLLL.NTSL.........
tr|F4HNK0  .........................................R...........NE.......VYIT.NIVK.........
tr|F4NZD5  FK..RP...........SH...G...........YLNSWC.Q...........QG.......VLLL.NASL.........
tr|F4QWL4  .........................................D...........  .......AFIT.NTVF.........
tr|F5SL17  .........................................R...........AE.......IFIT.NIVK.........
tr|F5Y215  .........................................RGAPGEEGDGAGGN.......VYIA.NVLK.........
tr|F5YGI3  .........................................R..........EKN.......CFIA.NVVK.........
tr|F7NHV1  .........................................R...........DR.......VYTT.NIIK.........
tr|F7PTA1  LNIKTP...........TH...G...........DLNCWA.K...........QG.......VLLL.NTFL.........
tr|F7S094  AQ..IP...........MH...G...........DLQAWA.E...........QG.......VLLL.NTSL.........
tr|F7XVS9  .........................................K...........EN.......LFIT.NTVF.........
tr|F8ADN2  .........................................R...........SE.......VYIT.NVVK.........
tr|F8L559  ....PPA..........HH...G...........NLEKWA.K...........QG.......VLML.NATL.........
tr|F9CXI5  .........................................R...........ES.......IYIT.NIVK.........
tr|F9DTW2  .........................................R...........ED.......VYMT.SSFR.........
tr|F9LK36  .........................................R...........EN.......IFIS.SIVR.........
tr|F9ZLD4  .........................................R...........WY.......QYGCANIVR.........
tr|G0LJW8  .........................................R...........DS.......IRIT.NCVR.........
tr|G0QBL8  .........................................R...........SE.......VYIT.NLVK.........
tr|G0QFK7  .........................................R...........RE.......LYIT.NLVK.........
tr|G0U4E4  FV..EP...........RH...G...........YLQHWA.E...........QG.......ILML.NATL.........
tr|G2RZ19  QIDHFN............N...H...........SLENWV.K...........QG.......VLLI.NTCW.........
tr|G4RL69  .........................................R..........SRD.......VFIT.NVVK.........
tr|G4STH7  .........................................R...........DD.......VFIT.NILK.........
tr|G4T6H9  FV..IP...........KS...G...........DLTPLA.Q...........AG.......VLWL.NTSL.........
tr|G7WNS9  .........................................R...........SR.......VFIT.NVVK.........
tr|G8BJ42  FK..VPDYKELSKSGKPGG...G...........NLTKWATD...........GG.......VLML.NAVL.........
tr|G8LWR8  .........................................R...........EE.......IYIT.NAIK.........
tr|G8TXV8  .........................................R...........DT.......IWLA.NAVR.........
tr|G8XT11  FQP..P...........NH...G...........CLDAWC.R...........QG.......VLLL.NTVF.........
tr|G9EIR0  ......................................................QD.......FYIA.NVLK.........
tr|G9XY09  GIDHHA............S...G...........NLDHWA.K...........EG.......VFLY.NTIG.........
tr|H0E6E4  .........................................R...........DE.......VFVT.NVLR.........
tr|H0KUY0  NINRTK............K...............ELDDWG.Q...........QG.......VLLL.NATL.........
tr|H0Q5N1  EV..............RSS...G...........DLTGWA.Q...........QG.......VLLL.NTIL.........
tr|H1D376  .........................................R...........DH.......VYVT.NIVK.........
tr|H1P1U4  ........................EVPAE.....AYEVWDGARFRELGICPRLKG.......AALS.NAFP........Q
tr|H1P505  .........................................R...........ED.......VYIA.NVVK.........
tr|H1XWX4  .........................................R...........DN.......VYIA.NVLK.........
tr|H2C8Z4  .........................................R...........EQ.......VYIT.NLVK.........
tr|H2J7L7  .........................................R...........ED.......LYIT.NVVK.........
tr|H3KDP1  R.R..............TQ...G...........RLIDWA.Q...........AG.......VLLI.NTIL.........
tr|H3NN78  VN..T.............N...T...........DLTNWA.N...........QG.......ILLL.NTVL.........
tr|H3NSX2  ....IK...........GR...G...........LFAHWE.S...........EG.......VLLL.NTVL.........
tr|H3RRL7  .........................................R...........ED.......VYIT.SVVR.........
tr|H3SCZ2  .........................................R...........ED.......AYVT.NVIK.........
tr|H4GIS2  .........................................R...........ED.......VYIT.SVVR.........
tr|H5SI33  .........................................R...........ER.......VFIT.NTVR.........
tr|H6RLZ5  .........................................R...........GR.......AAVL.NVVK.........
tr|H7F338  .........................................R...........ED.......VYMT.SAVR.........
```

```
tr|H8I7F5  ...........................................R............RD.......VYIT.NVVK..........
tr|H8ZDF2  GS..KC............EK....G...........SLVSWA.E............QG.......VLLL.NSIL..........
tr|H9UI22  ...........................................R...........HRN.......VFIT.NVVK..........
tr|H9ZZZ0  ...........................................R............IN.......VYIT.NVVK..........
tr|I0GI60  ...........................................R............SE.......VFIG.NIVK..........
tr|I0I6B7  ...........................................R............SD.......VYIT.NIVK..........
tr|I1A005  AKFGTN..............................SLIKWA.K............QG.......VLLL.NTVL..........
tr|I1B0L2  .V..SR............DR....T...........DLADWA.A............QG.......VLLL.NTAL..........
tr|I2K0D9  FQ..IP............KS....G...........DLTKWA.E............QG.......VLLL.NTCL..........
tr|I3CUN9  ...........................................R...........GEN.......AYIA.NIVK..........
tr|I3VQA4  FIA..P............SS....G...........CLDAWC.K............EG.......VLLI.NSVF..........
tr|I4CBR6  ...........................................R............ED.......VYIA.NIVK..........
tr|I7LIA9  ...........................................R............DD.......LYIT.NVVK..........
tr|I7LKX1  ...........................................R............DE.......VFIA.NIVK..........
tr|J0UVA7  ...........................................N............AS.......IYKA.HLVK..........
tr|J5SPB4  FV..AP............KH....G...........DLTEWA.K............HG.......VLLL.NTSL..........
tr|J7R1V5  .........................YADFQIDNSKGDLSCWS.K............QG.......VLLL.NTAL..........
tr|J9E0C3  ...........................................R............ND.......IYIT.NLVP..........
tr|J9Z1B8  KM..DF............SN....G...........NLEHWA.K............QG.......VFLL.NTTL..........
tr|K0IF13  ...........................................R............SQ.......VFIT.NVVK..........
tr|K0KF39  FQ..IPT.........GTSS....G...........DLTKWA.D............RG.......VLML.NACL..........
tr|K1JXC9  ...........................................R............RD.......IAIM.NVLK..........
tr|K1JYF3  PVT...............QQ....G...........SLLSWA.H............QG.......VLLL.NAIL..........
tr|K1LNP2  PF...............RQS....G...........DLSDWA.K............QG.......VLLL.NRTL..........
tr|K1ZRE1  ...........................................R............KD.......VYIT.NVVK..........
tr|K2A3B6  ...........................................R............NDT......VYIT.NVVH..........
tr|K2B4F4  ...........................................R............ED.......VYIA.NVIK..........
tr|K2C359  ...........................................R............EE.......VYIT.NVVK..........
tr|K2CLQ5  ...........................................E............ED.......VYIT.SPVK..........
tr|K2DEQ6  ...........................................R............TD.......VWIG.NIIK..........
tr|K2E7J1  GITSLP.............S....G...........DLSRWA.R............QG.......VLML.NSVL..........
tr|K2E9B4  ...........................................R...........EKD.......VYIC.NTVK..........
tr|K2ETW7  ...........................................R............DD.......VYIA.NILK..........
tr|K2F640  ...........................................R............KE.......VFIT.NIVH..........
tr|K4KIT8  .E..YP............D.....G...........DLSHWA.R............QG.......VLLL.NASL..........
tr|K6GHM9  NIPICK.............H....G...........DLEFWA.Q............QN.......VLLL.NSVL..........
tr|K7YPM8  ...........................................R...........FKS.......VYIT.NIIP..........
tr|K8ZAC1  ....EPVS...........H....G...........ELVSWA.K............QG.......VLLL.NTVL..........
tr|K9VTI9  .........................................TGK............DGD......VYIA.NIIK..........
tr|L0A9A2  ...........................................R............DE.......VYIT.NVVK..........
tr|L0B4R8  .DL..E............TA....K...........DLSFWA.M............QG.......VLLL.NTIL..........
tr|L0B866  VEF..I............RL....S...........NLSCWA.N............QG.......VLLL.NSIL..........
tr|L0B8A6  ...........................................R...........SSN.......AFIS.NIVK..........
tr|L0DFI2  ...........................................R............SE.......VYIA.NVLK..........
tr|L0L178  ...........................................E............NE.......YAII.NTVK..........
tr|L2GQC8  FV..AP............GH....G...........DLTEWA.A............QG.......VLLL.NDTL..........
tr|L2GX11  ..................AT....C...........DLESWA.K............QG.......VLLL.NDTL..........
tr|L5N8A0  ...........................................R............DD.......LYIT.SVVR..........
tr|L8HA23  FT..KP............DH....G...........NLEKWA.R............QG.......VLLL.NAVL..........
tr|L9X1W2  ...........................................R............RD.......LYVT.NLVK..........
tr|M0BKB2  ........................................................DE.......SYYT.NAVK..........
tr|M0CBA6  ...........................................R............ET.......IYVT.NLVK..........
tr|M0MGS7  ...........................................N............DD.......LYYT.NAVK..........
tr|M1E4Q4  ...........................................R............KD.......IYIT.NVVK..........
tr|M1LNF1  FTF..E............KE....Y...........NLISWA.N............QG.......VLLL.NNIL..........
tr|M1QKH3  ...........................................Q............RD.......YYIT.NVVK..........
tr|M1VCA8  FR..PP............NH....G...........CLEAWA.D............QG.......VLLL.NAVL..........
tr|M1Z9H8  ...........................................R............EN.......VYIT.NSVK..........
tr|M2XVU5  YR..RP............AQ....G...........NLEHWS.E............QG.......VLLL.NSVL..........
tr|M3EFG6  ...........................................R............ED.......VYIT.SAVR..........
tr|M4YU22  ...........................................R............GR.......LMIT.NTVK..........
tr|M5ADY8  KTSYYP.............IS...G...........SLIHWA.K............QG.......VLLL.NSIL..........
tr|M5IVB6  TTL..P............SS....H...........DLSNWG.R............QG.......VLLL.NTSL..........
tr|M6D8H9  ...........................................R............DS.......VYIA.NITK..........
tr|M7WR77  FD..VP............KH....G...........NLISWA.R............SG.......VLLL.NTSL..........
tr|N0BBN4  ...........................................R............KD.......VFIT.NILK..........
tr|N1V4N6  ........................................WDVL............AD.......LDFL.PLLW..........
tr|N1Z687  ...........................................R............EE.......IYIT.NVVK..........
tr|N2BLL9  ICM..P............NH....G...........DLTQWA.T............KG.......VLLL.NAIL..........
tr|N9SHT1  .PDFSK.............KD...G...........NLKNWA.K............QG.......VLLL.NIVL..........
tr|N9V498  ...........................YPNSIFETYSLDFWA.K............QG.......VLLI.NTVL..........
tr|Q025I0  ...........................................K............EGIPLKRGDVYIC.NVVK..........
tr|Q0C0Y4  ...........................................R...........AEN.......ALIT.NVNY..........
tr|Q0EZR7  ...........................................R............DA.......VYIM.NTIK..........
tr|Q12TN4  ...........................................S............SD.......WAVI.NTIK..........
tr|Q1AV32  ...........................................R............SE.......VYVT.NLVK..........
tr|Q1AYQ1  ...........................................R............EQ.......VYIT.NIVK..........
tr|Q1GY28  ............................VGQTLSSGNLMPWA.K............QG.......VLLL.NASL..........
tr|Q1N1I3  LS..IP............KT....G...........NLESWA.K............QG.......VLLL.NASL..........
tr|Q23W06  FK..TP............NH....G...........DLTKWA.N............QG.......VFLL.NDIM..........
tr|Q3SA81  ...........................................R............DD.......VYIT.NIVK..........
tr|Q53CZ3  FTA..P............SH....G...........CLDCWA.K............RG.......VLLL.NTIL..........
tr|Q5DC15  NW..PP............KH....G...........DLTGWA.K............QG.......VLLL.NAVL..........
tr|Q5WES9  ...........................................R............ED.......IYIT.SVVR..........
tr|Q6CAC0  FD..VPA...........KS....G...........LLTPWA.E............QG.......VLML.NTVL..........
tr|Q6F1M3  GIKHYD.............N....N...........DLSNWV.K............QG.......VLLI.NTCW..........
tr|Q6KIL8  IKIKSN..............................DLSCWA.K............QG.......ILLQ.NTFL..........
tr|Q7VBS6  ..........................................YE............KD.......VFIC.NLLK..........
tr|Q8TK31  ...........................................E............ED.......WFVA.NTVK..........
tr|Q9RTK9  ...........................................R............EE.......AYLT.NVTK..........
tr|Q9WR44  LPL..P............TH....G...........CLDNWA.R............QG.......VLLL.NTTL..........
tr|R1ATM3  ...........................................R............KE.......LYIT.NTVK..........
tr|R1E4B2  ...........................................R............KD.......VYIT.NVVK..........
tr|R1FRL1  FK..PP............QH....G...........HLAGWA.R............QG.......VLMF.NATL..........
tr|R1GL12  ...........................................R............TS.......CYIT.NIVP..........
tr|R2SFV7  ...........................................R............EQ.......IYIT.SAVR..........
tr|R2SXC9  ...........................................R............DD.......IYIT.SAVR..........
tr|R4KRR4  ...........................................R............SD.......VFIT.NAIK..........
tr|R4VE87  ...........................................R...........THD.......VFIT.NVAK..........
tr|R5ARL3  ...........................................R............VD.......LYVT.NAVK..........
tr|R5B4U8  ...........................................R............GD.......VFVT.NAVK..........
tr|R5DX78  ...........................................R...........DED.......VYVI.NTVK..........
tr|R5MPG0  ...................KRTKT............DLTDWA.Y............QG.......VLLL.NSIL..........
```

```
tr|R5PZ23  .........................................R...........ES.......VYIS.NVLP.........
tr|R5QX00  .........................................R...........TQ.......YYIT.NILP.........
tr|R5Z1D7  PV..RR...........S....G...........DLSDWA.S...........QG.......VLLL.NAIL.........
tr|R6HGV3  SM..............PES...G...........DLTSWA.G...........QG.......VLLL.NTTL.........
tr|R6HL97  .................IDDSCG...........TLIGWA.K...........QG.......VLLL.NTVL.........
tr|R6PFR8  .........................................R..........KTQ.......LYIC.NTIK.........
tr|R6PI86  .........................................R..........QND.......VYIC.NTLK.........
tr|R6R865  KM..KN............N...G...........DLTYLA.K...........QG.......VLLL.NSLL.........
tr|R7BZ83  GIH..............TN...G...........DLSGWA.E...........QG.......VLLL.NAVL.........
tr|R7DQ64  HM..KP............N...G...........DLTYLA.K...........QG.......VLLL.NALL.........
tr|R7DXQ1  .........................................R...........DM.......VYIS.NIVK.........
tr|R7I8P1  .........................................R...........DR.......VYIC.NVVK.........
tr|R7IAS2  .........................................R..........GKD.......IAII.NVLK.........
tr|R7IHQ8  .........................................R...........ED.......IYIS.NVVK.........
tr|R9ADN8  FQ..PP...........NH...G...........NLERLCLE...........NG.......ILLL.NSTL.........
tr|R9LKN0  VI..D............RS...G...........DLRDWA.K...........QG.......VLLL.NPIL.........
tr|S0EV15  .........................................R...........KH.......IYIT.NLVR.........
tr|S0FG57  .........................................R...........ES.......VFIT.NVIKYRLGKINEK
tr|S2J4F7  FK..TP...........DH...G...........YLEEWA.K...........QG.......VLLL.NTSL.........
tr|S3JT40  .........................................K...........TN.......CYIT.NVVK.........
tr|S4NBY8  .........................................R...........ED.......IYIT.GVVN.........
tr|S5MFT9  GISHFE............N...N...........DLSMWV.K...........QG.......VFLL.NTSL.........
tr|S5RQ34  ....KR...........SE...T...........DLTDLA.K...........QG.......VFLL.NSIL.........
tr|S6G4W1  .........................................R..........SSN.......IYIS.NTVF.........
tr|S9VE50  FT..TP...........SH...G...........YLQSWA.D...........QG.......ILML.NATL.........
tr|T0AYQ1  FV..IP...........NH...G...........CLYGWA.K...........QG.......VLLL.NATL.........
tr|T0DRC9  VPV..P...........CC...G...........DLSAWA.K...........RG.......MLLL.NAIL.........
tr|T0LSQ5  .........................................K...........HR.......IYIT.NSVK.........
tr|T0MGQ8  FK..IP...........TH...G...........NLEKWA.M...........QG.......VLLI.NDVL.........
```

```
                          130                     140            150
                            .                       .              .
sp|P12295  TV.........R.A............GQA....HSHAS.L.G..WE.T.FTDKVIS....LINQ..............
tr|Q8KLI8  CR.........P.P...........QNRA....PLPDE.AKI..CTDK.WLLKQIE....LI..................
sp|A1QYK3  TV.........E.E............GRP....SSHKD.I.G..WE.I.FTNEVIK....IISK..............
sp|A1SUE5  TV.........E.Q............ADP....GCHAK.K.G..WE.T.FTDNAIS....EINN..............
sp|B0B8I2  TV.........R.A............GEA....FSHAG.R.G..WE.R.FTDAIVT....KLIQ..............
sp|B1AJI8  TV.........N.A............HQP....NSHKN.F.G..YE.E.LIKNVFN....ELRK..............
sp|P12888  TV.........Q.K............GKP....GSHAD.I.G..WA.W.FTDHVIS....LLSE..............
sp|Q057V4  TV.........S.K............GIP....NSHKN.L.G..WE.I.FTDQVIK....FISD..............
sp|Q315T1  TV.........E.A............GRA....GSHAA.L.G..WH.A.VTDDIIR....TVSE..............
tr|A0LFG4  CR.........P.P...........KNRT....PESEE.IAR..CG.P.FLIRQIE....AI................
tr|A0LH32  YR.........PRT.......STGANRS....PAAAE.CRY..AL.P.FLERELD....IL................
tr|A0LRS0  CR.........P.P...........GNRV....PNRVE.IAA..CR.P.WLEAQLA....LL................
tr|A0RV64  CR.........P.P...........KNRV....PSDEE.RDA..CI.E.YLKEEIS....VI................
tr|A1BDC5  CR.........P.P...........QNRN....PLADE.IEC..CM.P.WLLLQLE....LL................
tr|A1RYC0  CR.........P.P...........NNRE....PREEE.IAA..CL.P.YLLAQLR....AI................
tr|A1US05  WR.........P.P...........GNRT....PTARE.MAL..CR.P.FIERQIQ....LA................
tr|A4GHT2  TV.........E.D............SHP....DSHAG.H.G..WE.E.FTDRIVE....ILNL..............
tr|A4HXS3  TV.........E.A............HKA....NSHSKTS.G..WT.A.FTDAVIQ....HLSQ..............
tr|A5KS51  YR.........P.P...........NNRD....PSPEEKRAF..WP...YVARQLK....VI................
tr|A5UQ47  CC.........P.P...........GNRT....PTAAE.VQA..CR.E.WLDTELD....AI................
tr|A6DBB2  FR.........TIN.........ENKNRT....PKISE.LKI..AS.K.LLEEEIN....IV................
tr|A6DK00  SV.........R.D............GEP....GSHSK.G.H..WE.E.FSAQVLK....SLSE..............
tr|A6GN76  CH.........P.P...........GNRN....PEQAE.IDQ..CS.P.YLVQQIK....AS................
tr|A7ANQ0  TV.........I.D............SQP....FSHKD.Y.G..WT.K.FTDIVID....LINT..............
tr|A8A982  CR.........P.P...........GNRT....PTEEE.VEA..CK.P.YLIEELN....CI................
tr|A8E1M5  TV.........V.K............GQP....GSHEA.L.G..WQ.I.LSDRVLQ....ALSE..............
tr|A8F067  WR.........P.P...........ANRQ....PTLEE.VDI..CR.P.FVEKHIA....LI................
tr|A8MA01  CR.........P.P...........NNRD....PTEEE.INA..CK.P.YLIRQIA....VV................
tr|A8SNH5  TV.........E.K............SKP....NSHKD.I.G..WQ.I.FTDRVIE....IISN..............
tr|A8UAT5  SRPYRYKEKIDRKTK.....QVVLRKYNRP....PTKQE.LIA..HA.P.LLDQEVE....KV................
tr|A8UU74  CR.........P.P...........GNRK....PTPAE.ISA..CF.P.YLKKEID....II................
tr|A9AZ06  CR.........P.P...........GNRD....PEPSE.IAA..CA.H.FLDRQIA....AL................
tr|A9BHU0  CR.........P.P...........QNRV....PTQEE.VKS..CQ.S.YLDGQII....VI................
tr|A9F5H8  CR.........P.P...........KNRK....PEPAE.MAA..CS.P.YLTAQLA....LI................
tr|A9NHD4  TV.........E.A............SKP....LSHKN.K.G..WE.I.FTHQMIR....HINL..............
tr|B0VFE1  CR.........P.P...........GNRN....PESEE.RKA..CL.P.YLLEQIQ....II................
tr|B1GA08  TV.........E.R............NSA....ASHAK.R.G..WE.K.CTDTLIH....ELAM..............
tr|B1H0K1  CH............AMIDPSNPEKRSNDRP....PTLEE.MEI..CR.P.YLDMQLE....II................
tr|B1I4W5  CR.........P.P...........NNRE....PHSAE.QEA..CR.Q.LLKDELT....GLP...............
tr|B1VA82  TV.........Q.K............NKP....LSHQN.I.G..WQ.T.FTQIILE....SL.Q..............
tr|B1Y7I1  SV.........D.Q............DRA....GSHAR.L.G..WR.D.FTDAVIA....QVAA..............
tr|B1ZZ66  WR............PEMPVSASGVQYGNRP....PNGEE.MNY..CL.P.YLRAQIE....IV................
tr|B2A668  CR.........P.P...........NNRT....PNQEE.MDT..CL.P.ILRQQIR....LI................
tr|B2KCZ8  CH............PMTDPSNPEKHGNDRA....PSKEE.IAY..CR.K.YIEAQIA....LI................
tr|B3DV47  CR.........P.D.....IPKGQKGNRK....PTTEE.MST..CL.P.YLLSQLK....LI................
tr|B3U4N1  TV.........R.A............HAA....NSHRG.R.G..WE.A.VTDRIIA....VLNA..............
tr|B3U4U7  CR.........P.P...........NNRD....PEPQE.VET..CK.P.FLLQQIA....MI................
tr|B5II11  CR.........P.P...........ENRK....PTPAE.MAA..CR.P.WLDRQIQ....LN................
tr|B5LRM7  TV.........R.K............GHP....GSHVD.V.G..WS.K.IVKSVLQ....WLHD..............
tr|B5Y8I5  CR.........P.P...........NNAD....PTEDQ.KSN..CF.P.ILAAQLA....IG................
tr|B5YEZ9  CR.........P.P...........NNRE....PLPEE.IKA..CF.P.FLRRQIE....II................
tr|B6A3R0  HF.........KFT.........QRGKRRLHAKPNAGE.IKR..CA.W.WLGAEIN....IL................
tr|B6AFI4  SV.........E.E............GKP....MSHKG.L.G..WE.R.FTSAVIS....TLNSID............
tr|B6K2R2  TV.........R.A............HQA....NSHAG.K.G..WE.S.FTHAVLQ....TILKR.............
tr|B6KW10  TV.........R.E............STP....MSHRD..AG..WE.R.FTTKVIN....VINTQC............
tr|B7FQS1  SV.........R.R............GEA....NSHQK.K.G..WE.N.LTDEIVR....AVDR..............
tr|B7KM64  CR.........P.P...........NNRT....PTEKE.IET..CR.P.YLLAQIE....FI................
tr|B8C427  TV.........R.R............GEA....NSHAK.L.G..WE.E.FTDFIIN....SINE..............
tr|B8CX81  CR.........P.P...........NNRQ....PTRSE.AEA..CS.P.ILKAEIK....LI................
tr|B8EJL3  WR.........P.P...........GNRT....PTPIE.TAA..CL.P.FTRRQIE....LV................
tr|B8HFW5  SA.........F.PWHPHK.PGQPLTNRT....PRPSE.AAL..GT.P.FWHALAE....LF................
tr|B8I5K9  TR.........RIV............NRP....ATIKD.IKE..NQ.I.WLHREIH....IL................
tr|B9KXL9  CR.........P.P...........GNRD....PLPDE.IAA..CA.P.YLDEQIA....VI................
tr|B9XNJ8  CR.........P.P...........SNRT....PKTGE.IET..CT.SLYLYRQIE....LI................
tr|B9Z7Q7  TV.........E.A............DKA....GSHGK.L.G..WQ.N.VSDALID....AVN...............
tr|C0BNZ8  SV.........R.A............GEA....GSHLK...Y..WD.S.FTKSIVQ....GLNN..............
tr|C0X269  SRPFSVK.....TKQIAKTGERVTKFPNRT....PTKEE.IKI..FA.P.LLDWEIA....VC................
tr|C1A7L2  RC.........P.T.......DNGMTFRT....PTRAE.LENDLNLAR.LHAEIAR....LLPR..............
tr|C1D9B0  TV.........A.A............DQA....ASHRS.L.G..WQ.T.LTDQLVQ....AVSS..............
tr|C1F1L1  CR.........P.P...........QNRV....PEPDE.ANT..CT.Q.FLFRQMQ....IV................
tr|C1G9A4  TV.........R.A............HNA....NSHAN.Q.G..WE.K.FT....Q....KVIDIVAK..........
tr|C2MIJ3  SRPYKWREKKERN.G.EIIQKKY....NRT....PNQGE.IVA..HA.S.LLDYELE....KI................
tr|C3X2T7  CR.........A.S.......NESGKDRQ....PTEEE.AAI..CM.P.YLERQID....LI................
tr|C5CIT9  CR.........P.P...........NNRT....PTLQE.MKA..CE.P.YLLSQLA....VI................
tr|C5NVK4  TV.........E.K............NSP....ASHSK.M.G..WE.N.FTDYIIE....TISE..............
tr|C7H0A3  TV.........R.E............GKA....HSHKA.K.G..WE.R.FTDAVIS....HISK..............
tr|C7M312  CR.........P.P...........GNRT....PRPDE.VEA..CR.G.FLARQIH....LV................
tr|C8W879  CR.........P.P...........SNRN....PKVAE.VEE..CS.P.YLRDQIR....AI................
tr|C9M5Q7  CR.........P.P...........DNRT....PQKDE.LSC..CL.C.WLRRQLE....MI................
tr|C9RLM9  TV.........R.A............HMA....ASHAG.I.G..WQ.Q.FTDTIIQ....RLSQ..............
tr|D0MK10  SR.........P.P...........HNRD....PQPDE.IEA..HL.P.ILYKQIA....LV................
tr|D0PPD4  TV.........D.K............GRP....GSHHK.L.G..WG.W.FTDYVIS....TISE..............
tr|D0U4F0  TV.........E.K............NSP....GSHAK.S.G..WV.D.FTDTVID....ILNE..............
tr|D1ART8  TV.........Q.A............GKA....ASHRK.K.G..WE.T.FTDTIIK....KLND..............
tr|D1C3J5  CR.........P.T...MEGVGGRLRNRT....PTPAE.VE....WFRP.WLLRELD....VI................
tr|D1CDM9  CR.........PTA.....VTQGKITNRA....PRAGE.VSA..CE.P.WMWLEIH....LV................
tr|D1JDZ5  CR.........P.P...........GNRN....PLTDE.IRA..CT.P.FLDAQLE....LI................
tr|D1PN05  TV.........E.Q............GAA....FSHAG..HG..WE.T.FTRAALA....YVAELDS...........
tr|D1Y5H9  CR.........P.P...........KNRN....PLKTE.IAA..CL.P.WLTDQFE....AI................
tr|D2EF83  CR.........P.P...........NNRK....PKKEE.LDK..CR.S.YLVRQLS....SI................
tr|D2Z573  CR.........P.P...........GNRN....PKKGE.IRA..CL.Q.WLEDIVG....LL................
tr|D3B454  TV.........T.Q............AKP....NSHQP.F.G..WS.A.FTDAIMK....VINE..............
tr|D3E6H2  VR.........PSG.....ENVISRSNRT....PTKAE.ILA..HA.P.LLDEEIR....WV................
tr|D3FBP7  CR.........P.P...........GNRD....PRPTE.IAT..CR.D.YLHRQVE....LI................
tr|D3QZ35  TV.........R.E............NQP....MSHAG.H.G..WE.T.FTSHILA....HLFS..............
tr|D4G8B4  TV.........E.R............GKP....LSHKN.I.G..WQ.I.FTNKAIS....KISL..............
tr|D5C4I1  TH.........P.S...........GNRK....PYRGE.IKK..EL.P.FLLRQIE....LL................
tr|D5E730  CR.........P.P...........NNRR....PRKEE.LAA..CR.A.FFEQQIE....ML................
tr|D5EGK6  CR.........P.P...........ENRK....PTKDE.LDA..CR.P.FLEEQIR....LL................
tr|D5EHP1  WR.........PQH.......DKPYGNRP....PTEDE.MNF..CL.P.YLKAQLE....II................
tr|D5MIW4  CR.........P.P...........ANRN....PEPDE.IAA..CE.P.FLVAQLQ....AI................
tr|D5RKP0  TV.........E.E............GKA....GSHAR.A.G..WH.G.FTDAVLR....AVDA..............
tr|D5U040  CR.........P.P...........GNRK....PRRDE.ISQ..CL.G.YLGRQLR....LI................
```

```
tr|D5U570  CR..........P.P...........ENRD....PLPEE.KAN..CR.D.FFVRQLQ....IL.................
tr|D5V6V9  TV..........E.E............SKP....KSHHK.L.G..WE.I.FTDNIIK....HISA...............
tr|D5WWR6  CR..........P.P...........GNRK....PAPEE.MVA..CR.P.YLMAQFA....VL.................
tr|D7CKV6  CR..........P.P...........GNRL....PNPDE.VAA..CR.G.WLEAQIR....LM.................
tr|D7CWJ9  YR..........P.P...........GNRN....PKPEE.IEA..SA.P.LLLEQLR....LI.................
tr|D7UXZ6  SRPYKERQKWDRKTE.....RNEWKRYNRT....PNKGE.IIA..HA.P.VLDYEIE....TS.................
tr|D8LFW4  SV..........E.R............SKA....NSHKN.Q.G..WE.K.FTDAVVR....ELNK...............
tr|D8UI66  TV..........R.Q............GEA....NSHAK.R.G..WE.Q.FTDAAIR....ALSE...............
tr|D9Q1V3  CR..........P.P...........NNRT....PTEDE.VAA..CI.G.FLKEEIS....LV.................
tr|D9QQQ4  CR..........P.P...........GNRN....PKFSE.MKS..CL.W.FLAQEIK....LI.................
tr|D9S3H2  CR..........P.P...........MNRT....PQSKE.IKA..CK.R.ILELELE....FI.................
tr|D9SCW5  CR..........PLD............DRH....PHVGE.IAA..CL.P.YLKRQIG....LL.................
tr|D9Y2E5  TV..........V.E............GTA....NSHSK.K.G..WE.E.LTDEIIA....KVAS...............
tr|E0S9I8  TV..........T.K............NQA....NSHAG.I.G..WR.E.FTSRILQ....IINE...............
tr|E0SQC9  CR..........P.P...........NNRE....PSDEE.IEK..CG.I.YLETQIR....LI.................
tr|E0TBI7  WR..........P.P...........GNRA....PTGEE.QAI..CL.P.FLRRFIT....LA.................
tr|E0TIS2  CI..........S.K...........NIKNKNIFPNKKE.ILK..CL.F.YLKKEIK....II.................
tr|E1IBI3  LH..........PHR......PGNDQSNRP....PRSTE.LAL..GL.P.LLAEVCT....LF.................
tr|E1L0U9  SV..........E.E............HKP....NSHVE.L.G..WN.V.VTQEILR....VALM...............
tr|E1QFX8  CR..........P.P...........NNRD....PRPEE.VAA..CR.P.WLEAQAR....AV.................
tr|E1QNN8  CR..........P.P...........NNRD....PEPDE.IEA..CR.P.YLITQIL....MI.................
tr|E1QWE2  TV..........P.C............GKA....NGHAR.L.G..WL.D.VTSAILDCALTRTS................
tr|E1R614  CR..........P.P...........GNRD....PLPDE.AAA..CR.P.FLDRQID....IL.................
tr|E2IUG8  TV..........R.R............GAA....GSHSG.L.G..WG.R.FVAGVLR....RLAQ...............
tr|E2SYG7  CN..........P.K...........DERGNNATPTSTE.IAN..CA.P.FLRETVE....IL.................
tr|E3D056  CR..........P.P...........GNRV....PTFEE.MMA..CD.E.YLQTQIA....LG.................
tr|E3IP47  TV..........E.A............GQA....HSHAR.L.G..WQ.E.VTAAVVE....ALG................
tr|E4KQ36  TV..........K.E............GQA....NSMKD.V.G..WL.E.FTQAIIQ....LLGE...............
tr|E4RKG8  CR..........P.P...........DNRV....PYKKE.VKA..CS.A.ILKAEIE....LI.................
tr|E6N689  CR..........P.P...........ENRE....PTPEE.RQT..CTNK.YLFRQIE....AV.................
tr|E6PFU2  CR..........PTS.....QGERGLKNRA....PDPTE.MAN..CR.P.YLDEQIE....II.................
tr|E6W1H1  TV..........E.A............GKA....NSHRN.K.G..WE.R.FSEGVLR....ALS................
tr|E6W552  CR..........P.P...........GNRN....PEPGE.ISI..CQ.Q.FLQRQIR....LV.................
tr|E7A9L4  SV..........E.K............NKA....KSHAH.L.G..WE.S.FTDLILV....QLSN...............
tr|E7FX98  TV..........R.E............GEP....MSHGQ.L.G..WE.Q.FTDRVIS....ELGC...............
tr|E7H418  CR..........P.P...........RNRN....PQPEE.IVC..CG.H.WLARQLE....LI.................
tr|E7H4Y1  TV..........E.A............GKP....LAHAG.W.G..WE.T.LTDRLVA....QLSS...............
tr|E8LLT1  TV..........K.A............GEA....FSHHN.Q.G..WE.I.FTDGVIK....AIND...............
tr|E8N338  CR..........P.P...........GNRD....PQPEE.LEA..CN.A.YLERQIE....AI.................
tr|E8T398  CR..........P.P...........NNRD....PAPDE.IEA..CY.P.FLEKQIE....LI.................
tr|F0EKF3  SRPYSVKERINKR.S....GAHEIVHPNRT....PTKKE.VLL..HA.P.LLDYELS....IV.................
tr|F0NSC6  SRPFSVKKVFSKREN.....KEVIKRPNRK....PTKKE.VLA..HA.P.FLDYEIS....YA.................
tr|F0RSJ4  CR..........P.P...........ENRD....PEVDE.VQA..CL.P.YLKRQIQ....LV.................
tr|F0SG07  CR..........P.P...........GNRN....PTPIE.ASH..CR.E.YLDGQIE....TV.................
tr|F0Y985  TV..........R.E............GEA....HSHRG.R.G..WE.R.LTDAVVA....ALAA...............
tr|F2LXL6  CR..........P.P...........NNRD....PLPEE.LVA..CF.G.YLKQQIS....LI.................
tr|F1A0X7  TV..........E.E............STP....NSHKD.F.G..WY.Q.FTDAVLK....ILNE...............
tr|F2NFM2  CR..........P.P...........NNRT....PEADE.IAA..CR.P.FLWKQLQ....AI.................
tr|F3KRD3  TV..........E.D............SEP....ASHAG.L.G..WE.V.LTQRIIL....AVTE...............
tr|F3QMU0  CR..........P.P...........LNAT....PEKAE.TMA..CR.P.FLERQIE....LI.................
tr|F4A8X0  FR..........PIKKKVGKTGRTTISNRP....PKVSE.VNL..FR.D.ILDDEIA....VV.................
tr|F4C0J7  CR..........P.P...........QNRK....PNKKE.IEL..CL.P.YLEDQMD....AI.................
tr|F4GMI7  CR..........PLG............NRT....PTQDE.IEA..CM.P.YLQRQIA....LL.................
tr|F4GSY3  TV..........E.A............HKP....ASHAK.R.G..WE.I.ITDAIIE....RVLR...............
tr|F4HNK0  CR..........P.P...........NNRD....PTEEE.IKA..CS.P.YLDAQID....II.................
tr|F4NZD5  TV..........R.Q............SEP....NSHAK.C.G..WL.K.FTDAIIA....YINK...............
tr|F4QWL4  WK..........T.P...........GNRT....PTPQE.QAV..CA.P.FLERAAA....LL.................
tr|F5SL17  CR..........P.P...........GNRT....PQDEE.MAT..CI.T.ILREQFV....AI.................
tr|F5Y215  CR..........P.P...........GNRN....PEPDE.VAQ..CE.P.FLRRQVE....LL.................
tr|F5YGI3  CR..........P.P...........DNRD....PLVEE.TES..CA.P.FLARQIR....LL.................
tr|F7NHV1  CR..........P.K...........GNRT....PTVDE...GSFCATR.WLDNELA....AV.................
tr|F7PTA1  TV..........K.Q............NLP....GSHRE.I.G..WE.L.FTNQVIS....ELNK...............
tr|F7S094  TV..........R.E............SVA....GSHAK.L.G..WQ.Q.FTQNALR....YLAQ...............
tr|F7XVS9  WR..........P.P...........GNRR....PTPEE.IDA..CR.P.FLEKMIF....LL.................
tr|F8ADN2  CR..........P.P...........GNRT....PQPDE.IEA..CL.P.YLTKQIK....LI.................
tr|F8L559  TV..........Q.A............GNP....KSHYG.R.G..WE.T.FTDAVIW....RLCQ...............
tr|F9CXI5  CR..........P.P...........KNRV....PSISE.RDA..CD.N.YLQKEIA....II.................
tr|F9DTW2  SR..........PYKWGTKKERDGSLTKRKYNRPPTQKE.QLA..HA.P.VLDYEVA....NL.................
tr|F9LK36  SR..........PYN........SKNKNRK....PNKKE.IFA..QA.P.LIDWEIK....KV.................
tr|F9ZLD4  CR..........P.E...........GNRR....PTAEE.IWN..CG.D.FLLETLE....RS.................
tr|G0LJW8  CR..........P.P...........ENRN....PTTEE.LSN..CR.R.YLEHELS....AL.................
tr|G0QBL8  VR..........P.P...........DNRD....PRREE.IET..WS.P.LLERELN....LV.................
tr|G0QFK7  IR..........P.P...........DNRD....PKKKE.IQA..WK.P.LLRKEIR....DV.................
tr|G0U4E4  TV..........E.A............HKA....NSHSA.C.G..WQ.A.FTDGVIR....LLSK...............
tr|G2RZ19  TV..........A.A............HQP....GSHNN.L.G..WQ.K.ITKIILE....NVVL...............
tr|G4RL69  CR..........P.P...........NNRE....PLEDE.IKA..CL.P.YLVRQIK....AI.................
tr|G4STH7  CR..........P.P...........NNRD....PLTTE.VEA..CR.D.YLHRQIA....WL.................
tr|G4T6H9  TV..........R.A............SEA....GSHSK.I.G..WD.K.FTRAVME....AVLAR..............
tr|G7WNS9  CR..........P.P...........ENRR....PKRKE.VEA..CR.I.YLDAQIE....LL.................
tr|G8BJ42  TV..........E.A............HKA....NSHAN.R.G..WE.Q.FTEQVIR....VALEYHQK...........
tr|G8LWR8  HRLSKIN.....PDT........GRVSNRP....ATKEE.IEQ..NR.E.YLIEEIA....II.................
tr|G8TXV8  CR..........P.T.......DDGRKNRP....PLPDE.VIA..CR.P.WLMDDVR....TV.................
tr|G8XT11  TV..........V.R............NKP....GSHRH.L.G..WQ.Q.LSERIIR....QLSE...............
tr|G9EIR0  CR..........P.P...........NNRD....PQAAE.IRQ..CS.A.YLSRQIE....LL.................
tr|G9XY09  SV..........V.A............KTP....LSHKD.I.G..WT.N.FSQNLII....YLNE...............
tr|H0E6E4  CR..........P.P...........DNRD....PLPEE.IDH..CR.G.YLLETLA....LV.................
tr|H0KUY0  TV..........Q.A............HQP....NSHKD.L.G..WE.K.FTDYVIR....QISE...............
tr|H0Q5N1  SV..........E.K............DKP....KSHHK.L.G..WD.L.VTGALLR....ALS................
tr|H1D376  CR..........P.R...........GNRT....PTMEE...GRFCG.SIWLAAEIA....LV.................
tr|H1P1U4  C...........P.T.......SDGHKFRA....PSNTE.LRN.....PENLTRIQE....EIQTAAHRCE.........
tr|H1P505  CR..........P.P...........DNRT....PTPQE.AQA..CL.R.YLRKQIE....LI.................
tr|H1XWX4  CR..........P.P...........DNRD....PRPEE.IAR..CE.P.YLIKQIQ....MI.................
tr|H2C8Z4  CR..........P.P...........NNRD....PEEDE.IRA..CS.P.YLLIQLE....MI.................
tr|H2J7L7  CR..........P.P...........KNRV....PTGEE.MEK..CS.H.FLLAQVM....TI.................
tr|H3KDP1  TV..........E.E............GMP....LSHRR.F.G..WT.A.LTDGLIR....ALSD...............
tr|H3NN78  TV..........K.D............SKP....KSYAN.T.Y..WN.K.FTDKVIS....RVSK...............
tr|H3NSX2  TV..........S.E............GQP....ESHRN.R.G..WE.A.ITDCIIK....AIVA...............
tr|H3RRL7  RRPYSIK.....RVKDKKTGAIVEKTPNRT....PTKAE.VYA..YA.R.LFDWELD....QV.................
tr|H3SCZ2  VR..........P.P...........GNRK....PKTSE.VTE..AL.P.ILLREIE....LI.................
tr|H4GIS2  SRPYSVK.....QVKDRRTGKIVTKYPNRT....PSNKE.VRI..YA.P.LFDWELH....QV.................
tr|H5SI33  CR..........P.P...........GNRE....PHPDE.VAA..CL.P.YLVRQMS....VL.................
tr|H6RLZ5  CR..........P.P...........GNRT....PRADE.VGR..CS.G.WLRRQLQ....LL.................
tr|H7F338  SRPYREEWKIDAR.S....GEKVLKRYNRP....PTQKE.ILA..HA.P.ILDDELR....HV.................
```

```
tr|H8I7F5  CR..........P.P...........GNRP....PRACE.IEA..CH.P.YLQRQLE....AI.................
tr|H8ZDF2  TV..........T.Q............KKP....RSHSS.I.G..WM.S.FTDTIIK....KVSD...............
tr|H9UI22  CR..........P.P...........ANRN....PAAEE.TAA..CD.G.YLQRQLD....AL.................
tr|H9ZZZ0  CR..........P.P...........KNRK....PKTNE.VLS..CL.H.YLIFQLM....YI.................
tr|I0GI60  CR..........P.P...........NNRV....PTPNE.IEA..CV.P.YLYAQIS....VI.................
tr|I0I6B7  CR..........P.P...........GNRD....PLPDE.LAA..CE.D.YLLRQIE....MI.................
tr|I1A005  TV..........E.E............GKP....NSHKD.I.G..WE.K.FVEIVIQ....DIIL...............
tr|I1B0L2  SV..........P.K............GKP....KAHSR.L.G..WT.E.LTRQVL.......ETL..............
tr|I2K0D9  TV..........R.A............HXA....NSHSN.H.G..WE.R.FTSAAIR....KLIEYKNH...........
tr|I3CUN9  CR..........PTD........DNGRDRP....PSPQE.VAA..CL.P.YLQRQIE....LI.................
tr|I3VQA4  TV..........I.K............GKP....GSHET.L.G..WQ.L.LCDKVIA....SVSK...............
tr|I4CBR6  CR..........P.P...........ANRD....PEQDE.IDT..CL.P.FLKSQIR....AI.................
tr|I7LIA9  FR..........PTK...KDAKTQRLSNRA....PNKKE.IEL..SK.E.FLIKEIE....FI.................
tr|I7LKX1  HR..........P.P...........GNRD....PRDEE.IRT..CT.P.YLEEQIR....II.................
tr|J0UVA7  CR..........P.L...........GNRP....PAPEE.LAA..CR.A.FLDAQIR....LV.................
tr|J5SPB4  TV..........R.A............SEA....GSHAG.K.G..WE.Q.FTAAVMK....VVTSRLAGSASGVAVTATA
tr|J7R1V5  TV..........R.A............HSA....NSHSK..QG..WE.T.FTSRIVE....LLIQDREKSG.........
tr|J9E0C3  WR..........P.T...........GNRT....PTPDE.TAL..CR.P.FLLRHIE....LI.................
tr|J9Z1B8  TV..........E.R............SKP....LSHQK.L.G..WD.I.FTNKVIS....TISK...............
tr|K0IF13  CR..........P.P...........NNRV....PEEDE.VTA..CR.P.YLDRQIA....LI.................
tr|K0KF39  TV..........K.A............HEA....NSHAK.K.G..WE.Q.FTEVVLK....KAIS...............
tr|K1JXC9  CR..........P.P...........GNRN....PAPAE.VAC..CA.T.YLRRQLL....LV.................
tr|K1JYF3  TV..........R.G............GEA....ASHRS.L.G..WE.M.LTDSIIQ....AVSE...............
tr|K1LNP2  TV..........A.K............GQA....NSHQG.L.G..WK.A.LTRAIVQ....SLN................
tr|K1ZRE1  DR..........P.P...........NNRD....PEPEE.IKL..YS.P.FLDRQIL....II.................
tr|K2A3B6  HR..........P.P...........GNRD....PEPSE.IAA..YG.R.YLDEMIE....VI.................
tr|K2B4F4  CR..........P.P...........GNRD....PFPEE.IEA..CW.P.YLEAQIR....II.................
tr|K2C359  CR..........P.P...........NNRV....PQTAE.ADA..CK.K.HLFKQIA....LI.................
tr|K2CLQ5  YL..........P.............DAGT....PSPSD.IAH..GR.T.HLMKQFE....II.................
tr|K2DEQ6  HR..........P.P...........ENRD....PFPDE.IEA..CQ.P.YLNMQLK....II.................
tr|K2E7J1  TV..........S.A............GNP....ASHKD.K.G..WE.I.FTDAVIK....SLNE...............
tr|K2E9B4  CR..........P.P...........ENRV....PTDGE.KAA..CR.V.YLDAQIQ....LI.................
tr|K2ETW7  CR..........P.P...........GNRD....PQPEE.VKL..CT.P.YLQQQIA....LI.................
tr|K2F640  HR..........P.P...........ENRD....PEPHE.IEA..YR.K.YLDKIIK....II.................
tr|K4KIT8  TV..........E.Q............GQA....ASHAG.K.G..WE.Q.FTAACLQ....AVNR...............
tr|K6GHM9  TV..........E.K............GLP....NSHKG.I.G..WE.K.LTDKIIS....VLSE...............
tr|K7YPM8  WH..........P.P...........GNRQ....PTSDE.ISV..CI.P.FVERHIE....LA.................
tr|K8ZAC1  TV..........R.K............GEA....NSHQQ.K.G..WE.T.FTDHVLE....ALNQ...............
tr|K9VTI9  CR..........P.P...........DNRV....PTNKE.IAA..CK.P.YLLEQIR....LI.................
tr|L0A9A2  CR..........P.P...........NNRT....PNKEE.VSS..CL.P.YLIEEIN....IV.................
tr|L0B4R8  TV..........E.H............GKA....MSHAN.K.G..WE.L.ITDTIIK....LIAT...............
tr|L0B866  TV..........E.E............GKP....LSHKK.I.G..WE.N.ITDSIIK....IVSE...............
tr|L0B8A6  CR..........P.P...........GNRN....PRPEE.IIS..CR.P.YLIEQID....II.................
tr|L0DFI2  SR..........P.P...........ENRD....PAPDE.IAH..CL.P.YLERQIE....IV.................
tr|L0L178  CR..........P.P...........NNRK....PTHSE.LKT..CR.P.FLLAQIE....LL.................
tr|L2GQC8  TV..........E.K............NKP....ASHSE.I.G..WK.R.FTKRILE....LINT...............
tr|L2GX11  TV..........S.E............AKP....GSHST.Y.G..WS.I.FTDKILK....LVND...............
tr|L5N8A0  SR..........PYKWVDSNKKGKRKANRK....PNKKE.IEA..HA.P.LLDYQVE....QV.................
tr|L8HA23  TV..........E.A............RKP....NSHKD.K.G..WE.R.FTDAIIK....AVND...............
tr|L9X1W2  VR..........P.P...........ENRD....PRVAE.IEA..WW.P.VFEAEIE....CV.................
tr|M0BKB2  CMPADETRGAAAEPDTDSASESADSVSTRE....PAAEE.LAA..CR.P.HLLTELD....RV.................
tr|M0CBA6  AR..........P.P...........ENRD....PTADE.RRA..WK.P.LLDAELD....RV.................
tr|M0MGS7  CF..........P.E.......GGDGSNRE....PTSEE.RTN..CR.P.YLETEIE....EI.................
tr|M1E4Q4  CR..........P.P...........GNRN....PLPEE.IEQ..CR.P.FLDAQIK....IL.................
tr|M1LNF1  TV..........E.D............SKP....MSHKN.K.G..WE.L.FTDAIIK....AVAN...............
tr|M1QKH3  CR..........P.P...........DNRT....PEEQE.MIT..CS.Y.ILKEEIK....LI.................
tr|M1VCA8  TV..........E.A............HKP....GSHAK.HGG..WQ.K.FTDEVIR....VLDQ...............
tr|M1Z9H8  YR..........PTK...TNLKTKRLSNRT....PTNKE.IDA..FR.P.YIYREIN....II.................
tr|M2XVU5  TV..........E.A............HKP....ASHSN.R.G..WE.Y.FTNEVIK....LLNV...............
tr|M3EFG6  SR..........P..............YKW....GTKKK.RNG.....E.IIERKYN.......................
tr|M4YU22  CR..........P.P...........QNRE....PQPEE.MAA..CR.P.FLDSELA.......................
tr|M5ADY8  TV..........R.K............GFP....LSHKN.I.G..WE.L.FTDQIIQ....TISD...............
tr|M5IVB6  TV..........E.D............GLP....ASHAK.K.G..WE.Q.ITDSLLM....NLAQ...............
tr|M6D8H9  CR..........P.T.....VDLKFEKDRP....PEEDE.VIA..CS.P.FLLRQIS....II.................
tr|M7WR77  TV..........S.P............HKA....GSHSN.R.G..WE.D.FTDKVIE....LVDKHAGASEDGK......
tr|N0BBN4  CR..........P.P...........NNRD....PLEEE.IKA..CK.P.YLIRQLK....SI.................
tr|N1V4N6  SA..........Y.PFHP.HQTGRPLSNRT....PNAKE.IQA..GL.P.LWQELAR....IF.................
tr|N1Z687  FR..........PTN...VSPKTGNFVNRP....PKKTE.VDF..FL.P.YLKKELE....II.................
tr|N2BLL9  SV..........Q.K............GTA....GSHKH.F.G..WE.T.FTDSVIY....ALST...............
tr|N9SHT1  SV..........R.K............SSP....NSHAN.I.G..WE.I.FSYNLIK....FIVE...............
tr|N9V498  TV..........E.E............FKA....NSHKD.I.G..WR.Q.FVFKLIE....FIANIN.............
tr|Q025I0  CR..........P.P...........GNRT....PEPEE.MEI..CG.E.FLYRQLS....TI.................
tr|Q0C0Y4  WR..........P.P...........GNRS....PEADE.LAV..CR.P.FVDRFIE....VS.................
tr|Q0EZR7  CR..........P.P...........NNRD....PRADE.VKA..CN.L.WFEQQLD....AL.................
tr|Q12TN4  CR..........P.P...........NNRN....PNKKE.LDC..CK.P.FLIRQIQ....LL.................
tr|Q1AV32  VR..........PTK.....ESGGRIQNRP....PRAGEVREG..LE...VLEEELR....AI.................
tr|Q1AYQ1  CR..........A.A....VEENGRLRNRQ....PRAAE.INA..CN.P.YLQAQIE....AV.................
tr|Q1GY28  TT..........R.............GGVA....GAHSK.I....WQ.A.FTHAVMR....YLGQRE.............
tr|Q1N1I3  TV..........R.A............GQA....GSHSK.Q.G..WQ.R.FTNSCIQ....LINE...............
tr|Q23W06  TV..........E.Y............DKP....ASHSK.S.G..WA.D.FTSEVIK....IINK...............
tr|Q3SA81  CR..........P.P...........NNRD....PTDEE.IRA..CA.P.YLEWQIE....HM.................
tr|Q53CZ3  TV..........E.R............GKA....GSHSN.L.G..WD.W.FTSYIIS....CLSA...............
tr|Q5DC15  TV..........R.A............SMP....NSHKD.K.G..WE.F.ITDAAIR....YLNK...............
tr|Q5WES9  SRPYKHDVKSSKK.P.......IEERGNRT....PTQKE.IIA..HA.P.LLDEMIN....QL.................
tr|Q6CAC0  TV..........E.E............KKA....NAHKD.K.G..WE.A.VTFTALS....AAVK...............
tr|Q6F1M3  TV..........I.E............NQP....GSHSK.L.G..WQ.E.IVSDILV....NLNK...............
tr|Q6KIL8  TT..........E.L............EKA....RAHSF.L.P..WK.I.FTMNLIE....YINT...............
tr|Q7VBS6  SR..........P.P...........NNRT....PTRQE.IQN..HI.P.WLYQQIK....LV.................
tr|Q8TK31  CR..........P.P...........ENRR....PKASE.IEC..CK.P.FLIAQIT....LL.................
tr|Q9RTK9  CR..........A.P...........NNRT....PLPLE.TAT..CT.GLWLEPQLA....LL.................
tr|Q9WR44  TV..........K.R............GVP....GSHFY.M.G..WD.M.LIKSMLK....TLCL...............
tr|R1ATM3  FR..........PTR...ISKKTGRKINRA....PSKKA.IES..FK.E.YLLTEID....II.................
tr|R1E4B2  CR..........P.P...........ENRE....PEEEE.IKA..CS.I.WLEKQLE....II.................
tr|R1FRL1  TV..........R.R............GEA....NSHAK.L.G..WQ.D.FTDAVIR....VLDA...............
tr|R1GL12  WR..........P.P...........FNRQ....PSPLE.IEM..CL.P.FVDRHIQ....II.................
tr|R2SFV7  SRPYRIQQGINRKTG.....VAETKYPNRT....PTKKE.VLA..HA.P.ITDYELQ....TI.................
tr|R2SXC9  SRPFSIKKRLNKKTG.....IIETKYPNRT....PNKTE.VLA..YA.P.LLDYEIQ....TI.................
tr|R4KRR4  CR..........PTQ.......NDGRSNRK....PRACE.IKH..CA.H.WLDEELA....LL.................
tr|R4VE87  CH..........P.P...........GDRD....PRRGE.VQA..CQ.G.WLEAQIR....LL.................
tr|R5ARL3  YR..........PIV.....RGERSTRNRT....PSRKE.IEA..SL.P.LLREEIR....SI.................
tr|R5B4U8  FR..........PIK.....RGARSVANRT....PTAKE.LAS..GE.E.LLKSEII....AV.................
tr|R5DX78  CR..........P.P...........KNRV....PSDEE.KAS..CR.K.FLNAQID....II.................
tr|R5MPG0  TV..........E.K............DKP....LSHSY.L.N..WE.K.FTDVVIE....KLSS...............
```

```
tr|R5PZ23  WH..........P.P...........ANRK....PSEEE.VSL..FL.P.FLKRHIA....LV.................
tr|R5QX00  WR..........P.P...........GNRT....PLDSE.IAV..CL.P.FLKRQLD....II.................
tr|R5Z1D7  TV..........E.Q............SKP....LSHQN.I.G..WQ.N.FTNEAIR....WINK...............
tr|R6HGV3  TV..........R.E............HAA....NSHAK.L.G..WQ.V.LTNHVIE....RCF................
tr|R6HL97  SV..........R.A............GMP....KSHSA.L.G..WQ.T.FTDSIIL....ALNK...............
tr|R6PFR8  CR..........P.P...........QNRV....PSKEE.KLA..CQ.G.YLMGQIS....IV.................
tr|R6PI86  CR..........P.P...........ENRN....PLPEE.KAA..CR.E.YLDIQVG....AL.................
tr|R6R865  TV..........R.K............NEP....MSHQN.Y.G..YE.T.LIENVIK....VLDN...............
tr|R7BZ83  TV..........E.E............GEA....ASHGR.W.G..WE.K.LTDAVLA....KTAS...............
tr|R7DQ64  SV..........R.R............GIA....LSHQI.P.E..YE.Q.LLKDVLL....YLDQ...............
tr|R7DXQ1  FR..........P.A.....LPNQRTNNRA....PTPEE.IEA..CL.P.IIMNEIQ....VI.................
tr|R7I8P1  CR..........P.P...........QNRV....PTDEE.AAA..CK.L.HLRIQTA....LI.................
tr|R7IAS2  CR..........P.P...........HNRD....PKPEE.VAV..CR.Q.FLDRQLE....LL.................
tr|R7IHQ8  IR..........PTK....VSEKGRVSNRT....PNREE.LAL..FT.P.YLLEEIL....LV.................
tr|R9ADN8  TV..........R.K............DTA....GSHGN.K.G..WE.Q.FTTRVLQ....VLADRH.............
tr|R9LKN0  TV..........R.E............HQP....LSHQK.I.G..WQ.L.FTNEILR....TLND...............
tr|S0EV15  CR..........PFT.....TESGAARNRP....PTQEE.INA..CR.P.WLEQTID....II.................
tr|S0FG57  TK..........RVI............NRP....ATRED.IVN..NQ.H.WLHQEIA....LL.................
tr|S2J4F7  SV..........E.A............HKA....GSHAN.Q.G..WE.Q.FTDAIIK....YLNE...............
tr|S3JT40  CR..........P.P...........QNRD....PAPEE.RSC..CA.A.FLDAQIR....LL.................
tr|S4NBY8  SR..........PFS....IGKSGRKSDRR....PNQAE.VKK..SA.P.MFDWELA....HF.................
tr|S5MFT9  TV..........E.K............GLP....NSHKN.L.G..WN.N.VIIKILK....AVNE...............
tr|S5RQ34  TV..........C.A............GKA....GSHSK.K.G..WE.F.FTDNVIK....CLSN...............
tr|S6G4W1  WR..........P.P...........GNRK....PTDFE.LEV..CR.P.FVEKHIS....LV.................
tr|S9VE50  TV..........T.A............HEA....NSHAK.C.G..WQ.Q.FTDEVIV....RVSA...............
tr|T0AYQ1  TV..........E.A............HKA....YSHAK.K.G..WE.E.FTDKIIT....CISN...............
tr|T0DRC9  SV..........E.K............TQA....ASHKR.I.G..WE.A.FSDQILM....RLFE...............
tr|T0LSQ5  CR..........P.P...........DNEK....PKLEY.MKI..CN.G.YLRYEIE....LL.................
tr|T0MGQ8  TV..........E.K............NKP....GSHSN.Y.G..WQ.F.FTKSIIE....KINL...............
```

```
                                    160          170
                                      .            .
sp|P12295  .....H.R....EGVVFLLWGSHA......QK.K................GAII.............D..KQ........
tr|Q8KLI8  ............APQIIVPLGAVAAEFFLGEKVS................ITKV.............R..GKW.......
sp|A1QYK3  .....N.L....NNVVFMLWGNFA......RG.K................KELI...........D.A..SK........
sp|A1SUE5  .....A.R....SGVIFLLWGSYA......HK.K................GSLI.............D..KN........
sp|B0B8I2  .....N.R....THVIFVLWGNAA......RQ.K................CNLL..........FQTK..HQ........
sp|B1AJI8  .....Q.K....H.VVYLLWGKQA......MS.Y................INLI...........D.Q..KQ........
sp|P12888  .....R.L....KACVFMLWGAKA......GD.K................ASLI............NS..KK........
sp|Q057V4  .....I.C....KGVVFLLWGNIS......QK.K................YYLI...........D.S..KK........
sp|Q315T1  .....RCP.....AVVFMLWGNHA......RQ.K................AALV............DT..GR........
tr|A0LFG4  ............RPRVICALGACA....AQTL.VGTGSS...........ISRL.............R..GSV.......
tr|A0LH32  ............RPGLAVCLGLSPAKALLNDPHLK...............MGRA.............N..GAL.......
tr|A0LRS0  ............DPGVVVALGGTAVAWFFERPVT................LAQV.............R..GRV.......
tr|A0RV64  ............NPRIVCILGATALRSLLGLGG.................ITGI.............R..GTI.......
tr|A1BDC5  ............NPKLILLLGKVAANTILQNSLS................MGAM.............R..GRI.......
tr|A1RYC0  ............KPRLIVTLGKHSTKTILRMRGMSVDS............IMSV.............R..GKA.......
tr|A1US05  ............NPRILVALGGTAVQFFTGTQSG................IVRI.............R..GKW.......
tr|A4GHT2  .....K.K.....NIVFLLWGAKA......QR.K................GLMI.............D..QN........
tr|A4HXS3  .....H.HP...NRLVFLLWGGYA......QQ.K................KKLI.............D..AS........
tr|A5KS51  ............GPEVIVTLGRHSMEFFLPGQ.K................ISVV.............H..GDP.......
tr|A5UQ47  ............KPRLLVPVGRIALQEVGKRYLEGAPGAIRTLH......ATVL.............R..GRE.VT....
tr|A6DBB2  ............KPSLILLLGNSSIKAFSYLVPE................VKNL.............KKCGFY.......
tr|A6DK00  .....Q.S....EHLVFVLWGAHA......QS.H................KDLL............DA..QK........
tr|A6GN76  ............RPQVLLLVGRFAIQTLLNTKKP................VGEL.............R..GTV.HRVIVD
tr|A7ANQ0  .....H.R....ENVVFLLWGKSA......EQ.K................CSKI.............S..TT........
tr|A8A982  ............RPKLVVALGRTAAEALLGLKGP................LKGV.............R..GKV.......
tr|A8E1M5  .....Q.R....EGLVFLLWGLQA......QK.K................EYLI............DP..RK........
tr|A8F067  ............NPKLIILVGSTA....ATSL.LGKNSG...........ITKI.............R..QEY.......
tr|A8MA01  ............RPRKIITLGRHSTRVILSLNGLGFSE............LAKV.............R..GKV.......
tr|A8SNH5  .....F.D....YPKVFILWGSFA......IS.K................SNLI............KR..DE........
tr|A8UAT5  ............QAPYILTMGNIG....LQRL.LGPKAK...........VSAQ.............H..GQL.YKGPVL
tr|A8UU74  ............KPKVICCLGATAGEGILGKKLS................ITKV.............R..GKN.......
tr|A9AZ06  ............APKLIATLGRHSMNKFFPGGKITKIHG...........IRGV.............KRQGQTV......
tr|A9BHU0  ............KPKIIVTLGNTPLYYFTGES.K................ITQS.............R..GRF.......
tr|A9F5H8  ............KPKVIVALGATAVQGLIGTSEG................ITKL.............R..GTW.......
tr|A9NHD4  .....L.N....QPIVFILWGNQA......KD.I................KPLL...........T.N..PK........
tr|B0VFE1  ............QPKILLILGLVAAQTLLNNNNT................LGWH.............R..GKI.......
tr|B1GA08  .....R.H....EHLVFMLWGAHA......QA.K................RALF.............G..GK........
tr|B1H0K1  ............KPKIIVTLGASSTKGLLNSIES................ISRI.............R..GSV.......
tr|B1I4W5  ............SGVVVLTLGNVPLRFMRGTSGPG...............VTAC.............R..GVW.......
tr|B1VA82  .....T.K....KNVVYLLWGKFA......QT.Y................EKYI...........I.S..QN........
tr|B1Y7I1  .....D.P....SPKVFMLWGAHA......QA.K................RALI............NA..SG........
tr|B1ZZ66  ............NPEVLVALGSTAAQGLLGIGSFKA..............LGEV.............R..GRW.......
tr|B2A668  ............QSKIIVILGGAALKGLIDKNMK................ITKA.............R..GSW.......
tr|B2KCZ8  ............QPEFIVALGGVAAKSLIKDADS................LGAL.............R..NKV.......
tr|B3DV47  ............NPKVIVALGATAYEGLTGKVGIK...............ISEV.............R..GRC.......
tr|B3U4N1  .......KP...SRVVFVLWGVEA......KK.K................QALI.............T..AA........
tr|B3U4U7  ............RPKLVCSLGNWATQTLLERK.VG...............ITRV.............R..GQA.......
tr|B5II11  ............DPAVILLAGATAMEGVLGVKGG................ITRL.............R..GQW.......
tr|B5LRM7  .....N.K....EGLVFMLWGVHA......Q..E................AFKP............NC..RK........
tr|B5Y8I5  ............KPKYILALGRHAAEAFVPKPFRS...............IKEI.............R..GQV.......
tr|B5YEZ9  ............NPKVICTLGRYAAYAILGHSVN................ISNS.............H..GKD.......
tr|B6A3R0  ............KPKLVVTLGASALYALLGPKVK................LTPE.............R..GHI.......
tr|B6AFI4  ............RKIVFLLWGKSA......QTKA................ENVC.............K..NK........
tr|B6K2R2  .....H.P....KGLVFLAWGTPA......AN.R................LKNL...........SLA............
tr|B6KW10  ............EGVVFLLWGKPA....QKKS.AG...............VSRC.............R............
tr|B7FQS1  .....Q.SKKSGQRVVFLLWGKPA......TA.K................AQAL........IGSNSS..VN........
tr|B7KM64  ............NPKFIVLAGGSAYRGLTGKR.KP...............ISQV.............R..GKW.......
tr|B8C427  .....R.N....DSVVFMLWGAPA......QK.K................ACCV.............D..EA........
tr|B8CX81  ............DPLVIVPLGSVALKNLVDEKAS................IMRT.............R..GKW.......
tr|B8EJL3  ............GPKILVCLGLPSAQTLLGAKDS................MTRI.............R..GRW.......
tr|B8HFW5  ............AVRTIVAVGNVAHRSLLRNG..................LD............................
tr|B8I5K9  ............KPQIIVTLGNVPLKAVTDNFRLS...............IGDM.............H..GVL.......
tr|B9KXL9  ............RPSVIVTLGRYSMARWFPNE.K................ISRI.............H..GKP.......
tr|B9XNJ8  ............NPRIIVLLGGVAVKKMLGKKTVEEAR............CGIV.............EHEGRK.......
tr|B9Z7Q7  .....AEN....PGCVFLLWGNWA......QG.K................AERI............DT..RR........
tr|C0BNZ8  .....D.K....RPIIFVLWGNFA......QK.Y................ERFI............TE..DH........
tr|C0X269  ............QPELILTLGNIGLQRLLGPKPT................ITAV.............H..GTV..IQSPI
tr|C1A7L2  ............DLRGIVTLGKVAARTVEACLTR................YPVL.............A..AKV.V.....
tr|C1D9B0  .....A.P....GARVFLLWGKDA......HA.K................RHLV............DD..R.........
tr|C1F1L1  ............RPEMIVALGSTAATYLLGAKSP................LKAL.............R..GRI.......
tr|C1G9A4  ....TR.T....RGVVFLAWGKPA......GK.R................VAHI........L..KDK..GK........
tr|C2MIJ3  ............NPKLIVTLGNIG....LQRL.AGKDKK...........ITDV.............H..GQL.LKQPIQ
tr|C3X2T7  ............RPKIMVALGKTAAVALLNTG.RDVS.............LGSL.............R..NRE.HFFRTG
tr|C5CIT9  ............KPKLIVCLGATALAAFLESEKVQ...............ITKY.............R..GKL.......
tr|C5NVK4  .....K.N....ENVVFVLWGAYA......RS.K................NKLI............DP..SK........
tr|C7H0A3  .....N.N....ENIVFILWGNYA......RE.K................RRLI.............D..GS........
tr|C7M312  ............DPVVIVALGQSAAAWFLGPGAR................VGAL.............R..GQV.......
tr|C8W879  ............KPDVIVCLGNFATQFVMHTDKG................VTEL.............R..GKF.......
tr|C9M5Q7  ............RPDGIITVGNVPSRALLGRR.EG...............VTAL.............R..GRF.......
tr|C9RLM9  .....V.R....ENLVFLLWGSFA......IK.K................QVLI.............A..PN........
tr|D0MK10  ............KPRIILCVGRIAGNALLGRNGS................LRAL.............R..GKV.......
tr|D0PPD4  .....K.N....DKCVFMLWGNKA......IE.K................EVLI............NG..SK........
tr|D0U4F0  .....K.K....QNLVFLLWGAYA......QK.K................TELI.............D..QD........
tr|D1ART8  .....K.D....EQVIFVLWGGDA......KK.K................VPLI...........T.N..PK........
tr|D1C3J5  ............DPRAIVCLGATAGSAVLHRALR................ITRE.............R..GIW.FDGPNG
tr|D1CDM9  ............KPSVIVCVGAPAAKTLIDKNFR................LSDQ.............R..GDV.......
tr|D1JDZ5  ............EPAVIATLGNFSLAYIFAKFQLKPEK............IGEI.............H..GNV.......
tr|D1PN05  ............APLAAILWGKPAQKYAPLFK..................DASA.............R............
tr|D1Y5H9  ............RPRLVLAVGNVP....SQTL.LGVKTG...........INGL.............R..GRF.......
tr|D2EF83  ............NPKLILALGTSAATSLGIEFKH................LSEI.............K..GKV.......
tr|D2Z573  ............RPQVVVTVGNVPSQTIIDTKEG................ISTL.............R..GRF.......
tr|D3B454  .....Q.C....ENVVFIFWGAFA......KK.K................GKEI.............S..RK........
tr|D3E6H2  ............RPDILAPMGNTALHRLIGSHET................VTSL.............H..GKL......M
tr|D3FBP7  ............EPKVICTLGNFSTKLLRDDPTG................ISRV.............H..GQP.......
tr|D3QZ35  .....L.N....RPLVFMLWGKQA......QS.I................RPLH............PN..PH........
tr|D4G8B4  .....L.K....EGVIFLLWGNFA......KS.K................KNLI.............N..RN........
tr|D5C4I1  ............RPKLLVLLGATALQALYDPKAK................ITEL.............R..GNW.......
tr|D5E730  ............EPDVIILLGNTAEEAFLGNRQLQWGE............CRDI.............D..GKN.......
tr|D5EGK6  ............SPLLIVTAGNTPTQWFLNTRKG................VTAL.............R..GQY.......
tr|D5EHP1  ............QPKVIVALGNTAVSGLLGQDPNRR..............LGQV.............R..GNW.......
tr|D5MIW4  ............RPKLICALGTFAAQTLLRTREP................ISKL.............R..GRF.......
tr|D5RKP0  .....R.P....DPVAFLLWGGHA......RQ.R................AKLL.............T..GT........
tr|D5U040  ............KPRVLILLGNTAAETIYGLSGVEW..............HGMM.............RDHGKI.......
```

```
tr|D5U570 ............NPKIICALGRHGFGNLIDFDLKTP..............FGKA.............R..NKV.......
tr|D5V6V9 .....N.C....EDIIFILWGAPA......IA.K................TKLI.............D..SN........
tr|D5WWR6 ............QPEIVVTMGATAIQAVLGRT..................IPVG.............RASTRF.YHARHR
tr|D7CKV6 ............SPRIIVCLGLLATRTVIEAKAT................MAGI.............H..GQW.......
tr|D7CWJ9 ............RPQIIATLGNFPTQYFAGTKEG................ITKT.............R..GRW.......
tr|D7UXZ6 ............SPEVIVTLGNIGLKRLLGSDWS................VSAA.............H..GTV..YQGPI
tr|D8LFW4 .....G.D....RRLVFLLWGKPS......QK.K................GALI.............D..SS........
tr|D8UI66 .....R.R....SGIVFLLWGNPA......QA.K................ASLV.............N..TR........
tr|D9Q1V3 ............RPKVIVALGNTAGSALMSLAGKQWRG............VTAE.............R..GRK.......
tr|D9QQQ4 ............QPMIIVPLGSIAVKGLLDPDGK................ITEL.............R..GNW.......
tr|D9S3H2 ............APKVIITLGSVPLNYFKPNSSI................MQSR................GQW.......
tr|D9SCW5 ............QPAVIVALGKTAASALLDTDAT................IASL.............R..GIV.......
tr|D9Y2E5 .....E.G....RPVVFMLWGKSA......QE.K................KKLI..........RKFN..KD........
tr|E0S9I8 .....K.C....TNVVFMLWGRHA......QA.K................GKFI.............N..KN........
tr|E0SQC9 ............KPKIIVTLGNIAGKTIFFMGKERWEG............IMKM.............R..GRV.......
tr|E0TBI7 ............APEVIILMGNVSTQALYPGAPG................ITRT.............R..GSW.......
tr|E0TIS2 ............KPKIIITLGKIAFSSIFNIN.KNKFT............IKKN.............K..NKI.......
tr|E1IBI3 ............AGVPVLAMGKSAARS.......................LHQL................GIS.......
tr|E1L0U9 .....H.P....QSKVLLCWGNFA....FTIA.Q................DALS............HV..DA........
tr|E1QFX8 ............GPKVICTLGRPA....ALAV.LGSDAP...........ISAL.............R..GNW.......
tr|E1QNN8 ............RPQIIICLGRHSAREILTMAGYPEKSVSN.........ISSI.............R..GKV.......
tr|E1QWE2 .....RLG....HHVVLLCWGKQA......LA.Y...........AQG..SPAA............TL..PS........
tr|E1R614 ............RPKAILTVGRIAMQLLLDTT.DG...............IGRR.............H..GKR.......
tr|E2IUG8 .....R.R....DGLVFMLWGQHA......Q..G................AIKP............DT..QR........
tr|E2SYG7 ............DPSIVVTLGAVALKACGELEAH................SFSL.............R..DQV.......
tr|E3D056 ............RPSLLVLLGNTPTKWILKTTEG................ISKL.............R..GRW.......
tr|E3IP47 .....RCP....RPMAFLLWGRHA......AA.Y................GEGL.............A..AH........
tr|E4KQ36 .....K.A....EPLVFVLWGRQA......QN.Y................QKYL............KK..HH........
tr|E4RKG8 ............EPKVVVTLGSTATNFLLNPEDS................ITKM.............R..GKW.......
tr|E6N689 ............NPRLIVLLGSVA....VQTL.LGISS............VTAV.............R..GKA.......
tr|E6PFU2 ............RPRVLLALGAPAAKSFLGPAYA................ITKQ.............R..GRW.......
tr|E6W1H1 .....TQK....QGVIFVLWGQPA......QQ.L................QASI............DQ..DR........
tr|E6W552 ............NPRFILALGKFAANFLYGESGAPRT.............LGSM.............R..EKI.......
tr|E7A9L4 .....L.Q....RPLVFIFLGRVA......QE.K................IKLL...........NPN..PQ........
tr|E7FX98 .....R.K....EPIIFVLWGKKA......QA.K................KSLV............KD..HH........
tr|E7H418 ............KPDVLFLAGRFAISSLLKPADEMFS.............ISKM.............R..GRI.......
tr|E7H4Y1 .....G.R....NGLVFFLWGKPA......QR.L................RPMI............DE..RR........
tr|E8LLT1 .....N.C....ENVVFMLWGAAA......RA.K................CAHV.............D..RR........
tr|E8N338 ............SPKVIVTLGRFSMAKFLPGV.K................ISEV.............H..GQS.......
tr|E8T398 ............SPKVILCLGAFAARTILNLPERTP..............ISRI.............R..GKE.HKTTIG
tr|F0EKF3 ............DPPVIATVGNIGLQRLLGNAYY................IKDH.............H..GEV..IRSKI
tr|F0NSC6 ............DPQIIVALGNTGLERLLGPGHK................ISAE.............H..GKV.IENTPI
tr|F0RSJ4 ............KPQIILLVGSVAARSLLGVSDG................VGKL.............R..GRF.......
tr|F0SG07 ............NPRFIVCWGTVAAQNLLGVNTP................IGRM.............R..GKL.......
tr|F0Y985 .....R.E....RPVVFALWGNPA......RK.K................AAIV.............D..GA........
tr|F2LXL6 ............KPKIIATLGRYSTYELTNQKGA................LGTL.............R..GKV.......
tr|F1A0X7 .....Q.P....QPIVFILWGGFA......KK.K................KSIL.............K..GK........
tr|F2NFM2 ............RPQVIVTLGAVAIHALLETKAP................LNRL.............R..GHW.......
tr|F3KRD3 .....Q.V....SPVVFMLWGAHA......QK.A................LAHACAGQGWKEERVGAQ..GR........
tr|F3QMU0 ............DPEVIVAMGKPAASWFFPDLKT................LNPY.............R..GKV.HNLTVQ
tr|F4A8X0 ............NPKIIITLGNVPLKRLTEFK.S................IGDC.............H..GNL.......
tr|F4C0J7 ............RPRIICLMGNTAIQAVLGVVG.................VTVL.............R..GRI.......
tr|F4GMI7 ............KPHRILALGSLA....AQAV.LGLQND...........LEAL.............R..GKV.HAMRSE
tr|F4GSY3 .....E.T....RPKVFLLWGAHA......QS.K................EVLL........QAHPPA..GP........
tr|F4HNK0 ............RPKVIVTLGRFSMGYILRKYGFAVEP............ISKV.............H..GKV.FEARTL
tr|F4NZD5 .....N.N....QGVVFMLWGGFA......QK.K................GTVV.............D..KK........
tr|F4QWL4 ............KPKAVLLVGAAAAKAVLKTD..................DGIM.............RTRGQW.......
tr|F5SL17 ............RPKLIVTLGSAPTRALIDPAAR................ITRV.............R..GQW.......
tr|F5Y215 ............QPKIILAMGRFAVQSLLGTQEP................IGKL.............R..GRP.......
tr|F5YGI3 ............EPRIILCAGRVAAQTLLKTGSP................IGSL.............R..GQF.......
tr|F7NHV1 ............KPRIIIALGSVALKYLLNANAR................ITKD.............R..GKW.......
tr|F7PTA1 .....K.S....DPIVFVLWGKQA......QK.K................NVLV............TN..KN........
tr|F7S094 .....Y......QACAYLLWGKHA......QN.V................AEPI.............V..AN........
tr|F7XVS9 ............QPKLIIIVGSTAIESLLRIKSVS...............MNSL.............RTQNHL.......
tr|F8ADN2 ............RPAIICTLGRVAAQTVLATTSD................LGSL.............R..GKI.......
tr|F8L559 .....R.Q....DPLVFILWGKSA......KE.K................CENI...........LSQ..TN........
tr|F9CXI5 ............KPKIICILGNTAFNSILGGSEI................IKHR................GKI.......
tr|F9DTW2 ............KPELIVTLGNIALQRLLGKHAK................ITTL.............H..GQL.LTQPVQ
tr|F9LK36 ............DPRIIVTLGKTSYERITGFSKN................LSDC.............H..GKK..IITKI
tr|F9ZLD4 ............EPEAILLVGGTATSVFCGSGPLH...............THIV.............D..LRR.GDHQLN
tr|G0LJW8 ............NPAVIVTLGKVPSEHLLDRQIA................VTTA.............A..GDV.......
tr|G0QBL8 ............SPDRLVPLGNFASRELLDTSKG................ISSI.............H..GRE.......
tr|G0QFK7 ............DPDIVLTLGNFASKEMLDTS.KG...............ITQI.............H..GQI.......
tr|G0U4E4 .....E.HT...KSLVFLLWGGFA......RK.K................ISLI.............D..QK........
tr|G2RZ19 .....N.N....KKVIFCLWGNYA......KQ.V................YHSL...........D.H..KP........
tr|G4RL69 ............RPRLIIALGLYSARTLLSLANIKTPRL...........LEDV.............R..GEC.......
tr|G4STH7 ............KPKIILAVGRVSAQNLLKTDKN................IGQL.............R..GTV.......
tr|G4T6H9 ....PS.K....RGVVFMAWGAHA......QK.A................IAGF...........DQK............
tr|G7WNS9 ............QPRTVILMGNAAARAVLDVEGIGELR............GRVI.............R..GR........
tr|G8BJ42 .....H.K....SGFVIMAWGSPA......QK.R................LKPFEKILAGGGGGDKKD..GG........
tr|G8LWR8 ............EPKYIVTLGNVPLRSVLGDYKAS...............IGEF.............H..GRL.......
tr|G8TXV8 ............NPQVIVTFGRTAWESLTGGSWKDVTAGAP.........FPLA.............E..SRW.......
tr|G8XT11 .....T.R....EHLVFMLWGAEA......QT.R................EYLI............DK..TK........
tr|G9EIR0 ............QPSLILALGRFAGQFLLNKP.LA...............LKQL.............R..NNV.......
tr|G9XY09 .....I.R....DDIVYVLWGNYA......KQ.Y................SEYI.............L..NK........
tr|H0E6E4 ............RPKVVCSLGTFATRLLREDPAS................IRAV.............R..GRA.......
tr|H0KUY0 .....K.K....KNVVFVLWGAFA......QK.K................AALI...........D.T..SK........
tr|H0Q5N1 .....TTQ....SGIVFVLWGKPA......QA.L................GEHI............VG..NG........
tr|H1D376 ............KPKVIVGLGKVALRFFLGHE..................AGII.............RSRGHW.......
tr|H1P1U4 ............GWLRVITLGKRA.....QWI..................LTQL.............R..GAP.......
tr|H1P505 ............SPGVIVALGATPLRELLGVTTG................ITRI.............R..GQW.......
tr|H1XWX4 ............KPKLIVALGRFAAQSLLRLEQP................LGVM.............R.EGD........
tr|H2C8Z4 ............RPKVVVTLGRHS....TAFF.LTLAGNEKAS.......ISKV.............R..GRP.......
tr|H2J7L7 ............KPKIIVALGSTALNYFLKQTVP................ITKH.............R..GRF.......
tr|H3KDP1 .....E.R....DGLVFMLWGRPA......QE.K................IPLI............DA..GK........
tr|H3NN78 .....K......GNIIFLLWGNDA......IN.K................SRLI................CE........
tr|H3NSX2 .....S.D....KHTAFLLWGAHA......QK.K................ADLV.............K..GE........
tr|H3RRL7 ............RPKILMPLGNTSLQRLLGPSAK................IGSL.............H..GQI.......
tr|H3SCZ2 ............DPAIIVCLGSIAVQALVDRKAK................ITEI.............R..GTW.......
tr|H4GIS2 ............QPDVIIPVGNTALHRLLGPDEV................ISAV.............H..GQL..IQHPV
tr|H5SI33 ............RPRVVCLLGATATRALLGSDQK................LNQV.............R..GRA.......
tr|H6RLZ5 ............DPPAVIALGLSAAKWFLGPRTV................LARA.............REVGPV.......
tr|H7F338 ............QPKILLLMGNIA....LRRV.LGGKIS...........VSDV.............R..GMR.LYEEAL
```

```
tr|H8I7F5 ............SPKIIVLMGRTAAEAMLERK..................VNLG.............KEHGTV.......
tr|H8ZDF2 .....S.S....KEVVFMLWGKDA......QR.K................QSLI.............D..ES........
tr|H9UI22 ............QPPMLLVLGQVAAHSLLNTDTP................LADL.............R..GQL.......
tr|H9ZZZ0 ............NPLKIVLLGGTAVKYLYDESFPYKKIPKEKK.......ISEI.............R..GKL.......
tr|I0GI60 ............EPEIIVTLGNTPLNALVSPALK................VSAV.............H..GRV.......
tr|I0I6B7 ............RPRIIVTLGRLSMRRWFPEG.S................ITRL.............H..GRA.......
tr|I1A005 .....E.N....PNVLILALGGQA......HK.V................VENA.............L..KL........
tr|I1B0L2 .....S.A....RRRAFVLWGGPA......KA.F................EPHI.............R..GG........
tr|I2K0D9 ....VA.H....QGIVIIAWGSPA......QR.T................IGKV........G..RID..WG........
tr|I3CUN9 ............QPTVLVALGKTAAISLLGMDPATP..............VSRL.............R..GTV.......
tr|I3VQA4 .....N.N....ENVVFMLWGKHA......QE.K................EYLI............DG..GK........
tr|I4CBR6 ............EPDVIVTLGRIAAKALLKTSDP................LNVI.............R..GRV.......
tr|I7LIA9 ............KPKIIVTLGNVPLKALLYDKAS................IGEY.............H..GKL.......
tr|I7LKX1 ............GPEVIVTLGRHSSRYILSYLSVEFDR............ITDI.............R..GTV.......
tr|J0UVA7 ............QPRRLLAVGHVAAMALSGCEQD................LETL.............R..GQV.......
tr|J5SPB4 DAPGSS.A....NGIVLMAWGAHA......QK.M................IAGL...........DTK............
tr|J7R1V5 ............KNIVFLLWGNNAIKLVERLLEK................VTKE.............SGKSTW.......
tr|J9E0C3 ............EPDILVLVGGVSAKEMLNST.LG...............ITKL.............R..GSW.QDIDIK
tr|J9Z1B8 .....A.R....ENIVFILWGSFA......QS.K................KELI.............D..SN........
tr|K0IF13 ............RPKVICILGRTA....YSSL.LGGGS............ITAN.............R..GKI.......
tr|K0KF39 .....N.D....RKIVLLLWGTPA......QK.R................ITNI...........KIN..KD........
tr|K1JXC9 ............RPDVLLIMGRFAMQTLLGLGPDAS..............IGSQ.............R..GRV.HDVTIG
tr|K1JYF3 .....T.A....PHAVFMLWGNFA......QG.K................RALI............DE..EK........
tr|K1LNP2 .....QAD....PGIVFLLWGNPA......QA.L................IPDI............DQ..TK........
tr|K1ZRE1 ............KPKIIATLGRFSTKYILEKFNLSEKNES..........ITNL.............H..GKV.......
tr|K2A3B6 ............APAAIVTLGRFSMAKFLPGA.K................ISRD.............H..GRP.......
tr|K2B4F4 ............RPALIVTLGRHSMGRFLPGL.K................ISQA.............H..GQP.......
tr|K2C359 ............KPKFIGTLGSTASKFLLGDDIGT...............ISKI.............R..GNW.......
tr|K2CLQ5 ............QPKLVVLLGRVAAEGVLEKK..................VQVM.............KEHGEI.......
tr|K2DEQ6 ............APKLIVTLGRFS....MNHFYK................DGKI............SRDHGHI.......
tr|K2E7J1 .....N.R....KNIVYLLWGKYA......QT.K................GEVI...........N.R..EK........
tr|K2E9B4 ............RPRLILLAGATA....VKSM.LDTKAP...........ISKI.............R..GQW.......
tr|K2ETW7 ............QPKILVAVGRIAAQFLLDTAEP................MSRL.............R..GQT.......
tr|K2F640 ............DPEVIITLGRFSMAKFLPNV.F................ISSV.............H..GKK.......
tr|K4KIT8 .....Q.C....EHVVFLAWGRFA......HK.V................CESI............DA..KR........
tr|K6GHM9 .....R.G....K.MIFVLWGKEA......QR.K................KNLI...........D.P..KI........
tr|K7YPM8 ............APRILMLVGGTSVRALMKIHDG................IARL.............R..GNF.......
tr|K8ZAC1 .....S.E....KPIVFLLFGRQA......LN.K................RALI............DE..EK........
tr|K9VTI9 ............DPKIIVLIGATAVRGLLGNRGG................ITRI.............R..GTW.......
tr|L0A9A2 ............KPELILTLGRTAGEAISIIFNYEWEG............LEKE.............R..KKI.......
tr|L0B4R8 .....N.N....SPKAFLLWGLAA......QK.K................TNLI..........TNNV............
tr|L0B866 .....D.N....SPKVFMLWGSYA......QS.K................NSLI........KSSQRD............
tr|L0B8A6 ............NPKTILALGKFSANTLLCSDSS................IRGL.............R..GKI.......
tr|L0DFI2 ............RPEFLCLLGRIAAQNLLSTALS................MGRL.............R..GKW.......
tr|L0L178 ............DPEVIILLGNTAEQAFCVSP.LKWGEA...........VNMQ.............E..GKY.......
tr|L2GQC8 .....R.L....QHVVFMLWGNHA......RQ.N................ARLI............DG..AR........
tr|L2GX11 .....E.C....KHVVFMLWGSFA......GK.K................CALI.............D..GK........
tr|L5N8A0 ............DPDIILVLGGVALRRLTGEEGK................ISVL.............V..GQLMETPLLK
tr|L8HA23 .....K.R....EGVVFMLWGKYA......QD.K................GKII.............S..KR........
tr|L9X1W2 ............DPSVIVPMGSFATGELLETDET................ITDV.............R..GES...FECE
tr|M0BKB2 ............EPAVVVATGKHATTSVLAADDRSLDDLDGSGFLDTVLEPTRC.............EALETW.......
tr|M0CBA6 ............DPAVVVTLGAVASGAVLDTDEP................VGDL.............R..DET.......
tr|M0MGS7 ............EPRAVIATGKHATTSLLATEGKSIDGFLDSILEPVA..LGGL................GTT.......
tr|M1E4Q4 ............NPKIIILLGAVACKTILKNFSS................ITKI.............R..GEI.......
tr|M1LNF1 .....D.N....NPKVFMLWGLQA......QE.K................ERII........KNQNNH............
tr|M1QKH3 ............KPKLVIALGRTAAVGLSGIKQIS...............PTKV.............R..GSF.......
tr|M1VCA8 .....Q.KLAGTRGLVFMLWGNFA......KE.K................CSRV.............N..AN........
tr|M1Z9H8 ............NPKIIATLGNVPLKTIFEDD.LK...............IGEV.............H..GKP.MEIEIQ
tr|M2XVU5 .....N.C....NGIIFLLWGKYA......QQ.K................GKDI.............D..RK........
tr|M3EFG6 ..................................................RAPT.............K..KK........
tr|M4YU22 ............TKKVIVGLGKSAIRDLMGYEGP................MKPI..........VNTR..QHI.......
tr|M5ADY8 .....Q.K....KNIVFLLWGKYA......QR.K................IPLI...........NSF..HD........
tr|M5IVB6 .....QTT....RPIVYMLWGAHA......QA.K................EALI........QQHSQH...P........
tr|M6D8H9 ............QPKVIITLGNPSTRFILRTK.EG...............ITKL.............R..GNW.......
tr|M7WR77 .....K.G....KGVVVLAWGAWA......AK.R................VAKM...........DKK............
tr|N0BBN4 ............RPKAVVCLGRHSASLIFSFFGLEFNG............ISKE.............R..GKI.......
tr|N1V4N6 ............SIESIVAVGNIG....YRS...................VQAS................GTS.......
tr|N1Z687 ............QPNMIVTLGNVPLKAITGDDSSS...............IGDM.............H..GVM.......
tr|N2BLL9 .....H.F....SGIVFMLWGNFA......KK.K................ISLI............DS..TK........
tr|N9SHT1 .....N.K....VDIIFLLLGQKA......KT.S................VENV...........N.F..EK........
tr|N9V498 ............QNVIFGLWGKQS....YQFI..................KPLI............ER..GKI.SENQ..
tr|Q025I0 ............RPKAICALGGTAARAVTGHKEG................VTKM.............R..GKW.......
tr|Q0C0Y4 ............RPRLIVAAGGIPAVSLLGSREG................IMKL.............R..GNE.......
tr|Q0EZR7 ............QPKMICLLGRVAAQTVLGTDAT................LGAL.............R..GAW.......
tr|Q12TN4 ............DPNLIVLLGNTAEKAYCSGHKMKWGA............VEML.............E..GKN.......
tr|Q1AV32 ............GPGALVLLGSTPAKALIDRSFT................MREG.............R..GRI.......
tr|Q1AYQ1 ............RPEIILCLGGPAAKTIIDRDFR................ITRD.............R..GRW.......
tr|Q1GY28 ............APLVFILWGNHA.....QQF..................RPLI................ASW.......
tr|Q1N1I3 .....H.K....RGVVFLAWGRFA......HD.V................CSQV............DT..SK........
tr|Q23W06 .....E.C....KDIVFLLWGKPA......QK.K................ASIV.............D..TK........
tr|Q3SA81 ............RPRVICPLGRFSAKFILEKFGFTMKS............ITAA.............Q..GKI.......
tr|Q53CZ3 .....K.L....QRCVFMLWGRKA......ID.K................AVLI............NG..QR........
tr|Q5DC15 .....N.R....CNLVFMLWGASA......QK.Q................GQSL.............D..RK........
tr|Q5WES9 ............DPKLIVTLGTIA....LKRL.VGPGLN...........LQDV.............H..GKP.FCTRLL
tr|Q6CAC0 .....H.N....SGCVILAWGSPA......AK.I................ADKM...........NID..KS........
tr|Q6F1M3 .....Q.N....PNIVYCLWGNYA......KK.V................YENL...........E.H..KA........
tr|Q6KIL8 .....N.L....SNVIFLLWGNDA......KE..................MKCL.............I..DS........
tr|Q7VBS6 ............DPLIIVLIGSSA....LRAI.LGIKSK...........ISDL.............R..GTW.......
tr|Q8TK31 ............DPEIIILLGNTAEKAYCPERKLERGVP...........VEYE................GRM.......
tr|Q9RTK9 ............RPRVVLSLGNTATQFLLGTPRG................ITRL.............R..GQW.......
tr|Q9WR44 .....Q.R....TGLVFMLWGVNA......Q..R................TIQP............NP..SE........
tr|R1ATM3 ............KPDFIVTLGNIPLKTLVEDSNVK...............IGEL.............H..GKL.......
tr|R1E4B2 ............QPDIIVTLGHYSTKWIFDYFKLKFSS............ITKL.............R..GKI.......
tr|R1FRL1 .....R.T....TPTVFILWGGFA......QK.K................GKMI.............S..RT........
tr|R1GL12 ............NPKILLCVGAVACKALLRSHDN................VSVL.............Q..NSN.......
tr|R2SFV7 ............QPEIIIPLGNIGLHRLLGPKHS................ITQD.............H..GQI.......
tr|R2SXC9 ............QPKILAPMGNIGLQRLLGNTFK................ISNY.............H..GQL..LHSPI
tr|R4KRR4 ............SPRVIVALGDVALKRLIGGSGR................IGNC.............H..GQP.......
tr|R4VE87 ............APACILAVGKVSAETLTGTRDG................LGTL.............R..GQW.......
tr|R5ARL3 ............RPRVIVTLGNTPLLALLLLGIEAQT.............VGAV.............H..GKP.......
tr|R5B4U8 ............RPHVIATLGNSPLASMARISGVAAPK............IGEA.............H..GQA.......
tr|R5DX78 ............NPRAIVLCGATALKSFVELDKKQT..............ISKV.............R..GQW.......
tr|R5MPG0 .....R......GNVIFVLWGSFA......RS.K................KVLI............NT..QK........
```

```
tr|R5PZ23   ............NPKILLLLGGSAVAALTGITQG................ITRI.............R..GKW.......
tr|R5QX00   ............NPDFIFTLGGSA....ANAL.LDTADS...........ISKM.............R..GRW.MEYTKS
tr|R5Z1D7   .....K.K....GPVVFLLWGSKA......KQ.V................LPLL............DN..PK........
tr|R6HGV3   .....MLP....QPIVFLAWGRHA......VA.LV....ENARTKAAG..HESA............AL..DN........
tr|R6HL97   .....R.N....KPVVYILWGSDA......IA.K................RSII.............S..PK........
tr|R6PFR8   ............KPKIILLCGATAVQSFVEQDFK................ISLI.............R..GKW.LNIFDD
tr|R6PI86   ............KPRIILLCGNIAVQSFMEGA.KG...............ITKV.............R..GQW.......
tr|R6R865   .....D.D....NPKVFILWGNSA......KS.V................MKYI............HN..KN........
tr|R7BZ83   .....A.A....TPCVYMLWGNWA......QA.K................ADGI.........RHNAK..GP........
tr|R7DQ64   .....S.D....EPIVFLLWGRFA......RN.L................KPYI............TN..PN........
tr|R7DXQ1   ............QPKMIVALGGTAAVGLLGIQGS................VSSM.............R..GRF.......
tr|R7I8P1   ............RPKVIVLLGSTAAKNIIGAD..................IRIT.............RDRGKW.......
tr|R7IAS2   ............APKVLVLMGRPAASAVLGTDQA................ISRL.............R..GKL.......
tr|R7IHQ8   ............QPKMLVTLGNTALHALLGAKST................IGAC.............H..GQV.......
tr|R9ADN8   .....K.S....KGIVFFCWGNWA......KK.M................LEGA...........KVD..KK........
tr|R9LKN0   .....L.K....QPIVFILWGAKA......RE.A................KKFL.............T..NP........
tr|S0EV15   ............QPLVILCLGAPAANVLIHKGFK................MSQE.............R..GQW.......
tr|S0FG57   ............CPKIIVTLGNVPLKAVTDDFNAS...............IGEM.............H..GST.......
tr|S2J4F7   .....K.K....ANIVFMLWGSHA......QK.K................GARI...........NKS............
tr|S3JT40   ............RPKMILVMGRIAAQHLLQTSDG................IGKL.............R..GRF.......
tr|S4NBY8   ............PNVLLVPMGNASLQRLLGNKAK................IGDL.............H..GQL..LESPI
tr|S5MFT9   .....F.N....NNIIYCLWGNYA......KN.L................YNNL........INGD.N..KI........
tr|S5RQ34   .....K.H....KNLLFILWGNYA......IS.K................KIYI.............D..QS........
tr|S6G4W1   ............DPDVVVMVGSTACYSLLQTPDN................ISRL.............R..GKF.......
tr|S9VE50   .....Q.RE...RRVVFLLWGNFA......RG.K................KKLI.............D..TS........
tr|T0AYQ1   .....N.R....QNVVFMLWGGHA......QQ.K................EKLI.............K..SD........
tr|T0DRC9   .....A.N....APLIVVLLGKIA......QK.K................IALI............PK..NK........
tr|T0LSQ5   ............KPKVIIPLGNSSLKALTLITEVKYGN............ISKI.............V..GEK.......
tr|T0MGQ8   .....K.C....NNVVFMLWGRYA......KS.K................AILI.............D..CN........
```

```
                         180               190                           200
                          .                 .                             .
sp|P12295  ..............R.HH.......VLKAPHPSPL....SAHR...G......FF..G.....CN.......HFVL....
tr|Q8KLI8  ...........YEWHGIK.......VFPMFHPAYL..................LRNPSRAPGSPK.......HL......
sp|A1QYK3  ................HL.......ILETSHPSPY....SAHN...G......FL..G.....SN.......HFSQ....
sp|A1SUE5  ..............K.HT.......VLTSTHPSPL....SAYR...G......FL..G.....CK.......HFSQ....
sp|B0B8I2  ................HA.......VLACPHPSPL....AAHR...G......FF..G.....CC.......HFSK....
sp|B1AJI8  ................NL.......ILCASHPSPL....SAHR...G......FL..T.....CK.......HFSK....
sp|P12888  ................HL.......VLTSQHPSPLAQNSTRKS...A..QQK.FL..G.....NN.......HFVL....
sp|Q057V4  ................HF.......ILRSTHPSPL....SCYK...G......FF..G.....CN.......HFFK....
sp|Q315T1  ................HL.......LLESVHPSPF....SAHK...G......FL..G.....CG.......HFVT....
tr|A0LFG4  ...........HYWRGIP.......LICTFHPAYL..................LR..T.....P........AQKA...A
tr|A0LH32  ...........FRGAGLN.......ILVTYHPSPF......................N..............FMVP....
tr|A0LRS0  ...........HELRGWR.......VVPTYHPSAA..................LR.FG..............PTGAPRTA
tr|A0RV64  ...........INQDNTD.......YLVSLHPAAT..................MYRRD..............LLPA....
tr|A1BDC5  ...........VKWKAYD.......CMVTYHPAAL..................LRNPG..............WKKA....
tr|A1RYC0  .......YTVNAEWGQVT.......VFPTLHPAAA..................LY..N.....PA.......MRKV....
tr|A1US05  ......LTYETEDKVKIP.......VMPTFHPAYL..................LRTPS..............QKKL....
tr|A4GHT2  ..............R.HK.......IFYAPHPSPL....SAYK...G......FF..G.....CK.......HFSK....
tr|A4HXS3  ..............R.HI.......VLESVHPSPL....SASR...G......WF..G.....CH.......CFSA....
tr|A5KS51  .......KRVTFGKEKIV.......VVPLFHPAAA..................LYNGG..............MRQT....
tr|A5UQ47  .........................ILPMMHPARI.....................................SRVD....
tr|A6DBB2  .......DVDTEKFGKIK.......IGICYHPSPL..................AFNRK..............EIRESLEK
tr|A6DK00  ................HL.......IIESVHPSPL....SASR...G......FF..G.....SK.......PFSK....
tr|A6GN76  GLDIP....................AVVTYHPAYL..................LR..R.....SE.......EKQK....
tr|A7ANQ0  ..............R.HC.......ILKCGHPSPL....S.QK...L......FF..G.....CD.......HFNK....
tr|A8A982  .......LVASAGGARVK.......VLVTYHPAAV..................LR........NP.......KLKE....
tr|A8E1M5  ................HL.......ILRSSHPSPRA.....QG...A..RNP.FV..G.....NN.......HFVL....
tr|A8F067  .....YFYTNKYLSSPIQ.......TTTIFHPAYL..................LR..H.....PM.......QKRT....
tr|A8MA01  .......FKVNIAGVNAD.......VYPTYHPAAA..................LYNPS.....VR.......NLIK....
tr|A8SNH5  ...............NNF.......IITSTHPSPF....SAHR.........GFF..G.....SK.......PFSK....
tr|A8UAT5  RLVSLKDNTYTWTEKDYA.......VFSTFHPAAI..................FY..N.....RQ.......LLET....
tr|A8UU74  ........VPYPYDPSIT.......VLLTYHPAYI..................LRNPR..............ADGE....
tr|A9AZ06  .........................FLPLFHPAYV..................LRNMN..............AMPD....
tr|A9BHU0  ..........FNWYGDIK.......IFPTFHPSYL..................LR..N..............QSSTKGSP
tr|A9F5H8  ..........KLYKGSIP.......IMPTFHPAYL..................LRQPS..............AKRE....
tr|A9NHD4  ................HM.......VIESVHPSPF....SARL...G......FF..G.....SK.......PFSK....
tr|B0VFE1  ...........HYFMDIP.......AFVTYHPSAL..................LR........NQ.......EWKK...P
tr|B1GA08  ..............S.HY.......VLEAPHPSPL....SAHR...G......FL..G.....CG.......HFAK....
tr|B1H0K1  ...........REYKGIK.......LIPTFHPAAL..................LR........NQ.......DLKK...F
tr|B1I4W5  ........LESFRWQRSLVAA....VIPTYHPAFV..................LRRLD.....GD.......ILDQ....
tr|B1VA82  ................NY.......IIKSPHPSPF....SAAN...G......FF..G.....SK.......HFSK....
tr|B1Y7I1  ..............PAHL.......CLCSNHPSPL....SARRPPVP......FI..G.....SA.......PFSR....
tr|B1ZZ66  ...........HTFADKP.......LMVTYHPSYI..................LRNQS.....NR.......SKRM....
tr|B2A668  ...........LERKGIH.......ILPTYHPAYL..................LR..N.....PA.......KKKE....
tr|B2KCZ8  ..........HTLELDNFDLEGKTKIVATYHPAAL..................LR..N.....PN.......WKKD....
tr|B3DV47  ...........FSFNGAT.......LIPTYHPSFL..................LHNPS.....LT.......NKRK....
tr|B3U4N1  ..............Q.HV.......VISCAHPSPL....SA.R...K......FF..G.....SR.......CFSQ....
tr|B3U4U7  ...........FYLKEFV.......LFPLLHPAAA..................LHQGT..............MLQP....
tr|B5II11  ......RQGSGPVLAGRW.......LMPIFHPSYL..................LRNPS......R.......QSGSPKWL
tr|B5LRM7  ................HC.......VLKYSHPSPL...........S..RKP.FI..L.....CD.......HFKK....
tr|B5Y8I5  ...........VLSGETK.......VVVTYHPAAV..................LRNPR.....LG.......EIVE....
tr|B5YEZ9  ...........YVVDGKR.......IFVTYHPAAA..................LYHGK..............LLEN....
tr|B6A3R0  ...........LQPANHPP......VLVTIHPSYL..................LRIRD..EAEQQ.......RLRQ....
tr|B6AFI4  ................HK.......VLKSAHPSPL....SQKN..........FI..G.....CG.......HFIK....
tr|B6K2R2  ..............T.HC.......VLRSVHPSPL....SAHR...G......FF..E.....CQ.......HFKK....
tr|B6KW10  ................HR.......ILEAGHPSPL..............SVRFFQ..G.....CQ.......HFLK....
tr|B7FQS1  ..............R.HV.......VICTSHPSPL....GARK...T...NQPFL..G.....SK.......CFSR....
tr|B7KM64  ...........FKWHNSK.......CISIFHPAYL..................LR..N.....P...SLEKGSPKW...L
tr|B8C427  ..............K.HA.......VIKTSHPSPL....GATK...T...AFPFL..G.....SR.......CFSR....
tr|B8CX81  ...........KKIGKYY.......FFPTFHPAFL..................LRNPK..............MKKL....
tr|B8EJL3  .......LTYDCGGTPIR.......ALAMFHPAYL..................LRQPG..............QKKL....
tr|B8HFW5  .........................APRIRHPAHG......................G.....RA.......GFKS...G
tr|B8I5K9  .......HECNLAGKVYK.......IFPLYHPASI..................IY........NR.......TLKD...V
tr|B9KXL9  ...........RRFGDVV.......VVPMYHPAAA..................LHQPA.....LK.......ELVE....
tr|B9XNJ8  .........................FLVTYHPAVR..................FYREE..............LAEK....
tr|B9Z7Q7  ................HK.......VLCAAHPSPL....SASR...G......FH..G.....CR.......HFSQ....
tr|C0BNZ8  ................HR.......VLKSGHPSPL....SANR...G.....YWF..G.....NK.......HFSQ....
tr|C0X269  QTFDEQSQNYHWTQKTYQ.......IIPLFHPAAV..................FY........NY.......RLKEVVKE
tr|C1A7L2  .........................TRAVPHPSAQ...........GLLSMAPNR..G..............KGARLADL
tr|C1D9B0  ...............RHL.......VLCAPHPSPL....SAWR...G......FL..G.....CG.......HFRQ....
tr|C1F1L1  ...........HQAHGSK.......LIVTYHPAYL..................LR..D.....PN.......QKKE....
tr|C1G9A4  ..............R.HC.......VLQSVHPSPL....SVHG...G......FL..Q.....CG.......HFKK....
tr|C2MIJ3  KLKDMQSAEFTWSEKEYH.......IFPTFHPASI..................FY..N.....RG.......LLEL....
tr|C3X2T7  GRKIP....................LVVTYHPSYL..................LRTPG..............GKKQ....
tr|C5CIT9  ..........FDWHGGIK.......VFGMFHPSYL..................LRNPS...TAPG.......SPKR...L
tr|C5NVK4  ................HL.......IIESAHPSPL....SAYR...G......FF..G.....SK.......VFSQ....
tr|C7H0A3  ..............R.HK.......IIEGVHPSPL....SASR...G......FF..N.....KG.......YFLR....
tr|C7M312  ...........LEWEGRA.......LVVTYHPSAA..................LR..G.....GA.......AIVA...A
tr|C8W879  ...........YQIGAFS.......VLPTMHPAST..................IYRKE.....WA.......QFLK....
tr|C9M5Q7  .......YSLEIGGRTAW.......VRPIFHPAYL..................LR..N..............RTRRPGSP
tr|C9RLM9  ..............RGHL.......ILTAPHPSPL....SAYR...G......FF..G.....CK.......HFSK....
tr|D0MK10  ...........HDFHGLP.......VVVTFHPAAL..................LRNPQ..............WKRL....
tr|D0PPD4  ................HL.......VLKAQHPSPLASLGGYSS...K..QSP.FL..G.....CD.......HFSI....
tr|D0U4F0  ..............K.HL.......VLTAAHPSPF....SAHK...G......FF..G.....CK.......HFSK....
tr|D1ART8  ................HK.......IIEGVHPSPL....SAHT...G......FF..G.....SK.......PFSR....
tr|D1C3J5  LP.......................TLVTYHPAYL..................LR.................RTQDRDDR
tr|D1CDM9  ..........YEKEDGIR.......YIATLHPAYL..................LRLLS.....TDTE.....AYQRAKGL
tr|D1JDZ5  .......FTVSTIAGIKR.......IIPLYHPAVV..................TY........NP.......KMKE....
tr|D1PN05  ..............RPVL.......ILESAHPSPL....SAYRG.........FF..G.....SA.......PFGK....
tr|D1Y5H9  .......HQFTWNGRRMT.......LRPLFHPAYL..................LR.................NPRRVEGS
tr|D2EF83  .......IDISLNGKKFK.......CLVTFHPSFP..................MRFPS..............ARKA....
tr|D2Z573  .......HHGRLGGMDLT.......IRPIFHPAYL..................LR..N..............RSREEGSP
tr|D3B454  ..............K.HH.......VIESGHPSPL....SV.R...T......FM..G.....TK.......PFSK....
tr|D3E6H2  EGPVQQAADPENPGAGYVFSPETYKIFPLFHPAAV..................LYNRK.....LS.......TLIE....
tr|D3FBP7  .......EVHVLGRRAVR.......IFPIFHPAAA..................LYTPS..............MLDT....
tr|D3QZ35  ................HL.......YLTASHPSPL....SAYR...G......FF..G.....CG.......HFRL....
tr|D4G8B4  ..............K.HY.......VLESTHPSPF....SANF...G......FF..G.....CK.......HFLK....
tr|D5C4I1  ...........IEVKNLP.......TFVTYHPAAA..................LRDET..............KKKT....
tr|D5E730  .........................VVKFYHPAAL..................IYTKS.....RQ.......PEQQ....
tr|D5EGK6  .......YPWDFEGRKIK.......IRPLFHPSYL..................LR..N..............RSREKGRP
tr|D5EHP1  ...........HDFNGTD.......LMITFHPSYL..................LR..N..............DTMKTKRL
tr|D5MIW4  ...........HDYHGIL.......VLPTFHPAYL..................LR..N.....PH.......EKKT....
tr|D5RKP0  ..............R.HR.......LLESDHPSPL....AAAQ...AARSASPFA..G.....SR.......PFSR....
tr|D5U040  .......FEINVYGVKTI.......SIPTFHPAAA..................LYNPRLRTILLE.......DFTKVVKP
```

```
tr|D5U570  .......HYYSNNGKDVP.......VIATYHPAYI.................LRNQK..............EEDK....
tr|D5V6V9  .............K.HH.......IITAPHPSPL....SSYR...G......FF..G.....CK.......HFSK....
tr|D5WWR6  E.............REFL.......VAPCYHPSAI.................LR..D.....PR.......KYPD....
tr|D7CKV6  ...........FKRGDVW.......MMAVYHPAAL.................LR..D.....PR.......LKRP....
tr|D7CWJ9  ...........FSWHGVR.......VFPMFHPAYL.................LR..N.....PSR......ERGSPKWL
tr|D7UXZ6  QFWNQTKEQYDWTEKDYT.......VIPTFHPAAI.................FYNRK..............LLPL....
tr|D8LFW4  .............K.HR.......VITCAHPSPL....SATR...K...PDPFI..G.....SE.......CFKK....
tr|D8UI66  .............K.HH.......VLKAAHPSPL....SASR...G......FF..G.....CR.......HFSE....
tr|D9Q1V3  .......VKVSIGGVELT.......VIPTYHPAAA.................LYKPD.....LK.......DYLA....
tr|D9QQQ4  ...........VEREGYY.......LLPTFHPAAL.................LR........NE.......QWKK...P
tr|D9S3H2  ...........IYKRGFW.......IMPTFHPAYI.................LRQGG.....E........TLKRVKWQ
tr|D9SCW5  ...........HDYKGIP.......LVVTYHPAYL.................LR..S.....PL.......EKAR....
tr|D9Y2E5  ...............CL.......ILESNHPSPL....SAMR...G...PIPFI..G.....ND.......HFRK....
tr|E0S9I8  .............K.HL.......VLCCGHPSPL....SSNH..........FF..G.....CK.......HFSK....
tr|E0SQC9  .......YNMKIFDLTVY.......VIPTIHPAAG.................LYNPN.....LK.......NLLI....
tr|E0TBI7  ......REWPLSDGRQAA.......VLPMFHPAFL.................LRAPA..............QKRL....
tr|E0TIS2  ...........YYYYNIP.......LISIYHPSYL.................LR..N..............NFII....
tr|E1IBI3  ..........................HTPLRHPAQG.......................G.....AA.......QFAQ....
tr|E1L0U9  ...........AAARNVC.......VLRSTHPSPF....SALR...AAGRVPAFM..G.....SR.......PFSR....
tr|E1QFX8  ...........HEALGAR.......VLPTFHPAYL.................LR..S.....PQ.......RKGQ....
tr|E1QNN8  .......FNVKLGDLNVK.......VLPTYHPAAA.................LY..N.....PR.......LRGV....
tr|E1QWE2  ...............TH.......IVASTHPSPL....SARR...GTRDLPAFM..G.....SR.......PFST....
tr|E1R614  ...........FEYRGIP.......LVPTYHPSGV.................LR..N.....P........EYRA...P
tr|E2IUG8  ..............HL.......VLRFAHPSPL...........S..QTP.FG..T.....CR.......HFLL....
tr|E2SYG7  ........RTNNPWMHRS.......LIPVYHPGQR.................AMVHR..............SFAN....
tr|E3D056  ...........FSWKGMS.......VLPMFHPSYL.................LRYPSSTEGSPK.......HL......
tr|E3IP47  ...............HL.......VLHAAHPSPL....SAFR...G......FF..G.....CR.......HFSQ....
tr|E4KQ36  ...............H........IIKSAHPSPL....SAYR...G......FI..G.....SK.......PFSQ....
tr|E4RKG8  ...........LKRGDIY.......FLATFHPAYL.................LR........NE.......NMKK...Y
tr|E6N689  ...........FEKMGRR.......FFCTYHPAAA.................LY..N.....PG.......NRET....
tr|E6PFU2  ..........ERGPLDIP.......LLVTFHPAYV.................LRQTG.............GELTAVKRL
tr|E6W1H1  ...............HH.......ILTAPHPSPL....SAYR...G......FL..G.....CG.......HFSR....
tr|E6W552  ...........LHYGDIQ.......VVVTYHPSAL.................LRNEN..............LRRP....
tr|E7A9L4  ...............HL.......ILSAPHPSPL......AQ...R..KPPFFL..G.....SG.......IFSR....
tr|E7FX98  ................Q........FIESSHPSPL....GAYR...G......FI..G.....SR.......PFSK....
tr|E7H418  ......HLAQLPDGRQVP.......AVATYHPSYL.................LRSPA..............AKSK....
tr|E7H4Y1  ...............HL.......VLASSHPSPL....SATR...G..DDA.FM..K.....SD.......CFHK....
tr|E8LLT1  .............K.HL.......VLECAHPSPL....SANR...G......FF..G.....CH.......HFSR....
tr|E8N338  ...........FWRNGRL.......IVPMFHPAAA.................LHQPSLKASVER.......DFAR....
tr|E8T398  GVEVT.....................VIPTFHPAYL.................LR..N.....RR.......GEPE....
tr|F0EKF3  QESNPTKDGYQWSEKEYL.......IVPMYHPAAI.................FYNRT..............LTPQ....
tr|F0NSC6  LELNTAKDGYVWSKKKYT.......VIPEYHPAAV.................FY........NR.......KLSDDIVK
tr|F0RSJ4  ...........LRYEDVP.......VVVTYHPAAV.................LRNME..............LKRP....
tr|F0SG07  ...........YEYGRSQ.......VLCTYHPSYL.................LRNPS.....AR.......SLVR....
tr|F0Y985  ...............R.........VVASSHPSPL....SCAK...T...AAPFR..G.....SR.......CFSR....
tr|F2LXL6  ...........FVYGNIK.......VIPLYHPAYL.................LR..N.....PK.......AVDV....
tr|F1A0X7  .............H.HF.......ILESGHPSPL....SV.T...H.......FK..G.....CK.......HFSK....
tr|F2NFM2  ...........QQWRGIA.......VMPTFHPSYL.................LRVPQ..............ERVK....
tr|F3KRD3  ...............HL.......VLTANHPSPL....SALR...G..PQP.FL..G.....CG.......HFSQ....
tr|F3QMU0  GKPRK.....................VIVTYHPSYL.................LR..S.....PQ.......EKRK....
tr|F4A8X0  ................YYNVILKRNIFPMYHPSAL.................TYNRN.....D........KFKTMYED
tr|F4C0J7  .............LSDR.......FLVTYHPAAV.................LRNRN..............LMAD....
tr|F4GMI7  ADQDIP....................VVVTYHPTAL.................LL..R.....PQ.......HKPD....
tr|F4GSY3  ...............VL.......VLKANHPSPL....SALR...P..PRP.FM..G.....CG.......HFSK....
tr|F4HNK0  FGKLY.....................IIPMYHPAVA.................LY..R.....PQ.......LRKE....
tr|F4NZD5  .............K.HL.......VLKATHPSPL....GANKG..G......WF..G.....CK.......HFSK....
tr|F4QWL4  .....REWRLSEDGIAAP.......VMATLHPAFL.................LRQPQ..............AKGL....
tr|F5SL17  ...........VERKGVR.......MMPTFHPAYL.................LRNPE..............AKRE....
tr|F5Y215  ...........HQYHGVP.......VVVTYHPAYL.................LRNLP..............DKAK....
tr|F5YGI3  ......TPYHVHETLSIP.......VLATYHPSAL.................LR........DD.......TYKR...P
tr|F7NHV1  ..........FQTKYGIP.......AIATYHPAFL.................LRLTG.....K........ELVKAKWE
tr|F7PTA1  ...............HL.......SIHSPHPSPL....SAYR...G......FF..N.....SK.......PFTT....
tr|F7S094  .............SIHRKS...SLVLNSVHPSPL....SAHR...G......FL..T.....CG.......HFRA....
tr|F7XVS9  .............YSNCYLGSNTTEATAIFHPAYL.................LRQPA..............QKKA....
tr|F8ADN2  ...........HKLDDIK.......VLVTYHPAYL.................LRNNS..............QKSA....
tr|F8L559  ..............HPHV.......VLKAAHPSPY....SATE..........FF..G.....CR.......HFSK....
tr|F9CXI5  ...........VKKDKQI.......YFLTVHPAAT.................IYNQE..............LITV....
tr|F9DTW2  YLKSLNDTHYSWTDHSYT.......ILPTFHPASI.................FYRPS..............LRTD....
tr|F9LK36  VVLDNNYNNYTRSEKEYV.......VFPFYHPASV.................FYNSS..............LKKV....
tr|F9ZLD4  LDLCHPRLKPFFEQRAVR.......VFAIPHTSPL.................SW.....................NRNAPDGR
tr|G0LJW8  .......TDIRLGDQSYR.......VIISVHPAAT.................LY.....................DQSQ....
tr|G0QBL8  ...........FEREGQS.......IVPAFHPAAT.................LYDPS..............KRTE....
tr|G0QFK7  ...........FSRKGTK.......IMPILHPAAA.................LY..D..............QSKT....
tr|G0U4E4  .............R.HT.......VIECAHPSPL....SA.A...K......WW..G.....SR.......PFSK....
tr|G2RZ19  ...............DY.......VISSAHPSPF....SYHK...G......FQ..N.....TK.......PFSK....
tr|G4RL69  .......FRGKIDDVETT.......ICVTYHPAAA.................LY..N.....PR.......LRRS....
tr|G4STH7  ...........HSLDGIP.......LIVVYHPADL.................LR..S.....PL.......EKRK....
tr|G4T6H9  .............K.NK.......ILKSVHPSPL....SANK...G......FL..G.....CN.......HFKL....
tr|G7WNS9  ..........................FLVTFHPAAV.................LR........NG.......NLRE....
tr|G8BJ42  ...........DGRQ.FL.......VLKTVHPSPL....SAHR...G......FF..N.....SQ.......VFKI....
tr|G8LWR8  .......NKVVVLNKEYK.......LYPLYHPASI.................IY........NA.......ALKE....
tr|G8TXV8  ..........................AVALYHPAYL.................IYRRA..............FWPQ....
tr|G8XT11  ...............HL.......VLKSCHPSPR.......N...T..QKS.FV..G.....ND.......HFTL....
tr|G9EIR0  ...........HYYKNTP.......FIVSYHPAYL.................LR..K.....PA.......DKKK....
tr|G9XY09  ...............SN.......IIKGAHPSPF....SY.N...L......FK..D.....QQ.......FFVK....
tr|H0E6E4  .......EIRTIGRSTVR.......LLPLFHPAAA.................LYQPS.....SV.......ELLR....
tr|H0KUY0  ...............HH.......ILASAHPSPF....SVYR...G......FY..G.....SK.......PFSK....
tr|H0Q5N1  ...............HT.......VIQSSHPSPI..GGACNK...G......FF..G.....SR.......PFSR....
tr|H1D376  ...........IDYHGIP.......VMPTFHPAYL.................LRQSG.....R........SLVDAKWQ
tr|H1P1U4  ..............AFE.......LFCLPHPSAQ.......................G.............LLQAAPDK
tr|H1P505  ...........QRLDGIP.......VMPTFHPAYV.................LR..Q..............YTQDVRRA
tr|H1XWX4  ...........HQYQNIP.......VIVTYHPAAL.................LR..N.....PA.......LKQK....
tr|H2C8Z4  .......FKLKTGFGELL.......IYPTYHPAAA.................LY..N.....PS.......LMRE....
tr|H2J7L7  ..........KEWYGDIY.......IFPTFHPSYL.................LRNRSEKEGSPL.......FYAK....
tr|H3KDP1  ...............HL.......ILTASHPSPL....SVTR...G..PEP.FL..G.....CG.......HFKK....
tr|H3NN78  .............N.NY.......ILTSTHPSPL....SAYR...G......FL..G.....CR.......HFSK....
tr|H3NSX2  .............H.NL.......VLTAPHPSPL....SAYR...G......FF..D.....CQ.......HFSQ....
tr|H3RRL7  ..LHCLIQRAASDHQGYVMSNQSYTVIPMYHPAAF.................LYARK.....LE.......PIVR....
tr|H3SCZ2  ...........VEKDGIR.......IMPTYHPSAV.................FRDEQ.....KR.......ELLK....
tr|H4GIS2  LEFNSATGQYQWSQASYQ.......IVPMYHPAAV.................LYRRQ..............LMPT....
tr|H5SI33  ...........LREGGRW.......WFATYHPAAA.................LRSAS..............LANA....
tr|H6RLZ5  ............EWEGRG.......LYVTYHPSAA.................IR.FG.....PN.......GAPRAALA
tr|H7F338  ELSSLSLNTFQKTDRKFR.......MMATFHPASI.................FY..N...............RKLLPL
```

```
tr|H8I7F5  ...........IEKGGIK.......YMITYHPAAM..................IY..N...............QWLG..DT
tr|H8ZDF2  ..............K.HL.......VLCSPHPSPF....SART...G......FF..G.....CN........HFQK....
tr|H9UI22  ...........HRIKGIP.......AVVTWHPSAV..................LRNQD...............LRSE....
tr|H9ZZZ0  .......GNFKAYDRSFQ.......TYATYHPAMI..................LR..N.....PR........LYEV....
tr|I0GI60  ...........FEKDGLK.......FFVTFHPAAA..................LY........HG........EYKE...L
tr|I0I6B7  ..........RVVEDGRV.......VVAMFHPAAA..................LRNPSWRVAFER........DMAR....
tr|I1A005  ...........RNYNKDN.......LFKLSHPSPL....GYRH...S......FE..N.....SN........VFKK....
tr|I1B0L2  ..............G.H.......LVLRSAHPSPL....SAYR...G......FF..G.....SR........PFSR....
tr|I2K0D9  ..............Q.NL.......FLKSVHPSPL....SASR...G......FF..D.....CH........HFXX....
tr|I3CUN9  ...........HRYQDRP.......LVVTYHPAYL..................LRTLT...............DKSK....
tr|I3VQA4  ................HL.......ILKSSHPSPRV.....TS...T..RSP.FV..G.....NG........HFAT....
tr|I4CBR6  ...........YEVEGIP.......VIPTYHPSFL..................LREES.....DK........RFKA...M
tr|I7LIA9  ...........VNLDNKK.......IFPLYHPASI..................IYNRS...............LYDV....
tr|I7LKX1  .......YSCSLFGHPVR.......LIPTLHPAAA..................LY..N.....PA........YREA....
tr|J0UVA7  ......LSYTDTLGRSVP.......LVVTYHPASL..................LLRPQ...............HKAN....
tr|J5SPB4  ..............R.HL.......VLKSAHPSPL....SASR...G......FF..G.....NG........HFVK....
tr|J7R1V5  ...........KNFPNLL.......VLKSVHPSPL....SASRG.........FF..G.....NL........HFKQ....
tr|J9E0C3  GRKIP....................ALPILHPAYL..................LR..N.....PA........RKAE....
tr|J9Z1B8  ..............K.HL.......ILTAPHPSPL....SAHR...G......FF..G.....SK........PFSK....
tr|K0IF13  ...........VERAGQK.......YFLTFHPAAA..................IYNKN...............LLSA....
tr|K0KF39  ..............K.IH.......VLTSVHPSPL....SARR...G......YF..E.....AN........HYIK....
tr|K1JXC9  DFDTK....................AVVSYHPSYL..................LRAPD...............EKAK....
tr|K1JYF3  ................HL.......VLCANHPSPL....SALR...P..PVP.FI..G.....CG........HFGA....
tr|K1LNP2  ................HQ.......IFTSSHPSPL....SAYR...S......FF..G.....SR........PFSR....
tr|K1ZRE1  .......IEAKVPFGKTK.......IIPLYHPAVV..................LYSAG.....SR........KVLE....
tr|K2A3B6  .......VTMPWRGREIV.......VVPMYHPAAA..................LRSGE...............VMLA....
tr|K2B4F4  ......KRYKGIDGGKQV.......YYPMYHPAVA..................LY........NG........SYRE...I
tr|K2C359  ..........YDFGEGIK.......LMPLFHPSYL..................LRNPSKEPGTPK........HHMY....
tr|K2CLQ5  ..........IDTRGGVK.......YFLALHPAAA..................IRFAK...............NMPL....
tr|K2DEQ6  ...........FRSQGYN.......IFPVYHPAAA..................LR..N.....PE........MAMA....
tr|K2E7J1  ................NL.......VLISAHPSPY....SAQL..........FF..G.....KH........HFSL....
tr|K2E9B4  ..........FDGPFDSK.......IMPIFHPSYL..................LRYDS...EEPD........TPKW...L
tr|K2ETW7  .........YQYGQQKTP.......LLVTYHPAYL..................LRSPS...............EKRK....
tr|K2F640  .......YNVSWNGKNIV.......VVPMYHPAAS..................LRNGN...............VMEA....
tr|K4KIT8  ................HR.......VIKTSHPSPL....GATK...AGRDFVAFL..G.....SD........CFGE....
tr|K6GHM9  ................NS.......ILMAPHPSPL....SSYR...G......FF..G.....CN........HFSK....
tr|K7YPM8  .....MNWTSPNYQETIP.......ALATYHPAYL..................LRTPS...............QKAS....
tr|K8ZAC1  ................HY.......VIATPHPSPL....SAYR...G......FF..G.....SH........PFSK....
tr|K9VTI9  ...........MKWNRRF.......YMPILHPAYL..................LR........NH........DYSK..DG
tr|L0A9A2  .......REVMFNGTKIK.......ILSTYHPAAA..................LYKPE.....LK........ELLR....
tr|L0B4R8  ................HL.......ILKSNHPSPL....SAYR...K..PFP.FI..G.....CN........HFIK....
tr|L0B866  ................HL.......ILKANHPSPL....SASR...G..PLP.FI..G.....CN........HFKI....
tr|L0B8A6  .......HGFKSKGSSIP.......VVVSYHPAYL..................LRRPE...............EKSL....
tr|L0DFI2  ...........HRCRGVL.......TLVTYHPSYL..................LR..N.....PA........AKKD....
tr|L0L178  .........................LVKLYHPAAL..................LY........RR........SLME....
tr|L2GQC8  ................HL.......ILEAGHPSPF....SV.K.........MFE..G.....CK........HFSK....
tr|L2GX11  ..............K.HL.......VLMSTHPSPF....SADR...G......FN..G.....CN........HFLK....
tr|L5N8A0  YRPKEGGPRWCETEKAYR.......VIPLYHPAAV..................FYNPK...............IKPT....
tr|L8HA23  ..............K.HH.......VLTSAHPSPF.....AAH...N......FF..G.....NK........HFST....
tr|L9X1W2  VLRTSSSSNSSSGAGTDPRAQTVRTVRPAFHPAAA..................LYDRS...............KVDA....
tr|M0BKB2  .........................LLPILHPSYQ................DVWISRMG..............YERA....
tr|M0CBA6  ...........YEVDGQP.......VLPTYHPAAT..................FYDDG.....VG........PMLE....
tr|M0MGS7  .........................LVPVLHPSYQ..................AV..................WLSRLDYT
tr|M1E4Q4  ...........ILQDNRY.......YIPMFHPSYL..................LRNAS......R........SIGSPRWL
tr|M1LNF1  ................HL.......ILKANHPSPL....SANR...K..PIP.FI..G.....CN........HFIN....
tr|M1QKH3  ...ILANLKSPVLAPGTK.......IFATYHPSYL..................LRNPN...............ELSN....
tr|M1VCA8  ..............R.HL.......VLQAAHPSPM....AAARG..G......WF..G.....CN........HFRK....
tr|M1Z9H8  NI.............NYK.......VFPLYHPAAV..................IY........RR........ELKD...T
tr|M2XVU5  ..............R.HH.......VLIAAHPSPY....SAAN...G......WF..G.....CR........HFSK....
tr|M3EFG6  .........................SLPT.HPYWI......................................RKFE....
tr|M4YU22  ..........TVGGKEIL.......FIPTYHPMAC..................VY..................QKPA....
tr|M5ADY8  ................HY.......VLKTSHPSPF....SAHM...G......FF..G.....SK........HFEK....
tr|M5IVB6  ................YL.......ILKANHPSPL....AARR...P..PVP.FI..G.....CG........HFQQ....
tr|M6D8H9  ...........GDFHGIP.......VMPTYHPSYV..................IR..N..........GGENSPLKR...D
tr|M7WR77  ..............K.HL.......ILTSAHPSPL....SARR...G......FF..G.....NS........HFRK....
tr|N0BBN4  .......YEVEVEWGKMK.......VIAVYHPAAA..................LY..K.....P.........PLRE...V
tr|N1V4N6  .........................APKVRHPAHG......................G................KVQ....
tr|N1Z687  ..........TKTKDGYQ.......LFPLYHPAAI..................IY........NR........SLSE....
tr|N2BLL9  ................HA.......IITAPHPSPL......AR...G......FV..G.....SG........VFLQ....
tr|N9SHT1  ................QK.......VFFYSHPSPF....SFAK...S......LK..H.....SD........VFKK....
tr|N9V498  .........................IIYTSHPSPL..................SYART.....NQ........SFKD....
tr|Q025I0  ...........FKWQDIP.......VMVTYHPSYL..................LRPFN.....EN........AKRE....
tr|Q0C0Y4  ......YCYTTPGGYRVP.......LIPMLHPAYL..................LRRPQ...............DKSR....
tr|Q0EZR7  ...........HEYHGIP.......VWVTYHPAYL..................LRSPQ...............QKQK....
tr|Q12TN4  .........................VLKIFHPAAL..................IY..T...............RSRT....
tr|Q1AV32  .........FESKILGLP.......ALATYHPAYL..................LRVRGAGGADYG........RLRR....
tr|Q1AYQ1  ...........YEVGGIR.......AMATFHPAYI..................LRQSGE..............ELARTKRL
tr|Q1GY28  ................HQ.......VISSAHPSPL....SAHRG.........FF..G.....SQ........PYSR....
tr|Q1N1I3  ................HL.......VLKTSHPSPL....GATK...SGKDFTAFM..G.....SG........CFSE....
tr|Q23W06  ..............K.HH.......VLATSHPSPL....GYMK...G......FD..K.....AN........CFSA....
tr|Q3SA81  ............YRGDII.......IIPMFHPAAA..................LYHGK...............WKPE....
tr|Q53CZ3  ................HL.......VLKARHPSPLAAAHAATG...S..PWPQFL..G.....CN........HFKL....
tr|Q5DC15  ..............R.HL.......VLNAPHPSPL....SASR...G......FF..G.....CG........HFIK....
tr|Q5WES9  QLPSLESSKLVRGERQQH.......VFPTFHPASV..................FY..N.....PT........LATA....
tr|Q6CAC0  ..............K.HL.......VLKAVHPSPL....SAHR...G......FL..T.....CG........HFKQ....
tr|Q6F1M3  ................NN.......VINSAHPSPF....SYLK...G......FK..N.....SK........PFST....
tr|Q6KIL8  ..............TKHF.......ILESSHPSPL....SFYK...G......FF..K.....NN........HFVK....
tr|Q7VBS6  ...........HNWNGIY.......AMPIFHPSYL..................LR..................NPSKTIGG
tr|Q8TK31  .........................ILKLYHPAAL..................IY..................TASK....
tr|Q9RTK9  ............FTYRHPAWPQPALLMPLLHPAYL..................LR..NPVRTPGG........PKSL....
tr|Q9WR44  ................HL.......VLTYGHPSPL...........S..RSE.FR..V.....CK........HFLQ....
tr|R1ATM3  .......QKVCINNKEYI.......LFPLYHPAAV..................IY........RK........ELKE...V
tr|R1E4B2  .......FKVNKWGKNIT.......IIPTLHPAST..................LY........HK........EWRE...Y
tr|R1FRL1  ..............R.HK.......VIEAAHPSPL....SVTK..........FR..G.....CR........VFSK....
tr|R1GL12  .....LTYVSPIDQRSIP.......AFAFYHPAYL..................LRSPG...............QKRT....
tr|R2SFV7  ..ITSPVLELNADQDGYQWGNHVYTLVPMFHPAAI..................FY........NR........KLAPLIEQ
tr|R2SXC9  LAYSNETNKLAKTSEEYM.......LFPIFHPAAI..................FY........NQ........KLESAIEY
tr|R4KRR4  ...........FELEKYI.......VFPMYHPAAS..................IYRRA...............LVEV....
tr|R4VE87  .........MAWGEATIP.......LRVTYHPAYL..................LRYPG...............EKAA....
tr|R5ARL3  .......LPVKWEGWAVT.......LFPLYHPASG..................IY..N.....PA........LVPV....
tr|R5B4U8  .......MNVNIGGNNIA.......LFPLYHPASG..................IY........NR........ELVE....
tr|R5DX78  .......MTINVDGKDYR.......AITIFHPSYL..................LRNHSLDEGSPR........RLMK....
tr|R5MPG0  ................NY.......IIESVHPSPL....SCYR...G......FF..G.....SK........PFSK....
```

```
tr|R5PZ23  .......LTYASENTELP.......AMPSFHPAFL..................LRSPA..............QKKA....
tr|R5QX00  NGQKAM...................VLASFHPAYL..................LR..T.....PG.......QKAK....
tr|R5Z1D7  ................HL.......ILTSSHPSPL....SAYR.........GFF..G.....NK.......HFIK....
tr|R6HGV3  ................KY.......ILRSTHPSPL....SANK...QAGELRAFM..G.....SR.......PFSN....
tr|R6HL97  ................DY.......IISSPHPSPL....SAYR...G......FF..G.....SK.......PFSK....
tr|R6PFR8  IE.......................VMAIFHPSYL..................LR..N...............HSTEQGSP
tr|R6PI86  ..........FDGPYGSK.......MMPIFHPSYL..................LR..N....ESR.......AKGSPKWL
tr|R6R865  ................HL.......ILTSAHPSPL....SAYH...G......FF..N.....NN.......HFKK....
tr|R7BZ83  ................IL.......ILTSNHPSPL....SALR...G..PAP.FI..G.....CG.......HFRK....
tr|R7DQ64  ................RL.......ILESTHPSPL....SANRG..G......FF..N.....QH.......HFSK....
tr|R7DXQ1  ...........HDLNGIP.......VRVTYHPSYL..................LRSDS.....PR.......EKRK....
tr|R7I8P1  ...........FERKGIW.......MMATYHPSAL..................LR..D.....PS.......KKRD....
tr|R7IAS2  .......HETEVGGKKIP.......VIVTYHPSYL..................LRNLA..............DKEK....
tr|R7IHQ8  ...LTAHVQADKQAGDFP.......LFALYHPASI..................IYNQK.....LK.......DVYQ....
tr|R9ADN8  ..............K.HL.......ILSSVHPSPL....SASR...G......FF..N.....TG.......HFKQ....
tr|R9LKN0  ..............N.HL.......ILESAHPSPL....SVSR...G......FF..G.....SN.......CFKK....
tr|S0EV15  ..........FEGRYARY.......VAATWHPAYI..................LRLQG.....D........AYEAARRE
tr|S0FG57  .......HTCNIGEKAYR.......LFPLYHPASI..................IY........NR.......SLKD...T
tr|S2J4F7  ..............K.HY.......VLTSVHPSPL....SAHR...G......FF..E.....CK.......HFSK....
tr|S3JT40  ...........FDYQGIP.......LMPTYHPSAL..................LR........DE.......SLKR...P
tr|S4NBY8  QKYNPETNSFEFTQEKYR.......IFPLYHPSYS......................K..............RFKNMKAI
tr|S5MFT9  ................EN.......CIFSSHPSPF....SYKN...G......FE..N.....SK.......PFSK....
tr|S5RQ34  ..............K.HY.......IIFSSHPSPF....SANK...G......FF..G.....SR.......PFSF....
tr|S6G4W1  .....YEYTNSFLSRSIT.......AMVIFHPAYL..................LR..Q.....PQ.......QKRL....
tr|S9VE50  ..............R.HI.......VIENGHPSPL....SV.K...H......WV..G.....CK.......CFSK....
tr|T0AYQ1  ..............C.HC.......ILKSAHPSPL....SASR...G......FL..G.....NN.......HFIK....
tr|T0DRC9  ................HI.......IITAPHPSPL......SR...G......FL..G.....SG.......VFTS....
tr|T0LSQ5  ...........FEWNGTL.......IFPQFHPAAI..................LRDIK..............RLDK....
tr|T0MGQ8  ..............R.HL.......VLESAHPSPF....SAKY..........FF..N.....CK.......HFSK....
```

```
                 210                   220
                   .                     .
sp|P12295  AN....QWLEQ.R..G......E.....TPIDWMPV...LPAESE..
tr|Q8KLI8  TW....LDIQEVKRAL....DALPPKERRPVKAVSQEPLF.......
sp|A1QYK3  TL....KYLKE.H..N......K.....ISIDFQ.............
sp|A1SUE5  VN....DILAE.R..R......E.....QLIIW..............
sp|B0B8I2  IN....YLLKK.Q..G......K.....TMINWKIE...........
sp|B1AJI8  CN....DYLIK.H..L......R.....TPIKW..............
sp|P12888  AN....NFLRE.K..G......L.....GEIDWRL............
sp|Q057V4  TN....VLLQN.Q..K......K.....KPINWFLD...........
sp|Q315T1  AN....SWLAA.R..G......L.....LPVLW..............
tr|A0LFG4  AW....EDLILVRDLL..KTGCVQDGGIGREGIKPSP..........
tr|A0LH32  ......EKSSALRRAF............RQVAVRPE...........
tr|A0LRS0  LL....QDLALAAAVAGHRRDTDAGPGAVPYDGDR............
tr|A0RV64  FR....RDMKKLAGMI............LST................
tr|A1BDC5  CW....EDVQMMRQHY............DKLCSAEAHSRTMVTNNDQ
tr|A1RYC0  LE....EDFRFLRGLL......EGGGQRTLDSWLG............
tr|A1US05  TW....IDFLEVKNRL............NNLS...............
tr|A4GHT2  AN....QYLQE.K..G......K.....KPICWTN............
tr|A4HXS3  CN....AALQS.M..G......H.....LPMHWQLP...........
tr|A5KS51  LI....DDFSRLPKIV............ELIHKERK...........
tr|A5UQ47  AE....AFIAAMQPLV............DGRNEP.............
tr|A6DBB2  FF....KSLYQLTMEN............GQKKIMK............
tr|A6DK00  IN....KYLES.T..K......Q.....EAIDWRLE...........
tr|A6GN76  AW....DDLLLLKQQG......VI........................
tr|A7ANQ0  CN....EYLRN.T..K......Q.....KPIDWMLP...........
tr|A8A982  ......QFVSDLKKAF............NEVYKQRSLFDSG......
tr|A8E1M5  AN....EYLSR.R..G......E......RVDWNVL...........
tr|A8F067  SW....YDLLKIKEYL............VNNKLILN...........
tr|A8MA01  ......EDLAKAFNAS........KGGSSILDYLNG...........
tr|A8SNH5  AN....EFLVS.K..G......V.....KPIDWRV............
tr|A8UAT5  IH....EDLAEFKKLI............HKI................
tr|A8UU74  FT....EDMERLKELI............GL.................
tr|A9AZ06  AI....ADIKLIPRLI............ARLNQRLQEETNETTEPNL
tr|A9BHU0  KW....YAYLDMRIIGIMYRALKQGKDVEEVTKTVE...........
tr|A9F5H8  VW....SDLKEVMRHL............GKSAPDRG...........
tr|A9NHD4  AN....HYLMM.H..H......V.....NPVDWHIK...........
tr|B0VFE1  AW....IDLQEFQKEY............EKLLAATIS..........
tr|B1GA08  AN....EYLVQ.H..G......R.....TPIDWRLP...........
tr|B1H0K1  VW....EDMKKVMHYL............DTGEI..............
tr|B1I4W5  FV....RDIREAVGLA............LALRWPAFLSAYYHSGLSG
tr|B1VA82  TN....LYLKS.H..N......I.....QEINWQLH...........
tr|B1Y7I1  AA....GWLAS.M..G......R.....GAIDWHL............
tr|B1ZZ66  IW....EDLLKVMERA......GLPIAEKQRGYFLDK..........
tr|B2A668  SW....EDLQNLKAKY............MELTNNDID..........
tr|B2KCZ8  AW....ADLQMVMKEM......GLKSPQKPI................
tr|B3DV47  VW....EDLLTAMGKL......GMPISEKQRNYFLS...........
tr|B3U4N1  VN....RALTE.A..N......L.....KPIDWQIP...........
tr|B3U4U7  LR....EDFQKLKEFL............DRHSQPP............
tr|B5II11  TW....QDLQDVRRRL............DQGGTRRSGGGL.......
tr|B5LRM7  AN....EYLEG.R..G......V.....EPVDWQLP...........
tr|B5Y8I5  ......HDIQMMSRLI............AGE................
tr|B5YEZ9  IR....KDFEKLKELYELTLKSEEEQKETNKKEKE............
tr|B6A3R0  ......DFVSDLH...............KAAAFRPR...........
tr|B6AFI4  CN....DFLRELNR..............PEIDWVPT...........
tr|B6K2R2  TN....QWLKE.KFGE......E.....YCIDWGAV...........
tr|B6KW10  CN....ELLRSMGKAE..............IYFASS...........
tr|B7FQS1  CN....QALTE.M..G......C.....GAIDWNVD...........
tr|B7KM64  TW....QDLISLKRKL............NKLTTKLHQ..........
tr|B8C427  AN....AALIA.Y..G......K.....GPVDWHVR...........
tr|B8CX81  VW....RDFKVIRKAV............DRIKELKKTGEL.......
tr|B8EJL3  AW....QDLRMLARAL............ADMGEDAPKTPGETPDARA
tr|B8HFW5  LE....QLLLK....................................
tr|B8I5K9  YH....EDMMVLKKEI............KKLSTVNVFNPNKRVFHIS
tr|B9KXL9  ......KDFDALREII....AQARAEAARTVEAQQSEE.........
tr|B9XNJ8  IK....EDFALLKSEM............KKL................
tr|B9Z7Q7  AN....AWLAA.A..G......R.....GVIEWAPA...........
tr|C0BNZ8  VN....NILEQ.R..S......E.....KPINWLI............
tr|C0X269  DW....QVVQKQLQLL............KKV................
tr|C1A7L2  QE....TWMQQCVAAV......IEAGYPAPDTS..............
tr|C1D9B0  AN....DWLAE.Q..G......R.....LPVNWALE...........
tr|C1F1L1  AW....ADLQIAMREL.......GLKPQKPASKPAPASGQD......
tr|C1G9A4  TN....EWLSE.RYGK......D.....GIIDWSLV...........
tr|C2MIJ3  IY....EDLEKLKQYV......IKN.......................
tr|C3X2T7  AW....QDLCRALDIY............DEEKKREE...........
tr|C5CIT9  TW....EDIQKVRKMY......DQVINGREISLED............
tr|C5NVK4  IN....DYLDS.H..G......R.....GNIDFELK...........
tr|C7H0A3  CN....NYLEG.N..G......K.....GQIDWRV............
tr|C7M312  LR....SDLARASVAL............AERRR..............
tr|C8W879  ......DDLQLLADWL............KKHPRGG............
tr|C9M5Q7  IL....LTLEDLKDVA......CRVSAPAQSKGNLS...........
tr|C9RLM9  AN....AYLVS.K..G......L.....EPIDWSIK...........
tr|D0MK10  AW....EDLQLLRAHY............DRLMGTATR..........
tr|D0PPD4  AN....TYLTR.H..N......R.....PLISWNL............
tr|D0U4F0  TN....DYLKI.H..K......L.....KTINW..............
tr|D1ART8  IN....EYLEE.A..G......K.....KPINW..............
tr|D1C3J5  FG....EFIDDLRTAG............ERAAVVSTD..........
tr|D1CDM9  VL....QDLKKAKMLA............SR.................
tr|D1JDZ5  ......VLIDDFKVLA............NTISRHI............
tr|D1PN05  VD....AFLQEHGE..............APIIWQE............
tr|D1Y5H9  PWDKMLRDLVEARRFL............AGQE...............
tr|D2EF83  FL....SDLSMAFNLY............KNLST..............
tr|D2Z573  VS....STLEDLRTLV............DFVRNTS............
tr|D3B454  TN....DYLKS.I..G......Q.....EEIDWTLQ...........
tr|D3E6H2  ......DDLDILARIL............GG.................
tr|D3FBP7  LR....ADFAQLPGLL......ALDPPEQPAAEVFEPVPEA......
tr|D3QZ35  AN....EFLIK.N..G......E.....SPIDWQLP...........
tr|D4G8B4  AN....QILKK.N..N......I.....KLIDWSLP...........
tr|D5C4I1  LE....HDFSKLQERL............ASAV...............
tr|D5E730  ......EFIEK.................NSALW..............
tr|D5EGK6  LH....QTLCDLREIA............SFLK...............
tr|D5EHP1  VW....EDMLKVMERI......GLEISDKQRAYFLPKN.........
tr|D5MIW4  VW....EDMKLLMREY......EQLGNTRPLASAPEFS.........
tr|D5RKP0  AN....DFLRA.Q..G......R.....DPVDWRL............
tr|D5U040  VI....EELDKTFTQP...............................
```

```
tr|D5U570  VI....SDLEFMLSEL...........EKIRNK.............
tr|D5V6V9  TN....EILES.L..G.....K.....KEINW..............
tr|D5WWR6  AV....ARWVDIRKRV...........EAAGLIL............
tr|D7CKV6  AW....EDFKLIRDKY...........QQLKRGELG..........
tr|D7CWJ9  MW....QDIRALKAAI...........DELGPKPAVFVVDTARQEG
tr|D7UXZ6  ME....QDWDKIAIYI...........GRKKDA.............
tr|D8LFW4  AN....DALEE.L..G.....H.....GGVDWNVL...........
tr|D8UI66  TN....KLLTS.Q..S.....L.....EPIQWRPS...........
tr|D9Q1V3  ......KDIKEAAEEA...EEPARARKTLLDFMGG...........
tr|D9QQQ4  TW....HDFLKIKKGY.....QRYLELKEEGKL.............
tr|D9S3H2  VW....GDFNKAIEKV..........RELSDYKFN...........
tr|D9SCW5  AW....QDLCLAVQTM...........DGCTG..............
tr|D9Y2E5  AN....EWLTQ.K..G.....I.....SPINWTKH...........
tr|E0S9I8  AN....KYLRE.H..G.....K.....SPIKWQI............
tr|E0SQC9  ......QDLKIVKECI...........DKMSTNPQDMTAKPKTLLS
tr|E0TBI7  AW....ADLRAVAARL...........AAS................
tr|E0TIS2  ......NKYIKSLLFI...........KNIKIK.............
tr|E1IBI3  ...........................GLREWRIR...........
tr|E1L0U9  TN....EFLRA.H..G.....V.....EPIDWRLP...........
tr|E1QFX8  AY....QDMKALALAL.....KD.......................
tr|E1QNN8  IE....EDLRKVKTLL....GKGEGGGGILDYLGS...........
tr|E1QWE2  TN....AILAD.H..G.....E.....EPVDWCTV...........
tr|E1R614  VW....KDLKALRSLL...........EELG...............
tr|E2IUG8  AN....QYLEA.R..G.....L.....PPIDWSLG...........
tr|E2SYG7  QL....SDYQFVAETL...........RRLKKPRKRPVSTKPRPDA
tr|E3D056  TW....LDIQEVKRKW...........DEVRGTDLCSEGEGQLS..
tr|E3IP47  VN....TWLRA.R..G.....L.....DPIEW..............
tr|E4KQ36  IN....NYLVA.D..G.....K.....DPIDWRLT...........
tr|E4RKG8  SW....HDFKLIKKAA.....DRIIELSSSGDL.............
tr|E6N689  FF....SDIKKARELL.....ESLRPFDKQKTLFEV..........
tr|E6PFU2  VW....DDLKAVRDRL...........DELRVSPPNAAARQVGLFD
tr|E6W1H1  IN....AILQQ.Q..G.....Q.....QPIDWGGS...........
tr|E6W552  VW....DDVRVLRNLY.DTARQEPLRFPAELKYPVA...........
tr|E7A9L4  AQ....NFLKQ....K.....N.....IPMRWDLD...........
tr|E7FX98  IN....ALLYE.R..N.....Q.....KEINWSVT...........
tr|E7H418  AW....EDLLLLKAAM...KQAGLTLPVREKHWD.............
tr|E7H4Y1  AN....AWLEA.R..G.....E.....APIDWMGA...........
tr|E8LLT1  AN....AYLHA.H..N.....K.....TTVNWQLP...........
tr|E8N338  LS....EYVQAARQRM.......QAAEKPIAVEEK...........
tr|E8T398  FQ....KDLELALRLA...........GFLK...............
tr|F0EKF3  IE....ADWRGLRNIL...........SQIPANE............
tr|F0NSC6  DW....EVIKPYIDKE...........RESNE..............
tr|F0RSJ4  VW....DDLKKIAAYL............DIQLSGRS..........
tr|F0SG07  ......DDLQILLKEM.......GIDPDRLVKKE............
tr|F0Y985  IN....AHLED.L..G.....V.....EQIDW..............
tr|F2LXL6  FI....EDLKKVKQLL...........EQ.................
tr|F1A0X7  TN....EFLES.K..N.....I.....EKIDWTIK...........
tr|F2NFM2  TW....EDMQQVLALY...........RPSKD..............
tr|F3KRD3  SN....SFLQA.H..G.....R.....GALTW..............
tr|F3QMU0  AW....LDWCLILDTL.....S........................
tr|F4A8X0  DW....LKLKEVLKTI..............................
tr|F4C0J7  FV....SDLREAKIQS...........LKLSKDAFAE.........
tr|F4GMI7  AW....RDLNLARTLL............AVGAGGA...........
tr|F4GSY3  AN....DWLRE.H..G.....E.....AGIDWITS...........
tr|F4HNK0  LE....EDFKKLKALL...........ASLGI..............
tr|F4NZD5  AN....EYLKS.K..G.....R.....GVVDWASI...........
tr|F4QWL4  VW....KDILDLSARL..........RGEVVEPGA...........
tr|F5SL17  AW....IDFQAIRDAY...........REVLSASR...........
tr|F5Y215  AW....ADLCLALELM.........AQQPAGRAAAAQ.........
tr|F5YGI3  AW....EDLKLLRQRL...........IEIDESYAREAGEGGV...
tr|F7NHV1  TF....YDLQAAANRCTEIEPDYLLKSAAPLDLLA............
tr|F7PTA1  VN....NFLIK.H..N.....Q.....DPIDWEIK...........
tr|F7S094  AN....KWLQE.Q..G.....K.....SPIRWLNT...........
tr|F7XVS9  MW....FDILKISKMI...........RPFISY.............
tr|F8ADN2  AF....EDLKLLKKLY...........GDLCKIS............
tr|F8L559  TN....EYLKK.W..G.....K.....TPIDWALN...........
tr|F9CXI5  LK....EDIIKLFDLI...........RELKNNK............
tr|F9DTW2  LE....EDWLRIGEYI...........QGLNYNALE..........
tr|F9LK36  IF....EDWQNLF..............RYIQFQV............
tr|F9ZLD4  KWS...EWAKEEVRVL..............................
tr|G0LJW8  ST....TFVDTIKSAA.....SLAGVQSDIDNPTDTTSGGQSR...
tr|G0QBL8  FE....DDLRKAFGRR...........DSGQTRLDQL.........
tr|G0QFK7  ......PDLKKDFKKA.....FGKQESGQKKLTDI...........
tr|G0U4E4  CN....VALSR.M..G.....H.....TPIDWNLP...........
tr|G2RZ19  IN....NKLTE.L..N.....L.....EIIDWSK............
tr|G4RL69  LE....GDLGKFLGGP........PDKGLLKYL.............
tr|G4STH7  AW....QDLKLAIATF...........DEIKG..............
tr|G4T6H9  AN....EWLEE.KYGE.....E.....AKVDWTAI...........
tr|G7WNS9  ......AFIADLRRGR...........GMAEGGAP...........
tr|G8BJ42  CN....EWLQK.H..N.....R.....DVIDWSVS...........
tr|G8LWR8  IYI...KDLQNLREII...........VSTESKT............
tr|G8TXV8  YR....DHLDRLAAFL...........REMGVSTDPASGPFLTMVR
tr|G8XT11  TN....NYLQE.H..G.....Q.....HTIDWNL............
tr|G9EIR0  TY....LDLLAVKKFL...........QQQNSV.............
tr|G9XY09  IN....NLLLK.N..K.....K.....NIIDWKM............
tr|H0E6E4  ......EDLARLPALL............ELPAPTP...........
tr|H0KUY0  IN....EYLKT.K..G.....K.....KPILWG.............
tr|H0Q5N1  AN....QALVQ.A..G.....R.....SPISW..............
tr|H1D376  VY....YDLLAAKEKA.......AALSPQWVWKSETMPNLLE....
tr|H1P1U4  GRGLKLKDLQDAWEQD.....LANLLHP..................
tr|H1P505  VW....MDLQAAKIWL...........DEHP...............
tr|H1XWX4  AW....QDLKKIKRFL.....DARTGESDSPHPLISPVPE......
tr|H2C8Z4  LE....SDFSKLKELL.....GGSIASKAITLDNFFDGNE......
tr|H2J7L7  ......IDMQAIGYMY...........KQLKNNPDLDIKKLVTTVE
tr|H3KDP1  AD....GHL............G.....TAIPWTGA...........
tr|H3NN78  AN....NILVD.L..N.....K.....DIIDWSTN...........
tr|H3NSX2  TN....AWLEA.H..G.....Q.....KPVDWLGN...........
tr|H3RRL7  ......HDWEAIAQKL...........DQLETDKI...........
tr|H3SCZ2  ......QDIFSAAEAL...........KKLEP..............
tr|H4GIS2  VE....ADWQQLSQLV..............................
tr|H5SI33  LQ....EDFRRLRRLV............DVEWGTD...........
tr|H6RLZ5  ADLRAVAYLVGARETR..............................
tr|H7F338  IE....ADFDHLSGWL...........KEVTK..............
```

```
tr|H8I7F5 IA....GDFKKVSALL......LEG......................
tr|H8ZDF2 AN....EYLES.K..G......K.....DPIAW..............
tr|H9UI22 VW....RDLQYMRDEL............MQLRSDYRPEIR.......
tr|H9ZZZ0 FK....EDLDMYLKS................................
tr|I0GI60 LK....NDFLKLKQIL............DETKIVG............
tr|I0I6B7 LS....MLIERVREAA......RRGEALSPEALN.............
tr|I1A005 IN....EKLHS.F..N......Q.....KEIEWSL............
tr|I1B0L2 VN....DWLIE.N..G......E.....TPINWTTP...........
tr|I2K0D9 CN....DWLFR.RYGA......E.....GLIDWXIV...........
tr|I3CUN9 AW....ADLCLAMSTY............QAQSR..............
tr|I3VQA4 AN....EYLSR.R..G......D.....IMINWCAI...........
tr|I4CBR6 AW....EDLQKAMALL......ESRRSRTEYHYDE............
tr|I7LIA9 YL....EDLKKLRKVL............EGEEYI.............
tr|I7LKX1 LE....EDFRIVGREL............AKAGGSPAEG.........
tr|J0UVA7 AW....RDLSLLRQLQ...........AQP.................
tr|J5SPB4 SN....AWLQE.RYGA......D.....GGIDWKSL...........
tr|J7R1V5 IN....DWLHNERGEK.............MIDWSVV...........
tr|J9E0C3 TW....RDLCSLQKRL......PDTVPAQARNKTDETN.........
tr|J9Z1B8 TN....NYLES.K..Q......I.....DMINW..............
tr|K0IF13 LE....ADLKKLANEI............KKEEGKH............
tr|K0KF39 AN....EWLIE.Q..G......E.....DPIDWSLV...........
tr|K1JXC9 AW....EDLTLFRRLI.....REAGIKLAPVHHTH............
tr|K1JYF3 AN....TWLAA.H..G......L.....KPVDWTI............
tr|K1LNP2 VN....HYLIQ.Q..G......K.....TPIQW..............
tr|K1ZRE1 ......EDFRILKDLV.................................
tr|K2A3B6 ER....EDFASLESGL............KSLVDKRSETTKESEQMHL
tr|K2B4F4 LI....RDMKRIPKLL............EKIKNENLNERTQSAA...
tr|K2C359 ......NDMKKLKDAY............DAAK...............
tr|K2CLQ5 FK....GDFQKLKTLL.................................
tr|K2DEQ6 LK....RDFVKIPEVL............DKVKNNKIDMFTEANNQNS
tr|K2E7J1 CN....NYLEE.H..E......M.....KAIDWR.............
tr|K2E9B4 MR....QDIQEVRSVL............DSL................
tr|K2ETW7 AY....TDFLIIKNIA............EQIVSG.............
tr|K2F640 EK....TDFVNLREIL............KKIQNDKQSLVNSEKNDKV
tr|K4KIT8 AN....RQLSQ.W..G......K.....PTVAWRR............
tr|K6GHM9 IN....NILIK.G..G......M.....KPINWNLN...........
tr|K7YPM8 AW....NDMLTLRCRL............DSIANKF............
tr|K8ZAC1 AN....QKLAD.S..K......R.....VPIQWQLP...........
tr|K9VTI9 PWRLTERDLKVVRKAS............QQLARTIKPVD........
tr|L0A9A2 ......DDIKTASKII.......GNKHKNLIDFM.............
tr|L0B4R8 TN....EWLIS.Q..K......R.....KPITWLIP...........
tr|L0B866 AN....EWLIN.H..R......K.....KPVIWQI............
tr|L0B8A6 AW....ADLCLIASLL............GK.................
tr|L0DFI2 AW....DDLQMLMKQM.........GLTPPTREKR............
tr|L0L178 QQ....KEMIDLNKKL...............WQKI...........
tr|L2GQC8 AN....KYLSS.H..L......R.....GAIDWKLS...........
tr|L2GX11 AN....EYLKK.N..G......E.....VEINW..............
tr|L5N8A0 IQ....QSLDDVKRHL............YKED...............
tr|L8HA23 AN....NLLKQ.R..T......LTGESKKPIEWQLD...........
tr|L9X1W2 IE....SDLRAALEEA.................................
tr|M0BKB2 ......EYVTALERAI...........ADCVDRDSS...........
tr|M0CBA6 ......RDLRVAREIA............EEA................
tr|M0MGS7 YE....GYLDAIAETL............AGVE...............
tr|M1E4Q4 TW....KDIQKVKQFI............EEN................
tr|M1LNF1 AN....RWLHL.N..K......K.....NTIKWFFS...........
tr|M1QKH3 FR....EDILKIKSLI............DSLR...............
tr|M1VCA8 AN....EYLQS.L..G......L.....EPIRWHAI...........
tr|M1Z9H8 YM....EDLIKLKKEI............KNL................
tr|M2XVU5 VN....DILKS.K..Q......E.....VPINW..............
tr|M3EFG6 ......TFIRR......................................
tr|M4YU22 ......REQLKLTMVM............IKEEFLP............
tr|M5ADY8 TN....NFLKK.I..G......R.....KTIFWNFW...........
tr|M5IVB6 AN....EWLKE.Q..G......V.....TPIQWFAT...........
tr|M6D8H9 VW....EDIKKVL...............ERLGWKRSG..........
tr|M7WR77 AN....EWLQQ.KYGP......D.....AGIEWTKL...........
tr|N0BBN4 LE....KDFEKIGSIL......K..KRRTLEDFMEL...........
tr|N1V4N6 FR....EGLRELLAEG......M..........................
tr|N1Z687 VYQ...NDLQVLKTYL.................................
tr|N2BLL9 AN....KALQD.M..G......K.....MPMDWRIV...........
tr|N9SHT1 IN....IFLKQ.K..N......Q.....SEINWNL............
tr|N9V498 .S....NFFRKVNSKL...........TKSIDFSMRKEK........
tr|Q025I0 AW....EDLKKVLHFV...............YD.............
tr|Q0C0Y4 AW....RDLLLIEKRL............GELE...............
tr|Q0EZR7 GW....QDLCLLADRY............RDLTQS.............
tr|Q12TN4 ......EDQHRFLDEN.............RYIWENE...........
tr|Q1AV32 ......QVVEDLRTAW............ERASGG.............
tr|Q1AYQ1 VW....KDIQNVYAEY...........QKALERRGAS..........
tr|Q1GY28 VN....AALRKLG..L............GEVQWGEP...........
tr|Q1N1I3 AN....RYLLE.Q..G......Q.....KPINWQIQ...........
tr|Q23W06 CN....EILVK.L..G......K.....EPIDWQV............
tr|Q3SA81 LE....KAFATLKSVL............DDMEN..............
tr|Q53CZ3 AN....DYLVQ.N..Q......R.....GAVDWNIN...........
tr|Q5DC15 AN....EYLKQ.H..N......I.....EPVDWTKL...........
tr|Q5WES9 IT....NDMRQLRAVL............DSLHEQG............
tr|Q6CAC0 AN....EWLEK.RYGP......E.....GIIKWGLE...........
tr|Q6F1M3 IN....TILVN.N..S......L.....EPIDWSK............
tr|Q6KIL8 VN....EILKA.N..N......L.....KQIDWNIF...........
tr|Q7VBS6 PYNLTCLDLREVNRKL....VELQFVPKTTSFKIPMSH........
tr|Q8TK31 ......IDVQKAFIDK............NRDLWQ.............
tr|Q9RTK9 TW....RDIREVAAVL......RGEKEASPVQGQFPAAPDS......
tr|Q9WR44 AN....NYFLK.R..G......E.....PIINWTI............
tr|R1ATM3 YY....NDLYKLKEVL............KKKVY..............
tr|R1E4B2 FD....QDFKTIKEIL......NKDKREGILKFI.............
tr|R1FRL1 AN....EYLEQ.K..G......F.....APIDWAAL...........
tr|R1GL12 VW....RHMLRMKAFL......LARSEYAGLCDQAI...........
tr|R2SFV7 DW....TNLGELLHTK......S..........................
tr|R2SXC9 DW....SSLGSLLKNS.............ELN...............
tr|R4KRR4 IQ....ADFKRLGVWL............QEILN..............
tr|R4VE87 AW....EDLLAVK...............RALQGAVA...........
tr|R5ARL3 ME....RDAEKLREHL.........VKAEKIVFSTTTGMV.......
tr|R5B4U8 ......TMRQDIVKLG............QYIKEGMA...........
tr|R5DX78 ......QDLANIKNEI..LADVDHSQDDNQVEYMNTS..........
tr|R5MPG0 VN....AILKK.N..G......S.....EEIKW..............
```

```
tr|R5PZ23  AW....HDLLALKEKM............NELTK..............
tr|R5QX00  AW....SDVLRLYKQM............NSEKES.............
tr|R5Z1D7  TN....EYLIS.H..G......Q.....KPINWIQT...........
tr|R6HGV3  AN....TLLAR.Y..G......E.....NPIMWQSV...........
tr|R6HL97  AN....TYLRK.I..G......Y.....APVDWTNV...........
tr|R6PFR8  RWLMK.KDLENVKNKL............EMLQEMD............
tr|R6PI86  MW....QDIQEIKRAY............DAIKD..............
tr|R6R865  AN....EFLKQ.H..N......R.....QEIKWVNN...........
tr|R7BZ83  AN....EWLQS.Q..G......A.....EPVHWLEF...........
tr|R7DQ64  TN....QYLIE.H..H......L.....EPIHWQN............
tr|R7DXQ1  VW....EDMLAVMERL......GMPISEKQRGYFLPRE.........
tr|R7I8P1  AW....EDMQSLRDRL......MDLGLYNDIYQEQTP..........
tr|R7IAS2  CW....HDLLFVKRTL............KAAEAA.............
tr|R7IHQ8  ......ADLERLHSVL............AAQ................
tr|R9ADN8  AD....EWIKE.RYGR.............GIDWMVQ...........
tr|R9LKN0  AN....DYLIA.H..H......E.....TPIEWTCS...........
tr|S0EV15  LV....ADIAAARQKV......IEAKREPKLTLF.............
tr|S0FG57  YN....NDVLMLKEVL............SSLDASN............
tr|S2J4F7  AN....AYLKE.H..G......N.....EEINWNCL...........
tr|S3JT40  AW....EDLKRFRARL............HELQAQG............
tr|S4NBY8  VD....ADSQRLKQLL............AKQSNGR............
tr|S5MFT9  IN....DLLVK.K..N......Q.....KAINWQL............
tr|S5RQ34  TN....KYLIS.K..N......I.....NPINWINK...........
tr|S6G4W1  AW....EDLKMLRAYI............ETHKIQVNT..........
tr|S9VE50  CN....EALVK.A..G......H.....TPIDWKLP...........
tr|T0AYQ1  AN....EYLNQ.K..G......I.....KEIDWSFL...........
tr|T0DRC9  IQ....NAHRE.I..Y......H.....KDFDFSL............
tr|T0LSQ5  FN....NVFFEIIKIL............KN.................
tr|T0MGQ8  AN....KYLIS.H..C......K.....KPIVWNL............
```
